# Anemia Acuity Effect on Transfusion Strategies in Acute Myocardial Infarction: A Secondary Analysis of the MINT Trial

**DOI:** 10.1001/jamanetworkopen.2024.42361

**Published:** 2024-11-01

**Authors:** François M. Carrier, Howard A. Cooper, Gerard T. Portela, Marnie Bertolet, Gilles Lemesle, Micah Prochaska, Sarang Kim, John H. Alexander, Ian Crozier, Gregory Ducrocq, Alexandre S. Quadros, Akshay Bagai, Marianna Dracoulakis, Mina Madan, Maria M. Brooks, Jeffrey L. Carson, Paul C. Hébert

**Affiliations:** 1 Department of Anesthesiology and Department of Medicine, Critical Care Division, Centre Hospitalier de l’Université de Montréal, Montréal, Québec, Canada; 2 Department of Anesthesiology and Pain Medicine, Université de Montréal, Montréal, Québec, Canada; 3 Department of Cardiology, Westchester Medical Center, Valhalla, New York; 4 Department of Epidemiology, University of Pittsburgh, Pittsburgh, Pennsylvania; 5 Heart and Lung Institute, University Hospital of Lille, CHU Lille, Lille, France; 6 Université de Lille, F-59000, Lille, France; 7 French Alliance for Cardiovascular Trials, Paris, France; 8 Institut Pasteur of Lille, Inserm U1011-EGID, Lille, France; 9 Department of Medicine, University of Chicago, Chicago, Illinois; 10 Department of Medicine, Rutgers Robert Wood Johnson Medical School, New Brunswick, New Jersey; 11 Duke Clinical Research Institute, Division of Cardiology, Duke University, Durham, North Carolina; 12 Department of Cardiology, Christchurch Hospital, Christchurch, New Zealand; 13 Université de Paris, Assistance Publique-Hôpitaux de Paris, French Alliance for Cardiovascular Trials, INSERM U1148, Paris, France; 14 Department of Interventional Cardiology, Instituto de Cardiologia do Rio Grande do Sul, Porto Alegre, Brazil; 15 Terrence Donnelly Heart Center, St Michael’s Hospital, University of Toronto, Toronto, Ontario, Canada; 16 Clinical Center Research–Hospital da Bahia, Dasa, Brazil; 17 Division of Cardiology, Schulich Heart Program, Sunnybrook Health Sciences Centre, Toronto, Ontario, Canada; 18 Temerty Faculty of Medicine, University of Toronto, Toronto, Ontario, Canada; 19 Epidemiology Data Center, Faculty in Epidemiology and Biostatistics, School of Public Health, University of Pittsburgh, Pittsburgh, Pennsylvania; 20 Innovation and Health Evaluation Hub, Centre de Recherche du CHUM, Montréal, Québec, Canada; 21 Department of Medicine, Université de Montréal, Montréal, Québec, Canada

## Abstract

**Importance:**

In patients with acute myocardial infarction (MI), limited physiologic adaptation to acute anemia might lead to greater benefit from a liberal red blood cell (RBC) transfusion strategy. Data on such a possible benefit are lacking.

**Objectives:**

To compare acute anemia with chronic anemia and post-MI outcomes and estimate the differential effect of a restrictive RBC transfusion strategy compared with a liberal strategy on post-MI outcomes according to anemia acuity.

**Design, Setting, and Participants:**

A prespecified subgroup analysis of the Myocardial Ischemia and Transfusion (MINT) multicenter randomized clinical trial was conducted in 126 hospitals in 6 countries between April 26, 2017, and April 14, 2023, with 30-day follow-up and blinded adjudication of the primary outcome. The analysis included 3144 of 3504 MINT participants (89.7%) with acute MI, a hemoglobin (Hb) level less than 10 g/dL at randomization, and a first Hb measurement available on the day of or the day following hospital admission.

**Intervention:**

The MINT trial randomized participants to a restrictive (Hb <7-8 g/dL) or liberal (Hb <10 g/dL) RBC transfusion strategy. Acute anemia was defined as having a first Hb value greater than 13 g/dL (men) or 12 g/dL (women), or as having a decrease greater than or equal to 2 g/dL between the first Hb measurement and measurement at randomization. Other Hb levels were categorized as chronic anemia.

**Main Outcomes and Measures:**

The primary outcome was a composite of death or recurrent MI up to 30 days after randomization. Secondary outcomes were death, recurrent MI, cardiac death, heart failure, pulmonary complications, and major bleeding events. Intention-to-treat analysis was performed.

**Results:**

Among 3144 included participants (mean [SD] age, 72.3 [11.6] years; 1715 [54.5%] male; 1307 [41.6%] with type 1 MI), 1078 [34.3%]) had acute anemia. Acute anemia was associated with an increased risk of death or recurrent MI (adjusted risk ratio, 1.25; 95% CI, 1.05-1.48). The effect of a restrictive RBC transfusion strategy compared with a liberal strategy was similar for participants with either acute or chronic anemia for all outcomes.

**Conclusions and Relevance:**

In this secondary analysis of the MINT trial, acute anemia was associated with less favorable post-MI outcomes than chronic anemia but did not modify the effects of the randomized transfusion strategy. In patients with anemia and MI, the acuity of anemia should not influence the choice of transfusion trigger.

**Trial Registration:**

ClinicalTrials.gov Identifier: NCT02981407

## Key Points

**Question:**

Does acute anemia compared with chronic anemia modify the effect of red blood cell (RBC) transfusion strategies on a composite 30-day outcome of death or recurrent myocardial infarction (MI) in adults with acute MI?

**Findings:**

In this secondary analysis of a randomized clinical trial, acute anemia compared with chronic anemia was associated with a statistically significant 25% higher risk of a composite outcome of 30-day death or MI. However, anemia acuity did not significantly modify the effect of RBC transfusion strategies on the risk of this composite outcome.

**Meaning:**

The findings of this study suggest that in patients with anemia who experience MI, the choice of RBC transfusion strategy should not be based on anemia acuity.

## Teaser

This secondary analysis of a randomized clinical trial compares outcomes following myocardial infarction among patients with acute vs chronic anemia and estimates the effect of a liberal vs restrictive red blood cell transfusion strategy according to anemia acuity.

## Introduction

In patients with acute myocardial infarction (MI), anemia has a prevalence ranging from 10% to 43% and has been associated with a 30% increase in 30-day mortality.^1,2,3,4,5^ Before publication of the Myocardial Ischemia and Transfusion (MINT) trial, there was a lack of high-quality trial data regarding the effect of different red blood cell (RBC) transfusion strategies in patients with anemia and acute MI.^6,7,8,9,10,11^ In the MINT trial, 3504 patients with anemia and acute MI were randomized to either a liberal or restrictive RBC transfusion strategy. The risk of 30-day death or MI was higher in the restrictive strategy (16.9%) group than in the liberal strategy (14.5%) group. Despite not being statistically significant, these results suggested possible harm from a restrictive strategy. This primary finding did not consider the time course of the development of anemia, which may modify both the prognosis after MI and the response to transfusions.^12^

Chronic anemia is often present in patients at the time of MI admission because of comorbidities such as advanced age, occult bleeding, kidney disease, cancer, nutritional deficiencies, and chronic inflammatory conditions. Alternatively, acute anemia may be present on admission when abrupt blood loss precipitates MI, or it may develop during MI hospitalization because of blood loss, acute inflammation, or hemodilution.^13,14^ In patients with chronic anemia, compensatory mechanisms, such as increases in 2,3-diphosphoglycerate levels, cardiac output, and coronary and cerebral blood flow, may have had time to become established, whereas this is less likely in those with acute anemia.^12,15,16^ Resulting physiologic differences related to anemia acuity might influence the effect of various RBC transfusion strategies on outcomes after acute MI.

We therefore performed a preplanned secondary analysis of MINT to assess the outcomes of participants with acute vs chronic anemia and estimate whether there was a differential effect of a restrictive RBC transfusion strategy compared with a liberal strategy on post-MI outcomes in participants with acute or chronic anemia. We hypothesized that participants with acute anemia at the time of randomization would have fewer comorbidities than patients with chronic anemia, and consequently better post-MI outcomes. We also hypothesized that a liberal transfusion strategy compared with a restrictive one would be of greater benefit in patients with acute anemia compared with chronic anemia.

## Methods

### Design and Population

This is a secondary analysis of the MINT trial reporting prespecified subgroup (effect modification) analyses of the randomized intervention. The main trial protocol and the statistical analytical plan are available in Supplement 1. Each site’s institutional review board approved the study, and local research staff obtained informed consent from all participants before inclusion. We reported this secondary analysis using the Consolidated Standards of Reporting Trials (CONSORT) reporting guideline and interpreted the credibility of our results using the Instrument to assess the Credibility of Effect Modification Analyses (ICEMAN) framework.^17,18^

Briefly, the MINT trial randomized participants who were hospitalized with type 1, 2, 4b, or 4c MI and a hemoglobin (Hb) level less than 10 g/dL during hospitalization (to convert to grams per liter, multiply by 10) in a 1:1 ratio to either a restrictive (RBC transfusion permitted for an Hb level <8 g/dL and strongly encouraged for an Hb level <7 g/dL) or a liberal (RBC transfusion required for any Hb level <10 g/dL) RBC transfusion strategy.^11,19^ Patients were excluded if they had uncontrolled bleeding, were receiving palliative treatment, were already scheduled for cardiac surgery, or declined blood transfusion. For this secondary analysis, we included participants in whom a first Hb measurement was available prior to any transfusion on the day of or on the day after admission of the index hospitalization.

### Acute and Chronic Anemia Definitions

The exposure of interest was acute (acquired during the hospitalization) vs chronic anemia at randomization. Since there is no established definition of acute anemia in this context, we internally developed a definition through expert consensus and prior to conducting any analyses. We considered participants to have acute anemia if they met the following criteria: having an admission Hb level above the World Health Organization (WHO) anemia definition thresholds (13 g/dL in men, 12 g/dL in women) followed by a decrease to the randomization threshold (<10 g/dL) or having an admission Hb value below the WHO thresholds followed by a decrease of 2 g/dL or more prior to randomization. The remaining participants, those with an admission Hb level below the WHO thresholds and a decrease of Hb less than 2 g/dL prior to randomization, were considered to have chronic anemia. When only a single Hb measurement was available prior to randomization, participants were classified as having chronic anemia. An unobserved decrease of 1 g/dL was assumed for each RBC unit received between hospital admission and randomization.

### Outcomes

All outcomes were assessed at 30 days after randomization. The primary outcome was a composite of death or recurrent MI, as in the main study.^11^ Secondary outcomes were death, recurrent MI, cardiac death, heart failure, a composite of pulmonary complications (transfusion-related acute lung injury [TRALI], transfusion-associated circulatory overload [TACO], pneumonia, and acute respiratory failure), and major bleeding. Recurrent MI was adjudicated by an independent committee blinded to treatment allocation. These outcomes were prespecified in the main trial except the composite outcome of pulmonary complications.

### Data Source, Collection, and Management

All data were collected in the context of the MINT trial.^11,19^ Inclusion in this secondary analysis was based on the availability of a first Hb measurement from either the enrolling hospital or the referent hospital if the participant had been transferred to the enrolling institution. Participant’s biological sex was based on hospital records. Electrocardiograms, Hb, and troponin measurements were required before randomization (within 24 hours), 12 hours after randomization (troponin levels only), and daily up to 3 days after randomization. Other Hb measurements from the hospital admission to randomization were obtained at clinical discretion.

### Statistical Analysis

The sample size calculation of the main trial has been reported.^11,19^ We reported baseline variables at the time of randomization and crude distribution of study outcomes using standard measures of central tendency and dispersion, both by anemia acuity (acute and chronic anemia) and group allocation (liberal and restrictive RBC transfusion strategy). We assumed that no unobserved event occurred for those with incomplete 30-day follow-up (n = 50). We estimated the crude and adjusted association between anemia acuity and outcomes using mixed-effects log-binomial regressions with random effects for sites. Models were adjusted for the following potential confounders: baseline characteristics (age, sex, smoking status, Hb level at time of randomization), anemia-related comorbidities (cancer, kidney failure, and diabetes), and MI-related characteristics at the time of randomization (number of anticoagulants or antiplatelets prescribed, and MI type). We selected these variables based on their clinical potential associations with both anemia acuity and our outcomes. Missing values for smoking status (174 of 3144 [5.5%]) and medication data (1 [<0.1%]) were addressed using mean imputation. We report risk ratios (RRs), 95% CIs, and *P* values for all outcomes.

We estimated the intention-to-treat intervention effect (restrictive vs liberal transfusion strategies) on every outcome in the full sample and within each anemia acuity group. We used multivariable mixed-effects log-binomial regressions with a random effect for clinical sites, adjusted for the same prognostic factors and potential confounders mentioned above. We included a variable for anemia acuity, the randomized intervention, and an interaction term between the 2 to test whether anemia acuity modified the intervention effect. We reported RRs with 95% CIs and the *P* value for the interaction test (multiplicative scale [RR]). We also reported the relative excess risk due to the interaction with 95% clustered bootstrap CI with 1000 resamples to express the effect modification on the additive scale.^20^

With unpaired testing, the α level was set at .05. No adjustment was made for multiple comparisons. All analyses were conducted using SAS, version 9.4 (SAS Institute Inc).

We performed 4 sensitivity analyses. For the first, we used an alternative definition of acute anemia to only include patients with an Hb level at admission above the WHO threshold for anemia, thus creating a group with purely hospital-acquired acute anemia. In this modified definition, all patients with WHO-defined anemia at admission were classified as having chronic anemia. In a second sensitivity analysis, we restricted our analysis of pulmonary complications to only transfusion-related pulmonary complications (TACO and TRALI together). In a third sensitivity analysis, we restricted all analyses to participants randomized at least 3 days after hospital admission to allow sufficient time for acute anemia to develop and thus limit potential time bias. In a last sensitivity analysis, we excluded participants who received any RBC transfusion prior to randomization to evaluate whether our results were sensitive to the assumed Hb decrease concealed by transfusion.

## Results

### Participants

**Figure 1.**
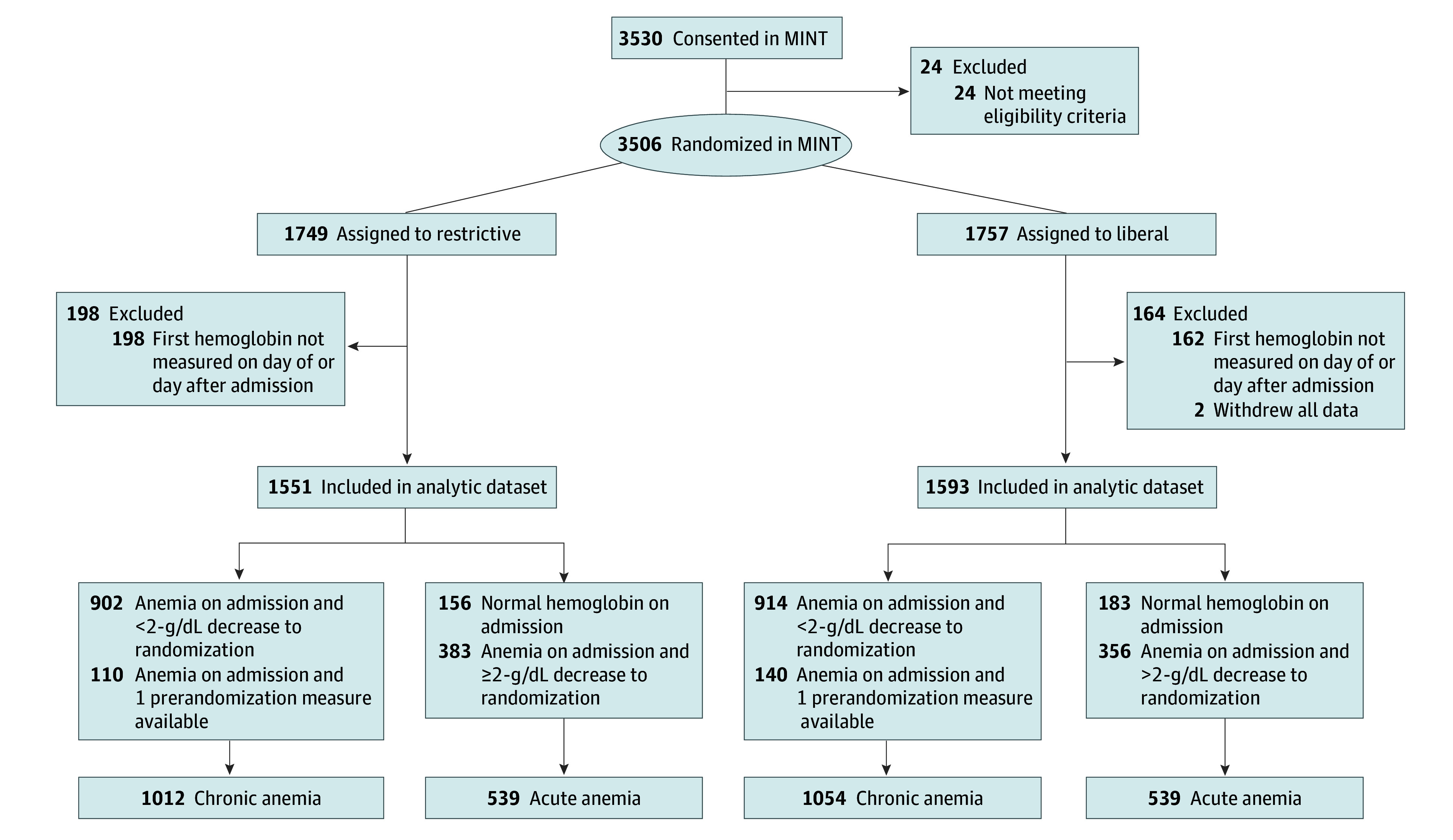
Patient Inclusion in Myocardial Ischemia and Transfusion (MINT) Acute Anemia Secondary Analysis Exclusions for hemoglobin not measured on the day of or day after admission were specific to this secondary analysis of the MINT trial. To convert hemoglobin to grams per liter, multiply by 10.

**Figure 2.**
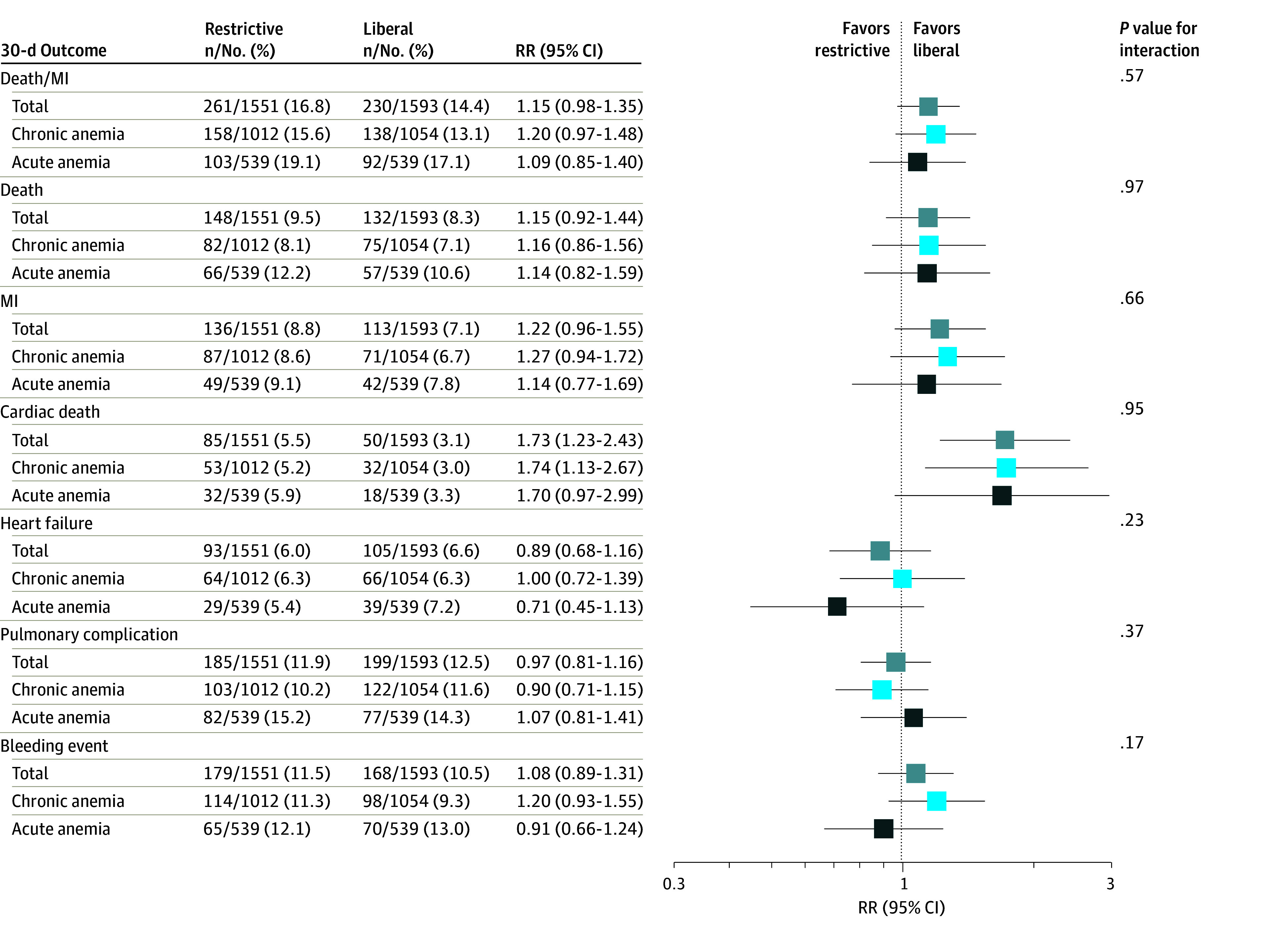
Effect of a Restrictive Strategy Compared With a Liberal Strategy on 30-Day Outcomes Results are presented for the full sample and in each group of anemia acuity. Risk ratio (RR) values greater than 1 favor a liberal strategy. Pulmonary complications were transfusion-related acute lung injury, transfusion-associated circulatory overload, pneumonia, and acute respiratory failure. Models were adjusted for age, sex, smoking status (never, ever, current, or unknown), anemia-related comorbidities (cancer, chronic kidney disease, or diabetes), baseline hemoglobin (grams per deciliter), myocardial infarction (MI) type (type 1, type 2, or other), number of anticoagulants (warfarin, heparin, or other) and antiplatelets (aspirin, P2Y12 inhibitor, or glycoprotein IIb/IIIa inhibitor) and included a random effect for clinical site.

Of the 3504 participants enrolled in the MINT trial from April 26, 2017, through April 14, 2023, 3144 individuals (89.7%) from 126 of 144 sites in the full trial (87.5%) were included in this secondary analysis (Figure 1). Overall, their mean (SD) age was 72.3 (11.6) years, 1715 (54.5%) were male, 1429 (45.5%) were female, and 1307 (41.6%) had type 1 MI. Baseline characteristics of included participants are reported in Table 1 and those of excluded participants are reported in eTable 1 in Supplement 2. The crude distributions of each outcome are presented in Figure 2.

**Table 1.** Baseline Characteristics According to Anemia Acuity and Randomized Intervention

Characteristic (N = 3144)	Acute anemia (n = 1078)			Chronic anemia (n = 2066)		
	Total	Liberal (n = 539)	Restrictive (n = 539)	Total	Liberal (n = 1054)	Restrictive (n = 1012)
Demographic, No. (%)						
Age, mean (SD), y	72.0 (11.4)	71.8 (11.5)	72.2 (11.3)	72.4 (11.7)	72.4 (11.6)	72.4 (11.7)
Sex						
Male	598 (55.5)	288 (53.4)	310 (57.5)	1117 (54.1)	562 (53.3)	555 (54.8)
Female	480 (44.5)	251 (45.6)	229 (42.5)	949 (45.9)	492 (46.7)	457 (45.2)
BMI, mean (SD)^a^	28.5 (7.1)	28.5 (7.1)	28.4 (7.1)	28.7 (7.1)	28.9 (7.2)	28.4 (6.9)
Comorbidities, No. (%)						
Smoking status^a^						
Never	390 (38.9)	193 (38.7)	197 (39.2)	804 (40.9)	412 (40.9)	392 (40.8)
Former	412 (41.1)	204 (40.9)	208 (41.4)	871 (44.3)	446 (44.3)	425 (44.3)
Current	200 (20.0)	102 (20.4)	98 (19.5)	293 (14.9)	150 (14.9)	143 (14.9)
History of MI	296 (27.5)	141 (26.2)	155 (28.8)	727 (35.2)	358 (34.0)	369 (36.5)
Most recent ejection fraction, mean (SD)^a^	46.8 (13.7)	47.0 (13.5)	46.7 (13.8)	47.7 (13.7)	47.5 (14.0)	47.9 (13.3)
History of stroke	190 (17.6)	97 (18.0)	93 (17.3)	361 (17.5)	174 (16.5)	187 (18.5)
History of atrial fibrillation	227 (21.1)	115 (21.3)	112 (20.8)	580 (28.1)	289 (27.4)	291 (28.8)
History of peripheral artery disease	197 (18.3)	89 (16.5)	108 (20.0)	442 (21.4)	245 (23.2)	197 (19.5)
History of kidney failure	416 (38.6)	203 (37.7)	213 (39.5)	1018 (49.3)	526 (49.9)	492 (48.6)
History of diabetes	511 (47.4)	259 (48.1)	252 (46.8)	1179 (57.1)	593 (56.3)	586 (57.9)
History of hypertension	878 (81.4)	441 (81.8)	437 (81.1)	1792 (86.7)	919 (87.2)	873 (86.3)
History of hypercholesterolemia	673 (62.4)	332 (61.6)	341 (63.3)	1365 (66.1)	712 (67.6)	653 (64.5)
History of chronic obstructive pulmonary disease or asthma	240 (22.3)	115 (21.3)	125 (23.2)	507 (24.5)	257 (24.4)	250 (24.7)
History of cancer	236 (21.9)	114 (21.2)	122 (22.6)	467 (22.6)	230 (21.8)	237 (23.4)
MI characteristic at randomization, No. (%)						
MI type						
Type 1	505 (46.8)	256 (47.5)	249 (46.2)	802 (38.8)	405 (38.4)	397 (39.2)
Type 2	550 (51.0)	272 (50.5)	278 (51.6)	1206 (58.4)	626 (59.4)	580 (57.3)
Other	23 (2.1)	11 (2.0)	12 (2.2)	58 (2.8)	23 (2.2)	35 (3.5)
Performed angiogram^a^	593 (55.0)	286 (53.1)	307 (57.0)	946 (45.8)	476 (45.2)	470 (46.5)
Percutaneous intervention	422 (39.1)	201 (37.3)	221 (41.0)	513 (24.8)	264 (25.0)	249 (24.6)
Coronary artery bypass graft surgery	5 (0.5)	3 (0.6)	2 (0.4)	6 (0.3)	4 (0.4)	2 (0.2)
Days from first symptoms to hospital admission, median (IQR)^a^	0 (0-0)	0 (0-0)	0 (0-0)	0 (0-1)	0 (0-1)	0 (0-1)
Days from hospital admission to randomization, median (IQR)	5 (3-8)	5 (3-8)	5 (3-8)	2 (1-4)	2 (1-3)	2 (1-4)
Anticoagulants at randomization, No. (%)^a^						
Cumulative No. of different antiplatelets or anticoagulants						
0	56 (5.2)	30 (5.6)	26 (4.8)	159 (7.7)	76 (7.2)	83 (8.2)
1	146 (13.6)	72 (13.4)	74 (13.7)	336 (16.3)	158 (15.0)	178 (17.6)
2	267 (24.8)	141 (26.2)	126 (23.4)	666 (32.2)	364 (34.5)	302 (29.8)
≥3	608 (56.5)	295 (54.8)	313 (58.1)	905 (43.8)	456 (43.3)	449 (44.4)
Antiplatelets						
Aspirin	901 (83.7)	441 (82.0)	460 (85.3)	1685 (81.6)	879 (83.4)	806 (79.6)
P2Y12 inhibitor	632 (58.7)	302 (56.1)	330 (61.2)	1067 (51.6)	541 (51.3)	526 (52.0)
Glycoprotein IIb/IIIa inhibitor	42 (3.9)	21 (3.9)	21 (3.9)	41 (2.0)	20 (1.9)	21 (2.1)
Anticoagulants						
Unfractionated or low molecular–weight heparin	849 (78.8)	430 (79.9)	419 (77.7)	1357 (65.7)	681 (64.6)	676 (66.8)
Warfarin	44 (4.1)	24 (4.5)	20 (3.7)	85 (4.1)	52 (4.9)	33 (3.3)
Any other anticoagulant	97 (9.0)	48 (8.9)	49 (9.1)	220 (10.6)	118 (11.2)	102 (10.1)
Other characteristics prior to randomization, No. (%)						
Any RBC transfusion	537 (49.8)	274 (50.8)	263 (48.8)	580 (31.9)	300 (32.8)	280 (31.0)
No. of RBC units transfused, median (IQR)3^a^	0 (0-2)	1 (0-2)	0 (0-2)	0 (0-1)	0 (0-1)	0 (0-1)
Hemoglobin level, mean (SD)	8.5 (0.9)	8.5 (0.9)	8.5 (0.8)	8.7 (0.8)	8.7 (0.8)	8.7 (0.8)
Clinical bleed	235 (21.8)	113 (21.0)	122 (22.6)	179 (9.9)	77 (8.4)	102 (11.3)
Critical care at randomization^b^	599 (55.6)	296 (54.9)	303 (56.2)	871 (48.0)	435 (47.6)	436 (48.3)
Dialysis	121 (11.2)	60 (11.1)	61 (11.3)	237 (13.1)	126 (13.8)	111 (12.3)
Mechanical ventilation	298 (27.6)	156 (28.9)	142 (26.3)	128 (7.0)	51 (5.6)	77 (8.5)

Abbreviations: BMI body mass index (calculated as weight in kilograms divided by height in meters squared); MI, myocardial infarction; RBC, red blood cell.

^a^ The following variables had missing values that were not included in the denominator of the percentages or the calculations of the means (SDs): BMI (n = 122), smoking status (n = 174), most recent ejection fraction (n = 857), performed angiogram (n = 1), days from symptoms to hospital admission (n = 413), anticoagulants at randomization (n = 1), and number of RBC units transfused (n = 11).

^b^ Hospitalization in a critical care unit or a coronary care unit.

### Acute and Chronic Anemia

Acute anemia was observed in 34.3% (n = 1078) of included participants and chronic anemia in 65.7% (n = 2066). The median time from admission to randomization was 5 (IQR, 3-8) days for patients with acute anemia and 2 (IQR, 1-4) days for those with chronic anemia (Table 1). The median (first and third quartiles) Hb decrease from admission to randomization was 3.6 (IQR, 2.6-5.3) g/dL in the acute anemia group and 0.7 (IQR, 0.2-1.3) g/dL in the chronic anemia group (among the 1816 participants with chronic anemia [87.9%] and at least 2 Hb measurements prior to randomization). Participants with acute anemia had fewer chronic comorbidities (eg, atrial fibrillation, chronic kidney disease, and diabetes), were more often current smokers, more frequently had a type 1 MI, were more likely to have undergone a percutaneous intervention and be treated with a higher number of antithrombotic agents, and had a more severe clinical course prior to randomization (more bleeding episodes, requirements for at least 1 transfusion, use of mechanical ventilation, and days in critical care units). Participants with acute anemia and those with chronic anemia received a similar number of RBC units both before and after randomization (Table 1; eTable 2 in Supplement 2).

### Association Between Acute Anemia and Outcomes

The crude and adjusted associations between anemia acuity and outcomes are reported in Table 2. After adjustment for potential confounders, acute anemia was associated with a 25% higher incidence of death or recurrent MI (RR, 1.25; 95% CI, 1.05-1.48), a 47% higher incidence of death (RR, 1.47; 95% CI, 1.16-1.86), and a 36% higher incidence of pulmonary complications (RR, 1.36; 95% CI, 1.12-1.66) compared with chronic anemia at 30 days after randomization (Table 2; eTable 3 in Supplement 2). Risks of recurrent MI, heart failure, cardiac death, and major bleeding were similar among patients with acute anemia and chronic anemia.

**Table 2.** Association Between Acute Anemia and 30-Day Outcomes^a^

30-d Outcome	RR (95% CI)	
	Crude	Adjusted^b^
Composite of death or recurrent MI	1.25 (1.05-1.47)	1.25 (1.05-1.48)
Death	1.48 (1.18-1.86)	1.47 (1.16-1.86)
Recurrent MI	1.10 (0.86-1.41)	1.10 (0.85-1.42)
Cardiac death	1.12 (0.80-1.58)	1.03 (0.72-1.46)
Heart failure	1.00 (0.75-1.33)	1.02 (0.76-1.36)
Pulmonary complications^c^	1.33 (1.10-1.61)	1.36 (1.12-1.66)
Major bleeding episode	1.23 (1.00-1.51)	1.20 (0.97-1.49)

Abbreviations: MI, myocardial infarction; RR, risk ratio.

^a^ Acute anemia (n = 1078) is compared with chronic anemia (n = 2066).

^b^ The models were adjusted for randomized transfusion strategy, demographic characteristics (age, sex, smoking [never, ever, current, unknown]), and anemia-related comorbidities (cancer, kidney failure, diabetes), prerandomization characteristics (baseline hemoglobin [grams per deciliter]), number of anticoagulants (warfarin, heparin, other) and antiplatelets (aspirin, P2Y12 inhibitor, glycoprotein), and MI type (type 1, type 2, other) with a random effect for site.

^c^ Pulmonary complications were transfusion-related acute lung injury, transfusion-associated circulatory overload, pneumonia, or acute respiratory failure.

### RBC Transfusion Strategy Effect Modification by Anemia Acuity

The estimated effects of the restrictive transfusion strategy compared with the liberal one in the full sample as well as in subgroups of anemia acuity are reported in Figure 2. There was no differential effect of transfusion strategy with respect to anemia acuity for any outcome on either the RR or the additive scale (Figure 2; eTable 4 and eTable 5 in Supplement 2). For the primary composite outcome of death or recurrent MI, the effect of a restrictive strategy compared with a liberal one had a RR of 1.20 (95% CI, 0.97-1.48) in the chronic anemia stratum and an RR of 1.09 (95% CI, 0.85-1.40) in the acute anemia stratum (*P* = .57 for interaction).

### Sensitivity Analyses

When using the alternative definition of acute anemia, 339 participants (10.8%) were classified as having pure hospital-acquired acute anemia. The association between acute anemia and the primary outcome became null (adjusted RR, 0.95; 95% CI, 0.72-1.26), and the point estimates for death and pulmonary complications became nonsignificant (eTable 3 in Supplement 2). Results of the effect modification analyses were similar (eFigure 1 in Supplement 2). The effect of the transfusion strategy on transfusion-related pulmonary complications only (TACO and TRALI) was not modified by anemia acuity (eTable 5 in Supplement 2), although the risk of this outcome was lower in the restrictive group. The results of our sensitivity analyses restricted to participants randomized 3 days or more after hospital admission and those who did not receive a transfusion prior to randomization are reported in eTable 6, eTable 7, eFigure 2, and eFigure 3 in Supplement 2. All results were similar to the main analyses (eTables 6 and 7, eFigures 2 and 3 in Supplement 2).

## Discussion

In this planned subgroup analysis of the MINT trial, we observed that one-third of the participants had acute anemia. These participants had a more severe acute clinical course than patients with chronic anemia and had a higher risk of short-term adverse outcomes, even after adjustment for potential confounders. However, we did not find a differential effect of the randomized RBC transfusion strategy according to anemia acuity for any 30-day outcome

In previous studies, anemia at the time of hospital admission for acute MI or anemia at hospital discharge, either hospital-acquired (acute) or present at admission (chronic), was associated with worse short- and long-term all-cause or cardiovascular mortality.^5,14,21,22^ One study that compared post-MI outcomes in patients with acute hospital-acquired anemia with those in patients with chronic anemia observed similar poor 1-year cardiovascular outcomes in both conditions.^14^ In contrast and contrary to our a priori hypothesis, we observed that acute anemia prior to randomization, using our internally developed definition, was independently associated with a higher risk of 30-day adverse outcomes. When we used our alternative definition of pure hospital-acquired anemia as others did, we did not observe any significant difference in outcomes between acute and chronic anemia.^5,14,21,22^ This may be explained by a lack of power of our sensitivity analyses, the prevalence of acute anemia decreasing from 34.3% to 10.8%, or by the fact that different definitions capture different constructs. Nonetheless, the directionality of most of our secondary outcome point estimates did not change. In contrast to one study,^5^ we did not observe in any analysis that chronic anemia was associated with worse outcomes, possibly because our study was conducted in a clinical trial setting and a decade later, thus including a different study population. In addition, the comparison between restrictive and liberal transfusion strategies on any outcomes was not modified by the presence of acute or chronic anemia.

Physiologic adaptative mechanisms to anemia, such as increases in cardiac output, 2,3-diphosphoglycerate levels (decreasing Hb oxygen–binding affinity), tissue oxygen extraction, and redistribution of regional blood flow in favor of coronary and cerebral circulation, may not have sufficient time to occur and sustain tissue oxygen delivery in acute anemia.^23^ In the context of acute MI, the additional myocardial oxygen consumption caused by the required acute increase in cardiac output may further worsen clinical outcomes. We thus hypothesized that a more liberal RBC transfusion strategy after acute MI might improve tissue oxygen delivery (including myocardial oxygen delivery), reduce tissue hypoxia, and improve outcomes, especially in patients with acute anemia. However, our findings did not support this hypothesis. Acute anemia was not associated with an increased risk of recurrent MI, and the transfusion strategy did not have a larger effect on the outcomes in patients with MI and acute anemia.^12^

Our findings suggest that acute anemia was a marker of a more complex and morbid clinical course. Patients with acute anemia had evidence of increased acute blood loss, hemodynamic instability, and requirement for critical care, including mechanical ventilation, prior to randomization.^24,25,26,27^ The associations we observed between anemia acuity and mortality could be the effect of acutely occurring anemia itself, with its limited time for physiologic adaptation and worse tissue hypoxia, but was likely more the effect of the more severe underlying disease. In this context, a liberal RBC transfusion strategy did not have a larger effect in these patients. This strategy either did not provide any additional oxygen delivery benefit because of unimportant or ineffective differences in the adaptative mechanisms between acute and chronic anemia or provided oxygen delivery benefits that were countered by harm from transfusions administered to acutely ill patients. In prior studies involving critically ill patients with acute multisystemic diseases or acute bleeding, a restrictive RBC transfusion strategy resulted in either similar or lower hospital mortality while requiring fewer RBC transfusions.^28,29,30^ Shared characteristics of multisystem organ dysfunction between patients with acute anemia in our study and other critically ill populations may explain our observations. Whether there is a larger benefit of a liberal transfusion strategy in patients with acute anemia, acute MI, and less severe illness will require further study.

### Limitations

The current study has several limitations. Our definition of acute anemia was empirical and limited by the absence of Hb levels prior to hospital admission. The classification of acute vs chronic anemia was based on Hb measurements made at only 2 time points (admission and randomization). Some patients were classified as having chronic anemia based on only 1 Hb measurement, and some patients who developed acute anemia prior to admission may have been classified as having chronic anemia. However, our definition was physiologically based and included different scenarios of acute anemia, including acute on chronic anemia. Despite this, and as suggested by our sensitivity analyses, our findings may not be transportable to other definitions of acute anemia, including acute anemia that developed prior to admission. Interaction testing has relatively low statistical power, and we may not have detected a differential effect while some existed. We did not adjust for multiple comparisons, increasing the risk of type I error regarding the association between acute anemia and outcomes. In addition, while excluded patients slightly differed from included patients, the number of excluded patients was small and likely did not have a substantial effect on the results.

## Conclusions

In this prespecified secondary analysis of the MINT trial, we observed that participants with acute anemia at the time of randomization had a higher risk of adverse outcomes at 30 days than those with chronic anemia. The effect of a restrictive transfusion strategy compared with a liberal one was not modified by anemia acuity. Therefore, anemia acuity did not appear to explain the potential harm of a restrictive RBC transfusion strategy observed in the MINT trial. In patients with anemia and MI, clinicians should not be guided by the acuity of the anemia in selecting a transfusion strategy.

## Notes

Supplement 1.
Trial Protocol and Statistical Analytical Plan

Supplement 2. **eTable 1.** Participants’ Characteristics Included and Excluded From the Sub-Study **eTable 2.** Distribution of RBC Transfusions From Randomization to Hospital Discharge **eTable 3.** Association Between Acute Anemia and 30-Day Outcomes Using an Alternate Definition of Acute and Chronic Anemia **eTable 4.** Relative Excess Risk Due to Interaction (RERI) for 30-Day Outcomes **eTable 5.** Distribution, Association With, and Intervention Effect on 30-Day Transfusion-Related Pulmonary Complications **eTable 6.** Association Between Acute Anemia and 30-Day Outcomes, Restricted to Participants Hospitalized for 3 Days or More at Time of Randomization **eTable 7.** Association Between Acute Anemia and 30-Day Outcomes, Restricted to Participants **eFigure 1.** Effect of a Restrictive Strategy, Compared to a Liberal Strategy, on 30-Day Outcomes Overall and Stratified by Anemia Acuity, Using an Alternate Definition of Acute and Chronic Anemia **eFigure 2.** Effect of a Restrictive Strategy, Compared to a Liberal Strategy, on 30-Day Outcomes Overall and Stratified by Anemia Acuity, Restricted to Participants Hospitalized for 3 Days or More at Time of Randomization **eFigure 3.** Effect of a Restrictive Strategy, Compared to a Liberal Strategy, on 30-Day Outcomes Overall and Stratified by Anemia Acuity, Restricted to Participants Who Did Not Receive RBCs Prior to Randomization

Supplement 3. **Nonauthor Collaborators.** MINT Investigators

Supplement 4.
Data Sharing Statement
