# Supplementary material for: Anemia Acuity Effect on Transfusion Strategies in Acute Myocardial Infarction: A Secondary Analysis of the MINT Trial
Source: JAMA Netw Open. 2024 Nov 1;7(11):e2442361. doi: 10.1001/jamanetworkopen.2024.42361 (PMC11530937; doi:10.1001/jamanetworkopen.2024.42361)
Supplement: Supplement 1. — Trial Protocol and Statistical Analytical Plan [file jamanetwopen-e2442361-s001.pdf]

MINT

MYOCARDIAL ISCHEMIA AND TRANSFUSION

Jeffrey L Carson, MD
Study Chair
Principal Investigator, Clinical Coordinating Center
Rutgers Robert Wood Johnson Medical School

Maria Mori Brooks, PhD
Principal Investigator, Data Coordinating Center
University of Pittsburgh

TABLE OF CONTENTS

A INTRODUCTION 8

A1 STATEMENT OF COMPLIANCE 8

A2 STUDY ABSTRACT 9

A3 PRIMARY HYPOTHESIS 9

A4 PURPOSE OF THE STUDY PROTOCOL 10

A5 SCHEMATIC OF STUDY DESIGN 11

A6 KEY ROLES 12

B BACKGROUND 13

B1 PRIOR LITERATURE AND STUDIES 13

B2 RATIONALE FOR THIS STUDY: EQUIPOISE 17

C STUDY OBJECTIVES 18

C1 PRIMARY AIM 18

C2 SECONDARY AIMS 18

C3 TERTIARY AIMS 18

C4 RATIONALE FOR THE SELECTION OF OUTCOME MEASURES 19

D STUDY DESIGN 20

D1 OVERVIEW OR DESIGN SUMMARY 20

D2 SUBJECT SELECTION AND WITHDRAWAL 21

2.a Inclusion Criteria 21

2.a Exclusion Criteria 22

2.b Ethical Considerations 22

2.c Randomization Method and Blinding *22*

2.d Subject Recruitment Plans and Consent Process *23*

2.e Risks and Benefits *23*

2.f Early Withdrawal of Subjects from Transfusion Strategy and Trial Procedures *24*

2.g When and How to Withdraw Subjects *24*

2.h Data Collection and Follow-up for Withdrawn Subjects *24*

D3 TRIAL TRANSFUSION STRATEGIES 25

3.a Description *25*

3.b Transfusion Strategies *25*

3.c Rationale for Transfusion Thresholds *25*

3.d Method for Assigning Subjects to Treatment Groups *26*

3.e Preparation and Administration of Red Blood Cell Transfusions *26*

3.f Subject Compliance Monitoring *27*

3.g Prior and Concomitant Therapy *28*

3.h Blinding of Study Intervention *28*

E STUDY PROCEDURES 28

E1 SCREENING FOR ELIGIBILITY 28

E2 SCHEDULE OF MEASUREMENTS 28

E3 IN-HOSPITAL FOLLOW-UP 29

E4 FOLLOW-UP AT 30 DAYS 29

E5 FOLLOW-UP AT 6 MONTHS 30

E6 SAFETY AND ADVERSE EVENTS 30

6.a Safety and Compliance Monitoring *30*

6.b Medical Monitoring *30*

i Investigator Only 30

ii Independent Expert to Monitor 31

iii Independent Data and Safety Monitoring Board 31

6.c Definitions of Adverse Events *32*

6.d Classification of Events *33*

i Severity 33

ii Relationship 33

iii Expectedness 34

6.e Data Collection Procedures for Adverse Events and Unanticipated Problems *34*

6.f Reporting Procedures *35*

6.g Adverse Event Reporting Period *36*

6.h Post-study Adverse Event *36*

E7 STUDY OUTCOME MEASUREMENTS AND ASCERTAINMENT 36

7.a Definitions of Outcomes *36*

i Myocardial Reinfarction 36

ii Death and Cause of Death 37

iii Unscheduled Coronary Revascularization (unstaged) 37

iv Readmission to Hospital, overall and for primary cardiac diagnosis 37

v Unstable Angina 37

vi Congestive Heart Failure 37

vii TIA or Stroke 38

viii Deep Venous Thrombosis or Pulmonary Embolism 38

ix Bleeding 38

x Infections 38

xi Length of Stay and Intensive Care Unit Days 39

xii Quality of Life 39

7.b Ascertainment *39*

E8 BASELINE AND INTERVENTION CLINICAL CHARACTERISTICS 40

F STATISTICAL PLAN 40

F1 SAMPLE SIZE DETERMINATION AND POWER 40

F2 INTERIM MONITORING AND EARLY STOPPING 41

F3 ANALYSIS PLAN 45

F4 STATISTICAL METHODS 45

4.a Baseline Characteristics of the Enrolled Patient Sample *45*

4.b Adherence to Assigned Intervention *46*

4.c Primary Outcome Analysis *46*

4.d Analysis of Secondary and Tertiary Outcomes *46*

4.e Missing Outcome Data *47*

4.f Subgroup Analyses *48*

4.g Combining Vanguard and Main Trial Data *48*

F5 UNBLINDING PROCEDURES 49

G DATA HANDLING AND RECORD KEEPING 49

G1 CONFIDENTIALITY AND SECURITY 49

G2 TRAINING 50

G3 CASE REPORT FORMS AND SOURCE DOCUMENTS 50

G4 RECORDS RETENTION 51

G5 PERFORMANCE MONITORING 51

H STUDY MONITORING, AUDITING, AND INSPECTING 51

H1 STUDY MONITORING PLAN 51

H2 AUDITING AND INSPECTING 51

I STUDY ADMINISTRATION 52

I1 ORGANIZATION AND PARTICIPATING CENTERS 52

1.a Clinical Coordinating Center 52

1.b Data Coordinating Center 52

1.c Clinical Consortium 53

I2 FUNDING SOURCE AND CONFLICTS OF INTEREST 53

I3 COMMITTEES 53

3.a Steering Committee 53

3.b Operations Committee 54

3.c Executive Committee 54

3.d Clinical Events Committee 54

I4 SUBJECT STIPENDS OR PAYMENTS 54

I5 STUDY TIMETABLE 55

J PUBLICATION PLAN 56

K REFERENCES 57

L APPENDICES........ 64

L1 APPENDIX 1: MINT DATA ELEMENTS BY STUDY TIME POINT 65

L2 APPENDIX 2: SAE AND UP EVENT REPORTING TIMELINES........ 66

L3 APPENDIX 3: ACCRUAL TIMELINE MILESTONES 67

A Introduction

A1 Statement of Compliance

The trial will be conducted in accordance with the ICH E6, the Code of Federal Regulations on the Protection of Human Subjects (45 CFR Part 46), and the NIH Terms of Award. The Principal Investigator will assure that no deviation from, or changes to the protocol will take place without prior agreement from the sponsor and documented approval from the Institutional Review Board (IRB), except where necessary to eliminate an immediate hazard(s) to the trial participants. All personnel involved in the conduct of this study have completed Human Subjects Protection Training.

I agree to ensure that all staff members involved in the conduct of this study are informed about their obligations in meeting the above commitments.

Principal Investigator: __Jeffrey L Carson, MD______________________________
Print/Type Name

Signed: ____________________________________ Date: ______________
Signature

A2 Study Abstract

Accumulating evidence from clinical trials suggests that a restrictive transfusion strategy is safe in most clinical settings. However, a low oxygen carrying capacity from moderate anemia may be deleterious in patients with cardiac ischemia. The potential for harm associated with anemia in patients with acute symptomatic coronary disease is supported by pathophysiological data that maintaining higher hemoglobin levels could benefit the ischemic heart by increasing oxygen delivery. Systematic reviews of clinical trials evaluating transfusion strategies in patients with known ischemic heart disease document the absence of high quality data, which has resulted in an ongoing controversy. The lack of high quality evidence to guide transfusions in patients with acute myocardial infarction has been cited in several major guidelines as well as by an NIH expert panel.

This multicenter trial, the Myocardial Ischemia and Transfusion (MINT) trial, randomly allocates 3500 patients with acute myocardial infarction and a hemoglobin concentration less than 10 g/dL to be treated either according to a liberal or restrictive blood transfusion strategy. Patients assigned to the liberal transfusion strategy receive one unit of packed red blood cells following randomization and enough blood to raise the hemoglobin concentration to 10 g/dL or above any time a concentration less than 10 g/dL is detected. Patients assigned to the restrictive transfusion strategy are permitted to receive a transfusion if the hemoglobin concentration falls below 8 g/dL or if angina symptoms clearly related to the anemia occur and are not controlled with anti-anginal medications. Only enough blood is given to reach a hemoglobin concentration of 8 g/dL or relieve the symptoms. Transfusion is strongly recommended if the hemoglobin concentration falls below 7 g/dL.

The transfusion protocol is followed during the index hospitalization (up to 30 days). Each patient is contacted at 30 days for a comprehensive follow-up for assessment of several relevant clinical outcomes. Patients are contacted again at 180 days to ascertain vital status for assessment of six-month mortality.

A3 Primary Hypothesis

The primary hypothesis is that among patients with an acute myocardial infarction and a hemoglobin concentration less than 10 g/dL, a liberal transfusion strategy with a threshold of 10 g/dL reduces the rate of the composite outcome of all-cause mortality or recurrent nonfatal acute myocardial infarction through 30 days following randomization compared to a restrictive transfusion strategy with a threshold of 7 to 8 g/dL.

A4 Purpose of the Study Protocol

The purpose of the trial is to assess red blood cell transfusion strategies that are currently used in clinical practice and important medical events. Red blood cells are a limited and expensive medical therapy. Physicians frequently transfuse patients to maintain specific (and often differing) hemoglobin levels, despite the lack of evidence supporting the strategy. The study results, which will determine the benefit (or risk) of a liberal transfusion strategy, will influence the allocation of red blood cells worldwide.

A5 Schematic of Study Design

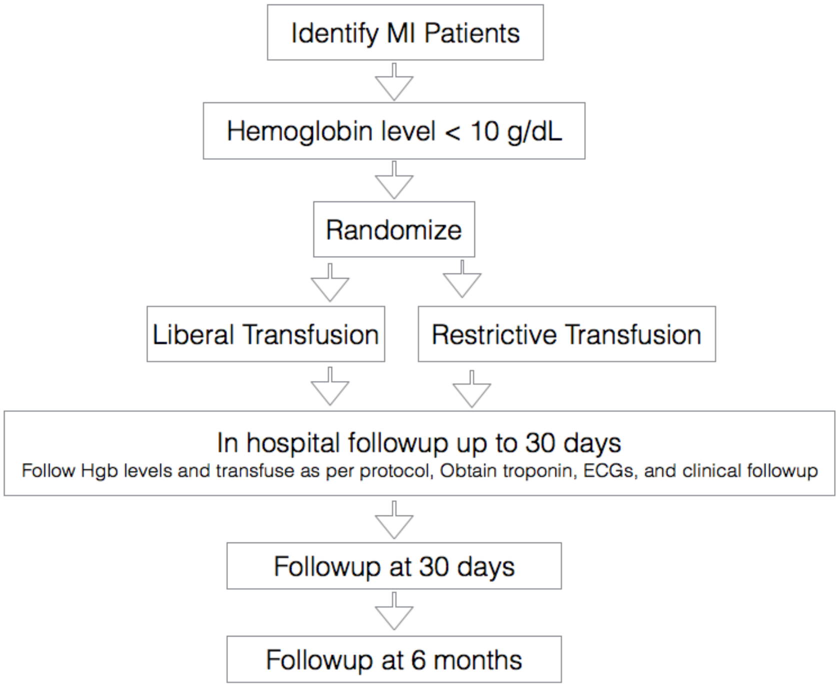

A6 Key Roles

Principal Investigator, Clinical Coordinating Center and Study Chair

Jeffrey L Carson, MD
Provost, Rutgers Biomedical Health Sciences
Rutgers Robert Wood Johnson Medical School
125 Paterson Street
New Brunswick, New Jersey 08901
Telephone: 732-235-7122
FAX: 732-235-7144
Email:jeffrey.carson@rutgers.edu

Principal Investigator, Data Coordinating Center

Maria Mori Brooks, PhD
Professor of Epidemiology
University of Pittsburgh
Graduate School of Public Health
A530 Crabtree Hall / 130 DeSoto Street
Pittsburgh, PA 15261
Telephone: (412) 624-1618
FAX: (412) 383-5891
Email: mbrooks@pitt.edu

Project Scientist

Simone Glynn MD, MSc, MPH
Branch Chief
Blood Epidemiology and Clinical Therapeutics Branch
Division of Blood Diseases and Resources
National Heart, Lung, and Blood Institute
Tel: 301-435-0065
Fax: 301-480-0867
Email: glynnsa@nhlbi.nih.gov

Project Officer

Pablo Cure, MD, MPH
Program Officer
Blood Epidemiology and Clinical Therapeutics Branch
Division of Blood Diseases and Resources
National Heart, Lung, and Blood Institute, NIH
6701 Rockledge Drive, Room 9163
Bethesda, MD 20892-7950
Tel: +1-301-451-7431Email: pablo.cure@nih.gov

B Background

B1 Prior Literature and Studies

Blood transfusion is a common medical intervention. In the United States, more than 16 million red blood cell units are transfused annually to 3.4 million patients.(1) Worldwide 108 million units of blood are collected per year.(2) Of all transfusions, approximately 25% of all red cells transfused are given to patients with a primary diagnosis of cardiac disease(3) and 8% of all cardiology admissions are transfused with RBCs.(4) The economic ramifications of this frequent intervention are significant. The latest estimates of the cost of a red blood cell unit range from \$522 to \$1183 (mean, \$761±\$294).(5) The safety of transfusion with respect to transmission of infectious agents has increased greatly throughout the past two decades, and complications are rare.(6, 7)

Anemia and Cardiac Disease: Generating evidence to guide transfusion threshold decisions in patients with myocardial infarction is especially important because coronary artery disease is so common,(8) anemia is present frequently in this setting, and is associated with increased mortality. A study of 44,242 patients with non-ST segment elevation myocardial infarction from 400 US hospitals found 22.2% of patients had a hematocrit <30% (Hgb ≤10 g/dL).(9) In-hospital mortality was 10.4% in patients with Hgb <10 g/dL compared to 2.7% in patients with Hgb >10 g/dL.(10) In a second study involving 17,676 patients with acute myocardial infarction, hospital-acquired anemia (Hgb <11 g/dL) developed in 20.1% of patients during the hospitalization. Patients with Hgb <9 g/dL had a strong association with mortality (odds ratio=3.39).(10) The mechanism of early mortality in patients with acute cardiac injury and anemia may be related to a low ischemic threshold leading to myocardial injury, congestive heart failure and ventricular arrhythmia.

Accumulating evidence from clinical trials suggests that a restrictive transfusion strategy is safe in most clinical settings(11-14) with the possible exception of patients with acute coronary syndrome. Most reviews and guidelines define a restrictive transfusion strategy as the administration of red cells once hemoglobin falls below either 7 or 8 g/dL while a liberal strategy is most often suggested as a transfusion trigger of 10 g/dL.

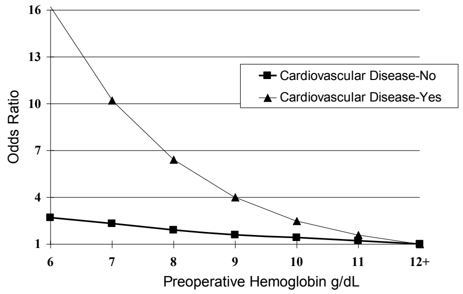

Pathophysiology: The AABB (formerly called the American Association of Blood Banks) guidelines on Red Blood Cell

Transfusion recently recommended the use of restrictive transfusion triggers in most patients with the exception of those with an acute coronary syndrome.(15, 16) Their rationale – in the absence of randomized trial evidence – was the large body of basic physiology and observational data would suggest that a restrictive strategy may be deleterious. This is because oxygen delivery to the myocardium is flow dependent since the heart extracts a high percentage of oxygen. Therefore, myocardial ischemia may be precipitated or worsened by low hemoglobin concentrations, especially in patients with coronary stenosis or active plaques. Studies performed in canines suggest a decreased ability to tolerate anemia in the presence of coronary artery disease.(17-19) Electrocardiographic changes consistent with ischemia are seen at hemoglobin concentration below 5 g/dL in normal animals but at 7-10 g/dL with experimentally induced coronary artery disease.

Data from patients who decline blood transfusion for religious reasons were congruent with animal data. In a retrospective cohort study that included 1,958 adult surgical patients who declined transfusion for religious reasons and found a significant interaction between underlying cardiovascular disease and preoperative Hgb level with respect to death (p=0.03).(20) In patients with underlying cardiovascular disease the adjusted odds of postoperative death began to rise sharply at Hgb level ≤10 g/dL while in patients without underlying cardiovascular disease there was a more subtle rate of increasing risk below 10 g/dL (see Figure 1). This study does not address whether transfusion would improve outcome.

Transfusion could be harmful by several possible pathophysiological mechanisms. Transfusion has been shown to increase platelet reactivity and increase procoagulant proteins.(21) Stored red blood cells take time to replenish 2, 3 DPG levels impairing release of oxygen(22, 23) and have low levels of nitrous oxide impairing oxgen delivery and vasodilation.(24) The membranes of stored red blood cells may become deformed and plug microvascular vessels.(25, 26)

Systematic Reviews, Observational Studies: We have identified or conducted systematic reviews of both observational studies and clinical trials. As expected, a systematic review of observational studies in all patients found that blood transfusion was associated with increased mortality and morbidity.(27) Two recent systematic reviews focusing specifically on patients with myocardial infarction identified 10 studies (9 observational and 1 pilot trial) reporting the effects of transfusions on mortality.(28, 29) Nearly all observational studies demonstrated an association between transfusion and higher mortality. The one exception was in a large study using Medicare billing data in 79,000 patients with acute myocardial infarction.(30) Transfusion was associated with a lower risk of death

when patients had an admission hematocrit below 0.33 (equal to a hemoglobin concentration of 11 g/dL) (odds ratio= 0.69; 95% CI, 0.53-0.89) and relationship between transfusion and better outcomes increased as the hematocrit fell. Indeed, based on subgroup analyses, the authors of the systematic review report that the adverse effects of transfusion appear to be mitigated in patients with a hematocrit less than 0.33. Unfortunately, observational studies cannot be used to evaluate the effect of red blood cell transfusion since the use of blood transfusion is a marker for illness burden.(29, 31) Thus, no matter how refined the adjustment is for differences in illness burden, it is difficult, if not impossible, to completely adjust for differences between patients receiving and not receiving blood transfusion.

Systematic Reviews, Clinical Trials: We have performed a systematic review of clinical trials evaluating transfusion triggers in a variety of populations that were published in the Cochrane database(32) and JAMA.(33) We have updated the review (34) and found that compared with higher hemoglobin transfusion thresholds (~10 g/dL), a hemoglobin transfusion threshold of 7 or 8 g/dL is associated with fewer red blood cell units transfused (mean difference, -1.22 units per patient), without adverse associations with mortality, cardiac morbidity, functional recovery, or length of hospital stay. The relative risk for the association of restrictive versus liberal transfusion on 30-day all-cause mortality was 0.99 (95% CI, 0.82 to 1.20).

A recent meta-analysis of 11 selected trials enrolling patients with cardiovascular disease (including data obtained from authors from four trials) was recently reported. The risk ratio for the association between transfusion thresholds and 30-day mortality was 1.15 (95% confidence interval 0.88 to 1.50), but the risk of acute coronary syndrome in patients in the restrictive compared with liberal transfusion group was increased in nine trials (risk ratio 1.78, 95% confidence interval 1.18 to 2.70).(35)

Clinical Trials in Acute Coronary Syndrome: Our systematic review identified two pilot clinical trials that included patients suffering an acute coronary syndrome. The first was a small pilot trial including 45 patients with acute myocardial infarction.(36) Patients with hematocrit less than 30% were randomly allocated to a liberal (hematocrit <30%) versus restrictive (hematocrit < 24%) transfusion threshold. The primary clinical safety measurement of in-hospital death, recurrent myocardial infarction, or new or worsening congestive heart failure occurred in 8 patients in the liberal arm and 3 in the restrictive arm (p< 0.046). There were 2 deaths in restrictive group and 1 death in the liberal group. The authors concluded a definitive trial was urgently needed.

In contrast, the MINT pilot enrolled 110 patients and found the pre-defined primary outcome of death, myocardial infarction, or unscheduled revascularization within 30 days occurred in 6 patients (10.9%) in the liberal-transfusion strategy and 14 (25.5%) in the restrictive-transfusion strategy p=0.054). Death at 30 days was less frequent with liberal transfusion 1 (1.8%) compared to restrictive transfusion 7 (13.0%); p=0.032.

Overall, there were 2 deaths in the liberal transfusion strategy and 9 deaths in the restrictive transfusion strategy (relative risk= 3.74, 95% CI 0.80-17.49; p=0.09) when the two trials in acute coronary syndrome are combined.

Other Trials (37) with Signal of Harm from Restrictive Transfusion: The two most recently published trials also found a higher mortality in patients in the restrictive transfusion group in patients with ischemic heart disease. The Titre2 trial contrasted liberal transfusion (9 g/dL) and restrictive transfusion (7.5 g/dL) in postoperative patients undergoing cardiac surgery. The short-term outcomes were comparable between the transfusion strategies, but at 90 days follow-up, overall mortality was higher in the restrictive transfusion strategy than the liberal transfusion strategy (hazard ratio=1.64; 95% confidence interval, 1.00 to 2.67, p=0.045). In a cluster randomized trial in 939 patients with GI bleeding, the mortality was trending higher in subgroup of patients with underlying ischemic heart disease; liberal transfusion strategy was 3% and in the restrictive transfusion strategy was 12% (difference =10.7%; 95% confidence intervals -9.8 to 31.2; interaction p=0.11)

Variation in Transfusion and Guidelines: The systematic reviews of observational studies and randomized trials evaluating the impact of anemia and transfusion highlight the lack of any study with sufficient numbers of patients to guide clinical care. All of this uncertainty has helped fuel significant practice variation. Two large studies in 44,242 patients with non-ST segment elevation myocardial infarction from 400 US hospitals(9) and 17,676 patients with acute myocardial infarction demonstrate substantial variation in transfusion.(10) Similar variation as observed in over 2000 hospitalized patients from the California Kaiser Permanente Health System. A significant proportion of patients had transfusion thresholds at every cut-off from 7 to 10 g/dL – again indicating important clinical uncertainty.

The variation in transfusion practice may further be exacerbated by the great variability in the transfusion guideline recommendations. While all conclude that there are too few high quality studies, recommendations vary widely among organizations. The American Red Cross, AABB, British

Committee for Standards in Haematology were not able to recommend a course of action,(38, 39) the American College of Physicians recommends 7-8 g/dL in patients with heart disease but is silent in acute coronary syndrome patients;(40) the American College of Cardiology/American Heart Association suggests avoidance of transfusion unless hemoglobin less than 8 g/dL(41, 42); and the European Society of Cardiology recommends transfusion only in case of compromised hemodynamic status and hemoglobin less than 7 g/dL.(43) The recently published guidelines from the AABB concluded that there was insufficient evidence in patients with acute MI and did not provide a specific recommendation, (44) while the UK National Clinical Guidelines Centre recommended transfusion at 8 g/dL (45). Given the lack of high quality evidence to guide transfusion in patients with acute myocardial infarction, it is not surprising that there is variation in recommendations emanating from different organizations.

B2 Rationale for this Study: Equipoise

Every day, clinicians encounter anemic patients who have acute ischemic heart disease where a decision to transfuse must be made. However, clinicians do not know what to do because: 1) Observational studies and randomized trials have come to different conclusions and are flawed; 2) Pathophysiological arguments can be made for liberal and restrictive strategies; 3) Some clinical trials in other settings suggest a restrictive transfusion approach is safe but other trials signal the possibility of reduced mortality with liberal transfusion in patients with cardiac disease; 4) Guidelines provide conflicting advice. This has led to practice variation(9, 46) and confusion in the clinical community with no clear guidance on when to transfuse. Thus, the NHLBI State of the Science expert panel concluded in March 2015 “equipoise for transfusion thresholds persists in patients with ischemic heart disease.” (47) The ACC/AHA guidelines for management of STEMI concluded, “the optimal hemoglobin level in the transfused patient is not known.”(41, 42) Furthermore, the recently published guidelines by the AABB identified acute coronary syndrome as clinical setting where the evidence is “judged to be insufficient” to recommend transfusion threshold.(16) Based on these findings and existing data there clearly is equipoise. For all these reasons, a high quality randomized trial to guide transfusion is urgently needed to answer this clinically relevant question.

C Study Objectives

C1 Primary Aim

The primary aim is to determine whether a liberal transfusion strategy with a threshold of 10 g/dL reduces the composite outcome of all-cause mortality or nonfatal myocardial reinfarction through 30 days following randomization, compared to a restrictive transfusion strategy with a threshold of 7 to 8 g/dL among patients with an acute myocardial infarction and a hemoglobin concentration less than 10 g/dL.

C2 Secondary Aims

1) To determine whether a liberal (10g/dL) transfusion strategy reduces all-cause mortality within 30 days, compared to a restrictive transfusion strategy.

2) To determine whether a liberal (10g/dL) transfusion strategy reduces myocardial reinfarction within 30 days, compared to a restrictive transfusion strategy.

3) To determine whether a liberal (10g/dL) transfusion strategy reduces the composite outcome of all-cause mortality, nonfatal myocardial reinfarction, ischemia driven unscheduled coronary revascularization (percutaneous coronary intervention or coronary artery bypass grafting), or readmission to the hospital for ischemic cardiac diagnosis within 30 days, compared to a restrictive transfusion strategy.

C3 Tertiary Aims

1) To determine whether a liberal (10g/dL) transfusion strategy reduces all-cause mortality, nonfatal myocardial reinfarction, or unstable angina (i.e. acute coronary syndrome) within 30 days, compared to a restrictive transfusion strategy.

2) To determine whether a liberal (10g/dL) transfusion strategy reduces ischemia driven unscheduled coronary revascularization within 30-days compared to a restrictive strategy.

3) To determine whether a liberal (10g/dL) transfusion strategy reduces unscheduled readmission to hospital for ischemic cardiac diagnosis within 30 days, compared to a restrictive strategy.

4) To determine whether a liberal (10g/dL) transfusion strategy increases congestive heart failure within 30 days, compared to a restrictive transfusion strategy.

5) To determine whether a liberal (10g/dL) transfusion strategy reduces unscheduled readmission to hospital for any reason within 30 days, compared to a restrictive strategy.

6) To determine whether a liberal (10g/dL) transfusion strategy increases each of the individual thrombotic/hemorrhagic outcomes of stroke, pulmonary embolism or deep venous thrombosis, and bleeding within 30 days, compared to a restrictive strategy.

7) To determine whether a liberal (10g/dL) transfusion strategy increases each of the individual infectious outcomes of pneumonia, blood stream, and urinary tract within 30 days, compared to a restrictive strategy.

8) To determine whether a liberal (10g/dL) transfusion strategy reduces each of the individual in-hospital outcomes of length of hospital stay post randomization and number of days in intensive care unit, compared to a restrictive strategy.

9) To determine whether a liberal (10g/dL) transfusion strategy increases patient reported quality of life using the EuroQol questionnaire (EQ-5D) at 30 days compared to a restrictive strategy

10 To determine whether a liberal (10g/dL) transfusion strategy reduces all-cause mortality at 6-months following randomization, compared to a restrictive strategy.

C4 Rationale for the Selection of Outcome Measures

The study outcomes assess the clinically important benefits and harms of transfusion and anemia in vulnerable individuals with compromised myocardium. Blood transfusions may decrease ischemic injury and improve myocardial performance by improving oxygen delivery to the myocardium in high-risk patients. However, transfusions may also harm patients. Blood transfusions acutely increase blood volume in patients who may not adapt rapidly enough resulting in increased rates of pulmonary edema and heart failure. The increased blood volume could also lead to higher risk of bleeding from increased intravascular pressure. Blood transfusion is also associated with immunosuppression and may lead to infection. In addition, laboratory data suggest that transfusions may not enhance oxygen delivery and may be associated with increased platelet aggregation.

If transfusion to maintain the hemoglobin concentration >10 g/dL does mitigate the clinical consequences of anemia in ischemic cardiac injury, there will be a reduction of mortality and reinfarction. Higher hemoglobin concentrations might also reduce other sequelae of decreased oxygen delivery to the myocardium; unscheduled coronary revascularization, hospital readmission for ischemic symptoms, unstable angina, and cardiovascular mortality.

On the other hand, if transfusion results in clinically important fluid overload, immunosuppression, increased viscosity, and inflammation there will be an increase in congestive heart failure, infection, bleeding, stroke, and pulmonary embolism or deep venous thrombosis. Each of these may contribute to death or reinfarction.

Higher hemoglobin levels may also be associated with a more positive feeling of well-being and a better perceived quality of life as measured by the EQ-5D.

Outcomes are assessed at 30 days since blood transfusion will have its maximum effect within this time period. Mortality will also be assessed at 6 months to determine if early effects of blood transfusion persist.

D Study Design

D1 Overview or Design Summary

This is a randomized, unblinded, two group multicenter clinical trial. Eligible study patients are randomized to receive either the liberal or the restrictive transfusion strategy. Transfusion strategy assignment is not blinded. The transfusion protocol is followed during the Index hospitalization (for up to 30 days). Each patient will be contacted at 30 days to ascertain study outcomes and at 6 months when vital status will be verified. The 30-day and 6-month follow-ups will be administered by telephone, but the follow-up questions could be administered in person, for example, if the patient is in the hospital during the follow-up window. During the 30-day follow-up, readmissions to the hospital that have occurred within the 30 days are identified and medical records will be obtained. The Clinical Events Committee, masked to treatment allocation, will adjudicate occurrences of myocardial infarction within the 30-day window.

Each of the transfusion strategies in this trial is routinely used in current medical practice. Study patients will be followed while they are in the hospital (for up to 30 days) during which time

hemoglobin levels, cardiac biomarker of necrosis levels, electrocardiograms, and number of units of red blood cell transfusions administered will be collected.

The transfusion strategies will be compared for differences in mortality, cardiac events and other important morbidity, hospital re-admissions, and patient perceived quality of life.

The goal of this study is to determine whether a liberal transfusion strategy is superior to a restrictive strategy in anemic patients with acute myocardial infarction.

D2 Subject Selection and Withdrawal

2.a Inclusion Criteria

The eligible study population includes patients who meet all of the following criteria: 1) 18 years of age or older; 2) with either ST segment elevation myocardial infarction or Non ST segment elevation myocardial infarction consistent with the 3rd Universal Definition of Myocardial Infarction criteria (37) that occurs on admission or during the index hospitalization, and 3) with a hemoglobin concentration less than 10 g/dL at the time of random allocation.

To simplify the diagnosis of acute myocardial infarction, we will require a rise in cardiac biomarker values [preferably cardiac troponin (cTn)] with at least one value above the upper reference limit of normal of the hospital. We anticipate (and will confirm) that the hospital upper limit of normal for troponin will be equivalent to or above the 99th percentile upper reference at all hospitals.

In addition to evidence of myocardial necrosis, we require patients to have at least one of the following: (1) symptoms of ischemia; (2) new/presumed new ST segment or T wave (ST-T) changes or new left bundle branch block (LBBB); (3) development of pathological Q waves; (4) imaging evidence of new loss of viable myocardium or new regional wall motion abnormality; and/or (5) identification of an intracoronary thrombus by angiography. (37) We will include patients with Type 1 (i.e., spontaneous MI presumably related to atherosclerotic plaque rupture, ulceration, fissuring, erosion, or dissection with resulting intraluminal thrombus in one or more of the coronary arteries, leading to decreased myocardial blood flow or distal platelet emboli with ensuing myocyte necrosis), Type 2 (i.e., secondary to an ischemic imbalance such that myocardial injury with necrosis occurs due to myocardial oxygen supply and/or demand mismatch),Type 4b (i.e., stent thrombosis at angiography), and Type 4c (i.e., severe in-stent restenosis without evidence of thrombus).

2.a Exclusion Criteria

Patients will be excluded if any of the following criteria are met 1) uncontrolled acute bleeding at the time of randomization defined as the need for uncrossed or non-type specific blood; 2) decline blood transfusion; 3) scheduled for cardiac surgery during the current admission; 4) are receiving only palliative treatment; 5) if known that follow-up will not be possible at 30 days; 6) if previously participated in MINT or 7) if currently enrolled in a competing study that interferes with the intervention or follow-up of MINT or enrolled in a competing study that has not been approved by the local IRB.

Patients who have had an episode of uncontrolled bleeding may be enrolled later if the hemoglobin remains below 10 g/dL, they are no longer actively bleeding, and they are otherwise still eligible.

2.b Ethical Considerations

Both transfusion strategies assessed in this trial are widely used in clinical practice. There is uncertainty about which strategy is better, and therefore there is clinical equipoise to conduct this study. Thus, there are no clinical risks to patients above those of usual practice. There are no other alternative treatments.

At the time consent is obtained, the clinical site study staff will also request the names and contact information of two additional individuals who may be contacted in the event study staff are not able to reach the patient directly at the follow-up time points. This will minimize loss to follow-up. This information will be retained at the clinical site and destroyed at the end of the trial.

2.c Randomization Method and Blinding

The Data Coordinating Center (DCC) will prepare the randomization schedules. Allocation of the transfusion intervention strategies will be in a 1:1 ratio. Given the diverse patient mix among participating clinical sites, the randomization will be stratified by clinical site, and a permuted block design with random block sizes will be used to balance treatment assignments within each clinical site.

Study patients and physicians caring for the patient cannot be masked to treatment assignment (i.e., administration of red blood cell transfusion). However, the central classification of the myocardial reinfarction component of the primary outcome is performed masked to assignment.

2.d Subject Recruitment Plans and Consent Process

The study recruits hospitalized patients diagnosed with acute myocardial infarction (Type 1, Type 2, Type 4b, or Type 4c). Prior to study initiation, each cardiologist at the clinical site will be personally contacted by the clinical site principal investigator or coordinator and permission will be sought to recruit patients who are eligible for the study. Patients of physicians who do not wish to participate in the trial will not be approached for recruitment.

Study staff will identify potential study patients, confirm that the physician agrees that the patient can be randomized into the study and approach the patient for consent. Surrogate consent, in accordance with local IRB rules, will be sought for each eligible patient who is not able to grant consent. A substantial number of patients eligible for this trial are likely to be critically ill, medicated, and/or cognitively impaired and unable to grant consent. It is essential that these patients be included as physicians routinely face the dilemma of whether or not they should be transfused.

2.e Risks and Benefits

In patients with heart disease, risks and benefits are considerably different than most other patient populations. Oxygen delivery to the myocardium is flow dependent since the heart extracts a high percentage of oxygen and myocardial ischemia may be precipitated or worsened by low hemoglobin concentrations, especially in patients with acute myocardial infarction. Anemia, if untreated may result in increased risks of further myocardial ischemia and injury. Transfusions, on the other hand, may result in increased risks of pulmonary edema and heart failure (Transfusion-Associated Circulatory Overload), from the significant amounts of volume given to patients with impaired ability of the heart to pump (systolic dysfunction) or ability to relax and fill (diastolic dysfunction). Other adverse effects of allogeneic blood transfusion were also a concern: immunologic (transfusion-related acute lung injury [TRALI],(48) and transfusion-related immune modulation [TRIM] possibly leading to increased bacterial infections).(49) Thus, there is clinical equipoise because it is unclear whether the benefits of immediate correction of anemia with transfusion are outweighed by the potential side effects of transfusion.

2.f Early Withdrawal of Subjects from Transfusion Strategy and Trial Procedures

The study participant (i.e. patient) can be withdrawn from the transfusion strategy (liberal or restrictive transfusion allocation) at any time, either at the request of the treating physician or the patient themselves. The clinical site director may also withdraw a patient. Following the withdrawal, all transfusion decisions will be per treating physician. The patient or physician may also request withdrawal from any trial procedure or follow-up.

2.g When and How to Withdraw Subjects

There are few reasons why a patient or physician might want to withdraw from the trial. If the patient has an adverse effect from a prior transfusion or proves to be difficult to cross match, the physician or patient may choose to withdraw from the study. However, the most common reasons are likely to be patient’s preference not to participate in research or desire not to have extra blood tests or be contacted for follow-up. If a concern is raised and a request for withdrawal is made by the patient or the physician, study staff will confirm the issue (e.g., transfusion strategy, study required measurements and/or telephone follow-up) and try to address the concern. Study staff will be required to contact the MINT CCC PI to discuss the individual situation. If appropriate, the patient will be given the opportunity to refrain from the objectionable study procedure(s) and remain in the overall study. All efforts will be made to avoid a participant feeling unduly pressured to remain in the study. If no intermediate solution is acceptable to the patient and/or treating physician, the patient will be withdrawn from the study. The study staff will document the date of withdrawal, any known reasons for withdrawal and whether the patient will continue with any of the study procedures and/or follow-up. All treating physicians will be immediately notified of a withdrawal from the transfusion strategy (liberal or restrictive transfusion allocation).

2.h Data Collection and Follow-up for Withdrawn Subjects

At the time of withdrawal, the study staff will request permission to continue with data collection and follow-up through the 6-month study time window. However, the exact amount of follow-up performed will vary in accordance with the patients’ authorization.

D3 Trial Transfusion Strategies

3.a Description

We are comparing two commonly used approaches to transfusion therapy, both of which can be considered as "standards of care." For both strategies, blood must be administered one unit at a time followed by a hemoglobin measurement. The transfusion strategy will be followed throughout the index hospitalization up to 30 days, discharge, or death.

3.b Transfusion Strategies

Restrictive Transfusion Strategy: Patients randomized to the restrictive transfusion strategy will be permitted to receive a transfusion if the hemoglobin concentration falls below 8 g/dL and will be strongly recommended to receive transfusion if the hemoglobin concentration is below 7 g/dL. Transfusion is also permitted if angina symptoms (i.e., retrosternal chest discomfort, chest discomfort described as pressure or heaviness) that are thought by the clinician to be related to anemia occur and are not controlled with anti-anginal medications (sublingual nitroglycerin or equivalent therapy). Blood will be administered one unit at a time and enough blood given to increase the hemoglobin concentration above 7 to 8 g/dL or to relieve symptoms of uncontrolled angina.

Liberal Transfusion Strategy: Patients randomly allocated to the liberal transfusion strategy will receive one unit of packed red cells following randomization and will receive enough blood to raise the hemoglobin concentration to 10 g/dL or above any time the hemoglobin concentration is detected to be below 10 g/dL. A post transfusion hemoglobin measurement showing a hemoglobin level of at least 10 g/dL must be obtained.

A patient in either group may be transfused at any time without a hemoglobin level if the patient is actively bleeding (e.g., brisk gastrointestinal bleeding) and the physician believes an emergency transfusion is needed. A patient in either group with history of congestive heart failure or low ejection fraction may receive diuretics prior to or after transfusion.

3.c Rationale for Transfusion Thresholds

The restrictive transfusion group reflects many current guidelines (including European guidelines)(43, 50). A blood transfusion will be permitted in the restrictive group if the hemoglobin concentration is less than 8 g/dL. Discussions with many cardiologists suggested that many clinicians are not

comfortable with a threshold as low as 7 g/dL (as used in TRICC); however, individual clinicians may choose to use a threshold of less than 7 g/dL to trigger transfusions. Patients may be transfused for signs or symptoms when the clinician believes it is necessary although this did not occur in the pilot trial and was uncommon in the FOCUS trial which also incorporated symptoms in the restrictive transfusion protocol.

In the liberal transfusion group, a threshold of less than 10 g/dL was chosen because oxygen delivery to the myocardium is flow dependent and myocardial ischemia may be precipitated or worsened by low hemoglobin concentrations in patients with coronary stenosis or active plaques. Studies performed in canines found electrocardiographic changes consistent with ischemia as high as 10 g/dL with experimentally induced coronary artery disease. Data from patients who decline blood transfusion for religious reasons were congruent with animal data and found the odds of death rose as the hemoglobin fell below 10 g/dL.

3.d Method for Assigning Subjects to Treatment Groups

Clinical site staff will obtain the randomly assigned transfusion strategy for each eligible consented patient using the MINT website. The randomization system will be available via a secure area of the MINT project website with access restricted to those clinical site personnel with permission to randomize patients. Those who are certified to use the randomization system will be trained to adhere to a strict randomization protocol. In particular, the clinical site personnel must confirm the patient’s eligibility status in the system before a transfusion strategy assignment is provided. If the web-based randomization system is not accessible, clinical site personnel will be instructed to follow back-up procedures in order to ensure that the clinical centers are able to randomize patient 24/7.

3.e Preparation and Administration of Red Blood Cell Transfusions

All red blood cell units are maintained and ordered through the hospital blood bank. The storage solution and storage time will be at the discretion of the blood bank, but only leukoreduced red blood cell transfusion will be used. The transfusions are administered by hospital staff in accordance with hospital policy. Study staff will alert the nursing and medical staff to the assigned transfusion strategy each time a new patient has been enrolled in the study. Treating staff will order (or not order) red cell transfusions in accordance with the protocol.

3.f Subject Compliance Monitoring

There are two primary site based mechanisms to ensure adherence to the assigned transfusion strategy 1) study staff review of the medical record and, if necessary, direct discussion with the treating physicians and, 2) direct assistance from the blood bank.

The clinical site is to obtain a daily hemoglobin level for each randomized patient for the first three days following randomization (or through hospital discharge, if sooner). Additional hemoglobin levels are measured as clinically indicated. Study staff will closely monitor each patient to ensure that each of the required hemoglobin levels are drawn, review the results, and confirm that the transfusions have been ordered (or not ordered) in accordance with the transfusion assignment. If there is a hemoglobin value that should trigger a transfusion and none is ordered or, as the alternative, a transfusion ordered without a hemoglobin value to trigger the transfusion, study staff will discuss the case with the treating physician. Likewise, if a required hemoglobin level has not been ordered, the study staff will alert the physician. The required hemoglobin measurements are of primary importance to verify that patients in the liberal strategy maintain a level of at least 10 g/dL.

The clinical site will also request assistance from the blood bank to help prevent protocol violations in the restrictive strategy. Study staff will notify the blood bank each time a patient is assigned to the restrictive strategy and request notification prior to the release of any red blood cell transfusions that are ordered. The study staff will then review the medical record to verify that the transfusion is in accordance with the protocol. If administration of blood is a violation of the protocol, study staff will contact the ordering physician to discuss transfusion plans and to clarify the study protocol. However, study staff do not approve or disapprove the transfusion. The final decision on the transfusion is always the treating physician's.

Study wide, the DCC will centrally monitor transfusions and hemoglobin levels on an ongoing basis. Transfusion rates for each strategy will be measured overall and by clinical site. Specifically, the DCC will identify instances when 1) patients randomized to the liberal strategy do not receive a transfusion, 2) patients randomized to the liberal strategy are discharged with a hemoglobin level < 10 g/dL and, 3) patients randomized to the restrictive strategy without anginal symptoms and a hemoglobin level ≥ 8 g/dL receive a transfusion. Reports of the protocol violations will be prepared by the DCC. The Clinical Coordinating Center (CCC) will review these reports and discuss as necessary with the clinical sites.

3.g Prior and Concomitant Therapy

Transfusion prior to randomization is at the discretion of the clinicians. An otherwise eligible patient may be randomized as long as the hemoglobin level is <10g/dL, regardless of prior transfusions at the time of randomization. Once the patient has been randomized all red cell transfusions are administered per the study protocol. The protocol will not control the administration of other blood products including platelets or fresh frozen plasma. Similarly, it will not mandate specific medical or procedural treatments to manage these patients.

3.h Blinding of Study Intervention

Due to the nature of the interventions, study patients and physicians caring for the patients cannot be blinded to transfusion assignment. However, transfusion strategy assignment will be concealed during the central classification of the myocardial reinfarction component of the primary outcome.

E Study Procedures

E1 Screening for Eligibility

Several approaches to screening for study patients will be used. Study staff will review the results of all troponin levels daily. Medical records for patients with values above the upper reference limit of normal are reviewed and assessed for eligibility. Study staff will screen all admissions to the cardiac care unit, and scheduled for cardiac catheterization. The physician of each eligible patient with a hemoglobin level <10 g/dL will be contacted to confirm that the patient can be entered into the trial, and consent will be sought. Study staff will follow otherwise eligible patients with hemoglobin levels ≥ 10 g/dL and consent the patient if the hemoglobin level drops to the entry level.

E2 Schedule of Measurements

Each randomized patient is required to have hemoglobin levels, troponin levels, and electrocardiogram readings at specified time points (while in the hospital). The blood samples will normally be drawn with the daily morning blood phlebotomy.

The required time points for <u>hemoglobin levels</u> are: 1) within 24 hours prior to randomization (eligibility hemoglobin level), 2) day 1 post randomization, 3) day 2 post randomization, 4) day 3 post randomization.

The required time points for the troponin levels are: 1) within 24 hours prior to randomization, 2) 12 hours following randomization, 3) day 1 post randomization, 4) day 2 post randomization, 5) day 3 post randomization.

The required time points for the ECGs are: 1) within 24 hours prior to randomization, 2) day 1 post randomization, 3) day 2 post randomization, 4) day 3 post randomization.

A listing of the types of data elements that will be collected at each time point is presented in Appendix 1.

E3 In-hospital Follow-up

Study staff will review the medical records and follow participants during the hospitalization, for up to 30 days post randomization. They will carefully follow and document hemoglobin levels and transfusion status to assure that the transfusion protocol is being followed. In addition, during the first 3 days following randomization they will confirm that each of the required cardiac biomarkers for necrosis, hemoglobin levels, and electrocardiograms has been ordered and the results are recorded.

At 30 days post randomization, or hospital discharge if sooner, the study staff will complete and submit all study data related to the hospitalization including baseline health status, laboratory results, post randomization events and copies of all electrocardiograms performed.

E4 Follow-up at 30 Days

Study staff at the clinical sites will contact each patient at 30 days following randomization. In general, the 30-day follow-up will be administered by telephone, but the follow-up questions could be administered in person. The follow-up interview will include questions about the patient's health and quality of life during the follow-up interval and will determine if there have been any re-admissions to a hospital within 30 days of randomization. Local site staff will obtain the hospital records for each re-admission.

There will be a 30-day window (from 30 days to 60 days following randomization) for study staff to obtain the 30-day follow-up information. In the event they are not able to contact the patient directly, they will try to reach the alternate contacts that have been provided, and a letter will be sent to the patient if needed.

E5 Follow-up at 6 Months

Study staff at the clinical sites will perform a final follow-up contact at 6 months following randomization to determine the patient's vital status. There will be a 30-day window (from 180 days to 210 days following randomization) to obtain the 6-month information. In the event that study staff are not able to contact the patient at the 6-month time point, they will follow the same procedure outlined at the 30-day follow-up.

E6 Safety and Adverse Events

6.a Safety and Compliance Monitoring

The DCC will monitor key aspects of protocol compliance, and reports will be developed and disseminated to monitor the ongoing progress of the study. Protocol safety and compliance reports will include enrollment of ineligible patients, follow-up data collection outside of protocol-defined windows, deviations from the protocol, and adherence to established adverse event reporting and event adjudication procedures. Reports will be provided to the CCC, the Steering Committee, and the DSMB on a systematic basis.

6.b Medical Monitoring

The DCC is responsible for tracking the Serious Adverse Events (SAEs) reported by clinical sites in the MINT trial. The DCC will track the time for each step of the process to ensure that turnaround times adhere to acceptable reporting practices specified by the FDA and NIH. The current reporting guidelines from NHLBI (https://www.nhlbi.nih.gov/research/funding/human-subjects/adverse-event) will be followed, and the timelines are included in Appendix 2.

i Investigator Only

The clinical site will submit a SAE form to the DCC for unexpected serious adverse events occurring within 30 days of randomization and for unexpected deaths occurring within 6 months of randomization. The clinical sites are responsible for notifying their local Institutional Review Board. Expected SAEs will be reported on the standard MINT in-hospital and follow-up data collection forms.

ii Independent Expert to Monitor

The DCC will notify the MINT Medical Safety Officer of unexpected SAEs submitted by a clinical site and make the relevant information available to the officer. The Medical Safety Officer will determine a) the severity of the event, b) the unexpectedness, and c) the relatedness to the study protocol. In accordance with safety reporting regulations, all SAEs that are determined by the Medical Safety Officer to be both unexpected and possibly, probably or definitely related to the study protocol will be subject to expedited reporting to the Data and Safety Monitoring Board (DSMB), NIH, and any other oversight entities as appropriate. The NIH Project Officer and the DSMB chairperson will review these documents and may decide to convene the DSMB to discuss issues related to monitoring such events.

iii Independent Data and Safety Monitoring Board

An external Data and Safety Monitoring Board (DSMB) consisting of members appointed by the National Heart, Lung, and Blood Institute (NHLBI) will monitor the study, advise the NIH Program Office and provide input to the Steering Committee. The MINT DSMB will include experts in cardiology, transfusion medicine, biostatistics and ethics. The DSMB will review the study protocol and provide NHLBI with recommendations including whether the protocol and patient recruitment can be initiated. NHBLI will approve the study protocol based on the DSMB recommendations before patient recruitment is initiated. Throughout the course of the trial, the Data and Safety Monitoring Board (DSMB) will review recruitment, retention, data completeness, and protocol deviations on a semi-annual basis and will provide recommendations to NHLBI.

Adverse events will be monitored in four distinct ways: 1) the DSMB will review all reported adverse events and monitor the incidence rates of adverse events on a semi-annual basis, 2) the expedited review of unexpected Serious Adverse Events (SAE) that are related to the MINT protocol and unanticipated problems (UP), 3) all study outcomes will be evaluated by assigned treatment group on semi-annual basis, and 4) a formal statistical interim monitoring of the efficacy of the primary outcome by assigned treatment group will be performed on an annual basis. If unexpected safety concerns arise from the trial data or from external research or literature, safety data will be examined on an ad hoc basis. The DCC will work with the NIH and with the DSMB to make certain that the board members have sufficient information to comprehensively monitor patient safety throughout the MINT trial. The DSMB may advise early termination of the trial for safety reasons or make other recommendations regarding modifications to the protocol.

In order to ensure that the MINT trial has adequate power to test its primary aims, the DSMB will review an interim sample size/power analysis after half of the patients complete the 30-day follow-up. Revised sample size estimates will be calculated based on the observed overall event rate (i.e. the two transfusion groups combined) and the proposed power and effect size.

6.c Definitions of Adverse Events

An adverse event is defined as an untoward or unfavorable medical occurrence in a human participant, including any abnormal sign (for example, abnormal physical exam or laboratory finding), symptom, or disease, temporally associated with a person's participation in the research, whether or not considered related to a person's participation in the research. A **Serious Adverse Event** (SAE) is defined as an adverse event that meets **any** of the following criteria:

- results in death;
- is life-threatening i.e. places a participant at immediate risk of death from the event as it occurred;
- requires inpatient hospitalization or prolongation of existing hospitalization;
- results in a persistent or significant disability/incapacity;
- results in a congenital anomaly/birth defect; OR
- any other adverse event that, based upon appropriate medical judgment, may jeopardize the participant's health and may require medical or surgical intervention to prevent one of the other outcomes listed in this definition.

An **Unanticipated Problem (UP)** is defined as any incident, experience, or outcome that meets **all** of the following criteria:

- Unexpected in terms of nature, severity, or frequency, taking protocol research procedures and participation population characteristics into consideration.
- Related or possibly related to a person's participation in the research.
- Places participants or others at a greater risk of harm (including physical, psychological, economic, or social harm) than was previously known or recognized.

6.d Classification of Events

i Severity

The **severity** of the adverse event refers to the intensity of an event and is categorized as 1) Mild; asymptomatic or mild symptoms; clinical or diagnostic observations only; intervention not indicated, 2) Moderate; minimal, local or noninvasive intervention indicated; limiting age-appropriate instrumental activities of daily living, 3) Severe or medically significant but not immediately life-threatening; hospitalization or prolongation of hospitalization indicated; disabling; limiting self-care activities of daily living, 4) Life-threatening consequences; urgent intervention indicated, 5) Death related to AE.

ii Relationship

Relatedness refers to the extent to which an adverse event is considered to be related to the intervention or study procedures. An adverse event is considered **related** if there is a reasonable possibility that the event may have been caused by the procedure. Note that the term "**suspected**" is also means possibly, probably or definitely related to the intervention or study procedures.

The following definitions apply to relatedness:

1. Unrelated
- Adverse event is clearly due to extraneous causes (e.g., underlying disease, environment)

2. Unlikely (adverse event **must meet 2** of the following):
- Does not have temporal relationship to intervention
- Could readily have been produced by the participant's clinical state
- Could have been due to environmental or other interventions
- Does not follow known pattern of response to intervention
- Does not reappear or worsen with reintroduction of intervention

3. Possible (adverse event **must meet 2** of the following):
- Has a reasonable temporal relationship to intervention
- Could not readily have been produced by the participant's clinical state
- Could not readily have been due to environmental or other interventions
- Follows a known pattern of response to intervention

4. Probable (adverse event **must meet 3** of the following):

- Has a reasonable temporal relationship to intervention
- Could not readily have been produced by the participant's clinical state or have been due to environmental or other interventions
- Follows a known pattern of response to intervention
- Disappears or decreases with reduction in dose or cessation of intervention

5. Definite (adverse event **must meet 4** of the following):

- Has a reasonable temporal relationship to intervention
- Could not readily have been produced by the participant's clinical state or have been due to environmental or other interventions
- Follows a known pattern of response to intervention
- Disappears or decreases with reduction in dose or cessation of intervention and recurs with re-exposure

iii Expectedness

An **unexpected** event is one that has not been documented previously as an established adverse reaction to the study intervention and that is not recognized as part of the natural progression of the disease. A particular event may also be considered unexpected if it has a higher severity grade than what has been documented or identified previously.

6.e Data Collection Procedures for Adverse Events and Unanticipated Problems

The clinical site staff will report all serious adverse events and unanticipated problems on the trial data collection forms. These events include those related to red blood cell transfusion including those specified in the informed consent form and those that are related to recent myocardial infarction. All of the expected SAEs are recorded on the in-hospital and the 30-day follow-up data collection forms. Site personnel are required to report and document all unexpected SAEs and unanticipated problems to the DCC. The DCC will send all unexpected SAEs (as reported by the site) to the study Medical Monitor for final assessment of severity, relatedness, and expectedness. The Medical Monitor will remain masked to the transfusion strategy while making his/her evaluation of the SAE.

6.f Reporting Procedures

All reported SAEs and unanticipated problems will be included in systematic reporting to the DSMB on a semi-annual basis. This includes adverse events and problems previously transmitted through expedited reporting.

The following three classes of events need to be reported to the NHLBI, the DSMB and the Local IRB in an expedited manner: 1) Fatal or life threatening unexpected suspected SAE, 2) Non-fatal, non-life threatening unexpected suspected SAE, and 3) Unanticipated problem. Fatalities that are related to blood transfusions must also be reported to the FDA within 7 days according to the guidelines (http://www.fda.gov/BiologicsBloodVaccines/GuidanceComplianceRegulatoryInformation/Guidances/Blood/ucm074947.htm).

The site personnel will compete and submit a SAE form to the DCC within 72 hours of learning of the event whenever the event is both serious and unexpected. In cases where the event is not serious but places the patient at greater risk of physical, psychological, economic, or social harm, and is both unexpected and related to the study, the site PI will fill out an Unanticipated Problem form within 14 days of learning about the problem. SAE forms are forwarded along with relevant patient history data to the Medical Monitor for review. The study Medical Monitor will assess the severity, relatedness, and expectedness of the event within 48 hours. Following the Medical Monitor's feedback on the expectedness and relatedness of the SAE, the DCC will complete an SAE report categorized as serious, unexpected and related. A report will also be sent for unanticipated problems. The DCC will send the reports to the NHLBI DSMB Executive Secretary and the NHLBI Medical Monitor for review. All reporting (from the time that the Site learns about the event until it is reported to the NHLBI, DSMB, FDA and IRBs) will follow the NHLBI DSMB established timelines as specified in (https://www.nhlbi.nih.gov/research/funding/human-subjects/adverse-event) as shown in Appendix 2. If other SAEs are noted to occur with abnormally high frequency during the trial, these will be reported promptly by the MINT DCC to the DSMB, and the NHLBI DSMB Executive Secretary and the NHLBI Medical Monitor. Upon receipt of an expedited report, the DSMB chair will decide whether the event should be discussed at the next scheduled DSMB meeting or discussed as soon as possible at an ad hoc meeting.

IRB actions regarding the MINT trial will be communicated to the NHLBI Project Officer and NHLBI Executive Secretary in an expedited fashion. If the IRB or ethics board at any MINT site, CCC or

DCC takes action regarding the MINT trial (e.g., the IRB places a hold on the trial or suspends the trial), the site will report this to the CCC within 24 hours of the action. This will be communicated by telephone and with an urgent help desk ticket through the MINT trial data management system. The Site will submit written documentation from the IRB, an explanation of the circumstances, and a plan of action to the CCC within 72 hours. The CCC will promptly communicate this information to the DCC and the NHLBI project officer. The DCC is responsible for notifying and the NHLBI Executive Secretary. A written report describing the IRB decision, the rationale for the decision and the plan of action based on this decision will be submitted to the NHLBI project officer and the NHLBI Executive Secretary within 7 days of the IRB action.

6.g Adverse Event Reporting Period

All randomized patients will be followed by the trial for 30-days from randomization, and vital status will be ascertained at 6 months from study enrollment. Serious adverse events that occur during the 30-day time period and all deaths up to 6 months will be reported.

6.h Post-study Adverse Event

MINT will not collect or report information about adverse events that occur more than 30-days after randomization with the exception of death which will be reported through 6 months after randomization. Reporting of Adverse events will cease at the conclusion of the MINT trial.

E7 Study Outcome Measurements and Ascertainment

7.a Definitions of Outcomes

i Myocardial Reinfarction

Myocardial reinfarction will be classified by the Clinical Events Committee using The Joint European Society of Cardiology/American College of Cardiology Committee definitions. (47) Patients with reinfarction will need to demonstrate a fall in the troponin value and then a subsequent rise of at least 20% with additional evidence (new ECG changes, imaging evidence, clinical history) as in the MI definition to diagnose a new event. Myocardial reinfarction will be classified as ST-segment elevation or non-ST Segment elevation.

ii Death and Cause of Death

For each death, the cause will be determined by the site personnel into one of three categories (47): cardiovascular death (e.g., congestive heart failure, dysrhythmia), noncardiovascular death (e.g., infection, cancer), or undetermined cause of death. Information about the specific cause of death will also be collected.

iii Unscheduled Coronary Revascularization (unstaged)

Ischemia driven, unscheduled coronary revascularization (coronary artery bypass surgery or PCI) within 30 days of randomization will be recorded by the sites. Prior to randomization, the site will record if a coronary revascularization is planned (staged). All coronary revascularization procedures will be recorded, but an elective planned staged procedure will not be included as an outcome. Information about the reason for the procedure will also be collected to ensure that the revascularization was done to treat ischemic heart disease.

iv Readmission to Hospital, overall and for primary cardiac diagnosis

All re-admissions to the hospital that had not been planned prior to randomization will be captured, and the primary diagnosis for each hospitalization will be classified as: ischemic cardiac diagnosis (e.g., myocardial infarction, unstable angina), non-ischemic cardiac diagnosis (e.g. heart failure) or non-cardiac.

v Unstable Angina

The MINT trial sites will use 2014 ACC/AHA Key Data Elements and Definitions for Cardiovascular Endpoint Events in Clinical Trials to define unstable angina. (47) To diagnose unstable angina requires that four criteria be met: 1) worsening ischemic discomfort, 2) unscheduled hospitalization, 3) negative cardiac biomarker, 4) objective evidence of myocardial ischemia.

vi Congestive Heart Failure

Sites personnel will use 2014 ACC/AHA Key Data Elements and Definitions for Cardiovascular Endpoint Events in Clinical Trials to define congestive heart failure. (47) New or worsening symptoms of congestive heart failure on presentation (increasing dyspnea, paroxysmal nocturnal dyspnea, orthopnea), has objective evidence of new or worsening heart failure, and receives initiation or intensification of treatment specifically for heart failure.

vii TIA or Stroke

The 2014 ACC/AHA Key Data Elements and Definitions for Cardiovascular Endpoint Events in Clinical Trials will be used to define stroke. (47) A transient ischemic attack (TIA) is defined as “a transient episode of focal neurological dysfunction caused by brain, spinal cord, or retinal ischemia, without acute infarction.” Stroke is defined on the basis of the presence of acute infarction as demonstrated by imaging or based on the persistence of symptoms more than 24 hours.

viii Deep Venous Thrombosis or Pulmonary Embolism

Deep vein thrombosis will be diagnosed if duplex ultrasound, magnetic resonance venogram (MRV), or venogram is definite or probable positive. Site investigators will record if location is proximal or distal. Pulmonary embolism will be diagnosed with a high probability ventilation perfusion lung scan, CT scan, or pulmonary angiogram.

ix Bleeding

Major bleeding will be defined as 1) fatal bleeding, and/or 2) symptomatic bleeding in a critical area or organ, such as intracranial, intraspinal, intraocular, retroperitoneal, intra- articular or pericardial, or intramuscular with compartment syndrome, and/or 3) bleeding causing a drop in hemoglobin concentration of 2 g/dL or greater (51) from the last hemoglobin concentration prior to randomization to the nadir hemoglobin concentration during hospitalization or up to 30 days post randomization. The drop in hemoglobin concentration will account for each unit of red blood cell transfusion transfused by subtracting 1 g/dL for each unit administered.

x Infections

x.1 Pneumonia

Pneumonia will be diagnosed using CDC criteria (48) which includes radiographic abnormalities and combination of symptoms (i.e., cough), signs (i.e., fever, tachypnea, or laboratory abnormalities (i.e., white blood cell count, hypoxemia).

x.2 Blood Stream Infection

Blood stream infection will be defined using CDC criteria which includes a recognized pathogen cultured from 1 or more blood cultures and organism cultured from blood is not related to an infection at another site, at least 1 of the following signs or symptoms: fever, chills, or hypotension and signs

and symptoms and positive laboratory results are not related to an infection at another site and common skin contaminant is cultured from 2 or more blood cultures drawn on separate occasions.

x.3 Urinary Tract Infection

Urinary Tract Infection will be defined using CDC criteria (48) which include one of the following signs or symptoms: fever (>38.0°C) or localized pain or tenderness, and laboratory evidence for infection.

xi Length of Stay and Intensive Care Unit Days

The trial will collect the number of days post randomization that patient is in the hospital and in intensive care unit.

xii Quality of Life

The EuroQol questionnaire (EQ-5D), a standardized instrument that measures health related quality of life in 5 dimensions: mobility, self-care, usual activities, pain/discomfort and anxiety/depression, will be used as a measure of patient perceived global health status 30 days after randomization.

7.b Ascertainment

Relevant data will be collected from the medical record during the index hospitalization. During the first 3 days following randomization (or hospital discharge if sooner) active surveillance is performed to ensure all occurrences of myocardial reinfarction are detected. It is required that the site obtains troponin levels (every 12 hours for 1 day, and then daily for the next 2 days) and electrocardiograms (daily for the first 3 days). These results, in combination with any additional cardiac biomarkers for necrosis and electrocardiograms and medical findings, will be reviewed by the Clinical Events Committee for each randomized patient, irrespective of a site identified event. We will therefore be able to identify myocardial reinfarction events that were not clinically recognized, in addition to those had been.

Study staff will contact the patient at 30 days to ascertain vital status, administer the quality of life questionnaire, and determine if there has been a subsequent hospital admission. Study staff will obtain and review the medical records for each readmission and record all relevant data and identify the study outcomes. If there is a suspect cardiac event the staff will submit de-identified copies of the documentation (from either the index admission or the readmission) for the Clinical Events Committee

adjudication. Study staff will perform a final follow-up contact at 6 months following randomization to ascertain the patient's vital status.

E8 Baseline and Intervention Clinical Characteristics

Baseline demographic (e.g., age, gender, race), co-morbidity (e.g. cardiovascular risk factors), characteristics of cardiac disease (e.g., left ventricular function, number of coronary vessels obstructed), renal disease, and medications (e.g., aspirin, P2Y12 receptor inhibitor, anticoagulant, beta-blocker statins) will be collected to reflect patient status at the time of randomization. Characteristics describing the study intervention including red blood cell transfusion information (e.g. expiration date, leukoreduction, storage solution, ABO) will be recorded. In hospital, hemoglobin levels and the number and timing of transfusions will be recorded for the two assigned transfusion strategies.

F Statistical Plan

F1 Sample Size Determination and Power

In the MINT pilot trial, the composite 30-day rate of death and myocardial infarction was 16.4%. Higher rates were observed in the Kaiser System preliminary data. Thirty-day rates of 16.4%, 18%, or 20% are reasonable for this trial since the proposed study population will not exclude the sickest patients as was done in the MINT pilot study. Using a two-sided inequality test and a simple chi-square statistic with alpha=0.05, we determined the samples sizes required to provide 80% and 90% power to detect varying relative reductions in the 30-day event rates for death and myocardial infarction between the two assigned treatment groups. Based on these estimates, we plan to enroll a sample of 3500 patients. If the rate of missing outcome data is no more than 5%, the trial would have 3324 patients with analyzable outcome data at 30-days. Assuming an overall event rate of 16.4%, the trial will have 80% power to detect a 20% relative reduction (i.e. 18.2% vs. 14.6%) and >90% power to detect a 25% relative reduction. If the overall event rate is 18%, then the trial will have >80% power to detect a 20% relative reduction (i.e. 20% vs. 16%), and if the overall event rate is 20%, the trial will have close to 90% power to detect 20% relative reduction (i.e. 22.2% vs. 17.8%). We also performed

a simulation study to compute the power for a log binomial model with a random intercept for site and a final alpha-level=0.045 to account for the interim monitoring. We simulated 1000 trials where each trial had 60 strata representing the sites, 56 participants within each stratum (i.e. N=3360 patients in total), and a mean intra-class correlation (ICC) of 0.05 to 0.08. The simulations indicate that the power is comparable for the proposed log binomial model and the simple chi-square statistic used for the original sample size calculations. Based on the simulations, the trial will have 81% power to detect a 20% relative reduction from 18.2% to 14.6%, and 90% power to detect a 25% relative reduction from 18.2% to 14.0%. In addition, the trial will have 85% power to detect a 20% relative reduction from 20% to 16%, and 89% power to detect a 20% relative reduction from 22.2% to 17.8%.

This pragmatic randomized clinical trial has excellent power to detect clinically meaningful differences between the two transfusion strategies with respect to the critical 30-day composite outcome of death and myocardial infarction on both the absolute and the relative scales. After half of the patients have been enrolled and followed, we will monitor the overall event rate in the trial to ensure that the trial has adequate power to detect clinically relevant treatment effects.

F2 Interim Monitoring and Early Stopping

The DSMB will review interim analyses of the outcomes by assigned treatment group annually. The interim monitoring is designed to test for evidence of beneficial effect with either treatment strategy while maintaining the overall type I error at the pre-specified level. Stopping rules are based on the information (i.e. number of primary outcome events) that has accumulated by each inspection time and the shape of the predetermined alpha-spending function. We recommend that the Lan-DeMets approach be used to allocate the type 1 error (i.e. alpha-level) at each interim time point and use of O'Brien Fleming monitoring boundaries. The DSMB may advise early termination of the trial for safety reasons or make other recommendations regarding modifications to the protocol.

The MINT trial will have 4 years of recruitment with a goal of enrolling N=3500 patients by March 2021. With this timeline, we propose that formal statistical interim monitoring will occur in May or June of 2018, 2019, and 2020 based on N=298 patients enrolled through March 1, 2018, N=872 patients enrolled through March 1, 2019, and N=1905 patients enrolled through March 1, 2020, respectively. All calculations assume an overall trial alpha of 0.05.

With an alpha-spending method, the alpha level to be used at any given look is based upon the amount of information available at the monitoring time. Once alpha is calculated, boundaries for the

standardized Z-test statistic are constructed. A standardized Z-test statistic is calculated based on the estimated relative risk from the log-binomial model which includes assigned transfusion strategy (according to the intention to treat principle) as the independent variable. If the observed Z-test statistic crosses the boundary the difference is considered statistically significant at that monitoring time. The "α spending function approach" allows the number and frequency of monitoring times to be changed at any point during the trial.

Based on the anticipated accrual shown in Appendix 3, below is the table of the expected percent of information, estimated number of participants, the Z-test statistic boundary level and the corresponding alpha spent based on the O'Brien-Fleming boundaries.

Estimated Boundary Values at Each Interim Monitoring Time Point

			O'Brien Fleming	
**Stage**	**Information Level**		**Boundary Values**	
	**Proportion**	**Number Recruited**	**Bound**	**Alpha Spent**
**1**	0.0900	298	.	.
**2**	0.2500	872	±4.33	<0.001
**3**	0.5400	1905	±2.84	0.005
**4**	1.0000	3500	±1.97	0.045

The O'Brien and Fleming (1979) repeated significance tests combined with the Lan and DeMets (1983) flexible alpha-spending curves are proposed due to their common usage and their conservative nature when the amount of accumulated data in the trial is small. They are designed to stop earlier in the trial if there is overwhelming evidence that there is a difference between the two treatment groups. The ability to detect a difference between the groups becomes easier as the amount of information in the trial increases.

The form of the O'Brien-Fleming alpha –spending function is:

$$\boldsymbol{\alpha(k) = 2 - 2\phi\left(\frac{Z_{\alpha/2}}{\sqrt{i_k}}\right)}$$

where: α(k) = cumulative alpha at inspection k
k = inspection number
ϕ(x) = the cumulative distribution function of the standard normal curve evaluated at x.

Z_α = the value of the standard normal curve where α is the area in the tail
i_k = the amount of information accumulated in the trial at inspection k

We assume a symmetric 2-sided boundary, with each side spending α=0.025. Below are plots of the cumulative O'Brien-Fleming alpha-spending curve and the corresponding boundary plot. We assume a symmetric 2-sided boundary, with each side spending α=0.025.

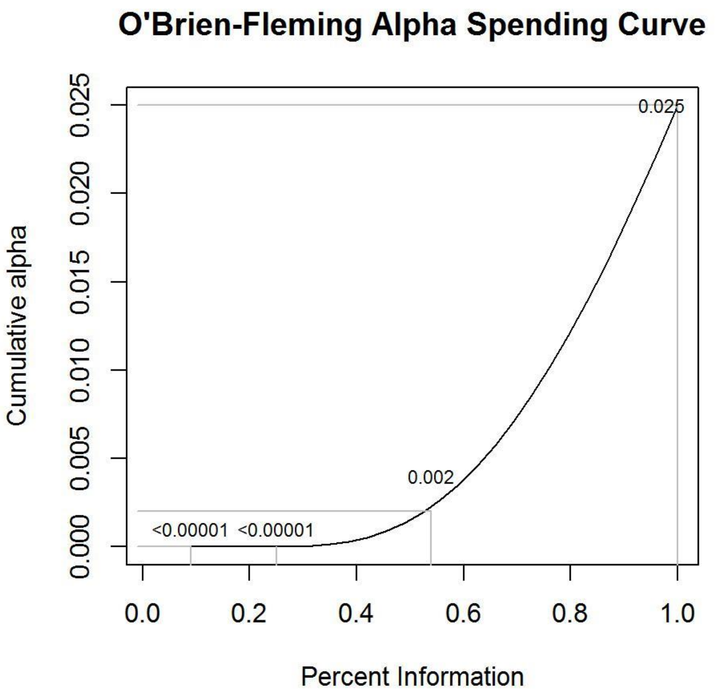

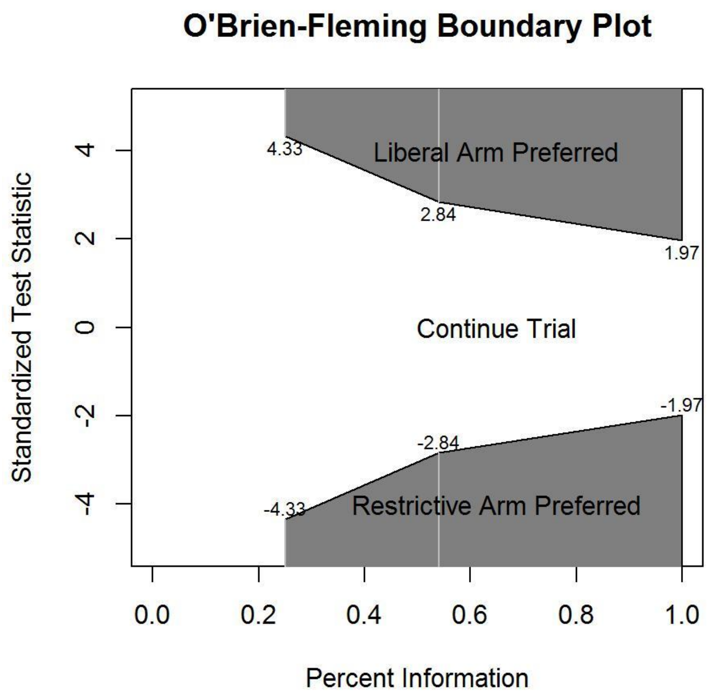

It is possible that the test statistic will cross the monitoring boundary. Statistical interim monitoring results should be taken as one component to the decision as to whether or not to stop a trial. To stop the trial for efficacy, results should be definitive enough to be able to change clinical practice. The DSMB will use the monitoring information to determine its recommendation to NHLBI. The DSMB can recommend that the MINT trial should continue as proposed, that the MINT protocol should be modified based on the results seen in one treatment comparison or in some well-defined subgroup of patients, or that the MINT trial should be terminated early. The final decision to stop trial rests with the NHLBI. If recommendation is to stop the trial, the MINT trial principal investigators shall be consulted before a final decision is made.

In order to ensure that the MINT trial has adequate power, an interim analysis to assess sample size will be conducted after when half of the participants are projected to have completed the 30-day follow-up. Revised sample size estimates will be calculated based on the original power and effect size estimates from the trial hypothesis (80% power to detect a RR=0.80) and on the observed overall event rate (i.e. the two transfusion groups combined). The DSMB will evaluate whether the trial sample size needs to be increased in order to restore power to maintain the ability to detect a clinically meaningful effect size.

Since the MINT trial compares two established transfusion strategies with different resource and cost implications, a null result from a well-powered trial would be important for establishing treatment guidelines and policy. Thus, no interim futility analyses will be conducted in the MINT trial.

F3 Analysis Plan

A Screening Log will be developed to include the number of patients with acute myocardial infarction and hemoglobin level < 10 g/dL, eligibility and enrollment status. Proportions of eligible and enrolled patients will be presented, and reasons why patients are not eligible or enrolled in the trial will be tabulated. Enrollment patterns will be compared across clinical sites and patient groups defined by sex and race/ethnicity. The baseline characteristics and co-interventions of the patients who are randomized in the MINT trial will be described, and characteristics of patients in the two assigned treatment groups will be compared. The proportion of enrolled patients who adhere to the assigned intervention will be ascertained by evaluating the hemoglobin levels and the number of units of blood transfused during the index hospitalization. The intention-to-treat principle will be used to compare the primary outcome (the composite of all-cause mortality and myocardial infarction by 30 days from randomization) and each pre-specified secondary outcome by the randomized transfusion strategy groups. A select number of subgroup analyses will be performed based on baseline factors identified prior to the initiation of the trial.

F4 Statistical Methods

4.a Baseline Characteristics of the Enrolled Patient Sample

Descriptive statistics will be examined for all relevant baseline measures collected on the MINT randomized patients. Transformations of measures will be considered on the basis of distribution diagnostics and outlier analysis. The baseline characteristics and co-interventions of the patients in the overall study will be described using frequencies, proportions, means and standard deviations, or medians and first and third quartiles. Characteristics of patients in the two arms of the trial will be compared using chi-square statistics for categorical variables and t-tests or Wilcoxon rank-sum statistics for continuous variables.

4.b Adherence to Assigned Intervention

The mean hemoglobin concentration at 1, 2, and 3 days post-randomization will be compared between the assigned treatment groups with a Student's t-test. Mixed random effect models will be used to describe the daily mean hemoglobin concentrations during the initial hospitalization following randomization and to compare these repeated measures by assigned treatment group. The mean number of red blood cell transfusions in each randomized treatment group will be described, and a simple Poisson test will be used to test whether the number of units of blood differs significantly by assigned transfusion strategy.

4.c Primary Outcome Analysis

The intention-to-treat principle will be used for all randomized transfusion strategy comparisons of study outcomes. A two-sided test will be used with an alpha-level=0.05 for the primary outcome, the composite of all-cause mortality and myocardial infarction (death/MI) by 30 days from randomization. The 30-day event rate for the primary outcome will be compared by assigned transfusion strategy using an unadjusted log-binomial regression model with a fixed effect variable for assigned treatment strategy and a random effect for clinical site. The primary analysis will involve the estimated relative risk and significance level obtained from the model accounting for interim monitoring and missing data. Event rates, the relative risk and the absolute difference will be from the observed data will also be presented, and 95% confidence intervals will be calculated.

If significant imbalances in baseline risk factors are detected between the two randomized treatment groups ($p<0.05$), multivariable log-binomial regression will be used to adjust for potential confounding factors as a sensitivity analysis. If adherence to the protocol is lower than expected, a per protocol analysis, including only patients who undergo transfusion according to their assignment and adjusting for baseline factors that are associated with adherence to the treatment protocol, will be conducted as a sensitivity analysis.

4.d Analysis of Secondary and Tertiary Outcomes

Event rates, the relative risk and the absolute difference between transfusions strategy groups for the dichotomous secondary and tertiary outcomes will be calculated with their 95% confidence intervals using log-binomial regression methods accounting for clinical site with random effects. The length of hospital stay from randomization and from admission will be analyzed using non-parametric Wilcoxon

rank sum tests, and long-term mortality will be analyzed with Kaplan-Meier methods and the log rank statistic. The Kaplan-Meier estimated mortality rate in each treatment group at 180 days, the absolute difference between the assigned treatment groups at 180 days, and the 95% confidence interval for the difference will be presented. Transfusion strategy comparisons of the EQ-5D quality life measures at 30 days will be based on t-tests or the Wilcoxon rank sum test. No adjustment of the alpha level will be made for the secondary and tertiary analyses. Rather the results will be interpreted based upon the observed findings, their designation as secondary or tertiary outcomes, and in comparison to the primary outcome result.

The secondary outcomes include: 1) all-cause mortality within 30 days; 2) myocardial reinfarction within 30 days; and 3) the composite outcome of all-cause mortality, nonfatal myocardial reinfarction, ischemia driven unscheduled coronary revascularization (percutaneous coronary intervention or coronary artery bypass grafting), or readmission to the hospital for ischemic cardiac diagnosis within 30 days.

The tertiary outcomes include: 1) all-cause mortality, nonfatal myocardial reinfarction, and unstable angina (i.e. acute coronary syndrome) 2) ischemia driven unscheduled coronary revascularization within 30-days; 3) readmission to the hospital for ischemic cardiac diagnosis, 4) congestive heart failure within 30 days; 5) unscheduled readmission to hospital for any reason within 30 days; 6) each of the individual cardiovascular outcomes of stroke, pulmonary embolism or deep venous thrombosis, and bleeding within 30 days; 7) each of the individual infectious outcomes of pneumonia, blood stream, and urinary tract within 30 days; 8) each of the individual in-hospital outcomes of length of hospital stay post randomization and number of days in intensive care unit; 9) patient reported quality of life measures based on the EuroQol questionnaire (EQ-5D) at 30 days; and 10) all-cause mortality at 6-months.

4.e Missing Outcome Data

For the analysis of the primary outcome, standard multiple imputation methods will be used to impute missing 30-day data. We expect that missing 30-day outcome data will be very low in this trial. All patients should have non-missing death and MI data from randomization to hospital discharge. Markov Chain Monte Carlo (MCMC) multiple imputation methods will be used to impute the missing 30-day outcome values (yes/no) for death and MI. Specifically, a log-binomial regression model to estimate the relationship between the outcome and key covariates (observed baseline and

in-hospital variables that are clinically relevant and related to the “missingness” of the 30-day outcomes) in the participants with full data. This model will be used to predict the probability of the outcome for participants who are missing the 30 day outcomes. Based on these probabilities, ten imputed data sets will be created. A log-binomial model with random effects for site will be estimated for each imputed data set, and the results will be pooled to obtain a single estimate of treatment effect with an appropriately adjusted standard error. As a sensitivity analysis, a log-binomial model with random effects for site will be created based on the non-missing 30-day death/MI data. Missing data will not be imputed for the analyses of the secondary and tertiary hypotheses unless critical issues are noted while investigating the missing 30-day data.

4.f Subgroup Analyses

Subgroup analyses will be performed based on baseline factors identified prior to the initiation of the trial. Clinically relevant subgroup variables include: ST segment elevation MI / non ST segment elevation MI, Universal Definition type 1 MI / type 2 MI, type 4b and 4c, baseline hemoglobin level (<8, 8-9, 9-10 g/dL), revascularization for treatment of index MI, bleeding, sex, race/ethnicity (White, Black, Other Race and Hispanic, non-Hispanic), age (<70, ≥70 years old), left ventricular dysfunction, diabetes status, and creatinine clearance (eGFR <30, 30-59, ≥60). The 30-day event rates by assigned transfusion strategy will be compared within each pre-defined subgroup. The reported significance-level will account for the number of treatment comparisons conducted in the study using the false discovery rate (FDR) algorithm developed by Benjamini and Hochberg. In addition, for each subgroup variable, a log-binomial regression model including treatment assignment, subgroup variable and the interaction between the subgroup variable and treatment assignment will be created, and the significance of the interaction term will be used to test whether the randomized treatment effect is significantly modified by the designated subgroup variable. Cox proportional hazards regression models will be created with similar covariates in order to test whether the effect of the randomized transfusion strategy for 6-month mortality varies significantly according to the pre-specified subgroup variables.

4.g Combining Vanguard and Main Trial Data

In 2016, the Canadian Institutes of Health Research (CIHR) funded a MINT Pilot trial. This pilot is coordinated by the University of Montreal, and the clinical sites are in Canada. The DCC and CCC have collaborated with the University of Montreal to ensure that the protocol (eligibility criteria,

interventions, and outcomes) as well as the randomization scheme and the data to be collected are nearly identical to those for the main MINT trial. The CIHR MINT pilot trial was funded to enroll 60 patients. This pilot trial will serve as a “Vanguard phase” for the MINT trial and will allow us to evaluate the feasibility of the MINT protocol. After the MINT protocol is approved by the DSMB/NHLBI and the appropriate ethics committees in Canada, patients randomized at the Canadian Sites will be enrolled as MINT participants. All data collection and data coordination for the MINT participants will occur through the University of Pittsburgh DCC. Patients who are randomized prior to the transition date will remain CIHR pilot trial participants throughout their follow-up. The complete Vanguard datasets housed at the University of Montreal will be transferred to the University of Pittsburgh DCC. The Vanguard patient data will be merged with the MINT trial data based on rigorous comparisons of variable definitions and variable coding. All Vanguard patients will be included in the target sample size and presented in DSMB and published data reports for the MINT trial.

F5 Unblinding Procedures

Patients and providers are not blinded in the MINT trial, and thus unblinding procedures are not required for patient safety. Trial monitors and adjudicators are blinded to transfusion strategy, and the DSMB may choose to be blinded to transfusion strategy as they monitor the outcome data. The Medical Monitor and DSMB can request to be unblinded if they deem this necessary.

G Data Handling and Record Keeping

G1 Confidentiality and Security

Patient identifiers (e.g. name and social security number) will not be sent to the trial DCC, however, some personal health information (e.g. birth date and hospitalization admission date) will be collected centrally. The University of Pittsburgh DCC has a strictly enforced security policy with standard operating procedures addressing key security risk areas. Procedures include password protection, limited access to study computers, encryption, IP restriction, basic or digest authentication, and firewalls between the Internet and the DCC network and servers. Regularly scheduled backups and

archives are performed at least once daily with backup media copies of project files stored off-site in a secure location. Virus detection will be enforced at the DCC. All servers will be connected to uninterruptible power supplies and housed in a computer room with an alarm.

Patient contact information will be maintained exclusively by the local site and stored in a secure location. Only local study staff responsible for the 30 day and 6 month telephone calls will have access to this contact information.

G2 Training

DCC personnel at the University of Pittsburgh will work with CCC personnel at Rutgers / Robert Wood Johnson to provide the training necessary for uniform data collection, protocol compliance, and data processing. Major elements to be covered are inclusion and exclusion criteria, procedures and tools to monitor adherence to protocol, randomization procedures, event adjudication procedures, adverse event reporting, methods for extracting data from different sources, follow-up schedules, what to do about missing information, reporting requirements, and use of the Manual of Operations and study website. Instruction will be provided for entering the data, transmitting them to the DCC, interpreting edit reports and correcting data.

On-going training will be required due to the addition of new clinical sites as well as turnover of staff at the existing clinical sites. The CCC and DCC will utilize webinars and conference calls to conduct detailed distance training sessions. On-line protocol and data collection training modules will be available on the trial website, and focused training sessions will be conducted in conjunction with Steering Committee meetings as required. Throughout the trial, there will be regularly scheduled conference calls with the coordinators and data collectors at all sites to discuss issues that arise with protocol and data collection.

G3 Case Report Forms and Source Documents

The data collection forms for this trial have been developed to facilitate enrollment and follow-up while still collecting the essential information needed to answer the trial hypotheses. A web-based distributed data management system will be used for data entry. Source documents will be maintained at the clinical site.

G4 Records Retention

Trial patient records will be retained at the clinical sites and at the Coordinating Centers for 6 years after MINT data collection is completed.

G5 Performance Monitoring

The DCC will monitor study performance. Routine reports will be developed to monitor the ongoing progress of the study including enrollment and retention. Protocol adherence reports include enrollment of ineligible patients, follow-up data collection outside of protocol-defined windows, and deviations from protocol. The timeliness of data collection and data entry is routinely monitored at the DCC using information entered into the data system (evaluation date and data collection date), and information stored in the systems inventory files (dates of entry, verification, and submission). The DCC will provide reports to the MINT CCC and clinical site coordinators including scheduling and delinquency. Inadequate performance of a site will be reported to the CCC and the site. The DCC and CCC will work with the site to find solutions to challenges. The DCC and CCC will evaluate the performance at that site to determine whether corrections have been made. Should problems persist, the Operations Committee will be notified along with recommendations for resolving persisting inadequacies. Trial and site performance reports will be provided to the sites, the CCC, the Steering Committee, and the DSMB.

H Study Monitoring, Auditing, and Inspecting

H1 Study Monitoring Plan

The CCC will coordinate the study monitoring site visits to each clinical site. In addition, the DCC will conduct data monitoring site visits to clinical sites where specific data issues are identified or data concerns arise based on a risk-based monitoring plan. The risk-based monitoring plan will be based on site performance metrics as well as the results of CCC safety and compliance monitoring visit.

H2 Auditing and Inspecting

During monitoring site visits, CCC personnel will review source documents, including informed consent documents, for a random sample of patients and for a defined subset with an exceptional number of inconsistencies identified through the data monitoring processes to confirm that data are

collected and entered accurately. Problems adhering to study protocols, data collection, entry, and management will be identified and corrective procedures will be addressed.

I Study Administration

I1 Organization and Participating Centers

1.a Clinical Coordinating Center

The Rutgers Robert Wood Johnson School of Medicine, Division of General Internal Medicine is responsible for clinical coordination of the trial. The responsibilities of the Clinical Coordinating Center are to: 1) Provide administrative and fiscal support for the Clinical Sites; 2) Provide technical, patient assessment and Protocol adherence advice to Clinical Site staff; 3) Assist Clinical Sites to correct problems with recruitment, Protocol adherence and data collection; 4) Participate in site visits; 5) Provide advice about any aspect of the trial; and 6) Lead presentation and publication of study results for the scientific and lay press.

A Canadian subsidiary clinical coordinating center to Rutgers Robert Wood Johnson Medical School will be housed at the University of Montreal. The Canadian Coordinating Center will carry out the clinical coordinating center responsibilities listed above for the Canadian sites and will be supervised by Dr. Paul Hebert and Dr. Jeffrey Carson. The Canadian Coordinating Center will have no data coordinating function.

1.b Data Coordinating Center

The Epidemiology Data Center at the University of Pittsburgh is responsible for the data coordination of the trial. The DCC will provide statistical leadership and resources for data management, quality control, and analysis. The responsibilities of the DCC include: 1) Maintain study website and provide all study materials, including the Manual of Procedures (MOP) and Data Collection Forms ; 2) Provide a 24 hour randomization service; 3) Establish and maintain an electronic database to receive study data and to verify for completeness, retrieving any missing data from the centers; 4) Conduct routine data edits; 5) Monitor performance to detect problems with recruitment, protocol adherence and data collection; 6) Participate in site visits; 7) Provide advice about any aspect of the trial; 8) Perform interim and final analyses.

1.c Clinical Consortium

The clinical sites recruiting patients into MINT comprise the Clinical Consortium of participating hospitals. Each site will be led by the Clinical Site Director and Clinical Site Coordinator. The Clinical Site Director and Coordinator will work closely together to assure successful performance of the trial.

The responsibilities of the Clinical Site Director include: 1) To insure that all medical staff involved with the care of myocardial infarction patients are well informed about the trial; 2) To insure that all patients with myocardial infarction are routinely considered for the trial; 3) To insure that the transfusion strategy is followed, i.e., blood is given in the Restrictive Transfusion Strategy only for symptoms from anemia or when the lower threshold is reached and blood is given in the Liberal Transfusion Strategy when Hgb < 10 g/dL; 4) To communicate with Clinical Coordinating Center staff and Data Coordinating Center staff any problems or concerns related to the study; and 5) To assist the Clinical Site Coordinator as necessary.

The responsibilities of the Clinical Site Coordinator include: 1) To identify myocardial infarction patients for the trial; 2) To obtain informed consent from the patient or surrogate; 3) To inform treating staff of the patient's treatment assignment assigned transfusion strategy; 4) To complete data collection forms and process data edit queries; 5) To perform 30 and 180 day telephone follow-up; 6) To obtain and submit all relevant source documents for the Clinical Event Committee adjudication; 7) To participate in telephone calls with the Clinical Coordinating Center; 8) To train assistant site coordination and other staff at the Clinical Site.

I2 Funding Source and Conflicts of Interest

This trial is supported by the National Heart Lung and Blood Institute. All investigators will be required to declare any potential conflicts of interest.

I3 Committees

3.a Steering Committee

The primary decision making body of the MINT trial will be the Steering Committee and will include the Principal Investigators of the CCC and DCC, experts in cardiology, transfusion medicine, biostatistics, and the NHLBI project officers. The Steering Committee will have quarterly conference

calls and annual in-person meetings. The group will focus on the science of the study and to review priorities on a regular basis.

3.b Operations Committee

A smaller committee, the Operations Committee, will be a subset of the Steering Committee that will meet approximately twice per month via conference call and will be charged with reviewing operational and scientific issues for the trial and formulating recommendations for consideration by the full Steering Committee. The Operations Committee will be chaired by Dr. Carson and will include DCC PI, selected Steering Committee members, and the CCC and DCC project directors.

The Operations Committee will be empowered to execute operational decisions, and the Operations Committee Chair will have the authority to set meeting agendas and appoint committee chairs as needed. The committee will ensure that recruitment for the network is progressing as planned for the clinical trial. In addition to reviewing monthly recruitment reports and quarterly follow-up data collection reports, the group will make specific recommendations for strategies to improve recruitment and protocol implementation.

3.c Executive Committee

A small Executive Committee will identify high level study design and implementation issues that are to be discussed by the Steering Committee and to resolve issues that require immediate action. The committee will include the PIs of the CCC and the DCC and the lead clinical investigator from the United States and from Canada.

3.d Clinical Events Committee

A Clinical Events Committee will be responsible for adjudication of myocardial infarctions, unstable angina, ischemia driven unscheduled coronary revascularization, and the classification of cause of death in the trial. Committee members will be masked to transfusion strategy assignment when they review and adjudicate patient event packets.

I4 Subject Stipends or Payments

Study patients will not receive any stipends or payments

I5 Study Timetable

This study will require 5 years for completion. The timeline and benchmarks are as follows:

**Benchmarks**	**Timeline**
Planning and Organization Phase 1: Finalize the Manual of Operations and data collection instruments. Communicate with the Clinical Site Directors at each study site. Obtain IRB approval at clinical sites with assistance from CCC staff. Develop computer software for randomization and data entry.	Months 1-4<br><br>(9/1/16 – 12/31/16)
Planning and Organization Phase 2: Communicate final study protocol and procedures. Plan and schedule training. Hold annual Collaborators' Meeting for Clinical Site Directors and Coordinators. Distribute final forms.	Months 5-6<br><br>(1/1/17 – 2/28/17)
Recruitment and Follow-up Phase: Initiate patient enrollment at the clinical sites. Recruitment will start once a site has IRB approval, an executed contract, and staff have been trained. Continue for 4.0 years, until patient recruitment is completed, or the study is stopped early. Hold annual Collaborators' Meetings.	Month 7- Year 4. 5.<br><br>(3/1/17– 2/28/21)
Close-out and Analysis Phase: Complete follow-up and data cleaning. Perform data analysis. Discuss results at Collaborators' Meeting. Present trial results.	Year 4. 5<br><br>(3/1/21 – 8/31/21)

The planned accrual timeline, including the target and the cumulative target enrollment for each quarter, is provided in Appendix 3.

J Publication Plan

The Publications Committee will include physicians, scientists, and statisticians involved with the MINT trial. The chair of the committee will rotate bi-annually. This committee will be charged with coordinating the publication of study results and ensuring that these publications move forward according to schedule. Our plan is that the primary results of the trial will be submitted for publication within 9 months of the completion the trial. This will allow three months for data management and cleaning, three months for analysis and three months for manuscript preparation.

Dr. Carson will coordinate the effort for the primary results paper and the author byline will include the writing team “and the MINT Trial Investigators”. The primary results writing team will be comprised of the members of the Steering Committee. An appendix listing all of the MINT investigators including the Steering Committee members, the Site Investigators, the NIH program officers, the DSMB and other relevant contributors to the trial will appear at the end of the paper.

There will be additional papers that evaluate secondary hypotheses. The Publications Committee will review proposals for all non-primary papers. Approval of a paper proposal will require that the topic is clinically relevant, the paper does not overlap with an existing topic, and the general methodology is sound. Secondary papers will have individually named authors and will include “for the MINT Trial Investigators” in the author byline. The investigators who propose the concept will automatically be part of the writing team. Investigators from the Steering Committee and the Sites will have the opportunity to sign up for proposed writing teams. If more than 12 investigators sign up for an individual writing group, the Publications Committee will allocate investigators to writing teams based on contributions to the trial and expertise on the specific topic.

In order to ensure that the trial is using its resources to report the topics of greatest clinical relevance, the trial Investigators will be asked for ideas for papers and the Steering Committee will be asked to identify the ten most important papers. If a paper on this list is not moving forward according to schedule, the Publication Committee will notify the Steering Committee and may recommend a change in leadership for the paper. The Steering Committee will make final decisions about the leadership for papers.

K References

1. Sullivan MT, Cotten R, Read EJ, Wallace EL. Blood collection and transfusion in the United States in 2001. Transfusion (Paris). 2007;47(3):385-94. PubMed PMID: 17319817.

2. Organization. WH. 10 facts on blood transfusion: World Health Organization; 2015 [cited 2015 September 11, 2015]. Available from: http://www.who.int/features/factfiles/blood_transfusion/en/.

3. Heddle NLY, ; Barty, R. Ontario Data Collection Startegy: Proof of Concept. Submitted to MOHLTC. Submitted. 2013.

4. Shehata NF, A,; Lawrence, N.; Rothwell, D.M.; Hebert, P.; Fergusson D. Use of administrative data to determine the recipients and trends of blood transfusion. Submitted. 2013.

5. Shander A, Hofmann A, Ozawa S, Theusinger OM, Gombotz H, Spahn DR. Activity-based costs of blood transfusions in surgical patients at four hospitals. Transfusion (Paris). 2010;50(4):753-65. Epub 2009/12/17. doi: TRF2518 [pii]

10.1111/j.1537-2995.2009.02518.x. PubMed PMID: 20003061.

6. Zou S, Stramer SL, Notari EP, Kuhns MC, Krysztof D, Musavi F, Fang CT, Dodd RY. Current incidence and residual risk of hepatitis B infection among blood donors in the United States. Transfusion (Paris). 2009;49(8):1609-20. Epub 2009/05/06. doi: 10.1111/j.1537-2995.2009.02195.x. PubMed PMID: 19413732.

7. Zou S, Dorsey KA, Notari EP, Foster GA, Krysztof DE, Musavi F, Dodd RY, Stramer SL. Prevalence, incidence, and residual risk of human immunodeficiency virus and hepatitis C virus infections among United States blood donors since the introduction of nucleic acid testing. Transfusion (Paris). 2010;50(7):1495-504. Epub 2010/03/30. doi: 10.1111/j.1537-2995.2010.02622.x. PubMed PMID: 20345570.

8. Rosamond W, Flegal K, Friday G, Furie K, Go A, Greenlund K, Haase N, Ho M, Howard V, Kissela B, Kittner S, Lloyd-Jones D, McDermott M, Meigs J, Moy C, Nichol G, O'Donnell CJ, Roger V, Rumsfeld J, Sorlie P, Steinberger J, Thom T, Wasserthiel-Smoller S, Hong Y. Heart disease and stroke statistics--2007 update: a report from the American Heart Association Statistics Committee and Stroke Statistics Subcommittee. Circulation. 2007;115(5):e69-171. Epub 2006/12/30. doi: CIRCULATIONAHA.106.179918 [pii]

10.1161/CIRCULATIONAHA.106.179918. PubMed PMID: 17194875.

9. Alexander KP, Chen AY, Wang TY, Rao SV, Newby LK, LaPointe NM, Ohman EM, Roe MT, Boden WE, Harrington RA, Peterson ED. Transfusion practice and outcomes in non-ST-segment elevation acute coronary syndromes. Am Heart J. 2008;155(6):1047-53. Epub 2008/06/03. doi: S0002-8703(08)00055-0 [pii]

10.1016/j.ahj.2008.01.009. PubMed PMID: 18513518.

10. Salisbury AC, Amin AP, Reid KJ, Wang TY, Masoudi FA, Chan PS, Alexander KP, Bach RG, Spertus JA, Kosiborod M. Hospital-acquired anemia and in-hospital mortality in patients with acute myocardial infarction. Am Heart J. 2011;162(2):300-9 e3. Epub 2011/08/13. doi: 10.1016/j.ahj.2011.05.021. PubMed PMID: 21835291.

11. Hebert PC, Wells G, Blajchman MA, Marshall J, Martin C, Pagliarello G, Tweeddale M, Schweitzer I, Yetisir E. A multicenter, randomized, controlled clinical trial of transfusion requirements in critical care. Transfusion Requirements in Critical Care Investigators, Canadian Critical Care Trials Group [see comments]. N Engl J Med. 1999;340(6):409-17. PubMed PMID: 219.

12. Hajjar LA, Vincent JL, Galas FR, Nakamura RE, Silva CM, Santos MH, Fukushima J, Kalil Filho R, Sierra DB, Lopes NH, Mauad T, Roquim AC, Sundin MR, Leao WC, Almeida JP, Pomerantzeff PM, Dallan LO, Jatene FB, Stolf NA, Auler JO, Jr. Transfusion requirements after cardiac surgery: the TRACS randomized controlled trial. JAMA. 2010;304(14):1559-67. Epub 2010/10/14. doi: 304/14/1559 [pii]

10.1001/jama.2010.1446. PubMed PMID: 20940381.

13. Carson JL, Terrin ML, Noveck H, Sanders DW, Chaitman BR, Rhoads GG, Nemo G, Dragert K, Beaupre L, Hildebrand K, Macaulay W, Lewis C, Cook DR, Dobbin G, Zakriya KJ, Apple FS, Horney RA, Magaziner J. Liberal or restrictive transfusion in high-risk patients after hip surgery. The New England journal of medicine. 2011;365(26):2453-62. Epub 2011/12/16. doi: 10.1056/NEJMoa1012452. PubMed PMID: 22168590; PMCID: 3268062.

14. Villanueva C, Colomo A, Bosch A, Concepcion M, Hernandez-Gea V, Aracil C, Graupera I, Poca M, Alvarez-Urturi C, Gordillo J, Guarner-Argente C, Santalo M, Muniz E, Guarner C. Transfusion strategies for acute upper gastrointestinal bleeding. N Engl J Med. 2013;368(1):11-21. doi: 10.1056/NEJMoa1211801. PubMed PMID: 23281973.

15. Carson JL, Grossman BJ, Kleinman S, Tinmouth AT, Marques MB, Fung MK, Holcomb JB, Illoh O, Kaplan LJ, Katz LM, Rao SV, Roback JD, Shander A, Tobian AA, Weinstein R, Swinton McLaughlin LG, Djulbegovic B, Clinical Transfusion Medicine Committee of the A. Red blood cell transfusion: a clinical practice guideline from the AABB*. Ann Intern Med. 2012;157(1):49-58. doi: 10.7326/0003-4819-157-1-201206190-00429. PubMed PMID: 22751760.

16. Carson JLG, G.; Heddle, N.M.; Grossman, B.J.; Cohn, C.S.; Fung, M.K.; Gersheimer, T.; Holcomb, J.B.; Kaplan, L.J.; Katz, L.M.; Peterson, N.; Ramsey, G.; Rao, S.V.; Roback, J.D.; Shander, A.; Tobian, A.A.R.;. Clinical practice guidelines from the AABB: Red bloodcell transfuison thresholds and storage. 2016;in press.

17. Anderson HT, Kessinger JM, McFarland WJ, Jr., Laks H, Geha AS. Response of the hypertrophied heart to acute anemia and coronary stenosis. Surgery. 1978;84(1):8-15. PubMed PMID: 272.

18. Hagl S, Heimisch W, Meisner H, Erben R, Baum M, Mendler N. The effect of hemodilution on regional myocardial function in the presence of coronary stenosis. Basic Res Cardiol. 1977;72(4):344-64. PubMed PMID: 9839.

19. Wilkerson DK, Rosen AL, Sehgal LR, Gould SA, Sehgal HL, Moss GS. Limits of cardiac compensation in anemic baboons. Surgery. 1988;103(6):665-70. PubMed PMID: 232.

20. Carson JL, Duff A, Poses RM, Berlin JA, Spence RK, Trout R, Noveck H, Strom BL. Effect of anaemia and cardiovascular disease on surgical mortality and morbidity. Lancet. 1996;348(9034):1055-60. Epub 1996/10/19. doi: S0140673696043309 [pii]. PubMed PMID: 8874456.

21. Silvain J, Abtan J, Kerneis M, Martin R, Finzi J, Vignalou JB, Barthelemy O, O'Connor SA, Luyt CE, Brechot N, Mercadier A, Brugier D, Galier S, Collet JP, Chastre J, Montalescot G. Impact of red blood cell transfusion on platelet aggregation and inflammatory response in anemic coronary and noncoronary patients: the TRANSFUSION-2 study (impact of transfusion of red blood cell on platelet activation and aggregation studied with flow cytometry use and light transmission aggregometry). J Am Coll Cardiol. 2014;63(13):1289-96. doi: 10.1016/j.jacc.2013.11.029. PubMed PMID: 24361322.

22. Rodman T, Close HP, Purcell MK. The oxyhemoglobin dissociation curve in anemia. Ann Intern Med. 1960;52:295-309. PubMed PMID: 731.

23. Kennedy AC, Valtis DJ. The oxygen dissociation curve in anemia of various types. JClinInvest. 1954;33:1372-81. PubMed PMID: 735.

24. McMahon TJ, Moon RE, Luschinger BP, Carraway MS, Stone AE, Stolp BW, Gow AJ, Pawloski JR, Watke P, Singel DJ, Piantadosi CA, Stamler JS. Nitric oxide in the human respiratory cycle. Nat Med. 2002;8(7):711-7. doi: 10.1038/nm718. PubMed PMID: 12042776.

25. LaCelle PL. Alteration of deformability of the erythrocyte membrane in stored blood. Transfusion (Paris). 1969;9:238-45. PubMed PMID: 4555.

26. Simchon S, Jan KM, Chien S. Influence of reduced red cell deformability on regional blood flow. Am J Physiol. 1987;253:H898-H903. PubMed PMID: 356.

27. Marik PE, Corwin HL. Efficacy of red blood cell transfusion in the critically ill: a systematic review of the literature. Crit Care Med. 2008;36(9):2667-74. Epub 2008/08/06. doi: 10.1097/CCM.0b013e3181844677. PubMed PMID: 18679112.

28. Chatterjee SW, J; Sharma,A.;Lichstein, E.; Mukherjee,D. Blood Transfusion is Associated with Increased Mortality in Myocardial Infarction-A Meta-analysis and Diversity-Adjusted Study Sequential Analysis. Arch Intern Med. 2012;172.

29. Garfinkle M, Lawler PR, Filion KB, Eisenberg MJ. Red blood cell transfusion and mortality among patients hospitalized for acute coronary syndromes: a systematic review. Int J Cardiol. 2013;164(2):151-7. doi: 10.1016/j.ijcard.2011.12.118. PubMed PMID: 22305811.

30. Wu WC, Rathore SS, Wang Y, Radford MJ, Krumholz HM. Blood transfusion in elderly patients with acute myocardial infarction. N Engl J Med. 2001;345(17):1230-6. PubMed PMID: 9513.

31. Carson JL, Reynolds RC, Klein HG. Bad bad blood? Crit Care Med. 2008;36(9):2707-8. Epub 2008/08/30. doi: 10.1097/CCM.0b013e3181843ee7

00003246-200809000-00047 [pii]. PubMed PMID: 18728495.

32. Carson JL, Carless PA, Hebert PC. Transfusion thresholds and other strategies for guiding allogeneic red blood cell transfusion. Cochrane Database Syst Rev. 2012;4:CD002042. doi: 10.1002/14651858.CD002042.pub3. PubMed PMID: 22513904.

33. Carson JL, Carless PA, Hebert PC. Outcomes using lower vs higher hemoglobin thresholds for red blood cell transfusion. JAMA. 2013;309(1):83-4. doi: 10.1001/jama.2012.50429. PubMed PMID: 23280228.

34. Carson JL, Stanworth SJ, Roubinian N, Fergusson DA, Triulzi D, Doree C, Hebert PC. Transfusion thresholds and other strategies for guiding allogeneic red blood cell transfusion. Cochrane Database Syst Rev. 2016;10:CD002042. doi: 10.1002/14651858.CD002042.pub4. PubMed PMID: 27731885.

35. Docherty AB, O'Donnell R, Brunskill S, Trivella M, Doree C, Holst L, Parker M, Gregersen M, Pinheiro de Almeida J, Walsh TS, Stanworth SJ. Effect of restrictive versus liberal transfusion strategies on outcomes in patients with cardiovascular disease in a non-cardiac surgery setting: systematic review and meta-analysis. BMJ. 2016;352:i1351. doi: 10.1136/bmj.i1351. PubMed PMID: 27026510; PMCID: PMC4817242.

36. Cooper HA, Rao SV, Greenberg MD, Rumsey MP, McKenzie M, Alcorn KW, Panza JA. Conservative versus liberal red cell transfusion in acute myocardial infarction (the CRIT Randomized Pilot Study). Am J Cardiol. 2011;108(8):1108-11. Epub 2011/07/28. doi: 10.1016/j.amjcard.2011.06.014. PubMed PMID: 21791325.

37. Thygesen K, Alpert JS, Jaffe AS, Simoons ML, Chaitman BR, White HD, Joint ESCAAHAWHFTFfUDoMI, Authors/Task Force Members C, Thygesen K, Alpert JS, White HD, Biomarker S, Jaffe AS, Katus HA, Apple FS, Lindahl B, Morrow DA, Subcommittee ECG, Chaitman BR, Clemmensen PM, Johanson P, Hod H, Imaging S, Underwood R, Bax JJ, Bonow JJ, Pinto F, Gibbons RJ, Classification S, Fox KA, Atar D, Newby LK, Galvani M, Hamm CW, Intervention S, Uretsky BF, Steg PG, Wijns W, Bassand JP, Menasche P, Ravkilde J, Trials, Registries S, Ohman EM, Antman EM, Wallentin LC, Armstrong PW, Simoons ML, Trials, Registries S, Januzzi JL, Nieminen MS, Gheorghiade M, Filippatos G, Trials, Registries S, Luepker RV, Fortmann SP, Rosamond WD, Levy D, Wood D, Trials, Registries S, Smith SC, Hu D, Lopez-Sendon JL, Robertson

RM, Weaver D, Tendera M, Bove AA, Parkhomenko AN, Vasilieva EJ, Mendis S, Guidelines ESCCfP, Bax JJ, Baumgartner H, Ceconi C, Dean V, Deaton C, Fagard R, Funck-Brentano C, Hasdai D, Hoes A, Kirchhof P, Knuuti J, Kolh P, McDonagh T, Moulin C, Popescu BA, Reiner Z, Sechtem U, Sirnes PA, Tendera M, Torbicki A, Vahanian A, Windecker S, Document R, Morais J, Aguiar C, Almahmeed W, Arnar DO, Barili F, Bloch KD, Bolger AF, Botker HE, Bozkurt B, Bugiardini R, Cannon C, de Lemos J, Eberli FR, Escobar E, Hlatky M, James S, Kern KB, Moliterno DJ, Mueller C, Neskovic AN, Pieske BM, Schulman SP, Storey RF, Taubert KA, Vranckx P, Wagner DR. Third universal definition of myocardial infarction. J Am Coll Cardiol. 2012;60(16):1581-98. doi: 10.1016/j.jacc.2012.08.001. PubMed PMID: 22958960.

38. Miller YB, G.; Benjamin, R.; Eklund, D.K.; et al. Practice Guidelines for Blood Transfusion; A Complilation from Recent Peer-Reivewed Literature. American Red Cross. 2nd ed: American Red Cross; 2007.

39. Carson JL, Grossman BJ, Kleinman S, Tinmouth AT, Marques MB, Fung MK, Holcomb JB, Illoh O, Kaplan LJ, Katz LM, Rao SV, Roback JD, Shander A, Tobian AA, Weinstein R, Swinton McLaughlin LG, Djulbegovic B. Red Blood Cell Transfusion: A Clinical Practice Guideline From the AABB. Annals of internal medicine. 2012. Epub 2012/03/29. doi: 10.1059/0003-4819-156-12-201206190-00429. PubMed PMID: 22454395.

40. Qaseem A, Humphrey LL, Fitterman N, Starkey M, Shekelle P, Clinical Guidelines Committee of the American College of P. Treatment of anemia in patients with heart disease: a clinical practice guideline from the American College of Physicians. Ann Intern Med. 2013;159(11):770-9. doi: 10.7326/0003-4819-159-11-201312030-00009. PubMed PMID: 24297193.

41. American College of Emergency P, Society for Cardiovascular A, Interventions, O'Gara PT, Kushner FG, Ascheim DD, Casey DE, Jr., Chung MK, de Lemos JA, Ettinger SM, Fang JC, Fesmire FM, Franklin BA, Granger CB, Krumholz HM, Linderbaum JA, Morrow DA, Newby LK, Ornato JP, Ou N, Radford MJ, Tamis-Holland JE, Tommaso CL, Tracy CM, Woo YJ, Zhao DX, Anderson JL, Jacobs AK, Halperin JL, Albert NM, Brindis RG, Creager MA, DeMets D, Guyton RA, Hochman JS, Kovacs RJ, Kushner FG, Ohman EM, Stevenson WG, Yancy CW. 2013 ACCF/AHA guideline for the management of ST-elevation myocardial infarction: executive summary: a report of the American College of Cardiology Foundation/American Heart Association Task Force on Practice Guidelines. J Am Coll Cardiol. 2013;61(4):485-510. doi: 10.1016/j.jacc.2012.11.018. PubMed PMID: 23256913.

42. O'Gara PT, Kushner FG, Ascheim DD, Casey DE, Jr., Chung MK, de Lemos JA, Ettinger SM, Fang JC, Fesmire FM, Franklin BA, Granger CB, Krumholz HM, Linderbaum JA, Morrow DA, Newby LK, Ornato JP, Ou N, Radford MJ, Tamis-Holland JE, Tommaso JE, Tracy CM, Woo YJ, Zhao DX, Force CAT. 2013 ACCF/AHA guideline for the management of ST-elevation myocardial infarction: executive summary: a report of the American College of Cardiology Foundation/American Heart Association Task Force on Practice Guidelines. Circulation. 2013;127(4):529-55. doi: 10.1161/CIR.0b013e3182742c84. PubMed PMID: 23247303.

43. Hamm CW, Bassand JP, Agewall S, Bax J, Boersma E, Bueno H, Caso P, Dudek D, Gielen S, Huber K, Ohman M, Petrie MC, Sonntag F, Uva MS, Storey RF, Wijns W, Zahger D, Guidelines ESCCfP, Bax JJ, Auricchio A, Baumgartner H, Ceconi C, Dean V, Deaton C, Fagard R, Funck-

Brentano C, Hasdai D, Hoes A, Knuuti J, Kolh P, McDonagh T, Moulin C, Poldermans D, Popescu BA, Reiner Z, Sechtem U, Sirnes PA, Torbicki A, Vahanian A, Windecker S, Document R, Windecker S, Achenbach S, Badimon L, Bertrand M, Botker HE, Collet JP, Crea F, Danchin N, Falk E, Goudevenos J, Gulba D, Hambrecht R, Herrmann J, Kastrati A, Kjeldsen K, Kristensen SD, Lancellotti P, Mehilli J, Merkely B, Montalescot G, Neumann FJ, Neyses L, Perk J, Roffi M, Romeo F, Ruda M, Swahn E, Valgimigli M, Vrints CJ, Widimsky P. ESC Guidelines for the management of acute coronary syndromes in patients presenting without persistent ST-segment elevation: The Task Force for the management of acute coronary syndromes (ACS) in patients presenting without persistent ST-segment elevation of the European Society of Cardiology (ESC). Eur Heart J. 2011;32(23):2999-3054. doi: 10.1093/eurheartj/ehr236. PubMed PMID: 21873419.

44. Carson JL, Guyatt G, Heddle NM, Grossman BJ, Cohn CS, Fung MK, Gernsheimer T, Holcomb JB, Kaplan LJ, Katz LM, Peterson N, Ramsey G, Rao SV, Roback JD, Shander A, Tobian AA. Clinical Practice Guidelines From the AABB: Red Blood Cell Transfusion Thresholds and Storage. JAMA. 2016. doi: 10.1001/jama.2016.9185. PubMed PMID: 27732721.

45. Alexander J, Cifu AS. Transfusion of Red Blood Cells. JAMA. 2016. doi: 10.1001/jama.2016.12870. PubMed PMID: 27732722.

46. Rao SV, Chiswell K, Sun JL, Granger CB, Newby LK, Van de Werf F, White HD, Armstong PW, Califf RM, Harrington RA. International variation in the use of blood transfusion in patients with non-ST-segment elevation acute coronary syndromes. Am J Cardiol. 2008;101(1):25-9. Epub 2007/12/26. doi: S0002-9149(07)01822-X [pii]

10.1016/j.amjcard.2007.07.042. PubMed PMID: 18157960.

47. Spitalnik SL, Triulzi D, Devine DV, Dzik WH, Eder AF, Gernsheimer T, Josephson CD, Kor DJ, Luban NL, Roubinian NH, Mondoro T, Welniak LA, Zou S, Glynn S, State of the Science in Transfusion Medicine Working G. 2015 proceedings of the National Heart, Lung, and Blood Institute's State of the Science in Transfusion Medicine symposium. Transfusion (Paris). 2015. doi: 10.1111/trf.13250. PubMed PMID: 26260861.

48. Vlaar AP, Juffermans NP. Transfusion-related acute lung injury: a clinical review. Lancet. 2013. doi: 10.1016/S0140-6736(12)62197-7. PubMed PMID: 23642914.

49. Klein HG, Spahn DR, Carson JL. Red blood cell transfusion in clinical practice. Lancet. 2007;370(9585):415-26. Epub 2007/08/07. doi: S0140-6736(07)61197-0 [pii]

10.1016/S0140-6736(07)61197-0. PubMed PMID: 17679019.

50. Bassand JP, Hamm CW, Ardissino D, Boersma E, Budaj A, Fernandez-Aviles F, Fox KA, Hasdai D, Ohman EM, Wallentin L, Wijns W. Guidelines for the diagnosis and treatment of non-ST-segment elevation acute coronary syndromes. Eur Heart J. 2007;28(13):1598-660. Epub 2007/06/16. doi: 10.1093/eurheartj/ehm161. PubMed PMID: 17569677.

51. Schulman S, Kearon C, Subcommittee on Control of Anticoagulation of the S, Standardization Committee of the International Society on T, Haemostasis. Definition of major bleeding in clinical investigations of antihemostatic medicinal products in non-surgical patients. J Thromb Haemost. 2005;3(4):692-4. doi: 10.1111/j.1538-7836.2005.01204.x. PubMed PMID: 15842354.

L Appendices

L1 Appendix 1: MINT Data Elements by Study Time Point

	Screening	Baseline	Randomization	12 hours Post Randomization	Day 1	Day 2	Day 3	Daily Up to 30 Days	Hospital Discharge/ 30 Days	30 Day Follow-up	6 Month Follow-up
**Confirm Eligibility**	X										
**Informed Consent**	X										
**Hemoglobin Level**[a]		X[b,c]			X[b]	X[b]	X[b]	X			
**Troponin Level**[a]		X[b,c]		X[b]	X[b]	X[b]	X[b]	X			
**ECG**[a]		X[b,c]			X[b]	X[b]	X[b]	X			
**Demographics**	X										
**Medical History**		X									
**Medications**		X									
**Implement Transfusion Strategy**			X--X								
**RBC Transfusions**[d]			X--X								
**Study Outcomes**									X	X	X[e]
**Quality of Life (EQ-5D)**										X	
**Vital Status**									X	X	X
**Assessment of AEs and UPs Safety Monitoring**		X---X									
[a] in addition to required time points, all performed for clinical reasons are collected											
[b] required, if still in hospital											
[c] within the 24 hours prior to randomization											
[d] including unit information (e.g, leukoreduction)											
[d] mortality outcome only											

L2 Appendix 2: SAE and UP Event Reporting Timelines

What Event is Reported	When is Event Reported	By Whom is Event Reported	To Whom is Event Reported
Fatal or life-threatening unexpected, suspected serious adverse reactions	Within **7 calendar days** of initial receipt of information	Investigator	Local/internal IRBs<br>NHLBI and/or Data Coordinating Center (DCC)
		Sponsor or designee[1]	FDA (if IND study)
Non-fatal, non-life-threatening unexpected, suspected serious adverse reactions	Within **15 calendar days** of initial receipt of information	Investigator	Local/internal IRBs/Institutional Officials<br>NHLBI and/or DCC
		Sponsor or designee	FDA (IND/Marketed Products)<br>All participating investigators
Unanticipated adverse device effects	Within **10** working days of investigator first learning of effect	Investigator	Local/internal IRBs<br>NHLBI and/or DCC
		Sponsor or designee	FDA (if IDE study)
Unanticipated Problem that is not an SAE	Within **14 days** of the investigator becoming aware of the problem	Investigator	Local/internal IRBs/Institutional Officials,<br>NHLBI and/or DCC
All Unanticipated Problems[2]	Within **30 days** of the IRB's receipt of the report of the UP from the investigator.	IRB	OHRP
		Investigator[3]	External IRBs

1. Designee is appointed by the sponsor; for example, DCC, CRO.
2. Per OHRP guidance: only when a particular AE or series of AEs is determined to meet the criteria for an UP should a report of the AE(s) be submitted to the IRB at each institution under the HHS regulations at 45 CFR part 46. Typically, such reports to the IRBs are submitted by investigators.
3. Investigators should also take into account local IRB guidance if reporting timelines for UPs are shorter than OHRP guidance

L3 Appendix 3: Accrual Timeline Milestones

The principles guiding the accrual timeline are as follows. The specific target numbers are provided in the table.

- Recruitment will start 6 months after funding of the trial
- 50% of the projected sites will initiate recruitment between month 6 and 12
- All sites will be recruiting by month 18
- Recruitment will extend for 4 years
- The recruitment rate will get faster over time.
- Total sample size is 3500 patients.

Date	Proposed Target Accrual	Proposed Cumulative Accrual
3/1/17	Start	Start
6/1/17	43	43
9/1/17	63	106
12/1/17	86	192
3/1/18	106	298
6/1/18	121	419
9/1/18	136	555
12/1/18	151	706
3/1/19	166	872
6/1/19	191	1063
9/1/19	227	1290
12/1/19	275	1565
3/1/20	340	1905
6/1/20	380	2285
9/1/20	400	2685
12/1/20	405	3090
3/1/21	410	3500

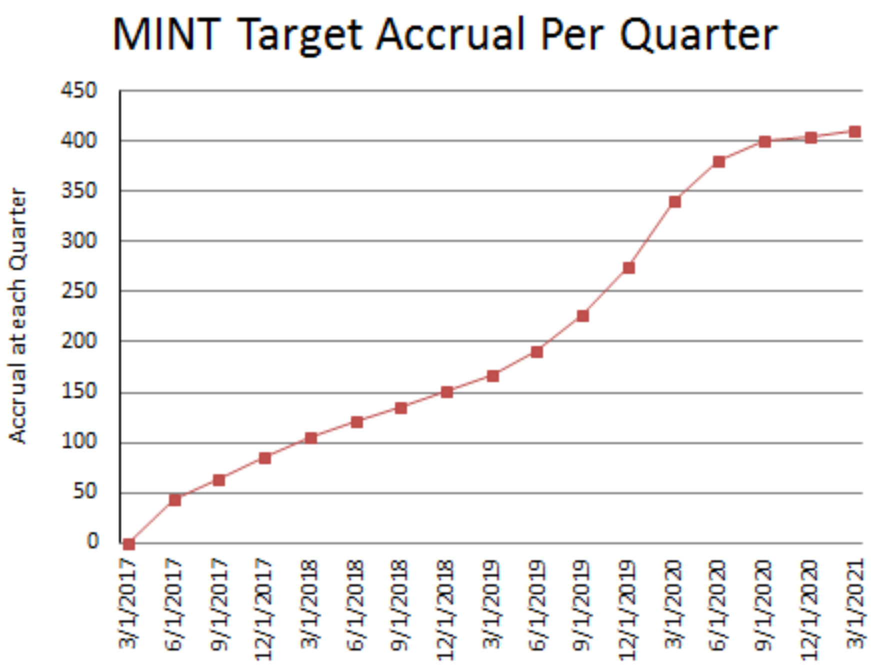
MINT Target Accrual Per Quarter
Accrual at each Quarter
450
400
350
300
250
200
150
100
50
0
3/1/2017
6/1/2017
9/1/2017
12/1/2017
3/1/2018
6/1/2018
9/1/2018
12/1/2018
3/1/2019
6/1/2019
9/1/2019
12/1/2019
3/1/2020
6/1/2020
9/1/2020
12/1/2020
3/1/2021

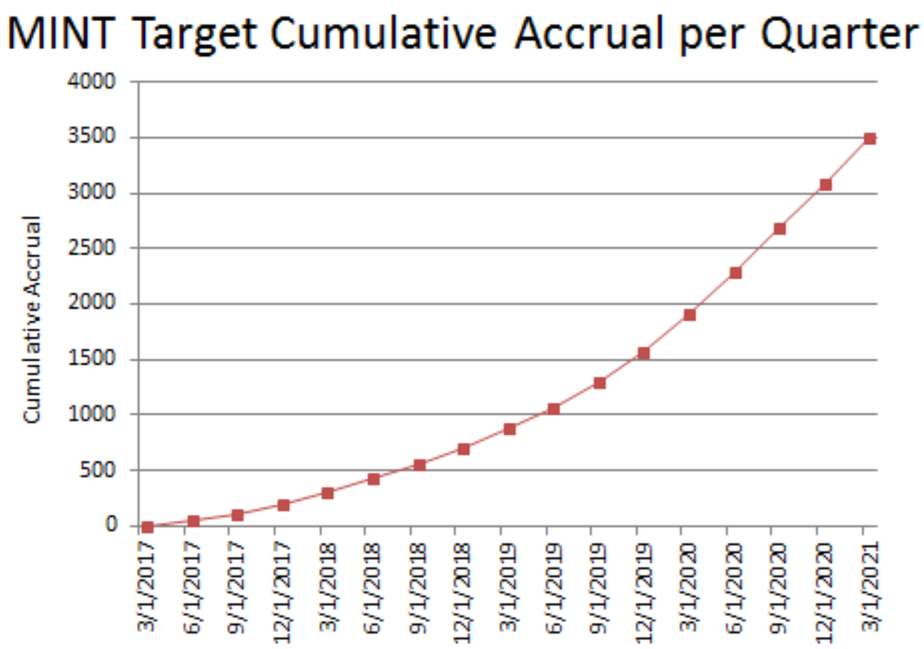
MINT Target Cumulative Accrual per Quarter
Cumulative Accrual
4000
3500
3000
2500
2000
1500
1000
500
0
3/1/2017
6/1/2017
9/1/2017
12/1/2017
3/1/2018
6/1/2018
9/1/2018
12/1/2018
3/1/2019
6/1/2019
9/1/2019
12/1/2019
3/1/2020
6/1/2020
9/1/2020
12/1/2020
3/1/2021

MINT

MYOCARDIAL ISCHEMIA AND TRANSFUSION

Jeffrey L Carson, MD
Study Chair
Principal Investigator, Clinical Coordinating Center
Rutgers Robert Wood Johnson Medical School

Maria Mori Brooks, PhD
Principal Investigator, Data Coordinating Center
University of Pittsburgh

TABLE OF CONTENTS

A INTRODUCTION 8

A1 STATEMENT OF COMPLIANCE 8

A2 STUDY ABSTRACT 9

A3 PRIMARY HYPOTHESIS 9

A4 PURPOSE OF THE STUDY PROTOCOL 10

A5 SCHEMATIC OF STUDY DESIGN 11

A6 KEY ROLES 12

B BACKGROUND 13

B1 PRIOR LITERATURE AND STUDIES 13

B2 RATIONALE FOR THIS STUDY: EQUIPOISE 18

C STUDY OBJECTIVES 18

C1 PRIMARY AIM 18

C2 SECONDARY AIMS 19

C3 TERTIARY AIMS 19

C4 RATIONALE FOR THE SELECTION OF OUTCOME MEASURES 20

D STUDY DESIGN 21

D1 OVERVIEW OR DESIGN SUMMARY 21

D2 SUBJECT SELECTION AND WITHDRAWAL 22

2.a Inclusion Criteria 22

2.b Exclusion Criteria 23

2.c Ethical Considerations 23

2.d Randomization Method and Blinding *23*

2.e Subject Recruitment Plans and Consent Process *24*

2.f Risks and Benefits *24*

2.g Early Withdrawal of Subjects from Transfusion Strategy and Trial Procedures *25*

2.h When and How to Withdraw Subjects *25*

2.i Data Collection and Follow-up for Withdrawn Subjects *25*

D3 TRIAL TRANSFUSION STRATEGIES 26

3.a Description *26*

3.b Transfusion Strategies *26*

3.c Rationale for Transfusion Thresholds *26*

3.d Method for Assigning Subjects to Treatment Groups *27*

3.e Preparation and Administration of Red Blood Cell Transfusions *27*

3.f Subject Compliance Monitoring *28*

3.g Prior and Concomitant Therapy *29*

3.h Blinding of Study Intervention *29*

E STUDY PROCEDURES 29

E1 SCREENING FOR ELIGIBILITY 29

E2 SCHEDULE OF MEASUREMENTS 29

E3 IN-HOSPITAL FOLLOW-UP 30

E4 FOLLOW-UP AT 30 DAYS 30

E5 FOLLOW-UP AT 6 MONTHS 31

E6 SAFETY AND ADVERSE EVENTS 31

6.a Safety and Compliance Monitoring ... *31*

6.b Medical Monitoring ... *31*

i Investigator Only ... 31

ii Independent Expert to Monitor ... 32

iii Independent Data and Safety Monitoring Board ... 32

6.c Definitions of Adverse Events ... *33*

6.d Classification of Events ... *34*

i Severity ... 34

ii Relationship ... 34

iii Expectedness ... 35

6.e Data Collection Procedures for Adverse Events and Unanticipated Problems ... *35*

6.f Reporting Procedures ... *36*

6.g Adverse Event Reporting Period ... *37*

6.h Post-study Adverse Event ... *37*

E7 STUDY OUTCOME MEASUREMENTS AND ASCERTAINMENT ... 37

7.a Definitions of Outcomes ... *37*

i Myocardial Reinfarction ... 37

ii Death and Cause of Death ... 38

iii Unscheduled Coronary Revascularization (unstaged) ... 38

iv Readmission to Hospital, overall and for primary cardiac diagnosis ... 38

v Unstable Angina ... 38

vi Congestive Heart Failure ... 38

vii TIA or Stroke ... 39

viii Deep Venous Thrombosis or Pulmonary Embolism ... 39

ix Bleeding ... 39

x Infections ... 39

xi Length of Stay and Intensive Care Unit Days ... 40

xii Quality of Life ... 40

7.b Ascertainment ... *40*

E8 BASELINE AND INTERVENTION CLINICAL CHARACTERISTICS ... 41

F STATISTICAL PLAN ... 41

F1 SAMPLE SIZE DETERMINATION AND POWER ... 41

F2 INTERIM MONITORING AND EARLY STOPPING ... 42

F3 ANALYSIS PLAN ... 46

F4 STATISTICAL METHODS ... 46

4.a Baseline Characteristics of the Enrolled Patient Sample ... *46*

4.b Adherence to Assigned Intervention ... *47*

4.c Primary Outcome Analysis ... *47*

4.d Analysis of Secondary and Tertiary Outcomes ... *47*

4.e Missing Outcome Data ... *48*

4.f Subgroup Analyses ... *49*

4.g Combining Vanguard and Main Trial Data ... *49*

F5 UNBLINDING PROCEDURES ... 50

G DATA HANDLING AND RECORD KEEPING ... 50

G1 CONFIDENTIALITY AND SECURITY 50

G2 TRAINING 51

G3 CASE REPORT FORMS AND SOURCE DOCUMENTS 51

G4 RECORDS RETENTION 52

G5 PERFORMANCE MONITORING 52

H STUDY MONITORING, AUDITING, AND INSPECTING 52

H1 STUDY MONITORING PLAN 52

H2 AUDITING AND INSPECTING 52

I STUDY ADMINISTRATION 53

I1 ORGANIZATION AND PARTICIPATING CENTERS 53

1.a Clinical Coordinating Center 53

1.b Data Coordinating Center 53

1.c Clinical Consortium 54

I2 FUNDING SOURCE AND CONFLICTS OF INTEREST 54

I3 COMMITTEES 54

3.a Steering Committee 54

3.b Operations Committee 55

3.c Executive Committee 55

3.d Clinical Events Committee 55

I4 SUBJECT STIPENDS OR PAYMENTS 55

I5 STUDY TIMETABLE 56

J PUBLICATION PLAN 57

K REFERENCES **58**

L APPENDICES **66**

L1 APPENDIX 1: STANDARDIZED APPROACH AND SCRIPT TO BE USED TO SEEK PERMISSION FROM ATTENDING PHYSICIAN TO RECRUIT A PATIENT 67

L2 APPENDIX 2: MINT DATA ELEMENTS BY STUDY TIME POINT 68

L3 APPENDIX 3: SAE AND UP EVENT REPORTING TIMELINES 69

L4 APPENDIX 4: ACCRUAL TIMELINE MILESTONES 70

A Introduction

A1 Statement of Compliance

The trial will be conducted in accordance with the ICH E6, the Code of Federal Regulations on the Protection of Human Subjects (45 CFR Part 46), and the NIH Terms of Award. The Principal Investigator will assure that no deviation from, or changes to the protocol will take place without prior agreement from the sponsor and documented approval from the Institutional Review Board (IRB), except where necessary to eliminate an immediate hazard(s) to the trial participants. All personnel involved in the conduct of this study have completed Human Subjects Protection Training.

I agree to ensure that all staff members involved in the conduct of this study are informed about their obligations in meeting the above commitments.

Principal Investigator: __Jeffrey L Carson, MD______________________________
Print/Type Name

Signed: ____________________________________ Date: ______________
Signature

A2 Study Abstract

Accumulating evidence from clinical trials suggests that a restrictive transfusion strategy is safe in most clinical settings. However, a low oxygen carrying capacity from moderate anemia may be deleterious in patients with cardiac ischemia. The potential for harm associated with anemia in patients with acute symptomatic coronary disease is supported by pathophysiological data that maintaining higher hemoglobin levels could benefit the ischemic heart by increasing oxygen delivery. Systematic reviews of clinical trials evaluating transfusion strategies in patients with known ischemic heart disease document the absence of high quality data, which has resulted in an ongoing controversy. The lack of high quality evidence to guide transfusions in patients with acute myocardial infarction has been cited in several major guidelines as well as by an NIH expert panel.

This multicenter trial, the Myocardial Ischemia and Transfusion (MINT) trial, randomly allocates 3500 patients with acute myocardial infarction and a hemoglobin concentration less than 10 g/dL to be treated either according to a liberal or restrictive blood transfusion strategy. Patients assigned to the liberal transfusion strategy receive one unit of packed red blood cells following randomization and enough blood to raise the hemoglobin concentration to 10 g/dL or above any time a concentration less than 10 g/dL is detected. Patients assigned to the restrictive transfusion strategy are permitted to receive a transfusion if the hemoglobin concentration falls below 8 g/dL or if angina symptoms clearly related to the anemia occur and are not controlled with anti-anginal medications. Only enough blood is given to reach a hemoglobin concentration of 8 g/dL or relieve the symptoms. Transfusion is strongly recommended if the hemoglobin concentration falls below 7 g/dL.

The transfusion protocol is followed during the index hospitalization (up to 30 days). Each patient is contacted at 30 days for a comprehensive follow-up for assessment of several relevant clinical outcomes. Patients are contacted again at 180 days to ascertain vital status for assessment of six-month mortality.

A3 Primary Hypothesis

The primary hypothesis is that among patients with an acute myocardial infarction and a hemoglobin concentration less than 10 g/dL, a liberal transfusion strategy with a threshold of 10 g/dL reduces the rate of the composite outcome of all-cause mortality or recurrent nonfatal acute myocardial infarction through 30 days following randomization compared to a restrictive transfusion strategy with a threshold of 7 to 8 g/dL.

A4 Purpose of the Study Protocol

The purpose of the trial is to assess red blood cell transfusion strategies that are currently used in clinical practice and important medical events. Red blood cells are a limited and expensive medical therapy. Physicians frequently transfuse patients to maintain specific (and often differing) hemoglobin levels, despite the lack of evidence supporting the strategy. The study results, which will determine the benefit (or risk) of a liberal transfusion strategy, will influence the allocation of red blood cells worldwide.

A5 Schematic of Study Design

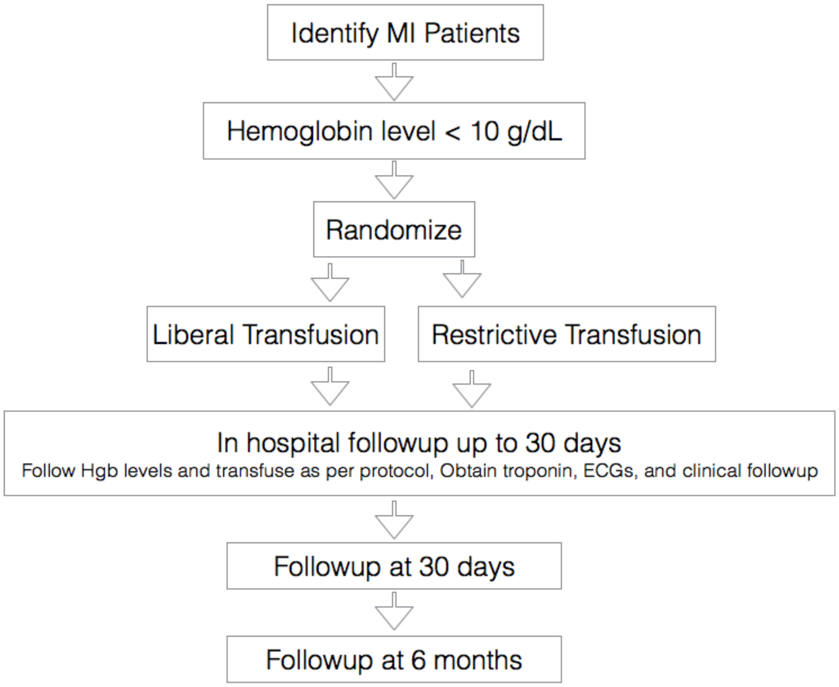

A6 Key Roles

Principal Investigator, Clinical Coordinating Center and Study Chair

Jeffrey L Carson, MD
Provost, Rutgers Biomedical Health Sciences
Rutgers Robert Wood Johnson Medical School
125 Paterson Street
New Brunswick, New Jersey 08901
Telephone: 732-235-7122
FAX: 732-235-7144
Email:jeffrey.carson@rutgers.edu

Principal Investigator, Data Coordinating Center

Maria Mori Brooks, PhD
Professor of Epidemiology
University of Pittsburgh
Graduate School of Public Health
A530 Crabtree Hall / 130 DeSoto Street
Pittsburgh, PA 15261
Telephone: (412) 624-1618
FAX: (412) 383-5891
Email: mbrooks@pitt.edu

Project Scientist

Simone Glynn MD, MSc, MPH
Branch Chief
Blood Epidemiology and Clinical Therapeutics Branch
Division of Blood Diseases and Resources
National Heart, Lung, and Blood Institute
Tel: 301-435-0065
Fax: 301-480-0867
Email: glynnsa@nhlbi.nih.gov

Project Officer

Pablo Cure, MD, MPH
Program Officer
Blood Epidemiology and Clinical Therapeutics Branch
Division of Blood Diseases and Resources
National Heart, Lung, and Blood Institute, NIH
6701 Rockledge Drive, Room 9163
Bethesda, MD 20892-7950
Tel: +1-301-451-7431Email: pablo.cure@nih.gov

B Background

B1 Prior Literature and Studies

Blood transfusion is a common medical intervention. In the United States, more than 16 million red blood cell units are transfused annually to 3.4 million patients.(1) Worldwide 108 million units of blood are collected per year.(2) Of all transfusions, approximately 25% of all red cells transfused are given to patients with a primary diagnosis of cardiac disease(3) and 8% of all cardiology admissions are transfused with RBCs.(4) The economic ramifications of this frequent intervention are significant. The latest estimates of the cost of a red blood cell unit range from $522 to $1183 (mean, $761±$294).(5) The safety of transfusion with respect to transmission of infectious agents has increased greatly throughout the past two decades, and complications are rare.(6, 7)

Anemia and Cardiac Disease: Generating evidence to guide transfusion threshold decisions in patients with myocardial infarction is especially important because coronary artery disease is so common,(8) anemia is present frequently in this setting, and is associated with increased mortality. A study of 44,242 patients with non-ST segment elevation myocardial infarction from 400 US hospitals found 22.2% of patients had a hematocrit <30% (Hgb ≤10 g/dL).(9) In-hospital mortality was 10.4% in patients with Hgb <10 g/dL compared to 2.7% in patients with Hgb >10 g/dL.(10) In a second study involving 17,676 patients with acute myocardial infarction, hospital-acquired anemia (Hgb <11 g/dL) developed in 20.1% of patients during the hospitalization. Patients with Hgb <9 g/dL had a strong association with mortality (odds ratio=3.39).(10) The mechanism of early mortality in patients with acute cardiac injury and anemia may be related to a low ischemic threshold leading to myocardial injury, congestive heart failure and ventricular arrhythmia.

Accumulating evidence from clinical trials suggests that a restrictive transfusion strategy is safe in most clinical settings(11-14) with the possible exception of patients with acute coronary syndrome.

Most reviews and guidelines define a restrictive transfusion strategy as the administration of red cells once hemoglobin falls below either 7 or 8 g/dL while a liberal strategy is most often suggested as a transfusion trigger of 10 g/dL.

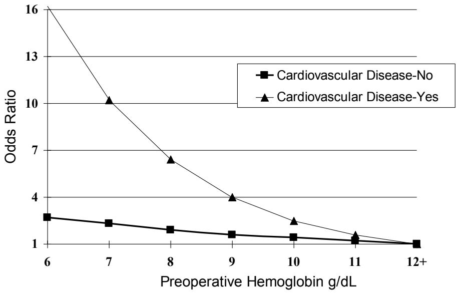

Pathophysiology: The AABB (formerly called the American Association of Blood Banks) guidelines on Red Blood Cell Transfusion recently recommended the use of restrictive transfusion triggers in most patients with the exception of those with an acute coronary syndrome.(15, 16) Their rationale – <u>in the absence of randomized trial evidence</u> – was the large body of basic physiology and observational data would suggest that a restrictive strategy may be deleterious. This is because oxygen delivery to the myocardium is flow dependent since the heart extracts a high percentage of oxygen. Therefore, myocardial ischemia may be precipitated or worsened by low hemoglobin concentrations, especially in patients with coronary stenosis or active plaques. Studies performed in canines suggest a decreased ability to tolerate anemia in the presence of coronary artery disease.(17-19) Electrocardiographic changes consistent with ischemia are seen at hemoglobin concentration below 5 g/dL in normal animals but at 7-10 g/dL with experimentally induced coronary artery disease.

Data from patients who decline blood transfusion for religious reasons were congruent with animal data. In a retrospective cohort study that included 1,958 adult surgical patients who declined transfusion for religious reasons and found a significant interaction between underlying cardiovascular disease and preoperative Hgb level with respect to death (p=0.03).(20) In patients with underlying cardiovascular disease the adjusted odds of postoperative death began to rise sharply at Hgb level ≤10 g/dL while in patients without underlying cardiovascular disease there was a more subtle rate of increasing risk below 10 g/dL (see Figure 1). This study does not address whether transfusion would improve outcome.

Transfusion could be harmful by several possible pathophysiological mechanisms. Transfusion has been shown to increase platelet reactivity and increase procoagulant proteins.(21) Stored red blood cells take time to replenish 2, 3 DPG levels impairing release of oxygen(22, 23) and have low levels of nitrous oxide impairing oxgen delivery and vasodilation.(24) The membranes of stored red blood cells may become deformed and plug microvascular vessels.(25, 26)

Systematic Reviews, Observational Studies: We have identified or conducted systematic reviews of both observational studies and clinical trials. As expected, a systematic review of observational studies in all patients found that blood transfusion was associated with increased mortality and morbidity.(27) Two recent systematic reviews focusing specifically on patients with myocardial infarction identified 10 studies (9 observational and 1 pilot trial) reporting the effects of transfusions on mortality.(28, 29) Nearly all observational studies demonstrated an association between transfusion and higher mortality. The one exception was in a large study using Medicare billing data in 79,000 patients with acute myocardial infarction.(30) Transfusion was associated with a lower risk of death when patients had an admission hematocrit below 0.33 (equal to a hemoglobin concentration of 11 g/dL) (odds ratio= 0.69; 95% CI, 0.53-0.89) and relationship between transfusion and better outcomes increased as the hematocrit fell. Indeed, based on subgroup analyses, the authors of the systematic review report that the adverse effects of transfusion appear to be mitigated in patients with a hematocrit less than 0.33. Unfortunately, observational studies cannot be used to evaluate the effect of red blood cell transfusion since the use of blood transfusion is a marker for illness burden.(29, 31) Thus, no matter how refined the adjustment is for differences in illness burden, it is difficult, if not impossible, to completely adjust for differences between patients receiving and not receiving blood transfusion.

Systematic Reviews, Clinical Trials: We have performed a systematic review of clinical trials evaluating transfusion triggers in a variety of populations that were published in the Cochrane database(32) and JAMA.(33) We have updated the review (34) and found that compared with higher hemoglobin transfusion thresholds (~10 g/dL), a hemoglobin transfusion threshold of 7 or 8 g/dL is associated with fewer red blood cell units transfused (mean difference, -1.22 units per patient), without adverse associations with mortality, cardiac morbidity, functional recovery, or length of hospital stay. The relative risk for the association of restrictive versus liberal transfusion on 30-day all-cause mortality was 0.99 (95% CI, 0.82 to 1.20).

A recent meta-analysis of 11 selected trials enrolling patients with cardiovascular disease (including data obtained from authors from four trials) was recently reported. The risk ratio for the association between transfusion thresholds and 30-day mortality was 1.15 (95% confidence interval 0.88 to 1.50), but the risk of acute coronary syndrome in patients in the restrictive compared with liberal transfusion group was increased in nine trials (risk ratio 1.78, 95% confidence interval 1.18 to 2.70).(35). This analysis is limited by the fact that data are only included from 27% of the eligible trials. The results of

a meta-analysis in cardiac surgery patients reports trend favoring liberal transfusion (36) but the results conflict with another analysis in cardiac surgery patients in which no trend was evident. (34)

Clinical Trials in Acute Coronary Syndrome: Our systematic review identified two pilot clinical trials that included patients suffering an acute coronary syndrome. The first was a small pilot trial including 45 patients with acute myocardial infarction.(37) Patients with hematocrit less than 30% were randomly allocated to a liberal (hematocrit <30%) versus restrictive (hematocrit < 24%) transfusion threshold. The primary clinical safety measurement of in-hospital death, recurrent myocardial infarction, or new or worsening congestive heart failure occurred in 8 patients in the liberal arm and 3 in the restrictive arm (p< 0.046). There were 2 deaths in restrictive group and 1 death in the liberal group. The authors concluded a definitive trial was urgently needed.

In contrast, the MINT pilot enrolled 110 patients and found the pre-defined primary outcome of death, myocardial infarction, or unscheduled revascularization within 30 days occurred in 6 patients (10.9%) in the liberal-transfusion strategy and 14 (25.5%) in the restrictive-transfusion strategy p=0.054). Death at 30 days was less frequent with liberal transfusion 1 (1.8%) compared to restrictive transfusion 7 (13.0%); p=0.032.

Overall, there were 2 deaths in the liberal transfusion strategy and 9 deaths in the restrictive transfusion strategy (relative risk= 3.74, 95% CI 0.80-17.49; p=0.09) when the two trials in acute coronary syndrome are combined.

Other Trials (38) with Signal of Harm from Restrictive Transfusion: The two most recently published trials also found a higher mortality in patients in the restrictive transfusion group in patients with ischemic heart disease. The Titre2 trial contrasted liberal transfusion (9 g/dL) and restrictive transfusion (7.5 g/dL) in postoperative patients undergoing cardiac surgery. The short-term outcomes were comparable between the transfusion strategies, but at 90 days follow-up, overall mortality was higher in the restrictive transfusion strategy than the liberal transfusion strategy (hazard ratio=1.64; 95% confidence interval, 1.00 to 2.67, p=0.045). In a cluster randomized trial in 939 patients with GI bleeding, the mortality was trending higher in subgroup of patients with underlying ischemic heart disease; liberal transfusion strategy was 3% and in the restrictive transfusion strategy was 12% (difference =10.7%; 95% confidence intervals -9.8 to 31.2; interaction p=0.11)

Variation in Transfusion and Guidelines: The systematic reviews of observational studies and randomized trials evaluating the impact of anemia and transfusion highlight the lack of any study with

sufficient numbers of patients to guide clinical care. All of this uncertainty has helped fuel significant practice variation. Two older large studies in 44,242 patients with non-ST segment elevation myocardial infarction from 400 US hospitals(9) and 17,676 patients with acute myocardial infarction demonstrate substantial variation in transfusion.(10) Similar variation was observed in over 19,315 hospitalized patients with a discharge diagnosis of myocardial infarction during 2009-2012 from the California Kaiser Permanente Health System (Percent transfused at nadir hemoglobin level ≥10 g/dL, 0.2%; 9-9.9 g/dL, 12.5%; 8-8.9 g/dL, 48.9%; 7-7.9 g/dL, 78.3%; < 7 g/dL, 91.5%). (39) A significant proportion of patients had transfusion thresholds at every cut-off from 7 to 10 g/dL. An updated analysis demonstrates variation in hospital acquired anemia in 17,676 patients with myocardial infarction (40) and transfusion among 34,937 patients with a myocardial infarction hospitalized between 2000-2008 in 57 centers. (41) In a retrospective cohort study based on the CathPCI Registry data from 2009 to 2013, the risk adjusted rates of transfusion by hospital varied from 0.3% to 9.3%. (42) The high transfusion hospitals used a threshold between 9 to 10 g/dL, and low transfusion hospitals used a threshold between 8 to 9 g/dL. More contemporary data in cardiac surgery patients from 2013 show continued variation in transfusion practice. (43)

The variation in transfusion practice may further be exacerbated by the great variability in the transfusion guideline recommendations. While all conclude that there are too few high-quality studies, recommendations vary widely among organizations. The American Red Cross, AABB, British Committee for Standards in Haematology were not able to recommend a course of action,(44, 45) the American College of Physicians recommends 7-8 g/dL in patients with heart disease but is silent in acute coronary syndrome patients;(46) the American College of Cardiology/American Heart Association suggests avoidance of transfusion unless hemoglobin less than 8 g/dL(47, 48); National Comprehensive Cancer Network recommends 10 g/dL threshold in patients with coronary heart disease including patients with acute coronary syndrome (49), National Blood Authority of Australia recommends 8 g/dL threshold (50), and the European Society of Cardiology recommends transfusion only in case of compromised hemodynamic status and hemoglobin less than 7 g/dL.(51) The recently published guidelines from the AABB concluded that there was insufficient evidence in patients with acute MI and did not provide a specific recommendation, (52) while the UK National Clinical Guidelines Centre recommended transfusion at 8 g/dL (53). Given the lack of high quality evidence to guide transfusion in patients with acute myocardial infarction, it is not surprising that there is variation in recommendations emanating from different organizations.

Despite the variation in guidelines for transfusion in patients with acute myocardial infarction, there has been a strong and consistent trend towards reducing the use of blood transfusion in the US. For example, red blood cell transfusions per 1000 US population fell by 13.9% between 2013 and 2015. (54) Data from California Kaiser Permanente Health System show that in 70,189 hospitalized patients with cardiovascular disease, the frequency of transfusion fell 25% between 2009 and 2013. (55) The pre-transfusion hemoglobin in patients with cardiovascular disease declined from 8.1 to 7.6 g/dL. This change in clinical practice may be due to the widespread implementation of blood management programs (54) and Choosing Wisely Campaigns which targeted “unnecessary” transfusions. (56)

B2 Rationale for this Study: Equipoise

Every day, clinicians encounter anemic patients who have acute ischemic heart disease where a decision to transfuse must be made. However, clinicians do not know what to do because: 1) Observational studies and randomized trials have come to different conclusions and are flawed; 2) Pathophysiological arguments can be made for liberal and restrictive strategies; 3) Some clinical trials in other settings suggest a restrictive transfusion approach is safe but other trials signal the possibility of reduced mortality with liberal transfusion in patients with cardiac disease; 4) Guidelines provide conflicting advice. This has led to practice variation(9, 57) and confusion in the clinical community with no clear guidance on when to transfuse. Thus, the NHLBI State of the Science expert panel concluded in March 2015 “equipoise for transfusion thresholds persists in patients with ischemic heart disease.” (58) The ACC/AHA guidelines for management of STEMI concluded, “the optimal hemoglobin level in the transfused patient is not known.”(47, 48) Furthermore, the recently published guidelines by the AABB identified acute coronary syndrome as clinical setting where the evidence is “judged to be insufficient” to recommend transfusion threshold.(16) Based on these findings and existing data there clearly is equipoise. For all these reasons, a high quality randomized trial to guide transfusion is urgently needed to answer this clinically relevant question.

C Study Objectives

C1 Primary Aim

The primary aim is to determine whether a liberal transfusion strategy with a threshold of 10 g/dL reduces the composite outcome of all-cause mortality or nonfatal myocardial reinfarction through 30 days following randomization, compared to a restrictive transfusion strategy with a threshold of 7 to 8

g/dL among patients with an acute myocardial infarction and a hemoglobin concentration less than 10 g/dL.

C2 Secondary Aims

1) To determine whether a liberal (10g/dL) transfusion strategy reduces all-cause mortality within 30 days, compared to a restrictive transfusion strategy.

2) To determine whether a liberal (10g/dL) transfusion strategy reduces myocardial reinfarction within 30 days, compared to a restrictive transfusion strategy.

3) To determine whether a liberal (10g/dL) transfusion strategy reduces the composite outcome of all-cause mortality, nonfatal myocardial reinfarction, ischemia driven unscheduled coronary revascularization (percutaneous coronary intervention or coronary artery bypass grafting), or readmission to the hospital for ischemic cardiac diagnosis within 30 days, compared to a restrictive transfusion strategy.

C3 Tertiary Aims

1) To determine whether a liberal (10g/dL) transfusion strategy reduces all-cause mortality, nonfatal myocardial reinfarction, or unstable angina (i.e. acute coronary syndrome) within 30 days, compared to a restrictive transfusion strategy.

2) To determine whether a liberal (10g/dL) transfusion strategy reduces ischemia driven unscheduled coronary revascularization within 30-days compared to a restrictive strategy.

3) To determine whether a liberal (10g/dL) transfusion strategy reduces unscheduled readmission to hospital for ischemic cardiac diagnosis within 30 days, compared to a restrictive strategy.

4) To determine whether a liberal (10g/dL) transfusion strategy increases congestive heart failure within 30 days, compared to a restrictive transfusion strategy.

5) To determine whether a liberal (10g/dL) transfusion strategy reduces unscheduled readmission to hospital for any reason within 30 days, compared to a restrictive strategy.

6) To determine whether a liberal (10g/dL) transfusion strategy increases each of the individual thrombotic/hemorrhagic outcomes of stroke, pulmonary embolism or deep venous thrombosis, and bleeding within 30 days, compared to a restrictive strategy.

7) To determine whether a liberal (10g/dL) transfusion strategy increases each of the individual infectious outcomes of pneumonia, blood stream, and urinary tract within 30 days, compared to a restrictive strategy.

8) To determine whether a liberal (10g/dL) transfusion strategy reduces each of the individual in-hospital outcomes of length of hospital stay post randomization and number of days in intensive care unit, compared to a restrictive strategy.

9) To determine whether a liberal (10g/dL) transfusion strategy increases patient reported quality of life using the EuroQol questionnaire (EQ-5D) at 30 days compared to a restrictive strategy

10 To determine whether a liberal (10g/dL) transfusion strategy reduces all-cause mortality at 6-months following randomization, compared to a restrictive strategy.

C4 Rationale for the Selection of Outcome Measures

The study outcomes assess the clinically important benefits and harms of transfusion and anemia in vulnerable individuals with compromised myocardium. Blood transfusions may decrease ischemic injury and improve myocardial performance by improving oxygen delivery to the myocardium in high-risk patients. However, transfusions may also harm patients. Blood transfusions acutely increase blood volume in patients who may not adapt rapidly enough resulting in increased rates of pulmonary edema and heart failure. The increased blood volume could also lead to higher risk of bleeding from increased intravascular pressure. Blood transfusion is also associated with immunosuppression and may lead to infection. In addition, laboratory data suggest that transfusions may not enhance oxygen delivery and may be associated with increased platelet aggregation.

If transfusion to maintain the hemoglobin concentration >10 g/dL does mitigate the clinical consequences of anemia in ischemic cardiac injury, there will be a reduction of mortality and reinfarction. Higher hemoglobin concentrations might also reduce other sequelae of decreased oxygen delivery to the myocardium; unscheduled coronary revascularization, hospital readmission for ischemic symptoms, unstable angina, and cardiovascular mortality.

On the other hand, if transfusion results in clinically important fluid overload, immunosuppression, increased viscosity, and inflammation there will be an increase in congestive heart failure, infection, bleeding, stroke, and pulmonary embolism or deep venous thrombosis. Each of these may contribute to death or reinfarction.

Higher hemoglobin levels may also be associated with a more positive feeling of well-being and a better perceived quality of life as measured by the EQ-5D.

Outcomes are assessed at 30 days since blood transfusion will have its maximum effect within this time period. Mortality will also be assessed at 6 months to determine if early effects of blood transfusion persist.

D Study Design

D1 Overview or Design Summary

This is a randomized, unblinded, two group multicenter clinical trial. Eligible study patients are randomized to receive either the liberal or the restrictive transfusion strategy. Transfusion strategy assignment is not blinded. The transfusion protocol is followed during the Index hospitalization (for up to 30 days). Each patient will be contacted at 30 days to ascertain study outcomes and at 6 months when vital status will be verified. The 30-day and 6-month follow-ups will be administered by telephone, but the follow-up questions could be administered in person, for example, if the patient is in the hospital during the follow-up window. During the 30-day follow-up, readmissions to the hospital that have occurred within the 30 days are identified and medical records will be obtained. The Clinical Events Committee, masked to treatment allocation, will adjudicate occurrences of myocardial infarction within the 30-day window.

Each of the transfusion strategies in this trial is routinely used in current medical practice. Study patients will be followed while they are in the hospital (for up to 30 days) during which time hemoglobin levels, cardiac biomarker of necrosis levels, electrocardiograms, and number of units of red blood cell transfusions administered will be collected.

The transfusion strategies will be compared for differences in mortality, cardiac events and other important morbidity, hospital re-admissions, and patient perceived quality of life.

The goal of this study is to determine whether a liberal transfusion strategy is superior to a restrictive strategy in anemic patients with acute myocardial infarction.

D2 Subject Selection and Withdrawal

2.a Inclusion Criteria

The eligible study population includes patients who meet all of the following criteria: 1) 18 years of age or older; 2) with either ST segment elevation myocardial infarction or Non ST segment elevation myocardial infarction consistent with the 3rd Universal Definition of Myocardial Infarction criteria (38) that occurs on admission or during the index hospitalization, 3) with a hemoglobin concentration less than 10 g/dL at the time of random allocation, and 4) the patient's attending physician, with expertise in cardiovascular care, believes that both of the transfusion strategies are consistent with good medical care for the patient as determined by his/her clinical judgement.

To simplify the diagnosis of acute myocardial infarction, we will require a rise in cardiac biomarker values [preferably cardiac troponin (cTn)] with at least one value above the upper reference limit of normal of the hospital. We anticipate (and will confirm) that the hospital upper limit of normal for troponin will be equivalent to or above the 99th percentile upper reference at all hospitals.

In addition to evidence of myocardial necrosis, we require patients to have at least one of the following: (1) symptoms of ischemia; (2) new/presumed new ST segment or T wave (ST-T) changes or new left bundle branch block (LBBB); (3) development of pathological Q waves; (4) imaging evidence of new loss of viable myocardium or new regional wall motion abnormality; and/or (5) identification of an intracoronary thrombus by angiography. (37) We will include patients with Type 1 (i.e., spontaneous MI presumably related to atherosclerotic plaque rupture, ulceration, fissuring, erosion, or dissection with resulting intraluminal thrombus in one or more of the coronary arteries, leading to decreased myocardial blood flow or distal platelet emboli with ensuing myocyte necrosis), Type 2 (i.e., secondary to an ischemic imbalance such that myocardial injury with necrosis occurs due to myocardial oxygen supply and/or demand mismatch),Type 4b (i.e., stent thrombosis at angiography), and Type 4c (i.e., severe in-stent restenosis without evidence of thrombus).

2.b Exclusion Criteria

Patients will be excluded if any of the following criteria are met 1) uncontrolled acute bleeding at the time of randomization defined as the need for uncrossed or non-type specific blood; 2) decline blood transfusion; 3) scheduled for cardiac surgery during the current admission; 4) are receiving only palliative treatment; 5) if known that follow-up will not be possible at 30 days; 6) if previously participated in MINT 7) if currently enrolled in a competing study that interferes with the intervention or follow-up of MINT or enrolled in a competing study that has not been approved by the local IRB; or 8) if the attending physician does not believe the patient is an appropriate candidate for the trial for any reason.

Patients who have had an episode of uncontrolled bleeding may be enrolled later if the hemoglobin remains below 10 g/dL, they are no longer actively bleeding, and they are otherwise still eligible.

2.c Ethical Considerations

Both transfusion strategies assessed in this trial are widely used in clinical practice. There is uncertainty about which strategy is better, and therefore there is clinical equipoise to conduct this study. Furthermore, at the time of recruitment, the investigator will request permission of the patient's doctor to seek the patient's consent and randomly allocate them to one of the transfusion arms.

At the time consent is obtained, the clinical site study staff will also request the names and contact information of two additional individuals who may be contacted in the event study staff are not able to reach the patient directly at the follow-up time points. This will minimize loss to follow-up. This information will be retained at the clinical site and destroyed at the end of the trial.

2.d Randomization Method and Blinding

The Data Coordinating Center (DCC) will prepare the randomization schedules. Allocation of the transfusion intervention strategies will be in a 1:1 ratio. Given the diverse patient mix among participating clinical sites, the randomization will be stratified by clinical site, and a permuted block design with random block sizes will be used to balance treatment assignments within each clinical site.

Study patients and physicians caring for the patient cannot be masked to treatment assignment (i.e., administration of red blood cell transfusion). However, the central classification of the myocardial reinfarction component of the primary outcome is performed masked to assignment.

2.e Subject Recruitment Plans and Consent Process

The study recruits hospitalized patients diagnosed with acute myocardial infarction (Type 1, Type 2, Type 4b, or Type 4c). Prior to study initiation, each cardiologist at the clinical site will be personally contacted by the clinical site principal investigator or coordinator and permission will be sought to recruit patients who are eligible for the study. Patients of physicians who do not wish to participate in the trial will not be approached for recruitment.

Study staff will identify potential study patients, confirm that the physician agrees that the patient can be randomized into the study (see script in Appendix 1) and approach the patient for consent. Surrogate consent, in accordance with local IRB rules, will be sought for each eligible patient who is not able to grant consent. A substantial number of patients eligible for this trial are likely to be critically ill, medicated, and/or cognitively impaired and unable to grant consent. It is essential that these patients be included as physicians routinely face the dilemma of whether or not they should be transfused.

2.f Risks and Benefits

In patients with heart disease, risks and benefits are considerably different than most other patient populations. Oxygen delivery to the myocardium is flow dependent since the heart extracts a high percentage of oxygen and myocardial ischemia may be precipitated or worsened by low hemoglobin concentrations, especially in patients with acute myocardial infarction. Anemia, if untreated may result in increased risks of further myocardial ischemia and injury. Transfusions, on the other hand, may result in increased risks of pulmonary edema and heart failure (Transfusion-Associated Circulatory Overload), from the significant amounts of volume given to patients with impaired ability of the heart to pump (systolic dysfunction) or ability to relax and fill (diastolic dysfunction). Other adverse effects of allogeneic blood transfusion were also a concern: immunologic (transfusion-related acute lung injury [TRALI],(59) and transfusion-related immune modulation [TRIM] possibly leading to increased bacterial infections).(60) Thus, there is clinical equipoise because it is unclear whether the benefits of immediate correction of anemia with transfusion are outweighed by the potential side effects of transfusion.

2.g Early Withdrawal of Subjects from Transfusion Strategy and Trial Procedures

The study participant (i.e. patient) can be withdrawn from the transfusion strategy (liberal or restrictive transfusion allocation) at any time, either at the request of the treating physician or the patient themselves. The clinical site director may also withdraw a patient. Following the withdrawal, all transfusion decisions will be per treating physician. The patient or physician may also request withdrawal from any trial procedure or follow-up.

2.h When and How to Withdraw Subjects

There are few reasons why a patient or physician might want to withdraw from the trial. If the patient has an adverse effect from a prior transfusion or proves to be difficult to cross match, the physician or patient may choose to withdraw from the study. However, the most common reasons are likely to be patient’s preference not to participate in research or desire not to have extra blood tests or be contacted for follow-up. If a concern is raised and a request for withdrawal is made by the patient or the physician, study staff will confirm the issue (e.g., transfusion strategy, study required measurements and/or telephone follow-up) and try to address the concern. Study staff will be required to contact the MINT CCC PI to discuss the individual situation. If appropriate, the patient will be given the opportunity to refrain from the objectionable study procedure(s) and remain in the overall study. All efforts will be made to avoid a participant feeling unduly pressured to remain in the study. If no intermediate solution is acceptable to the patient and/or treating physician, the patient will be withdrawn from the study. The study staff will document the date of withdrawal, any known reasons for withdrawal and whether the patient will continue with any of the study procedures and/or follow-up. All treating physicians will be immediately notified of a withdrawal from the transfusion strategy (liberal or restrictive transfusion allocation).

2.i Data Collection and Follow-up for Withdrawn Subjects

At the time of withdrawal, the study staff will request permission to continue with data collection and follow-up through the 6-month study time window. However, the exact amount of follow-up performed will vary in accordance with the patients’ authorization.

D3 Trial Transfusion Strategies

3.a Description

We are comparing two commonly used approaches to transfusion therapy, both of which can be considered as “standards of care.” For both strategies, blood must be administered one unit at a time followed by a hemoglobin measurement. The transfusion strategy will be followed throughout the index hospitalization up to 30 days, discharge, or death.

3.b Transfusion Strategies

Restrictive Transfusion Strategy: Patients randomized to the restrictive transfusion strategy will be permitted to receive a transfusion if the hemoglobin concentration falls below 8 g/dL and will be strongly recommended to receive transfusion if the hemoglobin concentration is below 7 g/dL. Transfusion is also permitted if angina symptoms (i.e., retrosternal chest discomfort, chest discomfort described as pressure or heaviness) that are thought by the clinician to be related to anemia occur and are not controlled with anti-anginal medications (sublingual nitroglycerin or equivalent therapy). Blood will be administered one unit at a time and enough blood given to increase the hemoglobin concentration above 7 to 8 g/dL or to relieve symptoms of uncontrolled angina.

Liberal Transfusion Strategy: Patients randomly allocated to the liberal transfusion strategy will receive one unit of packed red cells following randomization and will receive enough blood to raise the hemoglobin concentration to 10 g/dL or above any time the hemoglobin concentration is detected to be below 10 g/dL. A post transfusion hemoglobin measurement showing a hemoglobin level of at least 10 g/dL must be obtained.

A patient in either group may be transfused at any time without a hemoglobin level if the patient is actively bleeding (e.g., brisk gastrointestinal bleeding) and the physician believes an emergency transfusion is needed. A patient in either group with history of congestive heart failure or low ejection fraction may receive diuretics prior to or after transfusion.

3.c Rationale for Transfusion Thresholds

The restrictive transfusion group reflects many current guidelines (including European guidelines)(51, 61). A blood transfusion will be permitted in the restrictive group if the hemoglobin concentration is less than 8 g/dL. Discussions with many cardiologists suggested that many clinicians are not

comfortable with a threshold as low as 7 g/dL (as used in TRICC); however, individual clinicians may choose to use a threshold of less than 7 g/dL to trigger transfusions. Patients may be transfused for signs or symptoms when the clinician believes it is necessary although this did not occur in the pilot trial and was uncommon in the FOCUS trial which also incorporated symptoms in the restrictive transfusion protocol.

In the liberal transfusion group, a threshold of less than 10 g/dL was chosen because oxygen delivery to the myocardium is flow dependent and myocardial ischemia may be precipitated or worsened by low hemoglobin concentrations in patients with coronary stenosis or active plaques. Studies performed in canines found electrocardiographic changes consistent with ischemia as high as 10 g/dL with experimentally induced coronary artery disease. Data from patients who decline blood transfusion for religious reasons were congruent with animal data and found the odds of death rose as the hemoglobin fell below 10 g/dL.

3.d Method for Assigning Subjects to Treatment Groups

Clinical site staff will obtain the randomly assigned transfusion strategy for each eligible consented patient using the MINT website. The randomization system will be available via a secure area of the MINT project website with access restricted to those clinical site personnel with permission to randomize patients. Those who are certified to use the randomization system will be trained to adhere to a strict randomization protocol. In particular, the clinical site personnel must confirm the patient's eligibility status in the system before a transfusion strategy assignment is provided. If the web-based randomization system is not accessible, clinical site personnel will be instructed to follow back-up procedures in order to ensure that the clinical centers are able to randomize patient 24/7.

3.e Preparation and Administration of Red Blood Cell Transfusions

All red blood cell units are maintained and ordered through the hospital blood bank. The storage solution and storage time will be at the discretion of the blood bank, but only leukoreduced red blood cell transfusion will be used. The transfusions are administered by hospital staff in accordance with hospital policy. Study staff will alert the nursing and medical staff to the assigned transfusion strategy each time a new patient has been enrolled in the study. Treating staff will order (or not order) red cell transfusions in accordance with the protocol.

3.f Subject Compliance Monitoring

There are two primary site based mechanisms to ensure adherence to the assigned transfusion strategy 1) study staff review of the medical record and, if necessary, direct discussion with the treating physicians and, 2) direct assistance from the blood bank.

The clinical site is to obtain a daily hemoglobin level for each randomized patient for the first three days following randomization (or through hospital discharge, if sooner). Additional hemoglobin levels are measured as clinically indicated. Study staff will closely monitor each patient to ensure that each of the required hemoglobin levels are drawn, review the results, and confirm that the transfusions have been ordered (or not ordered) in accordance with the transfusion assignment. If there is a hemoglobin value that should trigger a transfusion and none is ordered or, as the alternative, a transfusion ordered without a hemoglobin value to trigger the transfusion, study staff will discuss the case with the treating physician. Likewise, if a required hemoglobin level has not been ordered, the study staff will alert the physician. The required hemoglobin measurements are of primary importance to verify that patients in the liberal strategy maintain a level of at least 10 g/dL.

The clinical site will also request assistance from the blood bank to help prevent protocol violations in the restrictive strategy. Study staff will notify the blood bank each time a patient is assigned to the restrictive strategy and request notification prior to the release of any red blood cell transfusions that are ordered. The study staff will then review the medical record to verify that the transfusion is in accordance with the protocol. If administration of blood is a violation of the protocol, study staff will contact the ordering physician to discuss transfusion plans and to clarify the study protocol. However, study staff do not approve or disapprove the transfusion. The final decision on the transfusion is always the treating physician's.

Study wide, the DCC will centrally monitor transfusions and hemoglobin levels on an ongoing basis. Transfusion rates for each strategy will be measured overall and by clinical site. Specifically, the DCC will identify instances when 1) patients randomized to the liberal strategy do not receive a transfusion, 2) patients randomized to the liberal strategy are discharged with a hemoglobin level < 10 g/dL and, 3) patients randomized to the restrictive strategy without anginal symptoms and a hemoglobin level ≥ 8 g/dL receive a transfusion. Reports of the protocol violations will be prepared by the DCC. The Clinical Coordinating Center (CCC) will review these reports and discuss as necessary with the clinical sites.

3.g Prior and Concomitant Therapy

Transfusion prior to randomization is at the discretion of the clinicians. An otherwise eligible patient may be randomized as long as the hemoglobin level is <10g/dL, regardless of prior transfusions at the time of randomization. Once the patient has been randomized all red cell transfusions are administered per the study protocol. The protocol will not control the administration of other blood products including platelets or fresh frozen plasma. Similarly, it will not mandate specific medical or procedural treatments to manage these patients.

3.h Blinding of Study Intervention

Due to the nature of the interventions, study patients and physicians caring for the patients cannot be blinded to transfusion assignment. However, transfusion strategy assignment will be concealed during the central classification of the myocardial reinfarction component of the primary outcome.

E Study Procedures

E1 Screening for Eligibility

Several approaches to screening for study patients will be used. Study staff will review the results of all troponin levels daily. Medical records for patients with values above the upper reference limit of normal are reviewed and assessed for eligibility. Study staff will screen all admissions to the cardiac care unit, and scheduled for cardiac catheterization. The physician of each eligible patient with a hemoglobin level <10 g/dL will be contacted to confirm that the patient can be entered into the trial using a standardized script (Appendix 1), and consent will be sought. Study staff will follow otherwise eligible patients with hemoglobin levels ≥ 10 g/dL and consent the patient if the hemoglobin level drops to the entry level.

E2 Schedule of Measurements

Each randomized patient is required to have hemoglobin levels, troponin levels, and electrocardiogram readings at specified time points (while in the hospital). The blood samples will normally be drawn with the daily morning blood phlebotomy.

The required time points for hemoglobin levels are: 1) within 24 hours prior to randomization (eligibility hemoglobin level), 2) day 1 post randomization, 3) day 2 post randomization, 4) day 3 post randomization.

The required time points for the troponin levels are: 1) within 24 hours prior to randomization, 2) 12 hours following randomization, 3) day 1 post randomization, 4) day 2 post randomization, 5) day 3 post randomization.

The required time points for the ECGs are: 1) within 24 hours prior to randomization, 2) day 1 post randomization, 3) day 2 post randomization, 4) day 3 post randomization.

A listing of the types of data elements that will be collected at each time point is presented in Appendix 2.

E3 In-hospital Follow-up

Study staff will review the medical records and follow participants during the hospitalization, for up to 30 days post randomization. They will carefully follow and document hemoglobin levels and transfusion status to assure that the transfusion protocol is being followed. In addition, during the first 3 days following randomization they will confirm that each of the required cardiac biomarkers for necrosis, hemoglobin levels, and electrocardiograms has been ordered and the results are recorded.

At 30 days post randomization, or hospital discharge if sooner, the study staff will complete and submit all study data related to the hospitalization including baseline health status, laboratory results, post randomization events and copies of all electrocardiograms performed.

E4 Follow-up at 30 Days

Study staff at the clinical sites will contact each patient at 30 days following randomization. In general, the 30-day follow-up will be administered by telephone, but the follow-up questions could be administered in person. The follow-up interview will include questions about the patient's health and quality of life during the follow-up interval and will determine if there have been any re-admissions to a hospital within 30 days of randomization. Local site staff will obtain the hospital records for each re-admission.

There will be a 30-day window (from 30 days to 60 days following randomization) for study staff to obtain the 30-day follow-up information. In the event they are not able to contact the patient directly, they will try to reach the alternate contacts that have been provided, and a letter will be sent to the patient if needed.

E5 Follow-up at 6 Months

Study staff at the clinical sites will perform a final follow-up contact at 6 months following randomization to determine the patient's vital status. There will be a 30-day window (from 180 days to 210 days following randomization) to obtain the 6-month information. In the event that study staff are not able to contact the patient at the 6-month time point, they will follow the same procedure outlined at the 30-day follow-up.

E6 Safety and Adverse Events

6.a Safety and Compliance Monitoring

The DCC will monitor key aspects of protocol compliance, and reports will be developed and disseminated to monitor the ongoing progress of the study. Protocol safety and compliance reports will include enrollment of ineligible patients, follow-up data collection outside of protocol-defined windows, deviations from the protocol, and adherence to established adverse event reporting and event adjudication procedures. Reports will be provided to the CCC, the Steering Committee, and the DSMB on a systematic basis.

6.b Medical Monitoring

The DCC is responsible for tracking the Serious Adverse Events (SAEs) reported by clinical sites in the MINT trial. The DCC will track the time for each step of the process to ensure that turnaround times adhere to acceptable reporting practices specified by the FDA and NIH. The current reporting guidelines from NHLBI (https://www.nhlbi.nih.gov/research/funding/human-subjects/adverse-event) will be followed, and the timelines are included in Appendix 3.

i Investigator Only

The clinical site will submit a SAE form to the DCC for unexpected serious adverse events occurring within 30 days of randomization and for unexpected deaths occurring within 6 months of

randomization. The clinical sites are responsible for notifying their local Institutional Review Board. Expected SAEs will be reported on the standard MINT in-hospital and follow-up data collection forms.

ii Independent Expert to Monitor

The DCC will notify the MINT Medical Safety Officer of unexpected SAEs submitted by a clinical site and make the relevant information available to the officer. The Medical Safety Officer will determine a) the severity of the event, b) the unexpectedness, and c) the relatedness to the study protocol. In accordance with safety reporting regulations, all SAEs that are determined by the Medical Safety Officer to be both unexpected and possibly, probably or definitely related to the study protocol will be subject to expedited reporting to the Data and Safety Monitoring Board (DSMB), NIH, and any other oversight entities as appropriate. The NIH Project Officer and the DSMB chairperson will review these documents and may decide to convene the DSMB to discuss issues related to monitoring such events.

iii Independent Data and Safety Monitoring Board

An external Data and Safety Monitoring Board (DSMB) consisting of members appointed by the National Heart, Lung, and Blood Institute (NHLBI) will monitor the study, advise the NIH Program Office and provide input to the Steering Committee. The MINT DSMB will include experts in cardiology, transfusion medicine, biostatistics and ethics. The DSMB will review the study protocol and provide NHLBI with recommendations including whether the protocol and patient recruitment can be initiated. NHBLI will approve the study protocol based on the DSMB recommendations before patient recruitment is initiated. Throughout the course of the trial, the Data and Safety Monitoring Board (DSMB) will review recruitment, retention, data completeness, and protocol deviations on a semi-annual basis and will provide recommendations to NHLBI.

Adverse events will be monitored in four distinct ways: 1) the DSMB will review all reported adverse events and monitor the incidence rates of adverse events on a semi-annual basis, 2) the expedited review of unexpected Serious Adverse Events (SAE) that are related to the MINT protocol and unanticipated problems (UP), 3) all study outcomes will be evaluated by assigned treatment group on semi-annual basis, and 4) a formal statistical interim monitoring of the efficacy of the primary outcome by assigned treatment group will be performed on an annual basis. If unexpected safety concerns arise from the trial data or from external research or literature, safety data will be examined on an ad hoc basis. The DCC will work with the NIH and with the DSMB to make certain that the board

members have sufficient information to comprehensively monitor patient safety throughout the MINT trial. The DSMB may advise early termination of the trial for safety reasons or make other recommendations regarding modifications to the protocol.

In order to ensure that the MINT trial has adequate power to test its primary aims, the DSMB will review an interim sample size/power analysis after half of the patients complete the 30-day follow-up. Revised sample size estimates will be calculated based on the observed overall event rate (i.e. the two transfusion groups combined) and the proposed power and effect size.

6.c Definitions of Adverse Events

An adverse event is defined as an untoward or unfavorable medical occurrence in a human participant, including any abnormal sign (for example, abnormal physical exam or laboratory finding), symptom**,** or disease, temporally associated with a person's participation in the research, whether or not considered related to a person's participation in the research. A **Serious Adverse Event** (SAE) is defined as an adverse event that meets **any** of the following criteria:

- results in death;
- is life-threatening i.e. places a participant at immediate risk of death from the event as it occurred;
- requires inpatient hospitalization or prolongation of existing hospitalization;
- results in a persistent or significant disability/incapacity;
- results in a congenital anomaly/birth defect; OR
- any other adverse event that, based upon appropriate medical judgment, may jeopardize the participant's health and may require medical or surgical intervention to prevent one of the other outcomes listed in this definition.

An **Unanticipated Problem (UP)** is defined as any incident, experience, or outcome that meets **all** of the following criteria:

- Unexpected in terms of nature, severity, or frequency, taking protocol research procedures and participation population characteristics into consideration.
- Related or possibly related to a person's participation in the research.
- Places participants or others at a greater risk of harm (including physical, psychological, economic, or social harm) than was previously known or recognized.

6.d Classification of Events

i Severity

The **severity** of the adverse event refers to the intensity of an event and is categorized as 1) Mild; asymptomatic or mild symptoms; clinical or diagnostic observations only; intervention not indicated, 2) Moderate; minimal, local or noninvasive intervention indicated; limiting age-appropriate instrumental activities of daily living, 3) Severe or medically significant but not immediately life-threatening; hospitalization or prolongation of hospitalization indicated; disabling; limiting self-care activities of daily living, 4) Life-threatening consequences; urgent intervention indicated, 5) Death related to AE.

ii Relationship

Relatedness refers to the extent to which an adverse event is considered to be related to the intervention or study procedures. An adverse event is considered **related** if there is a reasonable possibility that the event may have been caused by the procedure. Note that the term "**suspected**" is also means possibly, probably or definitely related to the intervention or study procedures.

The following definitions apply to relatedness:

1. Unrelated
- Adverse event is clearly due to extraneous causes (e.g., underlying disease, environment)

2. Unlikely (adverse event **must meet 2** of the following):
- Does not have temporal relationship to intervention
- Could readily have been produced by the participant's clinical state
- Could have been due to environmental or other interventions
- Does not follow known pattern of response to intervention
- Does not reappear or worsen with reintroduction of intervention

3. Possible (adverse event **must meet 2** of the following):
- Has a reasonable temporal relationship to intervention
- Could not readily have been produced by the participant's clinical state
- Could not readily have been due to environmental or other interventions
- Follows a known pattern of response to intervention

4. Probable (adverse event **must meet 3** of the following):

- Has a reasonable temporal relationship to intervention
- Could not readily have been produced by the participant's clinical state or have been due to environmental or other interventions
- Follows a known pattern of response to intervention
- Disappears or decreases with reduction in dose or cessation of intervention

5. Definite (adverse event **must meet 4** of the following):

- Has a reasonable temporal relationship to intervention
- Could not readily have been produced by the participant's clinical state or have been due to environmental or other interventions
- Follows a known pattern of response to intervention
- Disappears or decreases with reduction in dose or cessation of intervention and recurs with re-exposure

iii Expectedness

An **unexpected** event is one that has not been documented previously as an established adverse reaction to the study intervention and that is not recognized as part of the natural progression of the disease. A particular event may also be considered unexpected if it has a higher severity grade than what has been documented or identified previously.

6.e Data Collection Procedures for Adverse Events and Unanticipated Problems

The clinical site staff will report all serious adverse events and unanticipated problems on the trial data collection forms. These events include those related to red blood cell transfusion including those specified in the informed consent form and those that are related to recent myocardial infarction. All of the expected SAEs are recorded on the in-hospital and the 30-day follow-up data collection forms. Site personnel are required to report and document all unexpected SAEs and unanticipated problems to the DCC. The DCC will send all unexpected SAEs (as reported by the site) to the study Medical Monitor for final assessment of severity, relatedness, and expectedness. The Medical Monitor will remain masked to the transfusion strategy while making his/her evaluation of the SAE.

6.f Reporting Procedures

All reported SAEs and unanticipated problems will be included in systematic reporting to the DSMB on a semi-annual basis. This includes adverse events and problems previously transmitted through expedited reporting.

The following three classes of events need to be reported to the NHLBI, the DSMB and the Local IRB in an expedited manner: 1) Fatal or life threatening unexpected suspected SAE, 2) Non-fatal, non-life threatening unexpected suspected SAE, and 3) Unanticipated problem. Fatalities that are related to blood transfusions must also be reported to the FDA within 7 days according to the guidelines (http://www.fda.gov/BiologicsBloodVaccines/GuidanceComplianceRegulatoryInformation/Guidances/Blood/ucm074947.htm).

The site personnel will compete and submit a SAE form to the DCC within 72 hours of learning of the event whenever the event is both serious and unexpected. In cases where the event is not serious but places the patient at greater risk of physical, psychological, economic, or social harm, and is both unexpected and related to the study, the site PI will fill out an Unanticipated Problem form within 14 days of learning about the problem. SAE forms are forwarded along with relevant patient history data to the Medical Monitor for review. The study Medical Monitor will assess the severity, relatedness, and expectedness of the event within 48 hours. Following the Medical Monitor's feedback on the expectedness and relatedness of the SAE, the DCC will complete an SAE report categorized as serious, unexpected and related. A report will also be sent for unanticipated problems. The DCC will send the reports to the NHLBI DSMB Executive Secretary and the NHLBI Medical Monitor for review. All reporting (from the time that the Site learns about the event until it is reported to the NHLBI, DSMB, FDA and IRBs) will follow the NHLBI DSMB established timelines as specified in (https://www.nhlbi.nih.gov/research/funding/human-subjects/adverse-event) as shown in Appendix 3. If other SAEs are noted to occur with abnormally high frequency during the trial, these will be reported promptly by the MINT DCC to the DSMB, and the NHLBI DSMB Executive Secretary and the NHLBI Medical Monitor. Upon receipt of an expedited report, the DSMB chair will decide whether the event should be discussed at the next scheduled DSMB meeting or discussed as soon as possible at an ad hoc meeting.

IRB actions regarding the MINT trial will be communicated to the NHLBI Project Officer and NHLBI Executive Secretary in an expedited fashion. If the IRB or ethics board at any MINT site, CCC or

DCC takes action regarding the MINT trial (e.g., the IRB places a hold on the trial or suspends the trial), the site will report this to the CCC within 24 hours of the action. This will be communicated by telephone and with an urgent help desk ticket through the MINT trial data management system. The Site will submit written documentation from the IRB, an explanation of the circumstances, and a plan of action to the CCC within 72 hours. The CCC will promptly communicate this information to the DCC and the NHLBI project officer. The DCC is responsible for notifying and the NHLBI Executive Secretary. A written report describing the IRB decision, the rationale for the decision and the plan of action based on this decision will be submitted to the NHLBI project officer and the NHLBI Executive Secretary within 7 days of the IRB action.

6.g Adverse Event Reporting Period

All randomized patients will be followed by the trial for 30-days from randomization, and vital status will be ascertained at 6 months from study enrollment. Serious adverse events that occur during the 30-day time period and all deaths up to 6 months will be reported.

6.h Post-study Adverse Event

MINT will not collect or report information about adverse events that occur more than 30-days after randomization with the exception of death which will be reported through 6 months after randomization. Reporting of Adverse events will cease at the conclusion of the MINT trial.

E7 Study Outcome Measurements and Ascertainment

7.a Definitions of Outcomes

i Myocardial Reinfarction

Myocardial reinfarction will be classified by the Clinical Events Committee using The Joint European Society of Cardiology/American College of Cardiology Committee definitions. (47) Patients with reinfarction will need to demonstrate a fall in the troponin value and then a subsequent rise of at least 20% with additional evidence (new ECG changes, imaging evidence, clinical history) as in the MI definition to diagnose a new event. Myocardial reinfarction will be classified as ST-segment elevation or non-ST Segment elevation.

ii Death and Cause of Death

For each death, the cause will be determined by the site personnel into one of three categories (47): cardiovascular death (e.g., congestive heart failure, dysrhythmia), noncardiovascular death (e.g., infection, cancer), or undetermined cause of death. Information about the specific cause of death will also be collected.

iii Unscheduled Coronary Revascularization (unstaged)

Ischemia driven, unscheduled coronary revascularization (coronary artery bypass surgery or PCI) within 30 days of randomization will be recorded by the sites. Prior to randomization, the site will record if a coronary revascularization is planned (staged). All coronary revascularization procedures will be recorded, but an elective planned staged procedure will not be included as an outcome. Information about the reason for the procedure will also be collected to ensure that the revascularization was done to treat ischemic heart disease.

iv Readmission to Hospital, overall and for primary cardiac diagnosis

All re-admissions to the hospital that had not been planned prior to randomization will be captured, and the primary diagnosis for each hospitalization will be classified as: ischemic cardiac diagnosis (e.g., myocardial infarction, unstable angina), non-ischemic cardiac diagnosis (e.g. heart failure) or non-cardiac.

v Unstable Angina

The MINT trial sites will use 2014 ACC/AHA Key Data Elements and Definitions for Cardiovascular Endpoint Events in Clinical Trials to define unstable angina. (47) To diagnose unstable angina requires that four criteria be met: 1) worsening ischemic discomfort, 2) unscheduled hospitalization, 3) negative cardiac biomarker, 4) objective evidence of myocardial ischemia.

vi Congestive Heart Failure

Sites personnel will use 2014 ACC/AHA Key Data Elements and Definitions for Cardiovascular Endpoint Events in Clinical Trials to define congestive heart failure. (47) New or worsening symptoms of congestive heart failure on presentation (increasing dyspnea, paroxysmal nocturnal dyspnea, orthopnea), has objective evidence of new or worsening heart failure, and receives initiation or intensification of treatment specifically for heart failure.

vii TIA or Stroke

The 2014 ACC/AHA Key Data Elements and Definitions for Cardiovascular Endpoint Events in Clinical Trials will be used to define stroke. (47) A transient ischemic attack (TIA) is defined as “a transient episode of focal neurological dysfunction caused by brain, spinal cord, or retinal ischemia, without acute infarction.” Stroke is defined on the basis of the presence of acute infarction as demonstrated by imaging or based on the persistence of symptoms more than 24 hours.

viii Deep Venous Thrombosis or Pulmonary Embolism

Deep vein thrombosis will be diagnosed if duplex ultrasound, magnetic resonance venogram (MRV), or venogram is definite or probable positive. Site investigators will record if location is proximal or distal. Pulmonary embolism will be diagnosed with a high probability ventilation perfusion lung scan, CT scan, or pulmonary angiogram.

ix Bleeding

Major bleeding will be defined as 1) fatal bleeding, and/or 2) symptomatic bleeding in a critical area or organ, such as intracranial, intraspinal, intraocular, retroperitoneal, intra- articular or pericardial, or intramuscular with compartment syndrome, and/or 3) bleeding causing a drop in hemoglobin concentration of 2 g/dL or greater (62) from the last hemoglobin concentration prior to randomization to the nadir hemoglobin concentration during hospitalization or up to 30 days post randomization. The drop in hemoglobin concentration will account for each unit of red blood cell transfusion transfused by subtracting 1 g/dL for each unit administered.

x Infections

x.1 Pneumonia

Pneumonia will be diagnosed using CDC criteria (48) which includes radiographic abnormalities and combination of symptoms (i.e., cough), signs (i.e., fever, tachypnea, or laboratory abnormalities (i.e., white blood cell count, hypoxemia).

x.2 Blood Stream Infection

Blood stream infection will be defined using CDC criteria which includes a recognized pathogen cultured from 1 or more blood cultures and organism cultured from blood is not related to an infection at another site, at least 1 of the following signs or symptoms: fever, chills, or hypotension and signs

and symptoms and positive laboratory results are not related to an infection at another site and common skin contaminant is cultured from 2 or more blood cultures drawn on separate occasions.

x.3 Urinary Tract Infection

Urinary Tract Infection will be defined using CDC criteria (48) which include one of the following signs or symptoms: fever (>38.0°C) or localized pain or tenderness, and laboratory evidence for infection.

xi Length of Stay and Intensive Care Unit Days

The trial will collect the number of days post randomization that patient is in the hospital and in intensive care unit.

xii Quality of Life

The EuroQol questionnaire (EQ-5D), a standardized instrument that measures health related quality of life in 5 dimensions: mobility, self-care, usual activities, pain/discomfort and anxiety/depression, will be used as a measure of patient perceived global health status 30 days after randomization.

7.b Ascertainment

Relevant data will be collected from the medical record during the index hospitalization. During the first 3 days following randomization (or hospital discharge if sooner) active surveillance is performed to ensure all occurrences of myocardial reinfarction are detected. It is required that the site obtains troponin levels (every 12 hours for 1 day, and then daily for the next 2 days) and electrocardiograms (daily for the first 3 days). These results, in combination with any additional cardiac biomarkers for necrosis and electrocardiograms and medical findings, will be reviewed by the Clinical Events Committee for each randomized patient, irrespective of a site identified event. We will therefore be able to identify myocardial reinfarction events that were not clinically recognized, in addition to those had been.

Study staff will contact the patient at 30 days to ascertain vital status, administer the quality of life questionnaire, and determine if there has been a subsequent hospital admission. Study staff will obtain and review the medical records for each readmission and record all relevant data and identify the study outcomes. If there is a suspect cardiac event the staff will submit de-identified copies of the documentation (from either the index admission or the readmission) for the Clinical Events Committee

adjudication. Study staff will perform a final follow-up contact at 6 months following randomization to ascertain the patient's vital status.

E8 Baseline and Intervention Clinical Characteristics

Baseline demographic (e.g., age, gender, race), co-morbidity (e.g. cardiovascular risk factors), characteristics of cardiac disease (e.g., left ventricular function, number of coronary vessels obstructed), renal disease, and medications (e.g., aspirin, P2Y12 receptor inhibitor, anticoagulant, beta-blocker statins) will be collected to reflect patient status at the time of randomization. Characteristics describing the study intervention including red blood cell transfusion information (e.g. expiration date, leukoreduction, storage solution, ABO) will be recorded. In hospital, hemoglobin levels and the number and timing of transfusions will be recorded for the two assigned transfusion strategies.

F Statistical Plan

F1 Sample Size Determination and Power

In the MINT pilot trial, the composite 30-day rate of death and myocardial infarction was 16.4%. Higher rates were observed in the Kaiser System preliminary data. Thirty-day rates of 16.4%, 18%, or 20% are reasonable for this trial since the proposed study population will not exclude the sickest patients as was done in the MINT pilot study. Using a two-sided inequality test and a simple chi-square statistic with alpha=0.05, we determined the samples sizes required to provide 80% and 90% power to detect varying relative reductions in the 30-day event rates for death and myocardial infarction between the two assigned treatment groups. Based on these estimates, we plan to enroll a sample of 3500 patients. If the rate of missing outcome data is no more than 5%, the trial would have 3324 patients with analyzable outcome data at 30-days. Assuming an overall event rate of 16.4%, the trial will have 80% power to detect a 20% relative reduction (i.e. 18.2% vs. 14.6%) and >90% power to detect a 25% relative reduction. If the overall event rate is 18%, then the trial will have >80% power to detect a 20% relative reduction (i.e. 20% vs. 16%), and if the overall event rate is 20%, the trial will have close to 90% power to detect 20% relative reduction (i.e. 22.2% vs. 17.8%). We also performed

a simulation study to compute the power for a log binomial model with a random intercept for site and a final alpha-level=0.045 to account for the interim monitoring. We simulated 1000 trials where each trial had 60 strata representing the sites, 56 participants within each stratum (i.e. N=3360 patients in total), and a mean intra-class correlation (ICC) of 0.05 to 0.08. The simulations indicate that the power is comparable for the proposed log binomial model and the simple chi-square statistic used for the original sample size calculations. Based on the simulations, the trial will have 81% power to detect a 20% relative reduction from 18.2% to 14.6%, and 90% power to detect a 25% relative reduction from 18.2% to 14.0%. In addition, the trial will have 85% power to detect a 20% relative reduction from 20% to 16%, and 89% power to detect a 20% relative reduction from 22.2% to 17.8%.

This pragmatic randomized clinical trial has excellent power to detect clinically meaningful differences between the two transfusion strategies with respect to the critical 30-day composite outcome of death and myocardial infarction on both the absolute and the relative scales. After half of the patients have been enrolled and followed, we will monitor the overall event rate in the trial to ensure that the trial has adequate power to detect clinically relevant treatment effects.

F2 Interim Monitoring and Early Stopping

The DSMB will review interim analyses of the outcomes by assigned treatment group annually. The interim monitoring is designed to test for evidence of beneficial effect with either treatment strategy while maintaining the overall type I error at the pre-specified level. Stopping rules are based on the information (i.e. number of primary outcome events) that has accumulated by each inspection time and the shape of the predetermined alpha-spending function. We recommend that the Lan-DeMets approach be used to allocate the type 1 error (i.e. alpha-level) at each interim time point and use of O'Brien Fleming monitoring boundaries. The DSMB may advise early termination of the trial for safety reasons or make other recommendations regarding modifications to the protocol.

The MINT trial will have 4 years of recruitment with a goal of enrolling N=3500 patients by June 2021. With this timeline, we propose that formal statistical interim monitoring will occur in August or September of 2018, 2019, and 2020 based on N=298 patients enrolled through June 1, 2018, N=872 patients enrolled through June 1, 2019, and N=1905 patients enrolled through June 1, 2020, respectively. All calculations assume an overall trial alpha of 0.05.

With an alpha-spending method, the alpha level to be used at any given look is based upon the amount of information available at the monitoring time. Once alpha is calculated, boundaries for the

standardized Z-test statistic are constructed. A standardized Z-test statistic is calculated based on the estimated relative risk from the log-binomial model which includes assigned transfusion strategy (according to the intention to treat principle) as the independent variable. If the observed Z-test statistic crosses the boundary the difference is considered statistically significant at that monitoring time. The "α spending function approach" allows the number and frequency of monitoring times to be changed at any point during the trial.

Based on the anticipated accrual shown in Appendix 4, below is the table of the expected percent of information, estimated number of participants, the Z-test statistic boundary level and the corresponding alpha spent based on the O'Brien-Fleming boundaries.

Estimated Boundary Values at Each Interim Monitoring Time Point

			O'Brien Fleming	
**Stage**	**Information Level**		**Boundary Values**	
	**Proportion**	**Number Recruited**	**Bound**	**Alpha Spent**
**1**	0.0900	298	.	.
**2**	0.2500	872	±4.33	<0.001
**3**	0.5400	1905	±2.84	0.005
**4**	1.0000	3500	±1.97	0.045

The O'Brien and Fleming (1979) repeated significance tests combined with the Lan and DeMets (1983) flexible alpha-spending curves are proposed due to their common usage and their conservative nature when the amount of accumulated data in the trial is small. They are designed to stop earlier in the trial if there is overwhelming evidence that there is a difference between the two treatment groups. The ability to detect a difference between the groups becomes easier as the amount of information in the trial increases.

The form of the O'Brien-Fleming alpha –spending function is:

$$\boldsymbol{\alpha(k) = 2 - 2\phi\left(\frac{Z_{\alpha/2}}{\sqrt{i_k}}\right)}$$

where: α(k) = cumulative alpha at inspection k
k = inspection number
ϕ(x) = the cumulative distribution function of the standard normal curve evaluated at x.

Z_α = the value of the standard normal curve where α is the area in the tail
i_k = the amount of information accumulated in the trial at inspection k

We assume a symmetric 2-sided boundary, with each side spending α=0.025. Below are plots of the cumulative O'Brien-Fleming alpha-spending curve and the corresponding boundary plot. We assume a symmetric 2-sided boundary, with each side spending α=0.025.

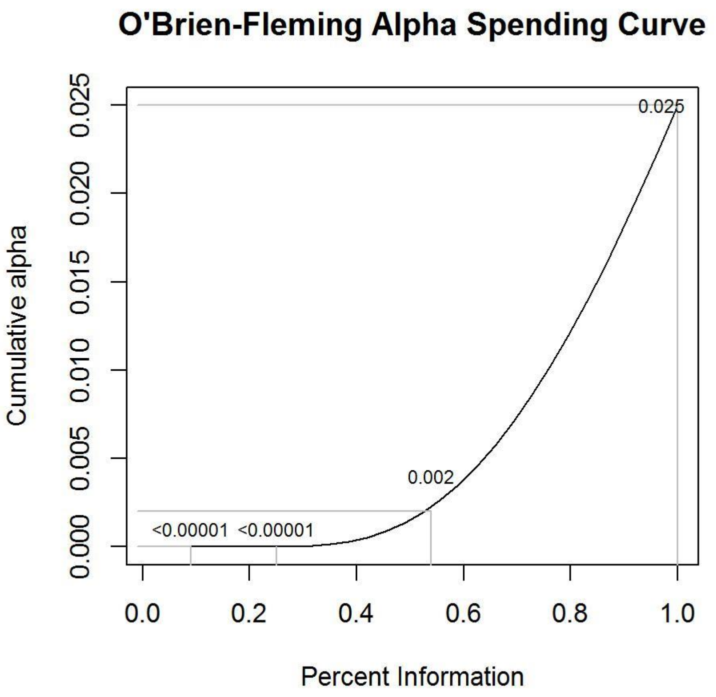

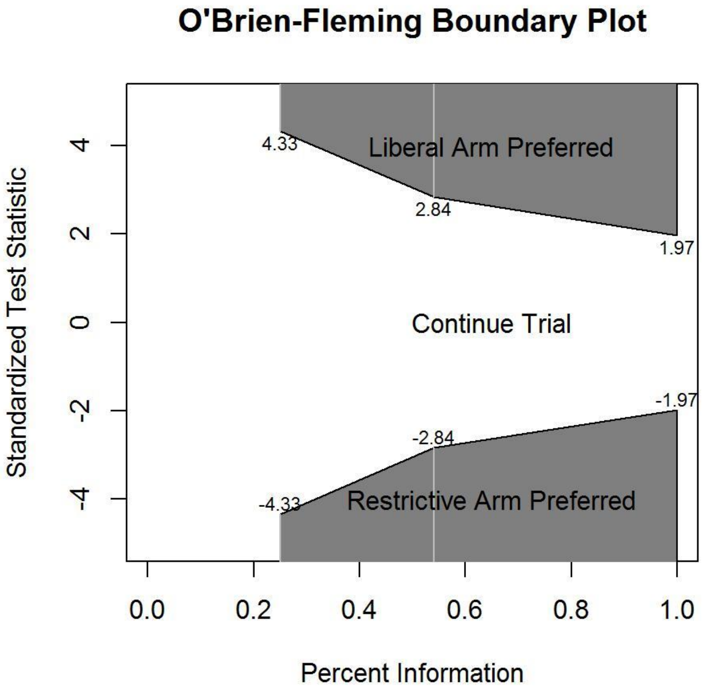

It is possible that the test statistic will cross the monitoring boundary. Statistical interim monitoring results should be taken as one component to the decision as to whether or not to stop a trial. To stop the trial for efficacy, results should be definitive enough to be able to change clinical practice. The DSMB will use the monitoring information to determine its recommendation to NHLBI. The DSMB can recommend that the MINT trial should continue as proposed, that the MINT protocol should be modified based on the results seen in one treatment comparison or in some well-defined subgroup of patients, or that the MINT trial should be terminated early. The final decision to stop trial rests with the NHLBI. If recommendation is to stop the trial, the MINT trial principal investigators shall be consulted before a final decision is made.

In order to ensure that the MINT trial has adequate power, an interim analysis to assess sample size will be conducted after when half of the participants are projected to have completed the 30-day follow-up. Revised sample size estimates will be calculated based on the original power and effect size estimates from the trial hypothesis (80% power to detect a RR=0.80) and on the observed overall event rate (i.e. the two transfusion groups combined). The DSMB will evaluate whether the trial sample size needs to be increased in order to restore power to maintain the ability to detect a clinically meaningful effect size.

Since the MINT trial compares two established transfusion strategies with different resource and cost implications, a null result from a well-powered trial would be important for establishing treatment guidelines and policy. Thus, no interim futility analyses will be conducted in the MINT trial.

F3 Analysis Plan

A Screening Log will be developed to include the number of patients with acute myocardial infarction and hemoglobin level < 10 g/dL, eligibility and enrollment status. Proportions of eligible and enrolled patients will be presented, and reasons why patients are not eligible or enrolled in the trial will be tabulated. Enrollment patterns will be compared across clinical sites and patient groups defined by sex and race/ethnicity. The baseline characteristics and co-interventions of the patients who are randomized in the MINT trial will be described, and characteristics of patients in the two assigned treatment groups will be compared. The proportion of enrolled patients who adhere to the assigned intervention will be ascertained by evaluating the hemoglobin levels and the number of units of blood transfused during the index hospitalization. The intention-to-treat principle will be used to compare the primary outcome (the composite of all-cause mortality and myocardial infarction by 30 days from randomization) and each pre-specified secondary outcome by the randomized transfusion strategy groups. A select number of subgroup analyses will be performed based on baseline factors identified prior to the initiation of the trial.

F4 Statistical Methods

4.a Baseline Characteristics of the Enrolled Patient Sample

Descriptive statistics will be examined for all relevant baseline measures collected on the MINT randomized patients. Transformations of measures will be considered on the basis of distribution diagnostics and outlier analysis. The baseline characteristics and co-interventions of the patients in the overall study will be described using frequencies, proportions, means and standard deviations, or medians and first and third quartiles. Characteristics of patients in the two arms of the trial will be compared using chi-square statistics for categorical variables and t-tests or Wilcoxon rank-sum statistics for continuous variables.

4.b Adherence to Assigned Intervention

The mean hemoglobin concentration at 1, 2, and 3 days post-randomization will be compared between the assigned treatment groups with a Student's t-test. Mixed random effect models will be used to describe the daily mean hemoglobin concentrations during the initial hospitalization following randomization and to compare these repeated measures by assigned treatment group. The mean number of red blood cell transfusions in each randomized treatment group will be described, and a simple Poisson test will be used to test whether the number of units of blood differs significantly by assigned transfusion strategy.

4.c Primary Outcome Analysis

The intention-to-treat principle will be used for all randomized transfusion strategy comparisons of study outcomes. A two-sided test will be used with an alpha-level=0.05 for the primary outcome, the composite of all-cause mortality and myocardial infarction (death/MI) by 30 days from randomization. The 30-day event rate for the primary outcome will be compared by assigned transfusion strategy using an unadjusted log-binomial regression model with a fixed effect variable for assigned treatment strategy and a random effect for clinical site. The primary analysis will involve the estimated relative risk and significance level obtained from the model accounting for interim monitoring and missing data. Event rates, the relative risk and the absolute difference will be from the observed data will also be presented, and 95% confidence intervals will be calculated.

If significant imbalances in baseline risk factors are detected between the two randomized treatment groups ($p<0.05$), multivariable log-binomial regression will be used to adjust for potential confounding factors as a sensitivity analysis. If adherence to the protocol is lower than expected, a per protocol analysis, including only patients who undergo transfusion according to their assignment and adjusting for baseline factors that are associated with adherence to the treatment protocol, will be conducted as a sensitivity analysis.

4.d Analysis of Secondary and Tertiary Outcomes

Event rates, the relative risk and the absolute difference between transfusions strategy groups for the dichotomous secondary and tertiary outcomes will be calculated with their 95% confidence intervals using log-binomial regression methods accounting for clinical site with random effects. The length of hospital stay from randomization and from admission will be analyzed using non-parametric Wilcoxon

rank sum tests, and long-term mortality will be analyzed with Kaplan-Meier methods and the log rank statistic. The Kaplan-Meier estimated mortality rate in each treatment group at 180 days, the absolute difference between the assigned treatment groups at 180 days, and the 95% confidence interval for the difference will be presented. Transfusion strategy comparisons of the EQ-5D quality life measures at 30 days will be based on t-tests or the Wilcoxon rank sum test. No adjustment of the alpha level will be made for the secondary and tertiary analyses. Rather the results will be interpreted based upon the observed findings, their designation as secondary or tertiary outcomes, and in comparison to the primary outcome result.

The secondary outcomes include: 1) all-cause mortality within 30 days; 2) myocardial reinfarction within 30 days; and 3) the composite outcome of all-cause mortality, nonfatal myocardial reinfarction, ischemia driven unscheduled coronary revascularization (percutaneous coronary intervention or coronary artery bypass grafting), or readmission to the hospital for ischemic cardiac diagnosis within 30 days.

The tertiary outcomes include: 1) all-cause mortality, nonfatal myocardial reinfarction, and unstable angina (i.e. acute coronary syndrome) 2) ischemia driven unscheduled coronary revascularization within 30-days; 3) readmission to the hospital for ischemic cardiac diagnosis, 4) congestive heart failure within 30 days; 5) unscheduled readmission to hospital for any reason within 30 days; 6) each of the individual cardiovascular outcomes of stroke, pulmonary embolism or deep venous thrombosis, and bleeding within 30 days; 7) each of the individual infectious outcomes of pneumonia, blood stream, and urinary tract within 30 days; 8) each of the individual in-hospital outcomes of length of hospital stay post randomization and number of days in intensive care unit; 9) patient reported quality of life measures based on the EuroQol questionnaire (EQ-5D) at 30 days; and 10) all-cause mortality at 6-months.

4.e Missing Outcome Data

For the analysis of the primary outcome, standard multiple imputation methods will be used to impute missing 30-day data. We expect that missing 30-day outcome data will be very low in this trial. All patients should have non-missing death and MI data from randomization to hospital discharge. Markov Chain Monte Carlo (MCMC) multiple imputation methods will be used to impute the missing 30-day outcome values (yes/no) for death and MI. Specifically, a log-binomial regression model to estimate the relationship between the outcome and key covariates (observed baseline and

in-hospital variables that are clinically relevant and related to the “missingness” of the 30-day outcomes) in the participants with full data. This model will be used to predict the probability of the outcome for participants who are missing the 30 day outcomes. Based on these probabilities, ten imputed data sets will be created. A log-binomial model with random effects for site will be estimated for each imputed data set, and the results will be pooled to obtain a single estimate of treatment effect with an appropriately adjusted standard error. As a sensitivity analysis, a log-binomial model with random effects for site will be created based on the non-missing 30-day death/MI data. Missing data will not be imputed for the analyses of the secondary and tertiary hypotheses unless critical issues are noted while investigating the missing 30-day data.

4.f Subgroup Analyses

Subgroup analyses will be performed based on baseline factors identified prior to the initiation of the trial. Clinically relevant subgroup variables include: ST segment elevation MI / non ST segment elevation MI, Universal Definition type 1 MI / type 2 MI, type 4b and 4c, baseline hemoglobin level (<8, 8-9, 9-10 g/dL), revascularization for treatment of index MI, bleeding, sex, race/ethnicity (White, Black, Other Race and Hispanic, non-Hispanic), age (<70, ≥70 years old), left ventricular dysfunction, diabetes status, and creatinine clearance (eGFR <30, 30-59, ≥60). The 30-day event rates by assigned transfusion strategy will be compared within each pre-defined subgroup. The reported significance-level will account for the number of treatment comparisons conducted in the study using the false discovery rate (FDR) algorithm developed by Benjamini and Hochberg. In addition, for each subgroup variable, a log-binomial regression model including treatment assignment, subgroup variable and the interaction between the subgroup variable and treatment assignment will be created, and the significance of the interaction term will be used to test whether the randomized treatment effect is significantly modified by the designated subgroup variable. Cox proportional hazards regression models will be created with similar covariates in order to test whether the effect of the randomized transfusion strategy for 6-month mortality varies significantly according to the pre-specified subgroup variables.

4.g Combining Vanguard and Main Trial Data

In 2016, the Canadian Institutes of Health Research (CIHR) funded a MINT Pilot trial. This pilot is coordinated by the University of Montreal, and the clinical sites are in Canada. The DCC and CCC have collaborated with the University of Montreal to ensure that the protocol (eligibility criteria,

interventions, and outcomes) as well as the randomization scheme and the data to be collected are nearly identical to those for the main MINT trial. The CIHR MINT pilot trial was funded to enroll 60 patients. This pilot trial will serve as a “Vanguard phase” for the MINT trial and will allow us to evaluate the feasibility of the MINT protocol. After the MINT protocol is approved by the DSMB/NHLBI and the appropriate ethics committees in Canada, patients randomized at the Canadian Sites will be enrolled as MINT participants. All data collection and data coordination for the MINT participants will occur through the University of Pittsburgh DCC. Patients who are randomized prior to the transition date will remain CIHR pilot trial participants throughout their follow-up. The complete Vanguard datasets housed at the University of Montreal will be transferred to the University of Pittsburgh DCC. The Vanguard patient data will be merged with the MINT trial data based on rigorous comparisons of variable definitions and variable coding. All Vanguard patients will be included in the target sample size and presented in DSMB and published data reports for the MINT trial.

F5 Unblinding Procedures

Patients and providers are not blinded in the MINT trial, and thus unblinding procedures are not required for patient safety. Trial monitors and adjudicators are blinded to transfusion strategy, and the DSMB may choose to be blinded to transfusion strategy as they monitor the outcome data. The Medical Monitor and DSMB can request to be unblinded if they deem this necessary.

G Data Handling and Record Keeping

G1 Confidentiality and Security

Patient identifiers (e.g. name and social security number) will not be sent to the trial DCC, however, some personal health information (e.g. birth date and hospitalization admission date) will be collected centrally. The University of Pittsburgh DCC has a strictly enforced security policy with standard operating procedures addressing key security risk areas. Procedures include password protection, limited access to study computers, encryption, IP restriction, basic or digest authentication, and firewalls between the Internet and the DCC network and servers. Regularly scheduled backups and

archives are performed at least once daily with backup media copies of project files stored off-site in a secure location. Virus detection will be enforced at the DCC. All servers will be connected to uninterruptible power supplies and housed in a computer room with an alarm.

Patient contact information will be maintained exclusively by the local site and stored in a secure location. Only local study staff responsible for the 30 day and 6 month telephone calls will have access to this contact information.

G2 Training

DCC personnel at the University of Pittsburgh will work with CCC personnel at Rutgers / Robert Wood Johnson to provide the training necessary for uniform data collection, protocol compliance, and data processing. Major elements to be covered are inclusion and exclusion criteria, procedures and tools to monitor adherence to protocol, randomization procedures, event adjudication procedures, adverse event reporting, methods for extracting data from different sources, follow-up schedules, what to do about missing information, reporting requirements, and use of the Manual of Operations and study website. Instruction will be provided for entering the data, transmitting them to the DCC, interpreting edit reports and correcting data.

On-going training will be required due to the addition of new clinical sites as well as turnover of staff at the existing clinical sites. The CCC and DCC will utilize webinars and conference calls to conduct detailed distance training sessions. On-line protocol and data collection training modules will be available on the trial website, and focused training sessions will be conducted in conjunction with Steering Committee meetings as required. Throughout the trial, there will be regularly scheduled conference calls with the coordinators and data collectors at all sites to discuss issues that arise with protocol and data collection.

G3 Case Report Forms and Source Documents

The data collection forms for this trial have been developed to facilitate enrollment and follow-up while still collecting the essential information needed to answer the trial hypotheses. A web-based distributed data management system will be used for data entry. Source documents will be maintained at the clinical site.

G4 Records Retention

Trial patient records will be retained at the clinical sites and at the Coordinating Centers for 6 years after MINT data collection is completed.

G5 Performance Monitoring

The DCC will monitor study performance. Routine reports will be developed to monitor the ongoing progress of the study including enrollment and retention. Protocol adherence reports include enrollment of ineligible patients, follow-up data collection outside of protocol-defined windows, and deviations from protocol. The timeliness of data collection and data entry is routinely monitored at the DCC using information entered into the data system (evaluation date and data collection date), and information stored in the systems inventory files (dates of entry, verification, and submission). The DCC will provide reports to the MINT CCC and clinical site coordinators including scheduling and delinquency. Inadequate performance of a site will be reported to the CCC and the site. The DCC and CCC will work with the site to find solutions to challenges. The DCC and CCC will evaluate the performance at that site to determine whether corrections have been made. Should problems persist, the Operations Committee will be notified along with recommendations for resolving persisting inadequacies. Trial and site performance reports will be provided to the sites, the CCC, the Steering Committee, and the DSMB.

H Study Monitoring, Auditing, and Inspecting

H1 Study Monitoring Plan

The CCC will coordinate the study monitoring site visits to each clinical site. In addition, the DCC will conduct data monitoring site visits to clinical sites where specific data issues are identified or data concerns arise based on a risk-based monitoring plan. The risk-based monitoring plan will be based on site performance metrics as well as the results of CCC safety and compliance monitoring visit.

H2 Auditing and Inspecting

During monitoring site visits, CCC personnel will review source documents, including informed consent documents, for a random sample of patients and for a defined subset with an exceptional number of inconsistencies identified through the data monitoring processes to confirm that data are

collected and entered accurately. Problems adhering to study protocols, data collection, entry, and management will be identified and corrective procedures will be addressed.

I Study Administration

I1 Organization and Participating Centers

1.a Clinical Coordinating Center

The Rutgers Robert Wood Johnson School of Medicine, Division of General Internal Medicine is responsible for clinical coordination of the trial. The responsibilities of the Clinical Coordinating Center are to: 1) Provide administrative and fiscal support for the Clinical Sites; 2) Provide technical, patient assessment and Protocol adherence advice to Clinical Site staff; 3) Assist Clinical Sites to correct problems with recruitment, Protocol adherence and data collection; 4) Participate in site visits; 5) Provide advice about any aspect of the trial; and 6) Lead presentation and publication of study results for the scientific and lay press.

A Canadian subsidiary clinical coordinating center to Rutgers Robert Wood Johnson Medical School will be housed at the University of Montreal. The Canadian Coordinating Center will carry out the clinical coordinating center responsibilities listed above for the Canadian sites and will be supervised by Dr. Paul Hebert and Dr. Jeffrey Carson. The Canadian Coordinating Center will have no data coordinating function.

1.b Data Coordinating Center

The Epidemiology Data Center at the University of Pittsburgh is responsible for the data coordination of the trial. The DCC will provide statistical leadership and resources for data management, quality control, and analysis. The responsibilities of the DCC include: 1) Maintain study website and provide all study materials, including the Manual of Procedures (MOP) and Data Collection Forms ; 2) Provide a 24 hour randomization service; 3) Establish and maintain an electronic database to receive study data and to verify for completeness, retrieving any missing data from the centers; 4) Conduct routine data edits; 5) Monitor performance to detect problems with recruitment, protocol adherence and data collection; 6) Participate in site visits; 7) Provide advice about any aspect of the trial; 8) Perform interim and final analyses.

1.c Clinical Consortium

The clinical sites recruiting patients into MINT comprise the Clinical Consortium of participating hospitals. Each site will be led by the Clinical Site Director and Clinical Site Coordinator. The Clinical Site Director and Coordinator will work closely together to assure successful performance of the trial.

The responsibilities of the Clinical Site Director include: 1) To insure that all medical staff involved with the care of myocardial infarction patients are well informed about the trial; 2) To insure that all patients with myocardial infarction are routinely considered for the trial; 3) To insure that the transfusion strategy is followed, i.e., blood is given in the Restrictive Transfusion Strategy only for symptoms from anemia or when the lower threshold is reached and blood is given in the Liberal Transfusion Strategy when Hgb < 10 g/dL; 4) To communicate with Clinical Coordinating Center staff and Data Coordinating Center staff any problems or concerns related to the study; and 5) To assist the Clinical Site Coordinator as necessary.

The responsibilities of the Clinical Site Coordinator include: 1) To identify myocardial infarction patients for the trial; 2) To obtain informed consent from the patient or surrogate; 3) To inform treating staff of the patient's treatment assignment assigned transfusion strategy; 4) To complete data collection forms and process data edit queries; 5) To perform 30 and 180 day telephone follow-up; 6) To obtain and submit all relevant source documents for the Clinical Event Committee adjudication; 7) To participate in telephone calls with the Clinical Coordinating Center; 8) To train assistant site coordination and other staff at the Clinical Site.

I2 Funding Source and Conflicts of Interest

This trial is supported by the National Heart Lung and Blood Institute. All investigators will be required to declare any potential conflicts of interest.

I3 Committees

3.a Steering Committee

The primary decision making body of the MINT trial will be the Steering Committee and will include the Principal Investigators of the CCC and DCC, experts in cardiology, transfusion medicine, biostatistics, and the NHLBI project officers. The Steering Committee will have quarterly conference

calls and annual in-person meetings. The group will focus on the science of the study and to review priorities on a regular basis.

3.b Operations Committee

A smaller committee, the Operations Committee, will be a subset of the Steering Committee that will meet approximately twice per month via conference call and will be charged with reviewing operational and scientific issues for the trial and formulating recommendations for consideration by the full Steering Committee. The Operations Committee will be chaired by Dr. Carson and will include DCC PI, selected Steering Committee members, and the CCC and DCC project directors.

The Operations Committee will be empowered to execute operational decisions, and the Operations Committee Chair will have the authority to set meeting agendas and appoint committee chairs as needed. The committee will ensure that recruitment for the network is progressing as planned for the clinical trial. In addition to reviewing monthly recruitment reports and quarterly follow-up data collection reports, the group will make specific recommendations for strategies to improve recruitment and protocol implementation.

3.c Executive Committee

A small Executive Committee will identify high level study design and implementation issues that are to be discussed by the Steering Committee and to resolve issues that require immediate action. The committee will include the PIs of the CCC and the DCC and the lead clinical investigator from the United States and from Canada.

3.d Clinical Events Committee

A Clinical Events Committee will be responsible for adjudication of myocardial infarctions. Committee members will be masked to transfusion strategy assignment when they review and adjudicate patient event packets.

I4 Subject Stipends or Payments

Study patients will not receive any stipends or payments

I5 Study Timetable

This study will require 5 years for completion. The timeline and benchmarks are as follows:

Benchmarks	Timeline
Planning and Organization Phase 1: Finalize the Manual of Operations and data collection instruments. Communicate with the Clinical Site Directors at each study site. Obtain IRB approval at clinical sites with assistance from CCC staff. Develop computer software for randomization and data entry.	Months 1-4<br><br>(9/1/16 – 12/31/16)
Planning and Organization Phase 2: Communicate final study protocol and procedures. Plan and schedule training. Hold annual Collaborators' Meeting for Clinical Site Directors and Coordinators. Distribute final forms.	Months 5-6<br><br>(1/1/17 – 2/28/17)
Recruitment and Follow-up Phase: Initiate patient enrollment at the clinical sites. Recruitment will start once a site has IRB approval, an executed contract, and staff have been trained. Continue for 4.0 years, until patient recruitment is completed, or the study is stopped early. Hold annual Collaborators' Meetings.	Month 7- Year 4. 5.<br><br>(3/1/17– 2/28/21)
Close-out and Analysis Phase: Complete follow-up and data cleaning. Perform data analysis. Discuss results at Collaborators' Meeting. Present trial results.	Year 4. 5<br><br>(3/1/21 – 8/31/21)

The planned accrual timeline, including the target and the cumulative target enrollment for each quarter, is provided in Appendix 4.

J Publication Plan

The Publications Committee will include physicians, scientists, and statisticians involved with the MINT trial. The chair of the committee will rotate bi-annually. This committee will be charged with coordinating the publication of study results and ensuring that these publications move forward according to schedule. Our plan is that the primary results of the trial will be submitted for publication within 9 months of the completion the trial. This will allow three months for data management and cleaning, three months for analysis and three months for manuscript preparation.

Dr. Carson will coordinate the effort for the primary results paper and the author byline will include the writing team “and the MINT Trial Investigators”. The primary results writing team will be comprised of the members of the Steering Committee. An appendix listing all of the MINT investigators including the Steering Committee members, the Site Investigators, the NIH program officers, the DSMB and other relevant contributors to the trial will appear at the end of the paper.

There will be additional papers that evaluate secondary hypotheses. The Publications Committee will review proposals for all non-primary papers. Approval of a paper proposal will require that the topic is clinically relevant, the paper does not overlap with an existing topic, and the general methodology is sound. Secondary papers will have individually named authors and will include “for the MINT Trial Investigators” in the author byline. The investigators who propose the concept will automatically be part of the writing team. Investigators from the Steering Committee and the Sites will have the opportunity to sign up for proposed writing teams. If more than 12 investigators sign up for an individual writing group, the Publications Committee will allocate investigators to writing teams based on contributions to the trial and expertise on the specific topic.

In order to ensure that the trial is using its resources to report the topics of greatest clinical relevance, the trial Investigators will be asked for ideas for papers and the Steering Committee will be asked to identify the ten most important papers. If a paper on this list is not moving forward according to schedule, the Publication Committee will notify the Steering Committee and may recommend a change in leadership for the paper. The Steering Committee will make final decisions about the leadership for papers.

K References

1. Sullivan MT, Cotten R, Read EJ, Wallace EL. Blood collection and transfusion in the United States in 2001. Transfusion (Paris). 2007;47(3):385-94. PubMed PMID: 17319817.

2. Organization. WH. 10 facts on blood transfusion: World Health Organization; 2015 [cited 2015 September 11, 2015]. Available from: http://www.who.int/features/factfiles/blood_transfusion/en/.

3. Heddle NLY, ; Barty, R. Ontario Data Collection Startegy: Proof of Concept. Submitted to MOHLTC. Submitted. 2013.

4. Shehata NF, A,; Lawrence, N.; Rothwell, D.M.; Hebert, P.; Fergusson D. Use of administrative data to determine the recipients and trends of blood transfusion. Submitted. 2013.

5. Shander A, Hofmann A, Ozawa S, Theusinger OM, Gombotz H, Spahn DR. Activity-based costs of blood transfusions in surgical patients at four hospitals. Transfusion (Paris). 2010;50(4):753-65. Epub 2009/12/17. doi: TRF2518 [pii]

10.1111/j.1537-2995.2009.02518.x. PubMed PMID: 20003061.

6. Zou S, Stramer SL, Notari EP, Kuhns MC, Krysztof D, Musavi F, Fang CT, Dodd RY. Current incidence and residual risk of hepatitis B infection among blood donors in the United States. Transfusion (Paris). 2009;49(8):1609-20. Epub 2009/05/06. doi: 10.1111/j.1537-2995.2009.02195.x. PubMed PMID: 19413732.

7. Zou S, Dorsey KA, Notari EP, Foster GA, Krysztof DE, Musavi F, Dodd RY, Stramer SL. Prevalence, incidence, and residual risk of human immunodeficiency virus and hepatitis C virus infections among United States blood donors since the introduction of nucleic acid testing. Transfusion (Paris). 2010;50(7):1495-504. Epub 2010/03/30. doi: 10.1111/j.1537-2995.2010.02622.x. PubMed PMID: 20345570.

8. Rosamond W, Flegal K, Friday G, Furie K, Go A, Greenlund K, Haase N, Ho M, Howard V, Kissela B, Kittner S, Lloyd-Jones D, McDermott M, Meigs J, Moy C, Nichol G, O'Donnell CJ, Roger V, Rumsfeld J, Sorlie P, Steinberger J, Thom T, Wasserthiel-Smoller S, Hong Y. Heart disease and stroke statistics--2007 update: a report from the American Heart Association Statistics Committee and Stroke Statistics Subcommittee. Circulation. 2007;115(5):e69-171. Epub 2006/12/30. doi: CIRCULATIONAHA.106.179918 [pii]

10.1161/CIRCULATIONAHA.106.179918. PubMed PMID: 17194875.

9. Alexander KP, Chen AY, Wang TY, Rao SV, Newby LK, LaPointe NM, Ohman EM, Roe MT, Boden WE, Harrington RA, Peterson ED. Transfusion practice and outcomes in non-ST-segment elevation acute coronary syndromes. Am Heart J. 2008;155(6):1047-53. Epub 2008/06/03. doi: S0002-8703(08)00055-0 [pii]

10.1016/j.ahj.2008.01.009. PubMed PMID: 18513518.

10. Salisbury AC, Amin AP, Reid KJ, Wang TY, Masoudi FA, Chan PS, Alexander KP, Bach RG, Spertus JA, Kosiborod M. Hospital-acquired anemia and in-hospital mortality in patients with acute myocardial infarction. Am Heart J. 2011;162(2):300-9 e3. Epub 2011/08/13. doi: 10.1016/j.ahj.2011.05.021. PubMed PMID: 21835291.

11. Hebert PC, Wells G, Blajchman MA, Marshall J, Martin C, Pagliarello G, Tweeddale M, Schweitzer I, Yetisir E. A multicenter, randomized, controlled clinical trial of transfusion requirements in critical care. Transfusion Requirements in Critical Care Investigators, Canadian Critical Care Trials Group [see comments]. N Engl J Med. 1999;340(6):409-17. PubMed PMID: 219.

12. Hajjar LA, Vincent JL, Galas FR, Nakamura RE, Silva CM, Santos MH, Fukushima J, Kalil Filho R, Sierra DB, Lopes NH, Mauad T, Roquim AC, Sundin MR, Leao WC, Almeida JP, Pomerantzeff PM, Dallan LO, Jatene FB, Stolf NA, Auler JO, Jr. Transfusion requirements after cardiac surgery: the TRACS randomized controlled trial. JAMA. 2010;304(14):1559-67. Epub 2010/10/14. doi: 304/14/1559 [pii]

10.1001/jama.2010.1446. PubMed PMID: 20940381.

13. Carson JL, Terrin ML, Noveck H, Sanders DW, Chaitman BR, Rhoads GG, Nemo G, Dragert K, Beaupre L, Hildebrand K, Macaulay W, Lewis C, Cook DR, Dobbin G, Zakriya KJ, Apple FS, Horney RA, Magaziner J. Liberal or restrictive transfusion in high-risk patients after hip surgery. N Engl J Med. 2011;365(26):2453-62. Epub 2011/12/16. doi: 10.1056/NEJMoa1012452. PubMed PMID: 22168590; PMCID: 3268062.

14. Villanueva C, Colomo A, Bosch A, Concepcion M, Hernandez-Gea V, Aracil C, Graupera I, Poca M, Alvarez-Urturi C, Gordillo J, Guarner-Argente C, Santalo M, Muniz E, Guarner C. Transfusion strategies for acute upper gastrointestinal bleeding. N Engl J Med. 2013;368(1):11-21. doi: 10.1056/NEJMoa1211801. PubMed PMID: 23281973.

15. Carson JL, Grossman BJ, Kleinman S, Tinmouth AT, Marques MB, Fung MK, Holcomb JB, Illoh O, Kaplan LJ, Katz LM, Rao SV, Roback JD, Shander A, Tobian AA, Weinstein R, Swinton McLaughlin LG, Djulbegovic B, Clinical Transfusion Medicine Committee of the A. Red blood cell transfusion: a clinical practice guideline from the AABB*. Ann Intern Med. 2012;157(1):49-58. doi: 10.7326/0003-4819-157-1-201206190-00429. PubMed PMID: 22751760.

16. Carson JLG, G.; Heddle, N.M.; Grossman, B.J.; Cohn, C.S.; Fung, M.K.; Gersheimer, T.; Holcomb, J.B.; Kaplan, L.J.; Katz, L.M.; Peterson, N.; Ramsey, G.; Rao, S.V.; Roback, J.D.; Shander, A.; Tobian, A.A.R.;. Clinical practice guidelines from the AABB: Red bloodcell transfuison thresholds and storage. 2016;in press.

17. Anderson HT, Kessinger JM, McFarland WJ, Jr., Laks H, Geha AS. Response of the hypertrophied heart to acute anemia and coronary stenosis. Surgery. 1978;84(1):8-15. PubMed PMID: 272.

29. Garfinkle M, Lawler PR, Filion KB, Eisenberg MJ. Red blood cell transfusion and mortality among patients hospitalized for acute coronary syndromes: a systematic review. Int J Cardiol. 2013;164(2):151-7. doi: 10.1016/j.ijcard.2011.12.118. PubMed PMID: 22305811.

30. Wu WC, Rathore SS, Wang Y, Radford MJ, Krumholz HM. Blood transfusion in elderly patients with acute myocardial infarction. N Engl J Med. 2001;345(17):1230-6. PubMed PMID: 9513.

31. Carson JL, Reynolds RC, Klein HG. Bad bad blood? Crit Care Med. 2008;36(9):2707-8. Epub 2008/08/30. doi: 10.1097/CCM.0b013e3181843ee7

00003246-200809000-00047 [pii]. PubMed PMID: 18728495.

32. Carson JL, Carless PA, Hebert PC. Transfusion thresholds and other strategies for guiding allogeneic red blood cell transfusion. Cochrane Database Syst Rev. 2012;4:CD002042. doi: 10.1002/14651858.CD002042.pub3. PubMed PMID: 22513904.

33. Carson JL, Carless PA, Hebert PC. Outcomes using lower vs higher hemoglobin thresholds for red blood cell transfusion. JAMA. 2013;309(1):83-4. doi: 10.1001/jama.2012.50429. PubMed PMID: 23280228.

34. Carson JL, Stanworth SJ, Roubinian N, Fergusson DA, Triulzi D, Doree C, Hebert PC. Transfusion thresholds and other strategies for guiding allogeneic red blood cell transfusion. Cochrane Database Syst Rev. 2016;10:CD002042. doi: 10.1002/14651858.CD002042.pub4. PubMed PMID: 27731885.

35. Docherty AB, O'Donnell R, Brunskill S, Trivella M, Doree C, Holst L, Parker M, Gregersen M, Pinheiro de Almeida J, Walsh TS, Stanworth SJ. Effect of restrictive versus liberal transfusion strategies on outcomes in patients with cardiovascular disease in a non-cardiac surgery setting: systematic review and meta-analysis. BMJ. 2016;352:i1351. doi: 10.1136/bmj.i1351. PubMed PMID: 27026510; PMCID: PMC4817242.

36. Patel NN, Avlonitis VS, Jones HE, Reeves BC, Sterne JA, Murphy GJ. Indications for red blood cell transfusion in cardiac surgery: a systematic review and meta-analysis. Lancet Haematol. 2015;2(12):e543-53. doi: 10.1016/S2352-3026(15)00198-2. PubMed PMID: 26686409.

37. Cooper HA, Rao SV, Greenberg MD, Rumsey MP, McKenzie M, Alcorn KW, Panza JA. Conservative versus liberal red cell transfusion in acute myocardial infarction (the CRIT Randomized Pilot Study). Am J Cardiol. 2011;108(8):1108-11. Epub 2011/07/28. doi: 10.1016/j.amjcard.2011.06.014. PubMed PMID: 21791325.

38. Thygesen K, Alpert JS, Jaffe AS, Simoons ML, Chaitman BR, White HD, Joint ESCAAHAWHFTFfUDoMI, Authors/Task Force Members C, Thygesen K, Alpert JS, White HD, Biomarker S, Jaffe AS, Katus HA, Apple FS, Lindahl B, Morrow DA, Subcommittee ECG, Chaitman BR, Clemmensen PM, Johanson P, Hod H, Imaging S, Underwood R, Bax JJ, Bonow JJ, Pinto F, Gibbons RJ, Classification S, Fox KA, Atar D, Newby LK, Galvani M, Hamm CW, Intervention S,

Uretsky BF, Steg PG, Wijns W, Bassand JP, Menasche P, Ravkilde J, Trials, Registries S, Ohman EM, Antman EM, Wallentin LC, Armstrong PW, Simoons ML, Trials, Registries S, Januzzi JL, Nieminen MS, Gheorghiade M, Filippatos G, Trials, Registries S, Luepker RV, Fortmann SP, Rosamond WD, Levy D, Wood D, Trials, Registries S, Smith SC, Hu D, Lopez-Sendon JL, Robertson RM, Weaver D, Tendera M, Bove AA, Parkhomenko AN, Vasilieva EJ, Mendis S, Guidelines ESCCfP, Bax JJ, Baumgartner H, Ceconi C, Dean V, Deaton C, Fagard R, Funck-Brentano C, Hasdai D, Hoes A, Kirchhof P, Knuuti J, Kolh P, McDonagh T, Moulin C, Popescu BA, Reiner Z, Sechtem U, Sirnes PA, Tendera M, Torbicki A, Vahanian A, Windecker S, Document R, Morais J, Aguiar C, Almahmeed W, Arnar DO, Barili F, Bloch KD, Bolger AF, Botker HE, Bozkurt B, Bugiardini R, Cannon C, de Lemos J, Eberli FR, Escobar E, Hlatky M, James S, Kern KB, Moliterno DJ, Mueller C, Neskovic AN, Pieske BM, Schulman SP, Storey RF, Taubert KA, Vranckx P, Wagner DR. Third universal definition of myocardial infarction. J Am Coll Cardiol. 2012;60(16):1581-98. doi: 10.1016/j.jacc.2012.08.001. PubMed PMID: 22958960.

39. Roubinian N. Variation of Transfusion Practice. 2015 (personal communication).

40. Salisbury AC, Reid KJ, Amin AP, Spertus JA, Kosiborod M. Variation in the incidence of hospital-acquired anemia during hospitalization with acute myocardial infarction (data from 57 US hospitals). Am J Cardiol. 2014;113(7):1130-6. doi: 10.1016/j.amjcard.2013.12.017. PubMed PMID: 24485696.

41. Salisbury AC, Reid KJ, Marso SP, Amin AP, Alexander KP, Wang TY, Spertus JA, Kosiborod M. Blood transfusion during acute myocardial infarction: association with mortality and variability across hospitals. J Am Coll Cardiol. 2014;64(8):811-9. doi: 10.1016/j.jacc.2014.05.040. PubMed PMID: 25145526.

42. Sherwood MW, Wang Y, Curtis JP, Peterson ED, Rao SV. Patterns and outcomes of red blood cell transfusion in patients undergoing percutaneous coronary intervention. JAMA. 2014;311(8):836-43. Epub 2014/02/27. doi: 10.1001/jama.2014.980. PubMed PMID: 24570247.

43. Magruder JT, Blasco-Colmenares E, Crawford T, Alejo D, Conte JV, Salenger R, Fonner CE, Kwon CC, Bobbitt J, Brown JM, Nelson MG, Horvath KA, Whitman GR. Variation in Red Blood Cell Transfusion Practices During Cardiac Operations Among Centers in Maryland: Results From a State Quality-Improvement Collaborative. Ann Thorac Surg. 2017;103(1):152-60. doi: 10.1016/j.athoracsur.2016.05.109. PubMed PMID: 27553501.

44. Miller YB, G.; Benjamin, R.; Eklund, D.K.; et al. Practice Guidelines for Blood Transfusion; A Complilation from Recent Peer-Reivewed Literature. American Red Cross. 2nd ed: American Red Cross; 2007.

45. Carson JL, Grossman BJ, Kleinman S, Tinmouth AT, Marques MB, Fung MK, Holcomb JB, Illoh O, Kaplan LJ, Katz LM, Rao SV, Roback JD, Shander A, Tobian AA, Weinstein R, Swinton McLaughlin LG, Djulbegovic B. Red Blood Cell Transfusion: A Clinical Practice Guideline From the AABB. Annals of internal medicine. 2012. Epub 2012/03/29. doi: 10.1059/0003-4819-156-12-201206190-00429. PubMed PMID: 22454395.

46. Qaseem A, Humphrey LL, Fitterman N, Starkey M, Shekelle P, Clinical Guidelines Committee of the American College of P. Treatment of anemia in patients with heart disease: a clinical practice guideline from the American College of Physicians. Ann Intern Med. 2013;159(11):770-9. Epub 2013/12/04. doi: 10.7326/0003-4819-159-11-201312030-00009. PubMed PMID: 24297193.

47. American College of Emergency P, Society for Cardiovascular A, Interventions, O'Gara PT, Kushner FG, Ascheim DD, Casey DE, Jr., Chung MK, de Lemos JA, Ettinger SM, Fang JC, Fesmire FM, Franklin BA, Granger CB, Krumholz HM, Linderbaum JA, Morrow DA, Newby LK, Ornato JP, Ou N, Radford MJ, Tamis-Holland JE, Tommaso CL, Tracy CM, Woo YJ, Zhao DX, Anderson JL, Jacobs AK, Halperin JL, Albert NM, Brindis RG, Creager MA, DeMets D, Guyton RA, Hochman JS, Kovacs RJ, Kushner FG, Ohman EM, Stevenson WG, Yancy CW. 2013 ACCF/AHA guideline for the management of ST-elevation myocardial infarction: executive summary: a report of the American College of Cardiology Foundation/American Heart Association Task Force on Practice Guidelines. J Am Coll Cardiol. 2013;61(4):485-510. doi: 10.1016/j.jacc.2012.11.018. PubMed PMID: 23256913.

48. O'Gara PT, Kushner FG, Ascheim DD, Casey DE, Jr., Chung MK, de Lemos JA, Ettinger SM, Fang JC, Fesmire FM, Franklin BA, Granger CB, Krumholz HM, Linderbaum JA, Morrow DA, Newby LK, Ornato JP, Ou N, Radford MJ, Tamis-Holland JE, Tommaso JE, Tracy CM, Woo YJ, Zhao DX, Force CAT. 2013 ACCF/AHA guideline for the management of ST-elevation myocardial infarction: executive summary: a report of the American College of Cardiology Foundation/American Heart Association Task Force on Practice Guidelines. Circulation. 2013;127(4):529-55. doi: 10.1161/CIR.0b013e3182742c84. PubMed PMID: 23247303.

49. Rogers GMB, M.; Cela, D.; Cleeland, C. et al. NCCN Guidelines Version 2.2014 Cancer- and Chemotherapy-Induced Anemia. NCCN Clinical Practice Guidelines in Oncology (NCCN Guidelines) [Internet]. 2013.

50. Patient Blood Management Guidelines: National Blood Authority, Australia; 2012 [June 14, 2014]. Available from: http://www.blood.gov.au/pbm-guidelines

51. Hamm CW, Bassand JP, Agewall S, Bax J, Boersma E, Bueno H, Caso P, Dudek D, Gielen S, Huber K, Ohman M, Petrie MC, Sonntag F, Uva MS, Storey RF, Wijns W, Zahger D, Guidelines ESCCfP, Bax JJ, Auricchio A, Baumgartner H, Ceconi C, Dean V, Deaton C, Fagard R, Funck-Brentano C, Hasdai D, Hoes A, Knuuti J, Kolh P, McDonagh T, Moulin C, Poldermans D, Popescu BA, Reiner Z, Sechtem U, Sirnes PA, Torbicki A, Vahanian A, Windecker S, Document R, Windecker S, Achenbach S, Badimon L, Bertrand M, Botker HE, Collet JP, Crea F, Danchin N, Falk E, Goudevenos J, Gulba D, Hambrecht R, Herrmann J, Kastrati A, Kjeldsen K, Kristensen SD, Lancellotti P, Mehilli J, Merkely B, Montalescot G, Neumann FJ, Neyses L, Perk J, Roffi M, Romeo F, Ruda M, Swahn E, Valgimigli M, Vrints CJ, Widimsky P. ESC Guidelines for the management of acute coronary syndromes in patients presenting without persistent ST-segment elevation: The Task Force for the management of acute coronary syndromes (ACS) in patients presenting without persistent ST-segment elevation of the European Society of Cardiology (ESC). Eur Heart J. 2011;32(23):2999-3054. doi: 10.1093/eurheartj/ehr236. PubMed PMID: 21873419.

52. Carson JL, Guyatt G, Heddle NM, Grossman BJ, Cohn CS, Fung MK, Gernsheimer T, Holcomb JB, Kaplan LJ, Katz LM, Peterson N, Ramsey G, Rao SV, Roback JD, Shander A, Tobian

AA. Clinical Practice Guidelines From the AABB: Red Blood Cell Transfusion Thresholds and Storage. JAMA. 2016. doi: 10.1001/jama.2016.9185. PubMed PMID: 27732721.

53. Alexander J, Cifu AS. Transfusion of red blood cells. JAMA. 2016. doi: 10.1001/jama.2016.12870. PubMed PMID: 27732722.

54. Ellingson KD, Sapiano MRP, Haass KA, Savinkina AA, Baker ML, Chung KW, Henry RA, Berger JJ, Kuehnert MJ, Basavaraju SV. Continued decline in blood collection and transfusion in the United States-2015. Transfusion (Paris). 2017;57 Suppl 2:1588-98. doi: 10.1111/trf.14165. PubMed PMID: 28591469.

55. Roubinian NH, Escobar GJ, Liu V, Swain BE, Gardner MN, Kipnis P, Triulzi DJ, Gottschall JL, Wu Y, Carson JL, Kleinman SH, Murphy EL, Epidemiology NR, Donor Evaluation S. Trends in red blood cell transfusion and 30-day mortality among hospitalized patients. Transfusion (Paris). 2014;54(10 Pt 2):2678-86. doi: 10.1111/trf.12825. PubMed PMID: 25135770; PMCID: 4197097.

56. Proceedings from the National Summit on Overuse. September 24, 2012 2012 [August 5, 2017]. Available from: https://www.jointcommission.org/assets/1/6/National_Summit_Overuse.pdf.

57. Rao SV, Chiswell K, Sun JL, Granger CB, Newby LK, Van de Werf F, White HD, Armstong PW, Califf RM, Harrington RA. International variation in the use of blood transfusion in patients with non-ST-segment elevation acute coronary syndromes. Am J Cardiol. 2008;101(1):25-9. Epub 2007/12/26. doi: S0002-9149(07)01822-X [pii]

10.1016/j.amjcard.2007.07.042. PubMed PMID: 18157960.

58. Spitalnik SL, Triulzi D, Devine DV, Dzik WH, Eder AF, Gernsheimer T, Josephson CD, Kor DJ, Luban NL, Roubinian NH, Mondoro T, Welniak LA, Zou S, Glynn S, State of the Science in Transfusion Medicine Working G. 2015 proceedings of the National Heart, Lung, and Blood Institute's State of the Science in Transfusion Medicine symposium. Transfusion (Paris). 2015. doi: 10.1111/trf.13250. PubMed PMID: 26260861.

59. Vlaar AP, Juffermans NP. Transfusion-related acute lung injury: a clinical review. Lancet. 2013. doi: 10.1016/S0140-6736(12)62197-7. PubMed PMID: 23642914.

60. Klein HG, Spahn DR, Carson JL. Red blood cell transfusion in clinical practice. Lancet. 2007;370(9585):415-26. Epub 2007/08/07. doi: S0140-6736(07)61197-0 [pii]

10.1016/S0140-6736(07)61197-0. PubMed PMID: 17679019.

61. Bassand JP, Hamm CW, Ardissino D, Boersma E, Budaj A, Fernandez-Aviles F, Fox KA, Hasdai D, Ohman EM, Wallentin L, Wijns W. Guidelines for the diagnosis and treatment of non-ST-segment elevation acute coronary syndromes. Eur Heart J. 2007;28(13):1598-660. Epub 2007/06/16. doi: 10.1093/eurheartj/ehm161. PubMed PMID: 17569677.

62. Schulman S, Kearon C, Subcommittee on Control of Anticoagulation of the S, Standardization Committee of the International Society on T, Haemostasis. Definition of major bleeding in clinical investigations of antihemostatic medicinal products in non-surgical patients. J Thromb Haemost. 2005;3(4):692-4. doi: 10.1111/j.1538-7836.2005.01204.x. PubMed PMID: 15842354.

18. Hagl S, Heimisch W, Meisner H, Erben R, Baum M, Mendler N. The effect of hemodilution on regional myocardial function in the presence of coronary stenosis. Basic Res Cardiol. 1977;72(4):344-64. PubMed PMID: 9839.

19. Wilkerson DK, Rosen AL, Sehgal LR, Gould SA, Sehgal HL, Moss GS. Limits of cardiac compensation in anemic baboons. Surgery. 1988;103(6):665-70. PubMed PMID: 232.

20. Carson JL, Duff A, Poses RM, Berlin JA, Spence RK, Trout R, Noveck H, Strom BL. Effect of anaemia and cardiovascular disease on surgical mortality and morbidity. Lancet. 1996;348(9034):1055-60. Epub 1996/10/19. doi: S0140673696043309 [pii]. PubMed PMID: 8874456.

21. Silvain J, Abtan J, Kerneis M, Martin R, Finzi J, Vignalou JB, Barthelemy O, O'Connor SA, Luyt CE, Brechot N, Mercadier A, Brugier D, Galier S, Collet JP, Chastre J, Montalescot G. Impact of red blood cell transfusion on platelet aggregation and inflammatory response in anemic coronary and noncoronary patients: the TRANSFUSION-2 study (impact of transfusion of red blood cell on platelet activation and aggregation studied with flow cytometry use and light transmission aggregometry). J Am Coll Cardiol. 2014;63(13):1289-96. doi: 10.1016/j.jacc.2013.11.029. PubMed PMID: 24361322.

22. Rodman T, Close HP, Purcell MK. The oxyhemoglobin dissociation curve in anemia. Ann Intern Med. 1960;52:295-309. PubMed PMID: 731.

23. Kennedy AC, Valtis DJ. The oxygen dissociation curve in anemia of various types. JClinInvest. 1954;33:1372-81. PubMed PMID: 735.

24. McMahon TJ, Moon RE, Luschinger BP, Carraway MS, Stone AE, Stolp BW, Gow AJ, Pawloski JR, Watke P, Singel DJ, Piantadosi CA, Stamler JS. Nitric oxide in the human respiratory cycle. Nat Med. 2002;8(7):711-7. doi: 10.1038/nm718. PubMed PMID: 12042776.

25. LaCelle PL. Alteration of deformability of the erythrocyte membrane in stored blood. Transfusion (Paris). 1969;9:238-45. PubMed PMID: 4555.

26. Simchon S, Jan KM, Chien S. Influence of reduced red cell deformability on regional blood flow. Am J Physiol. 1987;253:H898-H903. PubMed PMID: 356.

27. Marik PE, Corwin HL. Efficacy of red blood cell transfusion in the critically ill: a systematic review of the literature. Crit Care Med. 2008;36(9):2667-74. Epub 2008/08/06. doi: 10.1097/CCM.0b013e3181844677. PubMed PMID: 18679112.

28. Chatterjee SW, J; Sharma,A.;Lichstein, E.; Mukherjee,D. Blood Transfusion is Associated with Increased Mortality in Myocardial Infarction-A Meta-analysis and Diversity-Adjusted Study Sequential Analysis. Arch Intern Med. 2012;172.

L Appendices

L1 Appendix 1: Standardized approach and script to be used to seek permission from attending physician to recruit a patient

For each patient who is potentially eligible for the trial, the MINT site investigator or coordinator will contact the patient's attending physician, with expertise in cardiovascular care, and use the following script:

> "Dr. <name> I am calling to seek your permission to approach your patient <name> for consent to participate in the MINT trial. This is an NIH-sponsored trial that enrolls patients with an acute myocardial infarction and a hemoglobin level <10. According to the protocol, those assigned to the Liberal Arm receive red blood cell transfusions to maintain a hemoglobin level ≥ 10. Those assigned to the Restrictive Arm will be permitted to receive a transfusion if the hemoglobin concentration falls below 8 g/dL and will be strongly recommended to receive transfusion if the hemoglobin concentration is below 7 g/dL and you determine that a transfusion is in the patient's best interest. Transfusion is always permitted for active bleeding or if there are angina symptoms that you believe are related to the anemia and are not adequately controlled with medications. Patients can be withdrawn from the transfusion protocol if clinical circumstance change and adherence to the protocol places the patient at risk."
>
> "We want to confirm that <name> is an appropriate candidate for the trial; and that in your clinical judgement both approaches to transfusion are consistent with good medical care for this patient."

The first paragraph of the script will be used the initial time the trial team contacts the physician but could be skipped if the physician is already familiar with the protocol. The second paragraph will be stated when contacting an attending physician for any potential patient whether or not the physician has been contacted by the MINT team in the past. Patients will only be approached for consent if their physician confirms his/her belief that either transfusion strategy is appropriate, per the second paragraph.

L2 Appendix 2: MINT Data Elements by Study Time Point

	Screening	Baseline	Randomization	12 hours Post Randomization	Day 1	Day 2	Day 3	Daily Up to 30 Days	Hospital Discharge/ 30 Days	30 Day Follow-up	6 Month Follow-up
**Confirm Eligibility**	X										
**Informed Consent**	X										
**Hemoglobin Level**[a]		X[b,c]			X[b]	X[b]	X[b]	X			
**Troponin Level**[a]		X[b,c]		X[b]	X[b]	X[b]	X[b]	X			
**ECG**[a]		X[b,c]			X[b]	X[b]	X[b]	X			
**Demographics**	X										
**Medical History**		X									
**Medications**		X									
**Implement Transfusion Strategy**			X--X								
**RBC Transfusions**[d]			X--X								
**Study Outcomes**									X	X	X[e]
**Quality of Life (EQ-5D)**										X	
**Vital Status**									X	X	X
**Assessment of AEs and UPs Safety Monitoring**		X--X									

[a] in addition to required time points, all performed for clinical reasons are collected

[b] required, if still in hospital

[c] within the 24 hours prior to randomization

[d] including unit information (e.g, leukoreduction)

[d] mortality outcome only

L3 Appendix 3: SAE and UP Event Reporting Timelines

What Event is Reported	When is Event Reported	By Whom is Event Reported	To Whom is Event Reported
Fatal or life-threatening unexpected, suspected serious adverse reactions	Within **7 calendar days** of initial receipt of information	Investigator	Local/internal IRBs<br>NHLBI and/or Data Coordinating Center (DCC)
		Sponsor or designee[1]	FDA (if IND study)
Non-fatal, non-life-threatening unexpected, suspected serious adverse reactions	Within **15 calendar days** of initial receipt of information	Investigator	Local/internal IRBs/Institutional Officials<br>NHLBI and/or DCC
		Sponsor or designee	FDA (IND/Marketed Products)<br>All participating investigators
Unanticipated adverse device effects	Within **10** working days of investigator first learning of effect	Investigator	Local/internal IRBs<br>NHLBI and/or DCC
		Sponsor or designee	FDA (if IDE study)
Unanticipated Problem that is not an SAE	Within **14 days** of the investigator becoming aware of the problem	Investigator	Local/internal IRBs/Institutional Officials,<br>NHLBI and/or DCC
All Unanticipated Problems[2]	Within **30 days** of the IRB's receipt of the report of the UP from the investigator.	IRB	OHRP
		Investigator[3]	External IRBs

1. Designee is appointed by the sponsor; for example, DCC, CRO.
2. Per OHRP guidance: only when a particular AE or series of AEs is determined to meet the criteria for an UP should a report of the AE(s) be submitted to the IRB at each institution under the HHS regulations at 45 CFR part 46. Typically, such reports to the IRBs are submitted by investigators.
3. Investigators should also take into account local IRB guidance if reporting timelines for UPs are shorter than OHRP guidance

L4 Appendix 4: Accrual Timeline Milestones

The principles guiding the accrual timeline are as follows. The specific target numbers are provided in the table.

- Recruitment will start 6 months after funding of the trial
- 50% of the projected sites will initiate recruitment between month 6 and 12
- All sites will be recruiting by month 18
- Recruitment will extend for 4 years
- The recruitment rate will get faster over time.
- Total sample size is 3500 patients.

Date	Proposed Target Accrual	Proposed Cumulative Accrual
6/1/17	Start	Start
9/1/17	43	43
12/1/17	63	106
3/1/18	86	192
6/1/18	106	298
9/1/18	121	419
12/1/18	136	555
3/1/19	151	706
6/1/19	166	872
9/1/19	191	1063
12/1/19	227	1290
3/1/20	275	1565
6/1/20	340	1905
9/1/20	380	2285
12/1/20	400	2685
3/1/21	405	3090
6/1/21	410	3500

MINT Target Accrual Per Quarter

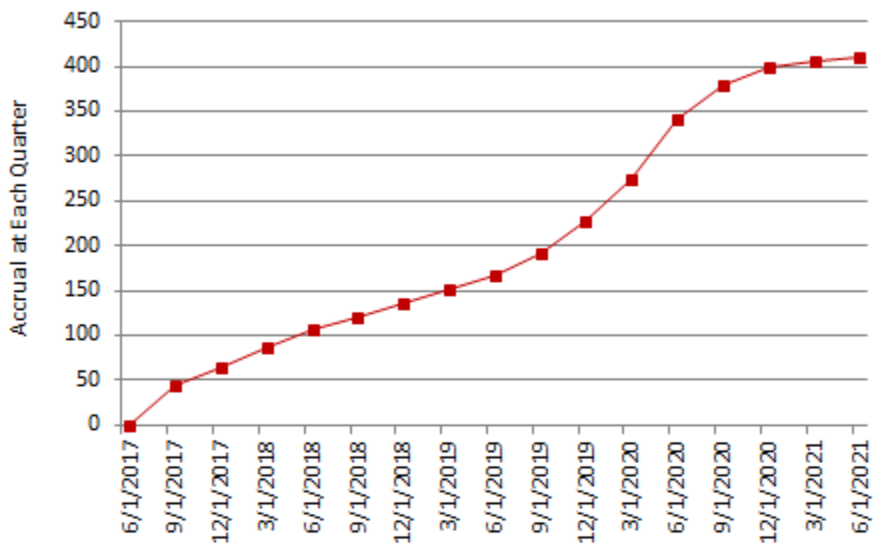

MINT Target Cumulative Accrual per Quarter

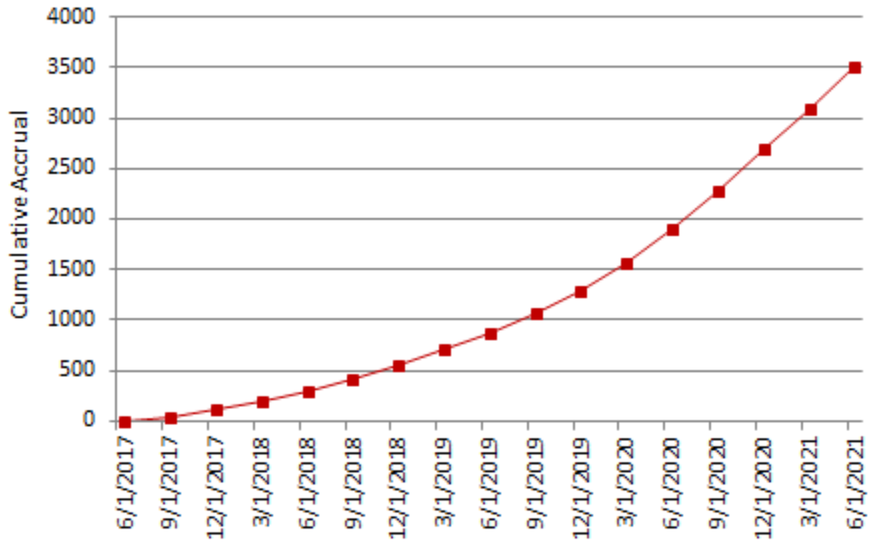

The specific changes to the protocol are as follows:

Myocardial Ischemia and Transfusion

Summary of changes to MINT Protocol

Protocol Version	7.1 October 24, 2017
Replace<br>Protocol Version	6.1 December 28 2016
Pages 15-16	**Systematic Reviews, Clinical Trials**: More detail to summarize the findings of published clinical trials has been added
Pages 17 -18	**Variation in Transfusion and Guidelines:** More detail and updated information has been added
Page 22	**Inclusion Criteria:** The additional criterion that patient physician agrees to patient's recruitment has been added
Page 23	**Exclusion Criteria:** Added that any patient can be excluded at attending physicians discretion<br><br>**Ethical Considerations:** Requirement that patient physician confirm his agreement that the patient could be recruited into the trial has been added
Pages 24 & 29 & Appendix 1 (page 67)	**Subject Recruitment Plans and Consent Process:** Standardized script to be used when requesting patient physician permission to recruit has been added
Page 43 & Appendix 4 (page 72)	**Interim Monitoring and Early Stopping:** The study timeline has been updated
Page 55	**Clinical Events Committee:** Outcomes that will not be adjudicated have been removed from the tasks
Pages 67 - 72	Standardized Script has been added as Appendix L1. Former Appendices L1- L3 have been renumbered to be Appendices L2-L4

The Statistical Analysis Plan for the
Myocardial Ischemia and Transfusion (MINT) Trial

July 8, 2022

INTRODUCTION

This Statistical Analysis Plan for the Myocardial Ischemia and Transfusion (MINT) trial is consistent with the statistical methods outlined in the trial protocol. This document includes 1) a brief summary of the MINT trial design, 2) the trial aims, 3) definitions of the specified MINT trial outcomes, 4) the original sample size and power calculations, 5) the interim monitoring plan, 6) analytic approach, and 7) proposed tables and figures.

1. TRIAL DESIGN

Study Design

The MINT trial is a randomized, open-label, multicenter clinical trial designed to determine whether a liberal transfusion strategy with a threshold of 10 g/dL reduces the composite outcome of all-cause mortality or nonfatal myocardial reinfarction through 30 days, compared with a restrictive transfusion strategy with a threshold of 7 to 8 g/dL among patients with an acute myocardial infarction and a hemoglobin concentration less than 10 g/dL. The trial is conducted in approximately 144 clinical sites in the United States, Canada, France, Spain, Brazil, New Zealand, and Australia. The two transfusion strategies are allocated with a 1:1 ratio using a permuted block design, with random variable block sizes, stratified by clinical site.

Population

The eligible study population includes adult patients with ST-segment elevation myocardial infarction or non ST-segment elevation myocardial infarction consistent with the 3rd Universal Definition of Myocardial Infarction criteria that occurs on admission or during the index hospitalization, and anemia defined as a hemoglobin concentration less than 10 g/dL at the time of randomization. Patients with Type 1, Type 2,

Type 4b, and Type 4c are eligible, and the occurrence of the index MI is determined by the enrolling site study team.

Trial Transfusion Strategies

The trial compares a restrictive and a liberal approach to transfusion therapy. The transfusion strategy is followed during the index hospitalization from the time of randomization up to 30-days post-randomization, hospital discharge, or death, whichever comes first.

Restrictive Transfusion Strategy: Patients randomized to the restrictive transfusion strategy are to receive a transfusion if the hemoglobin concentration falls below 8 g/dL and are strongly encouraged to receive transfusion if the hemoglobin concentration is below 7 g/dL. Transfusion is also allowed when anginal symptoms are determined by the patient's treating physician to be related to anemia and are not controlled with anti-anginal medications. Enough blood is given to increase the hemoglobin concentration to above 7 to 8 g/dL or to relieve anginal symptoms.

Liberal Transfusion Strategy: Patients randomly allocated to the liberal transfusion strategy receive one unit of packed RBCs following randomization and will receive enough blood to raise the hemoglobin concentration to 10 g/dL or above any time during the index hospitalization that the hemoglobin concentration is detected to be below 10 g/dL. A post transfusion hemoglobin measurement showing a hemoglobin level of at least 10 g/dL must be obtained.

For both strategies, blood is administered one unit at a time followed by a hemoglobin measurement. A patient in either group may be transfused at any time without a hemoglobin level if the patient is actively bleeding (e.g., brisk gastrointestinal bleeding) and the treating physician believes an emergency transfusion is needed. A patient in either group with history of congestive heart failure or low ejection fraction may receive diuretics prior to or after transfusion and transfusion may be delayed until the patient can safely tolerate the additional volume. Patients with end stage renal disease may receive transfusion during dialysis if requested by treating physician. The transfusion protocol is suspended for 24 hours if the patient goes to surgery.

Assessments of laboratory markers

The trial collects hemoglobin and troponin levels and electrocardiogram (ECG) readings at specified time points while the patient is in the hospital. The required collection time points for hemoglobin levels and ECGs are within 24 hours prior to randomization, and on days 1, 2 and 3 post-randomization. The

required time points for the troponin levels are within 24 hours prior to randomization and post randomization, at 12 hours, and on days 1, 2 and 3. All hemoglobin and troponin levels performed for during the index hospitalization are collected by the trial.

Participant Follow-up

At hospital discharge, death or 30 days post randomization, whichever comes first, the study staff submit data related to the hospitalization including health status, laboratory results, blood transfusions, and post randomization clinical events. Study staff contact the patient at 30 days post-randomization to ascertain vital status, administer the quality-of-life questionnaire, and determine if there has been a subsequent hospital admission or emergency room visit. Study staff obtain and review the medical records for each readmission and record relevant data and study outcomes. If an acute coronary syndrome event is suspected from the time of randomization until 30-days post-randomization, the staff submits documentation for central review. Study staff contact participants at 6 months following randomization to ascertain the patient's vital status.

Recurrent myocardial infarction is diagnosed when a suspected myocardial ischemic event is reported by the investigators at the clinical sites and confirmed by the Clinical Event Committee. In addition to adjudication of site suspected events, the trial has a surveillance process in which all cardiac troponin values are reviewed by the Clinical Event Committee to detect abnormal biomarker patterns. When criteria are met, the Clinical Event Committee requests that the site submit medical records and ECGs that are temporally related to the abnormal values. The Clinical Events Committee adjudicates occurrences of myocardial infarction, masked to assigned transfusion strategy, from randomization through 30-days post-randomization. Re-infarction is diagnosed when biomarker and clinical or ECG criteria of myocardial ischemia meet the 3rd Universal Definition of MI definition.

2. TRIAL AIMS

Primary Aim

The MINT primary aim is to determine whether a liberal transfusion strategy with a threshold of 10 g/dL reduces the composite outcome of all-cause mortality or nonfatal myocardial reinfarction through 30 days following randomization, compared to a restrictive transfusion strategy with a threshold of 7 to 8 g/dL among patients with an acute myocardial infarction and a hemoglobin concentration less than 10 g/dL.

Secondary Aims

1) To determine whether a liberal (10g/dL) transfusion strategy reduces all-cause mortality within 30 days, compared to a restrictive transfusion strategy.

2) To determine whether a liberal (10g/dL) transfusion strategy reduces myocardial reinfarction within 30 days, compared to a restrictive transfusion strategy.

3) To determine whether a liberal (10g/dL) transfusion strategy reduces the composite outcome of all-cause mortality, nonfatal myocardial reinfarction, ischemia driven unscheduled coronary revascularization (percutaneous coronary intervention or coronary artery bypass grafting), or readmission to the hospital for ischemic cardiac diagnosis within 30 days, compared to a restrictive transfusion strategy.

Tertiary Aims

1) To determine whether a liberal (10g/dL) transfusion strategy reduces all-cause mortality, nonfatal myocardial reinfarction, or unstable angina (i.e. acute coronary syndrome) within 30 days, compared to a restrictive transfusion strategy.

2) To determine whether a liberal (10g/dL) transfusion strategy reduces ischemia driven unscheduled coronary revascularization within 30-days compared to a restrictive strategy.

3) To determine whether a liberal (10g/dL) transfusion strategy reduces unscheduled readmission to hospital for ischemic cardiac diagnosis within 30 days, compared to a restrictive strategy.

4) To determine whether a liberal (10g/dL) transfusion strategy increases congestive heart failure within 30 days, compared to a restrictive transfusion strategy.

5) To determine whether a liberal (10g/dL) transfusion strategy reduces unscheduled readmission to hospital for any reason within 30 days, compared to a restrictive strategy.

6) To determine whether a liberal (10g/dL) transfusion strategy increases each of the individual thrombotic/hemorrhagic outcomes of stroke, pulmonary embolism or deep venous thrombosis, and bleeding within 30 days, compared to a restrictive strategy.

7) To determine whether a liberal (10g/dL) transfusion strategy increases each of the individual infectious outcomes of pneumonia, blood stream, and urinary tract within 30 days, compared to a restrictive strategy.

8) To determine whether a liberal (10g/dL) transfusion strategy reduces each of the individual in-hospital outcomes of length of hospital stay post randomization and number of days in intensive care unit, compared to a restrictive strategy.

9) To determine whether a liberal (10g/dL) transfusion strategy increases patient reported quality of life using the EuroQol questionnaire (EQ-5D) at 30 days compared to a restrictive strategy

10 To determine whether a liberal (10g/dL) transfusion strategy reduces all-cause mortality at 6-months following randomization, compared to a restrictive strategy.

3. DEFINITIONS OF OUTCOMES

Myocardial Reinfarction: Myocardial reinfarction will be classified by the Clinical Events Committee using The Joint European Society of Cardiology/American College of Cardiology Committee definitions. Patients with reinfarction will need to demonstrate a fall in the troponin value and then a subsequent rise of at least 20% with additional evidence (new ECG changes, imaging evidence, clinical history) as in the MI definition to diagnose a new event. Myocardial reinfarction will be classified as ST-segment elevation or non-ST Segment elevation.

Death and Cause of Death: For each death, the cause will be determined by the site personnel into one of three categories: cardiovascular death (e.g., congestive heart failure, dysrhythmia), noncardiovascular death (e.g., infection, cancer), or undetermined cause of death. Information about the specific cause of death will also be collected.

Unscheduled Coronary Revascularization (unstaged): Ischemia driven, unscheduled coronary revascularization (coronary artery bypass surgery or PCI) within 30 days of randomization will be recorded by the sites. Prior to randomization, the site will record if a coronary revascularization is planned (staged). All coronary revascularization procedures will be recorded, but an elective planned staged procedure will not be included as an outcome. Information about the reason for the procedure will also be collected to ensure that the revascularization was done to treat ischemic heart disease.

Readmission to Hospital, overall and for primary cardiac diagnosis: All re-admissions to the hospital that had not been planned prior to randomization will be captured, and the primary diagnosis for each

hospitalization will be classified as: ischemic cardiac diagnosis (e.g., myocardial infarction, unstable angina), non-ischemic cardiac diagnosis (e.g. heart failure) or non-cardiac.

Unstable Angina: The MINT trial sites will use 2014 ACC/AHA Key Data Elements and Definitions for Cardiovascular Endpoint Events in Clinical Trials to define unstable angina. To diagnose unstable angina requires that four criteria be met: 1) worsening ischemic discomfort, 2) unscheduled hospitalization, 3) negative cardiac biomarker, 4) objective evidence of myocardial ischemia.

Congestive Heart Failure: Sites personnel will use 2014 ACC/AHA Key Data Elements and Definitions for Cardiovascular Endpoint Events in Clinical Trials to define congestive heart failure. New or worsening symptoms of congestive heart failure on presentation (increasing dyspnea, paroxysmal nocturnal dyspnea, orthopnea), has objective evidence of new or worsening heart failure, and receives initiation or intensification of treatment specifically for heart failure.

TIA or Stroke: The 2014 ACC/AHA Key Data Elements and Definitions for Cardiovascular Endpoint Events in Clinical Trials will be used to define stroke. A transient ischemic attack (TIA) is defined as "a transient episode of focal neurological dysfunction caused by brain, spinal cord, or retinal ischemia, without acute infarction." Stroke is defined on the basis of the presence of acute infarction as demonstrated by imaging or based on the persistence of symptoms more than 24 hours.

Deep Venous Thrombosis or Pulmonary Embolism: Deep vein thrombosis will be diagnosed if duplex ultrasound, magnetic resonance venogram (MRV), or venogram is definite or probable positive. Site investigators will record if location is proximal or distal. Pulmonary embolism will be diagnosed with a high probability ventilation perfusion lung scan, CT scan, or pulmonary angiogram.

Bleeding: Major bleeding will be defined as 1) fatal bleeding, and/or 2) symptomatic bleeding in a critical area or organ, such as intracranial, intraspinal, intraocular, retroperitoneal, intra- articular or pericardial, or intramuscular with compartment syndrome, and/or 3) bleeding causing a drop in hemoglobin concentration of 2 g/dL or greater (51) from the last hemoglobin concentration prior to randomization to the nadir hemoglobin concentration during hospitalization or up to 30 days post randomization. The drop

in hemoglobin concentration will account for each unit of red blood cell transfusion transfused by subtracting 1 g/dL for each unit administered.

Infections recorded include pneumonia, blood stream infections and urinary tract infections.

Pneumonia: Pneumonia will be diagnosed using CDC criteria which includes radiographic abnormalities and combination of symptoms (i.e., cough), signs (i.e., fever, tachypnea, or laboratory abnormalities (i.e., white blood cell count, hypoxemia).

Blood Stream Infection: Blood stream infection will be defined using CDC criteria which includes a recognized pathogen cultured from 1 or more blood cultures and organism cultured from blood is not related to an infection at another site, at least 1 of the following signs or symptoms: fever, chills, or hypotension and signs and symptoms and positive laboratory results are not related to an infection at another site and common skin contaminant is cultured from 2 or more blood cultures drawn on separate occasions.

Urinary Tract Infection: Urinary Tract Infection will be defined using CDC criteria (48) which include one of the following signs or symptoms: fever (>38.0°C) or localized pain or tenderness, and laboratory evidence for infection.

Length of Stay and Intensive Care Unit Days: The trial records the number of days post randomization that the patient is in the hospital and in intensive care unit.

Quality of Life: The EuroQol questionnaire (EQ-5D), a standardized instrument that measures health related quality of life in 5 dimensions: mobility, self-care, usual activities, pain/discomfort and anxiety/depression, is used as a measure of patient perceived global health status 30 days after randomization. A EQ-5D summary score and a Heath Rating will be reported.

4. SAMPLE SIZE DETERMINATION AND POWER

In the MINT pilot trial, the composite 30-day rate of death and myocardial infarction was 16.4%. Using a two-sided inequality test and a simple chi-square statistic with alpha=0.05, we determined the samples sizes required to provide 80% and 90% power to detect varying relative reductions in the 30-day event rates for death and myocardial infarction between the two assigned treatment groups. Based on these estimates, we planned to enroll a sample of 3500 patients. If the rate of missing outcome data were ≤ 5%, the trial would have 3324 patients with analyzable outcome data at 30-days. Assuming an overall event

rate of 16.4%, the trial would have 80% power to detect a 20% relative reduction (i.e. 18.2% vs. 14.6%) and >90% power to detect a 25% relative reduction.

5. INTERIM MONITORING PLAN

The DSMB reviews interim analyses of the outcomes by assigned treatment group on an annual basis (from July 2018 through July 2022). The interim monitoring is designed to test for evidence of beneficial effect with either treatment strategy while maintaining the overall type I error at the pre-specified level. Stopping rules are based on the information (i.e. number of primary outcome events) that has accumulated by each inspection time and the shape of the predetermined alpha-spending function. The Lan-DeMets approach was used to allocate the type 1 error (i.e. alpha-level) at each interim time point and use of O'Brien Fleming monitoring boundaries. The DSMB will use the monitoring information to determine its recommendation to NHLBI. The DSMB may advise early termination of the trial for safety reasons or make other recommendations regarding modifications to the protocol. The final decision to stop the trial rests with the NHLBI. If the recommendation is to stop the trial, the MINT trial principal investigators are to be consulted before a final decision is made.

In order to ensure that the MINT trial has adequate power, an interim analysis to assess sample size was planned when approximately half of the participants are projected to have completed the 30-day follow-up. Revised sample size estimates were to be calculated based on the original power and effect size estimates from the trial hypothesis (80% power to detect a RR=0.80) and on the observed overall event rate (i.e. the two transfusion groups combined). The DSMB was to evaluate whether the trial sample size needs to be increased in order to restore power to maintain the ability to detect a clinically meaningful effect size. The DSMB reviewed this analysis at the January 2020 meeting. At that point, 1078 participants had finished 30-days follow-up plus an additional 6 months to ensure that all adjudication materials for the primary outcome were completed. Based on the results of this analysis, the DSMB voted to continue the MINT trial without any modification to the planned sample size (N=3500).

The original Data Safety Monitoring Plan stated that no interim futility analyses would be conducted since the MINT trial compares two established transfusion strategies with different resource and cost implications, and hence, a null result from a well-powered trial would be important for establishing treatment guidelines and policy. In February 2022, a futility analysis was requested by NHLBI. A conditional power plan was submitted to the NHLBI in April 2022 and was approved by a blinded statistical team at NHLBI in May 2022. The results will be presented to the DSMB at the July 2022 meeting.

The MINT interim monitoring plan includes a tiered approach for determining the continuation of the MINT trial. First, the efficacy monitoring that has occurred annually since the beginning of the trial will be performed to test if one arm is statistically superior to the other arm. Next, if superiority is not detected, then a futility analysis will occur. To determine futility, the conditional power for superiority of either arm with respect to the primary outcome will be performed assuming that the future trend is either consistent with the current trend (i.e. the maximum likelihood estimate), with the null hypothesis, with the alternative hypothesis favoring the restrictive strategy, and with the alternative hypothesis favoring the liberal strategy. If the conditional power of ANY of these tests is > 20%, then we recommend the study continue. If the conditional power of ALL of the specified tests for superiority are ≤ 20%, then we recommend moving to the non-inferiority testing. The conditional power for non-inferiority of the restrictive arm (compared to the liberal arm) will be performed assuming a relative 15% margin of non-inferiority (i.e. risk ratio for Restrictive versus Liberal < 1.15) and that the future trend is consistent with either the current trend (i.e. the maximum likelihood estimate), with the null hypothesis, with the alternative hypothesis favoring the restrictive strategy, and with the alternative hypothesis favoring the liberal strategy. If the conditional power of ANY of the non-inferiority conditional power tests is > 20%, then we recommend the study continue. If the conditional power of ALL the specified tests for non-inferiority are ≤ 20%, then termination of trial enrollment would be considered.

6. ANALYTIC APPROACH

CONSORT Chart

A CONSORT chart will be created as in Figure 1 to describe the flow of all participants who were consented to participate in the MINT trial through 180-days of follow-up.

Baseline Characteristics of the Enrolled Patient Sample

The baseline characteristics and co-interventions of the patients randomized in the trial will be described using frequencies, proportions, means and standard deviations, or medians and first and third quartiles. Characteristics will be presented for the entire sample and stratified by treatment assignment. Baseline characteristics will be compared by assigned treatment using chi-square statistics for categorical variables and t-tests or Wilcoxon rank-sum statistics for continuous variables. See Table 1.

Adherence to Assigned Intervention

The mean hemoglobin concentration at Baseline, 1, 2, and 3 days post-randomization will be compared graphically between the assigned treatment groups for all randomized patients (Figures 2a and 2b).

Linear mixed effect models will be used to compared the daily mean hemoglobin concentrations on day 1, 2 and 3 post-randomization by assigned treatment group adjusting for the baseline hemoglobin value and accounting for the repeated measures per participant using a random intercept.

$$\text{Post-rand Hgb}_{ij} = \beta_0 + \beta_1 \text{ Restrictive}_j + \beta_2 \text{ Day}_{jj} + \beta_3 \text{ Restrictive*Day}_{ij} + \beta_4 \text{ Baseline Hgb}_i + \alpha_i$$

where i=participant, j= day 1, 2 or 3; α_i is the random intercept for participant i

All patients with at least one non-missing in-hospital hemoglobin value on day 1, 2, or 3 will be included in the analysis. The estimated beta coefficients for Restrictive (versus Liberal) strategy and for the interaction between Restrictive strategy and Day will be presented along with their 95% confidence intervals and p-values.

The distribution of the number of red blood cell units transfused per participant will be described for each randomized treatment group using a histogram. The mean number of transfused units will be compared between assigned transfusion strategy groups using a simple Poisson test.

In addition, we will quantify the adherence to the protocol, by presenting the following among all randomized patients. For those assigned to Restrictive strategy, we will report the number (%) of randomized patients with at least one transfusion given when Hgb>8, and the number (%) of patients with at least one transfusion when no hemoglobin level was checked. Clinical reasons will be provided when possible. For those assigned to Liberal strategy, we will present the number (%) of randomized patients with no transfusion, and the number (%) of patients discharged with Hgb < 10. Clinical reasons will be provided when possible. Among all randomized patients, we will present the number (%) of patients with one and with two or more missing required hemoglobin measures, and number (%) of patients with one and with two or more missing troponin measures.

Trial Outcome Analyses

The intention-to-treat principle will be used to test the primary, secondary and tertiary aims comparing study outcomes by transfusion strategy. Two-sided tests will be used with an alpha-level=0.05 for all aims. No adjustment of the alpha level will be made for the secondary and tertiary analyses. Results will be interpreted based upon the observed findings, their designation as secondary or tertiary outcomes, and in comparison to the primary outcome result.

Primary Outcome Analysis

The primary outcome (the composite of all-cause mortality and myocardial infarction (death/MI) by 30 days from randomization) will be compared by assigned transfusion strategy using an unadjusted log-binomial regression model with a fixed effect variable for assigned treatment strategy and a random effect for clinical site and accounting for missing data using multiple imputation to impute missing 30-day outcome data.

$$Ln\,(p_{ij}) = \beta_0 + \beta_1\ Restrictive_{ij} + \alpha_i$$

where $p_{ij} = Prob(Y_{ij}=1|\ X_{ij})$, i=site, j= participant; α_i is the random intercept for site i

We will present the estimated risk ratio, 95% CI and p-value for the Restrictive Strategy versus the Liberal Strategy based on this model. Markov Chain Monte Carlo (MCMC) multiple imputation methods will be used to impute the missing 30-day outcome values (yes/no) for death and MI based on all available observed data (baseline and in-hospital variables). Ten imputed data sets will be created, a log-binomial model with random effects for site will be estimated for each imputed data set, and the results will be pooled to obtain a single estimate of treatment effect with an adjusted standard error. A log-binomial model with random effects for site will be created from patients with non-missing 30-day death/MI data as a sensitivity analysis. Also, if significant imbalances in baseline risk factors are detected between the two randomized treatment groups ($p<0.05$), a multivariable log-binomial regression model adjusting for the imbalanced factors will be created as a sensitivity analysis. If adherence to the protocol is lower than expected, a per protocol analysis, including only patients who undergo transfusion according to their assignment and adjusting for baseline factors that are associated with adherence to the treatment protocol, will be conducted as a sensitivity analysis.

For each of these models, the primary hypothesis test will be a two-sided test with alpha=0.05:

H_0: RR= 1.00 versus H_A: RR ≠ 1.00 using a two-sided alpha-level=0.05

such that RR = e^{β} where β is the coefficient for the Restrictive assigned strategy (versus Liberal assigned strategy) from the log-binomial model for the primary endpoint. If superiority of one of the two transfusion strategies is not demonstrated through the primary hypothesis test, a second hypothesis test of interest is the non-inferiority of the Restrictive Strategy compared to the Liberal strategy. Setting the non-inferior margin to a 15% relative increase, we will conduct the following one-sided non-inferiority hypothesis test with alpha=0.025:

H_0: RR ≥ 1.15 versus H_A: RR < 1.15 using a one-sided alpha-level=0.025.

With this test, rejecting the null hypothesis leads to the conclusion that the restrictive arm is non-inferior to the liberal arm. This is equivalent to the demonstrating that the entire 95% confidence interval for the estimated RR is < 1.15.

Among all patients with complete 30-day outcome data, the observed proportion of patients who experience the primary endpoint in each assigned group, the risk ratio (Restrictive versus Liberal) and 95% confidence interval, the risk difference (Restrictive – Liberal) and 95% confidence interval will be calculated and presented. See Table 2. We will determine whether this estimated 95% confidence interval for the RR includes 1.00 to assess superiority and whether it is <1.15 to assess non-inferiority of the Restrictive strategy.

Kaplan-Meier methods will be used to display the cumulative incidence of the primary endpoint over the 30-day post-randomization follow-up period for all randomized patients stratified by assigned treatment group (Figure 3a). The estimated risks at 30-days and the risk difference and 95% confidence interval at 30-days will be presented.

Analysis of Secondary and Tertiary Outcomes

All of the secondary outcomes and most of the tertiary outcomes are dichotomous endpoints (i.e. presence/absence of an event during the 30-day study period). The pre-defined secondary outcomes are: 1) all-cause mortality within 30 days; 2) myocardial reinfarction within 30 days; and 3) the composite outcome of all-cause mortality, nonfatal myocardial reinfarction, ischemia driven unscheduled coronary revascularization (percutaneous coronary intervention or coronary artery bypass grafting), or readmission to the hospital for ischemic cardiac diagnosis within 30 days. The dichotomous tertiary outcomes include: 1) all-cause mortality, nonfatal myocardial reinfarction, and unstable angina (i.e. acute coronary syndrome) 2) ischemia driven unscheduled coronary revascularization within 30-days; 3) readmission to the hospital for ischemic cardiac diagnosis, 4) congestive heart failure within 30 days; 5) unscheduled readmission to hospital for any reason within 30 days; 6) each of the individual cardiovascular outcomes of stroke, pulmonary embolism or deep venous thrombosis, and bleeding within 30 days; 7) each of the individual infectious outcomes of pneumonia, blood stream, and urinary tract within 30 days.

For each of the dichotomous secondary and tertiary endpoints listed above, the observed proportion of patients who experience the endpoint in each assigned group, the risk ratio (Restrictive versus Liberal) and 95% confidence interval, the risk difference (Restrictive – Liberal) and 95% confidence intervals will be calculated and presented (Table 2). Superiority of either transfusion strategy for each endpoint will first be evaluated using a two-sided test with alpha=0.05. If superiority is not detected, the non-inferiority

of the Restrictive strategy will be assessed using a 15% relative non-inferiority margin; that is, the Restrictive strategy will be deemed non-inferior for a given dichotomous endpoint when the entire 95% confidence interval for the estimated RR is < 1.15.

The length of hospital stay from randomization to discharge and the number of days in the ICU from randomization to discharge will be described as a mean (SD) or median (first and third quartile) in each assigned group and the difference will be analyzed using non-parametric Wilcoxon rank sum tests (Table 2).

The mean (SD) or median (first and third quartile) will be presented for the EQ-5D composite score and the EQ-5D Health Today score at 30 days post-randomization, and the difference will be tested based on t-tests or the Wilcoxon rank sum test depending on the distribution of the scores. The proportion of patients who report no problems with each of the 5 individual components of the EQ-5D (Mobility, Self-Care, Usual Activities, Pain/Discomfort, Anxiety/Depression) will be presented stratified by assigned treatment group (Table 2).

The cumulative risk of all-cause mortality through 180-day post-randomization will be estimated using Kaplan-Meier methods for all randomized patients stratified by assigned treatment group (Figure 3b). The estimated risk at 30 days and at 180 days for each treatment group will be presented, and a log-rank statistic will be used to test the hypothesis that the mortality risk over the 180-days is equivalent in the two assigned treatment groups. The estimated risk difference (Restrictive – Liberal) and 95% confidence interval will be presented for cumulative mortality risk at 30 days and at 180 days. If neither strategy is found to be superior based on the log rank test and the 95% confidence interval for the estimated risk difference (RD) at 180-days includes 1.0, the 95% confidence interval for the 180-day mortality risk difference will be assessed using a non-inferiority margin equal to an absolute difference of 3.0%. Thus, if the entire estimated 95% confidence for the 180-day mortality RD is <3.0%, the Restrictive strategy will be considered non-inferior to the Liberal Strategy.

Missing data will not be imputed for the analyses of the secondary and tertiary hypotheses unless critical issues are noted while investigating the missing 30-day primary outcome data.

Subgroup Analyses

Subgroup analyses will be performed based on the following baseline factors: ST segment elevation MI and non ST segment elevation MI, Universal Definition MI type (Type 1, Type 2), baseline hemoglobin level (<8, 8-8.9, 9-9.9 g/dL), revascularization for treatment of index MI prior to randomization (yes, no),

acute anemia (Acute Anemia defined as normal hemoglobin at admission, Possible Chronic Anemia defined as low hemoglobin at admission and no history of chronic anemia, and Probable Chronic Anemia defined as low hemoglobin at admission and history of chronic anemia), sex (male, female), age (<60, 60-69, 70-79, ≥80 years), heart failure defined as a history of congestive heart failure, congestive heart failure during the index hospitalization prior to randomization and/or left ventricular ejection fraction < 45% (yes, no), renal function (renal dialysis during index hospitalization prior to randomization, no dialysis and eGFR <30, no dialysis and eGFR 30-59, no dialysis and eGFR ≥ 60 $mL/mi/1.73m^2$) based on the CKD-EPI formula without race, and diabetes mellitus (diabetes mellitus treated with medication: yes, no). For participants from sites in the United States, Canada, New Zealand and Australia, we will also consider Race (White, Black, Non-White and Non-Black Race) and Hispanic ethnicity (yes, no). Participants from the European Union (EU) and Brazil are excluded from these analyses because race and ethnicity were not collected in the EU sites, and these concepts are challenging to define in Brazil.

The proportion of patients with a primary endpoint within 30 days will be reported for each assigned treatment group within each pre-defined subgroup. If subgroup categories are too small for meaningful inference, we will collapse or eliminate categories. The risk ratio (Restrictive / Liberal), 95% confidence interval and significance-level will be reported with a forest plot (Figures 4a and 4b). For each subgroup variable, a log-binomial regression model including subgroup variable, treatment assignment and the interaction between the subgroup variable and treatment assignment will be created, and the significance of the interaction term will be used to test whether the treatment effect is significantly modified by the designated subgroup variable.

$$Ln\,(p_i) = \beta_0 + \beta_1\ Subgroup_i + \beta_2\ Restrictive_{ij} + \beta_3\ Subgroup_i{*}Restrictive_i$$

where $p_i = Prob(Y_i=1|\ X_i)$, i= participant;

The focus of the subgroup analyses in the MINT trial is for descriptive purposes rather than on testing. When applicable, continuous versions of the subgroup variable will be considered for the regression models, and we will explore the existence of inflection points for treatment effectiveness. Subgroup analyses may be examined for secondary and/or tertiary outcomes utilizing similar methods. For 6-month mortality, Cox proportional hazards regression models will be created with the same covariates to test whether the effect of the randomized transfusion strategy for 6-month mortality varies significantly according to the pre-specified subgroup variables.

A number of secondary manuscripts will be undertaken to evaluate the effect of specified subgroup variables on treatment effectiveness in greater depth. These analyses will consider specified trial

endpoints and other clinical outcomes that are relevant to the research question. The analyses will also explore confounders and mediators that explain the relationships between the subgroup variable, transfusion treatment strategy, and outcomes.

Predictors of Clinical Outcomes and Other Planned Secondary Analyses

Multivariable regression models (linear, logistic and Cox regression) will be used to elucidate the roles of demographic and clinical factors as predictors of defined clinical and patient reported outcomes in this patient population. Outcomes of interest include the trial primary endpoint, the three secondary endpoints, congestive heart failure, unstable angina or unscheduled coronary revascularization, infection, health related quality of life as measured by the EuroQol-5D at 30-days, and six-month mortality.

We will also take a personalized medicine approach to characterize patients who benefit from a liberal transfusion strategy and patients who do as well, or better, with a restrictive transfusion strategy, with respect to the primary outcome and a few key secondary outcomes. Machine learning methods, including classification and regression trees (CART) and random forests, will be used to identify baseline characteristics that lead to better outcomes. We will use the Gini Index of variables in these models to identify the most important predictive factors and compare these to bivariate regression models of all factors to confirm associations with outcomes. The relationship between assigned treatment and these variables will be used in generated effect modifier (GEM) models to create functions of risk factors that interact with assigned treatment for a given outcome. Based on this function, data will be split in a ratio of 5:1 for training and testing to validate prediction of the treatment strategy from which each patient would benefit based on their clinical and demographic profile.

We will employ causal inference principles to assess the impact of various hemoglobin thresholds for transfusion on the primary outcome and 30-day death in the MINT trial. Using the observed trial data, we will emulate a parallel target trial with several different hemoglobin transfusion thresholds (< 10 g/dL, < 9 g/dL, < 8 g/dL and < 7 g/dL). All patients will be followed under each of the four transfusion strategies until the time they no longer adhere to that strategy based on the measured hemoglobin levels and transfusions administered. We will apply contemporary causal inference data analytic approaches aimed at minimizing biases to analyze the effect of treatment received (rather than assigned treatment) to determine the best hemoglobin threshold for this patient population.

7. TABLEs and FIGURES

Figure 1: CONSORT Chart

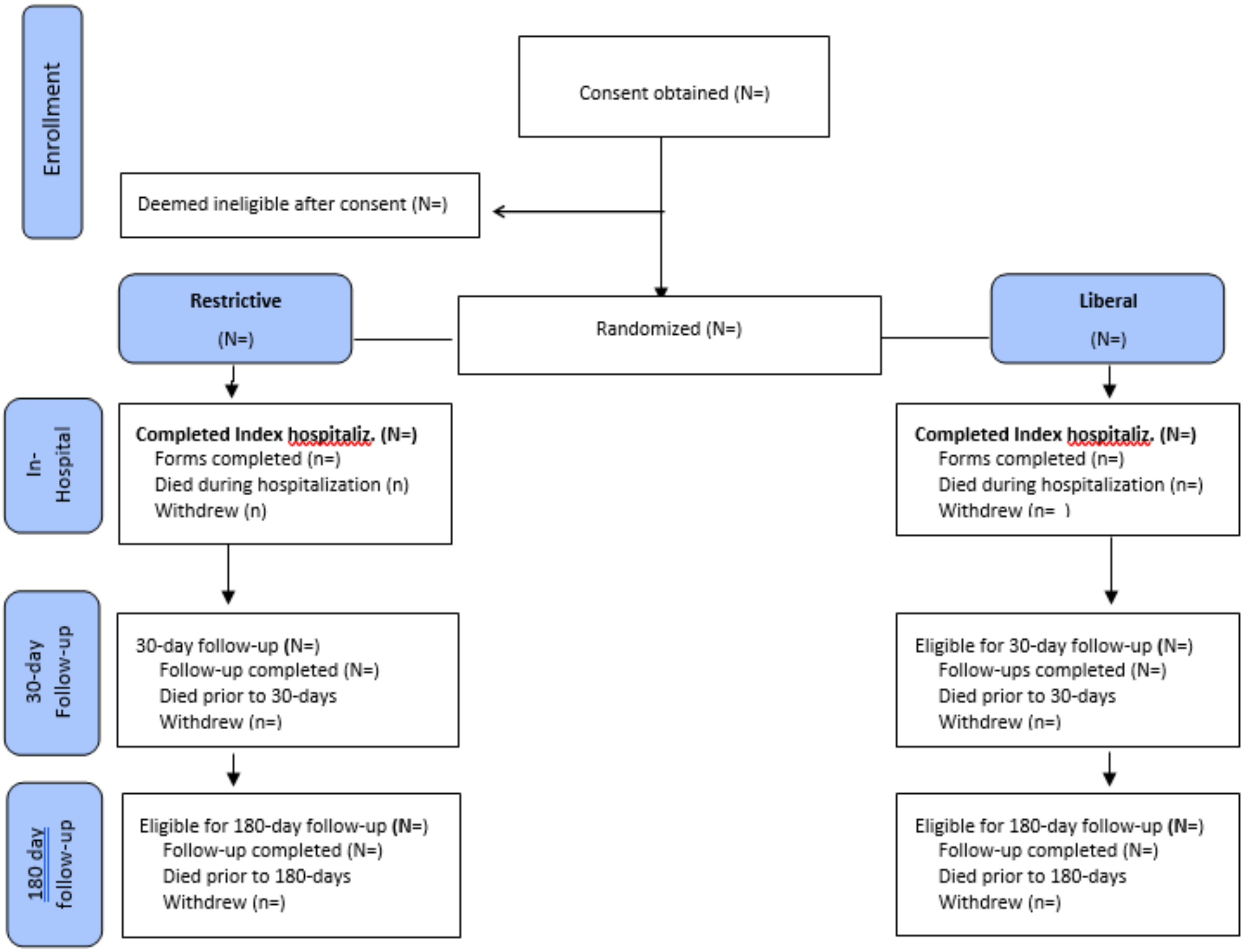

Table 1: Baseline Characteristics

Characteristic	All Participants (N=)	Restrictive (N=)	Liberal (N=)	p-value
Patient Characteristics				
Country, n (%)				
US				
Canada				
France				
Brazil				
New Zealand				
Australia				
Age, mean (SD)				
Male, n (%)				
Hispanic Latino or Latina, n (%)				
Race				
White or Caucasian, n (%)				
Black or African-American, n (%)				
Asian, n (%)				
American Indian or Alaska Native, n (%)				
Native Hawaiian or other Pacific Islander, n (%)				
First Nations Inuit or Metis, n (%)				
MultiRacial, n (%)				
Other Race, n (%)				
Unknown at this time, n (%)				
BMI, mean (SD)				
Tobacco smoker, n (%)				
Never				
Former				
Current				
Medical History				
MI, n (%)				
PCI, n (%)				
CABG, n (%)				
CHF, n (%)				
TIA, n (%)				
Atrial fibrillation, n (%)				
PAD, n (%)				
Renal failure/insufficiency, n (%)				
Diabetes, n (%)				
Hypertension, n (%)				
Hypercholesterolemia/hyperlipidemia, n (%)				
COPD/asthma, n (%)				
Cancer, n (%)				
Anemia, n (%)				
Documented diagnosis of COVID-19, n (%)				
Angiogram results, n (%)				
Number of vessels with > 50% obstruction, n (%)				
0				
1				

Characteristic	All Participants (N=)	Restrictive (N=)	Liberal (N=)	p-value
2				
3				
Left main disease, n (%)				
LVEF results available, n (%)				
Most recent LV ejection fraction (%) within the past year, mean (SD)				
LV ejection fraction: Severity level, n (%)				
55% or greater (normal)				
45% to less than 55% (mild)				
30% to less than 45% (moderate)				
less than 30% (severe)				
Index Myocardial Infarction (MI)				
MI classification, n (%)				
Type 1				
Type 2				
Type 4b				
Type 4c				
Unknown				
Symptoms of ischemia, n (%)				
New ST-T changes or new left bundle branch block, n (%)				
Development of pathological Q waves, n (%)				
New loss of viable myocardium or regional wall motion abnormality, n (%)				
Identification of an intracoronary thrombus, n (%)				
Chest pain, n (%)				
Symptoms related to onset of index MI				
Jaw pain, n (%)				
Back pain, n (%)				
Shortness of breath, n (%)				
Epigastric pain, n (%)				
Palpitations, n (%)				
Left arm pain, n (%)				
Syncope, n (%)				
Other symptoms related to MI, n (%)				
Diagnosis of MI at time of randomization confirmed as MI at time of discharge, n (%)				
Events during index hospitalization prior to randomization				
PCI occurred prior to randomization, n (%)				
CABG occurred prior to randomization, n (%)				
Revascularization post-MI and prior to randomization, n (%)				
CHF prior to randomization, n (%)				
History of CHF, CHF during index hospitalization prior to randomization and/or LVEF < 45%, n (%)				
Renal dialysis prior to randomization, n (%)				
Intubated on ventilator prior to randomization, n (%)				
Active bleeding prior to randomization, n (%)				
Received RBC transfusion prior to randomization, n (%)				
Number of units, mean (SD)				

Characteristic	All Participants (N=)	Restrictive (N=)	Liberal (N=)	p-value
Laboratory Measures most recent prior to randomization				
Hemoglobin: Level (g/dL), mean (SD)				
Hemoglobin: Level (g/dL), n (%)				
<8.0				
8.0-8.9				
9.0-9.9				
Acute Anemia				
Normal hemoglobin at admission				
Low hemoglobin at admission and no history of chronic anemia				
Low hemoglobin at admission and history of chronic anemia				
Most recent creatinine value (mg/dL), mean (SD)				
eGFR (mL/mi/1.73m^2), mean (SD)				
Renal function, n (%)				
Renal dialysis during hospitalization pre-randomization				
<30 mL/mi/1.73m^2 and no dialysis				
30-59 mL/mi/1.73m^2 and no dialysis				
≥60 mL/mi/1.73m^2 and no dialysis				
Location at time of randomization				
In ICU/CCU at randomization, n (%)				

Figure 2a: Post-randomization Transfusions during Index Hospitalization

Histogram of number of units transfused as part of the protocol for all randomized patients stratified by assigned treatment.

Figure 2b: Post-randomization Hemoglobin by Assigned Strategy

Line plot of mean hemoglobin at Baseline, Day1, Day 2 and Day 3 for all randomized patients stratified by assigned treatment.

Table 2: MINT Primary, Secondary and Tertiary Outcomes

	Cumulative Events at 30 days (Patient-Level)				In-Hospital Events		Hospital Discharge to 30 days Events	
**Characteristic**	**Restrictive (N=)**	**Liberal (N=)**	**Risk Ratio* (95% CI)**	**p-value**	**Restrictive (N=)**	**Liberal (N=)**	**Restrictive (N=)**	**Liberal (N=)**
Primary Outcome (at 30 days)								
All-cause mortality/adjudicated non-fatal myocardial infarction (MI), n (%)								
Secondary Outcome (at 30 days)								
All-cause mortality, n (%)								
Adjudicated myocardial infarction, n (%)								
All-cause mortality, adjudicated non-fatal MI, ischemia driven unscheduled revascularization, unscheduled readmission to the hospital for ischemic cardiac diagnosis, n (%)								
Tertiary Outcome (at 30 days unless noted)								
All-cause mortality, adjudicated non-fatal MI, unstable angina, n (%)								
Ischemia driven unscheduled revascularization, n (%)								
Unscheduled readmission to the hospital for ischemic cardiac diagnosis, n (%)								
Any Indication of CHF, n (%)								
Unscheduled readmission to hospital for any reason, n (%)								
Stroke, n (%)								
Pulmonary Embolism (PE), n (%)								
Deep Vein Thrombosis (DVT), n (%)								
Bleeding event, n (%)								
Pneumonia, n (%)								
Bacteremia, n (%)								
Urinary tract infection, n (%)								

** Risk Ratio = Restrictive / Liberal for event outcomes*

Table 3: MINT 30-day Tertiary Outcomes

	Restrictive (N=)	Liberal (N=)	Mean Difference* (95% CI)	p-value
Tertiary Outcome (continued)				
Length of hospital stay, number of days, mean (sd)				
Length of stay in the ICU, number of days, mean (sd)				
**EuroQol 5D at 30-days, N**				
EuroQol 5D Score, mean (SD)				
EQ-5D Health Today, mean (SD)				
No problems identified with:	**Restrictive (N=)**	**Liberal (N=)**	**Risk Ratio* (95% CI)**	**p-value**
EQ-5D Mobility, n (%)				
EQ-5D Self-Care, n (%)				
EQ-5D Usual Activities, n (%)				
EQ-5D Pain/Discomfort, n (%)				
EQ-5D Anxiety/Depression, n (%)				

Mean difference = Restrictive - Liberal

Figure 3a: 30-day Cumulative Incidence Curves for All-Cause Death / Adjudicated MI
Cumulative incidence using Kaplan Meier methods stratified by assigned treatment with estimates at 30-days presented

Figure 3b: 180-day Cumulative Incidence Curves for All-Cause Death
Cumulative incidence using Kaplan Meier methods stratified by assigned treatment with estimates at 30-days and at 180-days presented.

Figure 4a: 30-day Death/MI by Assigned Transfusion Strategy and Predefined Subgroups

**All Randomized Participants: Subgroups**	**Subgroup Sample Size**	**Restrictive (N=)**	**Liberal (N=)**	**Risk Ratio**	**95% CI**	**FIGURE RR* (95% CI)**	**Interaction p-value**
All Participants	N(%)	n (%)	n (%)	RR	LL - UL	**---------X----------**	
MI Category							p-value
STEMI	N(%)	n (%)	n (%)	RR	LL - UL	**---------X----------**	
non STEMI	N(%)	n (%)	n (%)	RR	LL - UL	**---------X--------**	
MI Type							p-value
Type 1 MI	N(%)	n (%)	n (%)	RR	LL - UL	**---------X----------**	
Type 2 MI	N(%)	n (%)	n (%)	RR	LL - UL	**---------X--------**	
Baseline hemoglobin level							p-value
<8 g/dL	N(%)	n (%)	n (%)	RR	LL - UL	**---------X----------**	
8-8.9 g/dL	N(%)	n (%)	n (%)	RR	LL - UL	**---------X--------**	
9-9.9 g/dL	N(%)	n (%)	n (%)	RR	LL - UL	**------X-------**	
Revascularization post-MI and pre-randomization*							p-value
Yes	N(%)	n (%)	n (%)	RR	LL - UL	**---------X----------**	
No	N(%)	n (%)	n (%)	RR	LL - UL	**---------X--------**	
Acute Anemia							p-value
Acute Anemia	N(%)	n (%)	n (%)	RR	LL - UL	**------X-------**	
Possible Chronic Anemia	N(%)	n (%)	n (%)	RR	LL - UL	**---------X----------**	
Probably Chronic Anemia	N(%)	n (%)	n (%)	RR	LL - UL	**---------X--------**	
Sex							
Male							
Female							
Age							
< 60 years							
60-69 years							
70-79 years							
≥ 80 years							
Heart Failure							
Yes							
No							
Renal Function							
Renal dialysis							
< 30 mL/mi/1,73m^2							
30-59 mL/mi/1,73m^2							
≥69 mL/mi/1,73m^2							
Diabetes Mellitus (treated)							
Yes							
No							

N (%) indicates the sample size of the subgroup and the proportion of the total sample in that subgroup.

n (%) indicates the number of outcome events in the specified treatment group and subgroup, and the proportion of people in the specified treatment group and subgroup who experienced the event.

RR: Rate Ratio = Restrictive / Liberal

* the No Revascularization group includes XX who received revascularization between randomization and hospital discharge YY of whom had a revascularization that was planned prior to randomization and was not based on new signs or symptoms consistent with ischemia that occurred post-randomization.

Figure 4b: 30-day Death/MI by Assigned Transfusion Strategy and Predefined Subgroups

**U.S., Canada, New Zealand and Australia Participants: Subgroups**	**Subgroup Sample Size**	**Restrictive (N=)**	**Liberal (N=)**	**Risk Ratio**	**95% CI**	**FIGURE RR* (95% CI)**	**Interaction p-value**
U.S., Canada, New Zealand and Australia Participants	N(%)	n (%)	n (%)	RR	LL - UL	---------X----------	
Race							p-value
White	N(%)	n (%)	n (%)	RR	LL - UL	---------X----------	
Black	N(%)	n (%)	n (%)	RR	LL - UL	---------X----------	
non-White & non-Black	N(%)	n (%)	n (%)	RR	LL - UL	---------X----------	
Hispanic							p-value
Yes	N(%)	n (%)	n (%)	RR	LL - UL	---------X----------	
No	N(%)	n (%)	n (%)	RR	LL - UL	---------X----------	

N (%) indicates the sample size of the subgroup and the proportion of the total sample in that subgroup.

n (%) indicates the number of outcome events in the specified treatment group and subgroup, and the proportion of people in the specified treatment group and subgroup who experienced the event.

RR: Rate Ratio = Restrictive / Liberal

Supplementary Information: In-Hospital and 30-day Outcomes and Adverse Events

	Cumulative Rate of Event at 30 days (Patient-Level)		In-Hospital Events		Hospital Discharge to 30 days Events	
**Characteristic**	**Restrictive (N=)**	**Liberal (N=)**	**Restrictive (N=)**	**Liberal (N=)**	**Restrictive (N=)**	**Liberal (N=)**
Outcome Events						
Cardiac Mortality, n (%)						
New suspected MI, n (%)						
Procedures: PCI, n (%)						
Ischemia driven unscheduled PCI, n (%)						
Procedures: CABG, n (%)						
Ischemia driven unscheduled CABG, n (%)						
New unstable angina, n (%)						
Transient Ischemic Attack (TIA), n (%)						
Strict Definition of CHF, n (%)						
Any Indication of TACO, n (%)						
Strict Definition of TACO, n (%)						
Arrhythmias						
Atrial fibrillation/flutter, n (%)						
Supraventricular tachycardias other than atrial fibrillation, n (%)						
Paroxysmal supraventricular tachycardia, n (%)						
Ventricular tachycardia, n (%)						
Ventricular fibrillation, n (%)						
Cardiac arrest, n (%)						
Mobitz type 1 AV block, n (%)						
Mobitz type 2 AV block, n (%)						
Complete (3rd degree) AV block, n (%)						
Right bundle branch block, n (%)						
Left bundle branch block, n (%)						
Bifascicular block, n (%)						
Asystole, n (%)						
Multifocal atrial tachycardia, n (%)						
Nonparoxysmal junctional tachycardia, n (%)						
Thrombotic/Ischemic Events and Complications of MI						
Chest pain or stable angina, n (%)						
Mechanical complications of MI, n (%)						
Pericarditis, n (%)						
Pericardial effusion/tamponade, n (%)						
Syncope, n (%)						
Cardiomyopathy, n (%)						
Other Adverse Events						
Acute respiratory failure, n (%)						
Acute renal failure, n (%)						
Urinary tract infection, n (%)						
Transfusion Reactions						
Transfusion related acute lung injury, n (%)						
Acute hemolytic transfusion reaction, n (%)						

Characteristic	Cumulative Rate of Event at 30 days (Patient-Level)		In-Hospital Events		Hospital Discharge to 30 days Events	
	Restrictive (N=)	Liberal (N=)	Restrictive (N=)	Liberal (N=)	Restrictive (N=)	Liberal (N=)
Transfusion associated sepsis, n (%)						
Anaphylactic transfusion reaction, n (%)						
Urticarial transfusion reaction, n (%)						
Febrile non-hemolytic reaction, n (%)						

[2]Strict Definition of CHF: Defined as having all of the following: **Symptoms** (SOB, orthopnea, paroxysmal nocturnal dyspnea, edema), **signs** (New or worsening development of pulmonary rales, S3 gallop, Pulmonary congestion/pleural effusions on chest X-ray, abnormal elevated BNP/pro-BNP) **and treatment** (new use/increased dose of diuretics, requirement for inotropes or vasoactive drugs, assisted ventilator support) and did not receive a transfusion within 6 hours of onset of CHF.

[3]Any Indication of TACO: Defined as having any of the following: **Symptoms** (SOB, orthopnea, paroxysmal nocturnal dyspnea, edema), **signs** (New or worsening development of pulmonary rales, S3 gallop, Pulmonary congestion/pleural effusions on chest X-ray, abnormal elevated BNP/pro-BNP) **or treatment** (new use/increased dose of diuretics, requirement for inotropes or vasoactive drugs, assisted ventilator support) and received a transfusion within 6 hours of onset of CHF.

[4]A Strict definition of TACO: Defined as having all of the following: **Symptoms** (SOB, orthopnea, paroxysmal nocturnal dyspnea, edema), **signs** (New or worsening development of pulmonary rales, S3 gallop, Pulmonary congestion/pleural effusions on chest X-ray, abnormal elevated BNP/pro-BNP) **and treatment** (new use/increased dose of diuretics, requirement for inotropes or vasoactive drugs, assisted ventilator support) and received a transfusion within 6 hours of onset of CHF.

Supplementary 30-day Information

Characteristic	Restrictive (N=)	Liberal (N=)
Number of ER visits, n (%)		
0		
1		
2		
3+		
Number of ER visits, mean (SD)		
Number of hospital readmissions, n (%)		
0		
1		
2		
3		
Number of hospital readmissions, mean (SD)		

The Statistical Analysis Plan for the Myocardial Ischemia and Transfusion (MINT) Trial

July 24, 2023

INTRODUCTION

This Statistical Analysis Plan for the Myocardial Ischemia and Transfusion (MINT) trial is consistent with and expands upon the statistical methods outlined in the trial protocol. This document includes 1) a brief summary of the MINT trial design, 2) the trial aims, 3) definitions of the specified MINT trial outcomes, 4) the original sample size and power calculations, 5) the interim monitoring plan, 6) analytic approach, and 7) proposed template tables and figures.

1. TRIAL DESIGN

Study Design

The MINT trial is a randomized, open-label, multicenter clinical trial designed to determine whether a liberal transfusion strategy with a threshold of 10 g/dL reduces the composite outcome of all-cause mortality or nonfatal myocardial reinfarction through 30 days, compared with a restrictive transfusion strategy with a threshold of 7 to 8 g/dL among patients with an acute myocardial infarction and a hemoglobin concentration less than 10 g/dL. The trial is conducted in approximately 144 clinical sites in the United States, Canada, France, Brazil, New Zealand, and Australia. The two transfusion strategies are allocated with a 1:1 ratio using a permuted block design, with random variable block sizes, stratified by clinical site.

Population

The eligible study population includes adult patients with ST-segment elevation myocardial infarction or non ST-segment elevation myocardial infarction consistent with the 3rd Universal Definition of Myocardial Infarction criteria[1] that occurs on admission or during the index hospitalization, and anemia defined as a hemoglobin concentration less than 10 g/dL at the time of randomization. Patients with Type 1, Type 2,

Type 4b, and Type 4c are eligible, and the occurrence of the index MI is determined by the enrolling site study team.

Trial Transfusion Strategies

The trial compares a restrictive and a liberal approach to transfusion therapy. The transfusion strategy is followed during the index hospitalization from the time of randomization up to 30-days post-randomization, hospital discharge, or death, whichever comes first.

Restrictive Transfusion Strategy: Patients randomized to the restrictive transfusion strategy may receive a transfusion if the hemoglobin concentration falls below 8 g/dL and are strongly encouraged to receive transfusion if the hemoglobin concentration is below 7 g/dL. Transfusion is also allowed when anginal symptoms are determined by the patient's treating physician to be related to anemia and are not controlled with anti-anginal medications. Enough blood is given to increase the hemoglobin concentration to above 7 to 8 g/dL or to relieve anginal symptoms. A post transfusion hemoglobin measurement is required.

Liberal Transfusion Strategy: Patients randomly allocated to the liberal transfusion strategy receive one unit of packed RBCs following randomization and will receive enough blood to raise the hemoglobin concentration to 10 g/dL or above any time during the index hospitalization that the hemoglobin concentration is detected to be below 10 g/dL. A post transfusion hemoglobin measurement showing a hemoglobin level of at least 10 g/dL must be obtained.

For both strategies, blood is administered one unit at a time followed by a hemoglobin measurement. A patient in either group may be transfused at any time without a hemoglobin level if the patient is actively bleeding (e.g., brisk gastrointestinal bleeding) and the treating physician believes an emergency transfusion is needed. A patient in either group with history of congestive heart failure or low ejection fraction may receive diuretics prior to or after transfusion and transfusion may be delayed until the patient can safely tolerate the additional volume. Patients with end stage renal disease may receive transfusion during dialysis if requested by treating physician. The transfusion protocol is suspended for 24 hours if the patient goes to surgery.

Assessments of laboratory markers

The trial collects hemoglobin and troponin levels and electrocardiogram (ECG) readings at specified time points while the patient is in the hospital. The required collection time points for hemoglobin levels and

ECGs are within 24 hours prior to randomization, and on days 1, 2 and 3 post-randomization. The required time points for the troponin levels are within 24 hours prior to randomization and post randomization, at 12 hours, and on days 1, 2 and 3. All hemoglobin and troponin levels performed for during the index hospitalization are collected by the trial.

Participant Follow-up

At hospital discharge, death or 30 days post randomization, whichever comes first, the study staff submit data related to the hospitalization including health status, laboratory results, blood transfusions, and post randomization clinical events. Study staff contact the patient at 30 days post-randomization to ascertain vital status, administer the quality-of-life questionnaire, and determine if there has been a subsequent hospital admission or emergency room visit. Study staff obtain and review the medical records for each readmission and record relevant data and study outcomes. If an acute coronary syndrome event is suspected from the time of randomization until 30-days post-randomization, the staff submits documentation for central review. Study staff contact participants at 6 months following randomization to ascertain the patient's vital status.

Recurrent myocardial infarction is diagnosed when a suspected myocardial ischemic event is reported by the investigators at the clinical sites and confirmed by the Clinical Event Committee. In addition to adjudication of site suspected events, the trial has a surveillance process in which all cardiac troponin values are reviewed by the Clinical Event Committee to detect abnormal biomarker patterns. When criteria are met, the Clinical Event Committee requests that the site submit medical records and ECGs that are temporally related to the abnormal values. The Clinical Events Committee adjudicates occurrences of myocardial infarction, masked to assigned transfusion strategy, from randomization through 30-days post-randomization. Myocardial infarction is classified according to MI type as specified in the 3rd Universal Definition of Myocardial Infarction[1] and whether MI type was ST segment elevation MI (STEMI) non-ST segment elevation MI (NSTEMI), or cannot be determined.

2. TRIAL AIMS

Primary Aim

The MINT primary aim is to determine whether a liberal transfusion strategy with a threshold of 10 g/dL reduces the composite outcome of all-cause mortality or nonfatal myocardial reinfarction through 30 days following randomization, compared to a restrictive transfusion strategy with a threshold of 7 to 8 g/dL among patients with an acute myocardial infarction and a hemoglobin concentration less than 10 g/dL.

Secondary Aims

1) To determine whether a liberal (10g/dL) transfusion strategy reduces all-cause mortality within 30 days, compared to a restrictive transfusion strategy.

2) To determine whether a liberal (10g/dL) transfusion strategy reduces myocardial reinfarction within 30 days, compared to a restrictive transfusion strategy.

3) To determine whether a liberal (10g/dL) transfusion strategy reduces the composite outcome of all-cause mortality, nonfatal myocardial reinfarction, ischemia driven unscheduled coronary revascularization (percutaneous coronary intervention or coronary artery bypass grafting), or readmission to the hospital for ischemic cardiac diagnosis within 30 days, compared to a restrictive transfusion strategy.

Tertiary Aims

1) To determine whether a liberal (10g/dL) transfusion strategy reduces all-cause mortality, nonfatal myocardial reinfarction, or unstable angina (i.e. acute coronary syndrome) within 30 days, compared to a restrictive transfusion strategy.

2) To determine whether a liberal (10g/dL) transfusion strategy reduces ischemia driven unscheduled coronary revascularization within 30-days compared to a restrictive strategy.

3) To determine whether a liberal (10g/dL) transfusion strategy reduces unscheduled readmission to hospital for ischemic cardiac diagnosis within 30 days, compared to a restrictive strategy.

4) To determine whether a liberal (10g/dL) transfusion strategy increases congestive heart failure within 30 days, compared to a restrictive transfusion strategy.

5) To determine whether a liberal (10g/dL) transfusion strategy reduces unscheduled readmission to hospital for any reason within 30 days, compared to a restrictive strategy.

6) To determine whether a liberal (10g/dL) transfusion strategy increases each of the individual thrombotic/hemorrhagic outcomes of stroke, pulmonary embolism or deep venous thrombosis, and bleeding within 30 days, compared to a restrictive strategy.

7) To determine whether a liberal (10g/dL) transfusion strategy increases each of the individual infectious outcomes of pneumonia, blood stream, and urinary tract within 30 days, compared to a restrictive strategy.

8) To determine whether a liberal (10g/dL) transfusion strategy reduces each of the individual in-hospital outcomes of length of hospital stay post randomization and number of days in intensive care unit, compared to a restrictive strategy.

9) To determine whether a liberal (10g/dL) transfusion strategy increases patient reported quality of life using the EuroQol questionnaire (EQ-5D) at 30 days compared to a restrictive strategy

10 To determine whether a liberal (10g/dL) transfusion strategy reduces all-cause mortality at 6-months following randomization, compared to a restrictive strategy.

3. DEFINITIONS OF OUTCOMES

Myocardial Reinfarction: Myocardial reinfarction will be classified by the Clinical Events Committee using 3rd Universal Definition of MI definition.[1] Patients with reinfarction will need to demonstrate a fall in the troponin value and then a subsequent rise of at least 20% with additional evidence (new ECG changes, imaging evidence, clinical history) as in the MI definition to diagnose a new event. Myocardial infarction is classified according to MI type[1] and whether MI type was STEMI, NSTEMI, or cannot be determined.

Death and Cause of Death: For each death, the cause will be determined by the site personnel into one of three categories: cardiac death (e.g., congestive heart failure, dysrhythmia), noncardiac death (e.g., infection, cancer), or undetermined cause of death. Information about the specific cause of death will also be collected.

Unscheduled Coronary Revascularization (unstaged): Ischemia driven, unscheduled coronary revascularization (coronary artery bypass surgery or PCI) within 30 days of randomization will be recorded by the sites. Prior to randomization, the site will record if a coronary revascularization is planned (staged). All coronary revascularization procedures will be recorded, but an elective planned staged procedure will not be included as an outcome. Information about the reason for the procedure will also be collected to ensure that the revascularization was done to treat ischemic heart disease.

Readmission to Hospital, Overall and for Primary Cardiac Diagnosis: All re-admissions to the hospital that had not been planned prior to randomization will be captured, and the primary diagnosis for each

hospitalization will be classified as: ischemic cardiac diagnosis (e.g., myocardial infarction, unstable angina), non-ischemic cardiac diagnosis (e.g. heart failure) or non-cardiac.

Unstable Angina: The MINT trial sites will use 2014 ACC/AHA Key Data Elements and Definitions for Cardiovascular Endpoint Events in Clinical Trials[2] to define unstable angina. To diagnose unstable angina requires that four criteria be met: 1) worsening ischemic discomfort, 2) unscheduled hospitalization, 3) negative cardiac biomarker, 4) objective evidence of myocardial ischemia.

Congestive Heart Failure: Sites personnel will use 2014 ACC/AHA Key Data Elements and Definitions for Cardiovascular Endpoint Events in Clinical Trials[2] to define congestive heart failure. New or worsening symptoms of congestive heart failure on presentation (increasing dyspnea, paroxysmal nocturnal dyspnea, orthopnea), has objective evidence of new or worsening heart failure, and receives initiation or intensification of treatment specifically for heart failure.

TIA or Stroke: The 2014 ACC/AHA Key Data Elements and Definitions for Cardiovascular Endpoint Events in Clinical Trials[2] will be used to define stroke. A transient ischemic attack (TIA) is defined as "a transient episode of focal neurological dysfunction caused by brain, spinal cord, or retinal ischemia, without acute infarction." Stroke is defined on the basis of the presence of acute infarction as demonstrated by imaging or based on the persistence of symptoms more than 24 hours.

Deep Venous Thrombosis or Pulmonary Embolism: Deep vein thrombosis will be diagnosed if duplex ultrasound, magnetic resonance venogram (MRV), or venogram is definite or probable positive. Site investigators will record if location is proximal or distal. Pulmonary embolism will be diagnosed with a high probability ventilation perfusion lung scan, CT scan, or pulmonary angiogram.

Bleeding: Major bleeding will be defined as 1) fatal bleeding, and/or 2) symptomatic bleeding in a critical area or organ, such as intracranial, intraspinal, intraocular, retroperitoneal, intra- articular or pericardial, or intramuscular with compartment syndrome, and/or 3) bleeding causing a drop in hemoglobin concentration of 2 g/dL or greater[3] from the last hemoglobin concentration prior to randomization to the nadir hemoglobin concentration during hospitalization or up to 30 days post randomization. The drop in

hemoglobin concentration will account for each unit of red blood cell transfusion transfused by subtracting 1 g/dL for each unit administered.

Infections recorded include pneumonia and blood stream infections.

Pneumonia: Pneumonia will be diagnosed using CDC criteria[4] which includes radiographic abnormalities and combination of symptoms (i.e., cough), signs (i.e., fever, tachypnea, or laboratory abnormalities (i.e., white blood cell count, hypoxemia).

Blood Stream Infection: Blood stream infection will be defined using CDC criteria[4] which includes a recognized pathogen cultured from 1 or more blood cultures and organism cultured from blood is not related to an infection at another site, at least 1 of the following signs or symptoms: fever, chills, or hypotension and signs and symptoms and positive laboratory results are not related to an infection at another site and common skin contaminant is cultured from 2 or more blood cultures drawn on separate occasions.

Length of Stay and Intensive Care Unit Days: The trial records the number of days post randomization that the patient is in the hospital and in intensive care unit.

Quality of Life: The EuroQol questionnaire (EQ-5D),[5] a standardized instrument that measures health related quality of life in 5 dimensions: mobility, self-care, usual activities, pain/discomfort and anxiety/depression, is used as a measure of patient perceived global health status 30 days after randomization. The EQ-5D utility index value[6] and heath today[5] scores will be reported.

4. SAMPLE SIZE DETERMINATION AND POWER

In the MINT pilot trial, the composite 30-day rate of death and myocardial infarction was 16.4%. Using a two-sided inequality test and a simple chi-square statistic with alpha=0.05, we determined the samples sizes required to provide 80% and 90% power to detect varying relative reductions in the 30-day event rates for death and myocardial infarction between the two assigned treatment groups. Based on these estimates, we planned to enroll a sample of 3500 patients. If the rate of missing outcome data were ≤ 5%, the trial would have 3324 patients with analyzable outcome data at 30-days. Assuming an overall event rate of 16.4%, the trial would have 80% power to detect a 20% relative reduction (i.e. 18.2% vs. 14.6%) and >90% power to detect a 25% relative reduction.

5. INTERIM MONITORING PLAN

The DSMB reviews interim analyses of the outcomes by assigned treatment group on an annual basis (from July 2018 through July 2022). The interim monitoring is designed to test for evidence of beneficial effect with either treatment strategy while maintaining the overall type I error at the pre-specified level. Stopping rules are based on the information (i.e. number of primary outcome events that have accumulated) at each inspection time and the shape of the predetermined alpha-spending function. The Lan-DeMets approach was used to allocate the type 1 error (i.e. alpha-level) at each interim time point and use of O'Brien Fleming monitoring boundaries. The DSMB will use the monitoring information to determine its recommendation to NHLBI. The DSMB may advise early termination of the trial for safety reasons or make other recommendations regarding modifications to the protocol. The final decision to stop the trial rests with the NHLBI. If the recommendation is to stop the trial, the MINT trial principal investigators are to be consulted before a final decision is made.

In order to ensure that the MINT trial has adequate power, an interim analysis to assess sample size was planned when approximately half of the participants are projected to have completed the 30-day follow-up. Revised sample size estimates were to be calculated based on the original power and effect size estimates from the trial hypothesis (80% power to detect a RR=0.80) and on the observed overall event rate (i.e. the two transfusion groups combined). The DSMB was to evaluate whether the trial sample size needs to be increased in order to restore power to maintain the ability to detect a clinically meaningful effect size. The DSMB reviewed this analysis at the January 2020 meeting. At that point, 1078 participants had finished 30-days follow-up plus an additional 6 months to ensure that all adjudication materials for the primary outcome were completed. Based on the results of this analysis, the DSMB voted to continue the MINT trial without any modification to the planned sample size (N=3500).

The original Data Safety Monitoring Plan stated that no interim futility analyses would be conducted since the MINT trial compares two established transfusion strategies with different resource and cost implications, and hence, a null result from a well-powered trial would be important for establishing treatment guidelines and policy. The study team submitted an application for additional trial funding to the NHLBI; in response, the NHLBI requested a futility analysis in February 2022. The study team submitted a conditional power plan in April 2022, and this plan was approved by a blinded statistical team at NHLBI in May 2022. The plan and results were presented to the DSMB on July 22, 2022.

The MINT interim monitoring plan included a tiered approach for determining the continuation of the MINT trial. First, the efficacy monitoring that has occurred annually since the beginning of the trial will be

performed to test if one arm is statistically superior to the other arm. Next, if superiority is not detected, then a futility analysis will occur. To determine futility, the conditional power for superiority of either arm with respect to the primary outcome will be performed assuming that the future trend is either consistent with the current trend (i.e. the maximum likelihood estimate), with the null hypothesis, with the alternative hypothesis favoring the restrictive strategy, and with the alternative hypothesis favoring the liberal strategy. If the conditional power of ANY of these tests is > 20%, then we recommend the study continue. If the conditional power of ALL of the specified tests for superiority are ≤ 20%, then we recommend moving to the non-inferiority testing. The conditional power for non-inferiority of the restrictive arm (compared to the liberal arm) will be performed assuming a relative 15% margin of non-inferiority (i.e. risk ratio for Restrictive versus Liberal < 1.15) and that the future trend is consistent with either the current trend (i.e. the maximum likelihood estimate), with the null hypothesis, with the alternative hypothesis favoring the restrictive strategy, and with the alternative hypothesis favoring the liberal strategy. If the conditional power of ANY of the non-inferiority conditional power tests is > 20%, then we recommend the study continue. If the conditional power of ALL the specified tests for non-inferiority are ≤ 20%, then termination of trial enrollment would be considered. Based on the results of this analysis, the DSMB voted that the MINT trial continue to the planned sample size (N=3500).

6. ANALYTIC APPROACH

CONSORT Chart

A CONSORT chart will be created to describe the flow of all participants who were consented to participate in the MINT trial through 180-days of follow-up. See Figure 1 as an example in the Supplement to this document.

Baseline Characteristics of the Enrolled Patient Sample

The baseline characteristics and co-interventions of the patients randomized in the trial will be described using frequencies, proportions, means and standard deviations, or medians and first and third quartiles. Characteristics will be presented for the entire sample and stratified by treatment assignment. See Table 1 in the Supplement for an example.

Adherence to Assigned Intervention

The distribution of the number of red blood cell units transfused per participant will be described for each randomized treatment group (e.g. Figure 2a). The counts and mean number of units per participant as

well as the total number of transfused units will be described, and the mean number of transfused units will be compared between assigned transfusion strategy groups using a simple Poisson test.

The mean hemoglobin concentration at Baseline, 1, 2, and 3 days post-randomization will be compared graphically between the assigned treatment groups for all randomized patients (e.g. Figure 2b). Linear mixed effect models will be used to compared the daily mean hemoglobin concentrations on day 1, 2 and 3 post-randomization by assigned treatment group adjusting for the baseline hemoglobin value and accounting for the repeated measures per participant using a random intercept.

$$\text{Post-rand Hgb}_{ij} = \beta_0 + \beta_1 \text{ Restrictive}_j + \beta_2 \text{ Day}_{jj} + \beta_3 \text{ Restrictive}_j\text{*Day}_{ij} + \beta_4 \text{ Baseline Hgb}_i + \alpha_i + \varepsilon_{ij}$$

where i=participant, j= day 1, 2 or 3,
Restrictive = 1 for assigned to Restrictive and 0 for assigned to Liberal;
Day is categorical Day1, Day 2, Day 3;
α_i is a random intercept for participant i.

All patients with at least one non-missing in-hospital hemoglobin value on day 1, 2, or 3 will be included in the analysis. If the interaction is not statistically significant ($p>0.05$) it will be removed from the model. The estimated beta coefficients for Restrictive (versus Liberal) strategy and for the interaction between Restrictive strategy and Day will be presented along with their 95% confidence intervals and p-values. Contrasts will be created from this model to display the estimated difference in hemoglobin levels between the Restrictive and the Liberal groups on Day 1, Day 2 and Day 3.

We will also quantify adherence to the protocol by presenting the following among all randomized patients. For those assigned to Restrictive strategy, we will report the number (%) of randomized patients with at least one transfusion given when Hgb>8, and the number (%) of patients with at least one transfusion when no hemoglobin level was checked. Clinical reasons will be provided when possible. For those assigned to Liberal strategy, we will present the number (%) of randomized patients with no transfusion, and the number (%) of patients where the last hemoglobin value before hospital discharge is < 10 g/dL. Clinical reasons will be provided when possible. Among all randomized patients, we will present the number (%) of patients with one and with two or more missing required hemoglobin measures, and number (%) of patients with one and with two or more missing troponin measures.

Trial Outcome Analyses

The intention-to-treat principle will be used to test the primary, secondary and tertiary aims comparing study outcomes by transfusion strategy. Two-sided tests will be used with an alpha-level=0.05 for all

aims. No adjustment of the alpha level will be made for the secondary and tertiary analyses. Results will be interpreted based upon the observed findings, their designation as secondary or tertiary outcomes, and in comparison to the primary outcome result.

Primary Outcome Analysis

The primary endpoint (the composite of all-cause mortality and myocardial infarction (death/MI) by 30 days from randomization) will be compared by assigned transfusion strategy using an unadjusted log-binomial regression model with a fixed effect variable for assigned treatment strategy and a random effect for clinical site and accounting for missing data using multiple imputation to impute missing 30-day outcome data.

$$\text{Ln}\,(p_{ij}) = \beta_0 + \beta_1 \text{ Restrictive}_{ij} + \alpha_i + \varepsilon_{ij}$$

where p_{ij} = Prob(Primary Endpoint =1 | Participant Assigned Treatment and Site),
i=site, j= participant;
α_i is the random intercept for site i

We will present the estimated risk ratio, 95% CI and p-value for the Restrictive Strategy versus the Liberal Strategy based on this model. Markov Chain Monte Carlo (MCMC) multiple imputation methods will be used to impute the missing 30-day outcome values (yes/no) for death and MI based on all available observed data (baseline, in-hospital and 30-day variables). Ten imputed data sets will be created, a log-binomial model with random effects for site will be estimated for each imputed data set, and the results will be pooled to obtain a single estimate of treatment effect with an adjusted standard error. A log-binomial model with random effects for site will be created from patients with non-missing 30-day death/MI data as a sensitivity analysis. Also, if significant imbalances in baseline risk factors are detected between the two randomized treatment groups, a multivariable log-binomial regression model adjusting for the imbalanced factors will be created as a sensitivity analysis. If adherence to the protocol is a concern, a per protocol analysis, including only patients who undergo transfusion according to their assignment (and adjusting for baseline factors that are associated with adherence to the treatment protocol, will be conducted as a sensitivity analysis.

For each of these models, the primary hypothesis test will be a two-sided test with alpha=0.05:

H_0: RR= 1.00 versus H_A: RR ≠ 1.00 using a two-sided alpha-level=0.05

such that RR = e^{β} where β is the coefficient for the Restrictive assigned strategy (versus Liberal assigned strategy) from the log-binomial model for the primary endpoint. If superiority of one of the two transfusion

strategies is not demonstrated through the primary hypothesis test, a second hypothesis test of interest is the non-inferiority of the Restrictive Strategy compared to the Liberal strategy. Setting the non-inferior margin to a 15% relative increase, we will conduct the following one-sided non-inferiority hypothesis test with alpha=0.025:

H_0: RR ≥ 1.15 versus H_A: RR < 1.15 using a one-sided alpha-level=0.025.

With this test, rejecting the null hypothesis leads to the conclusion that the restrictive arm is non-inferior to the liberal arm. This is equivalent to the demonstrating that the entire 95% confidence interval for the estimated RR is < 1.15.

Among all patients with 30-day outcome data, the observed proportion of patients who experience the primary endpoint in each assigned group, the risk ratio (Restrictive versus Liberal) and 95% confidence interval, the risk difference (Restrictive – Liberal) and 95% confidence interval will be calculated and presented. See Table 2. We will determine whether this estimated 95% confidence interval for the RR includes 1.00 to assess superiority and whether it is <1.15 to assess non-inferiority of the Restrictive strategy. All available data from randomization through 30 days will be used to identify death and MI outcomes; for patients with incomplete follow-up, we assume that no event occurred after the time of last contact.

Kaplan-Meier methods will be used to display the cumulative risk of the primary endpoint over the 30-day post-randomization follow-up period for all randomized patients stratified by assigned treatment group (Figure 3a). For the Kaplan-Meier analyses, follow-up will be censored at the time of withdrawal, lost-to-follow-up, or 30 days, and a log-rank statistic will be used to compare the two curves. The estimated risks at 30-days and the risk difference and 95% confidence interval at 30-days will be presented.

Analysis of Secondary and Other Outcomes

All of the secondary outcomes and most of the tertiary outcomes are dichotomous endpoints (i.e. presence/absence of an event during the 30-day study period). The pre-defined secondary outcomes are: 1) all-cause mortality within 30 days; 2) myocardial reinfarction within 30 days; and 3) the composite outcome of all-cause mortality, nonfatal myocardial reinfarction, ischemia driven unscheduled coronary revascularization (percutaneous coronary intervention or coronary artery bypass grafting), or readmission to the hospital for ischemic cardiac diagnosis within 30 days. Other dichotomous outcomes include: 1) all-cause mortality, nonfatal myocardial reinfarction, and unstable angina (i.e. acute coronary syndrome) 2) ischemia driven unscheduled coronary revascularization within 30-days; 3) readmission to the hospital for ischemic cardiac diagnosis, 4) congestive heart failure within 30 days; 5) unscheduled readmission to

hospital for any reason within 30 days; 6) each of the individual cardiovascular outcomes of stroke, pulmonary embolism or deep venous thrombosis, bleeding, and cardiac death within 30 days; 7) each of the individual infectious outcomes of pneumonia and blood stream infections within 30 days.

For each of the dichotomous endpoints listed above, the observed proportion of patients who experience the endpoint in each assigned group, the risk ratio (Restrictive versus Liberal) and 95% confidence interval, the risk difference (Restrictive – Liberal) and 95% confidence intervals will be calculated and presented (Table 2). Superiority of either transfusion strategy for each endpoint will first be evaluated using a two-sided test with alpha=0.05. If superiority is not detected, the non-inferiority of the Restrictive strategy will be assessed using a 15% relative non-inferiority margin; that is, the Restrictive strategy will be deemed non-inferior for a given dichotomous endpoint when the entire 95% confidence interval for the estimated RR is < 1.15. For the 30-day dichotomous outcomes, all available data from randomization through 30 days will be used to identify outcomes. Patients with incomplete follow-up will be assumed to have no event after the time of last contact for these analyses.

The length of hospital stay from randomization to discharge and the number of days in the ICU from randomization to discharge will be described as a mean (sd) or median (first and third quartile) in each assigned group and the difference will be analyzed using non-parametric Wilcoxon rank sum tests (see for example, Table 3). The mean (sd) or median (first and third quartile) will be presented for the EQ-5D utility index value and the EQ-5D Health Today score at 30 days post-randomization, and the difference will be tested based on t-tests or the Wilcoxon rank sum test depending on the distribution of the scores. The proportion of patients who report no problems with each of the 5 individual components of the EQ-5D (Mobility, Self-Care, Usual Activities, Pain/Discomfort, Anxiety/Depression) will be presented stratified by assigned treatment group (Table 3).

The cumulative risk of all-cause mortality through 180-day post-randomization will be estimated using Kaplan-Meier methods for all randomized patients stratified by assigned treatment group (Figure 3b). For the time-to-event analyses, follow-up will be censored at the time of withdrawal, lost-to-follow-up, or 180 days. The estimated risk at 30 days and at 180 days for each treatment group will be presented, and a log-rank statistic will be used to test the hypothesis that the mortality risk over the 180-days is equivalent in the two assigned treatment groups. The estimated risk difference (Restrictive – Liberal) and 95% confidence interval will be presented for cumulative mortality risk at 30 days and at 180 days. If neither strategy is found to be superior based on the log rank test and the 95% confidence interval for the estimated risk difference (RD) at 180-days includes 1.0, the 95% confidence interval for the 180-day mortality risk difference will be assessed using a non-inferiority margin equal to an absolute difference of

3.0%. Thus, if the entire estimated 95% confidence for the 180-day mortality RD is <3.0%, the Restrictive strategy will be considered non-inferior to the Liberal Strategy.

Missing data will not be imputed for the analyses of the secondary and tertiary hypotheses unless critical issues are noted while investigating the missing 30-day primary outcome data.

Subgroup Analyses

Subgroup analyses will be performed based on the following baseline factors: STEMI and NSTEMI, MI types 1 and 2, baseline hemoglobin level (<8, 8-8.9, ≥9 g/dL), revascularization for treatment of index MI prior to randomization (yes, no), acute anemia (acute anemia, chronic anemia), sex (male, female), age (<60, 60-69, 70-79, ≥80 years), heart failure (defined as history of congestive heart failure, congestive heart failure during the index hospitalization prior to randomization and/or left ventricular ejection fraction < 45%: yes, no), renal function (renal dialysis during index hospitalization prior to randomization, no dialysis and eGFR <30, no dialysis and eGFR 30-59, no dialysis and eGFR ≥ 60 mL/mi/1.73m^2) based on the CKD-EPI formula without race, and diabetes mellitus (diabetes mellitus treated with medication: yes, no). For participants from sites in the United States, Canada, New Zealand and Australia, we will also consider Race (White, Black, Non-White and Non-Black Race) and Hispanic ethnicity (yes, no). Participants from the European Union (EU) and Brazil are excluded from these analyses because race and ethnicity were not collected in the EU sites, and these concepts are challenging to define in Brazil.

The proportion of patients with a primary endpoint within 30 days will be reported for each assigned treatment group within each pre-defined subgroup. If subgroup categories are too small for meaningful inference, we will collapse or eliminate categories. The risk ratio (Restrictive / Liberal), 95% confidence interval and significance-level will be reported with a forest plot (Figures 4a and 4b). For each subgroup variable, a log-binomial regression model including subgroup variable, treatment assignment and the interaction between the subgroup variable and treatment assignment will be created, and the significance of the interaction term will be used to test whether the treatment effect is significantly modified by the designated subgroup variable.

$$\text{Ln}\,(p_i) = \beta_0 + \beta_1\,\text{Subgroup}_i + \beta_2\,\text{Restrictive}_i + \beta_3\,\text{Subgroup}_i{*}\text{Restrictive}_i + \varepsilon_i$$

where p_i = Prob(Primary Endpoint=1 | Participant Assigned Treatment and Subgroup), i= participant;

The focus of the subgroup analyses in the MINT trial is for descriptive purposes. When applicable, continuous versions of the subgroup variable will be considered for the regression models, and we will

explore the existence of inflection points for treatment effectiveness. Subgroup analyses may be examined for secondary and other outcomes utilizing similar methods. For 6-month mortality, Cox proportional hazards regression models will be created with the same covariates to test whether the effect of the randomized transfusion strategy for 6-month mortality varies significantly according to the pre-specified subgroup variables.

Planned Secondary Analyses

A number of secondary manuscripts will be undertaken to evaluate the effect of specified subgroup variables on treatment effectiveness in greater depth. These analyses will consider specified trial endpoints and other clinical outcomes that are relevant to the research question. The analyses will also explore confounders and mediators that explain the relationships between the subgroup variable, transfusion treatment strategy, and outcomes.

Multivariable regression models (linear, logistic and Cox regression) will be used to elucidate the roles of demographic and clinical factors as predictors of defined clinical and patient reported outcomes in this patient population. Outcomes of interest include the trial primary endpoint, the three secondary endpoints, congestive heart failure, unstable angina or unscheduled coronary revascularization, infection, health related quality of life as measured by the EuroQol-5D at 30-days, and six-month mortality.

We will also take a personalized medicine approach to characterize patients who benefit from a liberal transfusion strategy and patients who do as well, or better, with a restrictive transfusion strategy, with respect to the primary outcome and a few key secondary endpoints. For a given endpoint, we will use machine learning methods to identify baseline characteristics that lead to better outcomes, and we will use generated effect modifier (GEM) models to create a function of the identified risk factors that interacts with assigned treatment. This function may allow us to predict the treatment strategy with which each patient would benefit based on their individual clinical and demographic profile.

We will employ causal inference principles to assess the impact of various hemoglobin thresholds for transfusion on the primary outcome and 30-day death in the MINT trial. Using the observed trial data, we will emulate a parallel target trial with several different hemoglobin transfusion thresholds (< 10 g/dL, < 9 g/dL, < 8 g/dL and < 7 g/dL). All patients will be followed under each of the four transfusion strategies until the time they no longer adhere to that strategy based on the measured hemoglobin levels and transfusions administered. We will apply contemporary causal inference data analytic approaches aimed at minimizing biases to analyze the effect of treatment received (rather than assigned treatment) to determine the best hemoglobin threshold for this patient population.

REFERENCES

1. Thygesen K, Alpert JS, Jaffe AS, Simoons ML, Chaitman BR, White HD, et al. Third universal definition of myocardial infarction. J Am Coll Cardiol 2012;60(16):1581-98.

2. Hicks KA, Tcheng JE, Bozkurt B, Chaitman BR, Cutlip DE, Farb A, et al. 2014 ACC/AHA Key Data Elements and Definitions for Cardiovascular Endpoint Events in Clinical Trials: A Report of the American College of Cardiology/American Heart Association Task Force on Clinical Data Standards (Writing Committee to Develop Cardiovascular Endpoints Data Standards). J Am Coll Cardiol 2015;66(4):403-69.

3. Schulman S, Kearon C, Subcommittee on Control of Anticoagulation of the S, Standardization Committee of the International Society on T, Haemostasis. Definition of major bleeding in clinical investigations of antihemostatic medicinal products in non-surgical patients. J Thromb Haemost 2005;3(4):692-4.

4. CDC/NHSN surveillance definitions for specific types of infections. In: CDC; 2016.

5. van Hout B, Janssen MF, Feng YS, Kohlmann T, Busschbach J, Golicki D, et al. Interim scoring for the EQ-5D-5L: mapping the EQ-5D-5L to EQ-5D-3L value sets. Value Health 2012;15(5):708-15.

6. Pickard S, Law E, Jiang R, Pullenayegum E, Shaw J, Xie F, Oppe F, Boye K, Chapman R, Gong C, Balch A, Busschbach J. United States Valuation of EQ-5D-5L Health States Using an International Protocol. VALUE HEALTH. 2019; 22(8):931–941.

Figure 1: CONSORT Chart

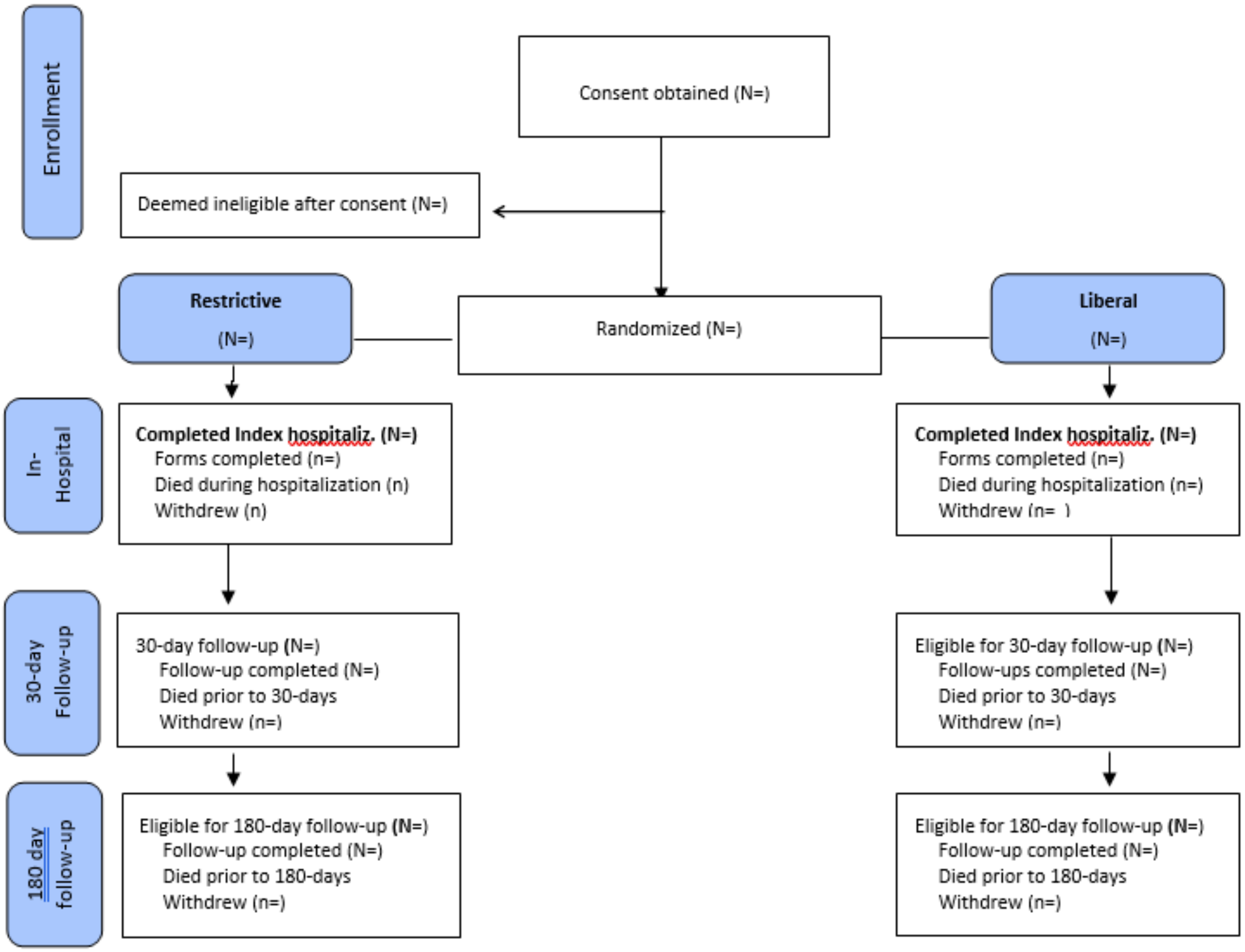

Table 1: Baseline Characteristics

Characteristic	All Participants (N=)	Restrictive (N=)	Liberal (N=)
Patient Characteristics			
Country, n (%)			
US			
Canada			
France			
Brazil			
New Zealand / Australia			
Age, years, mean (sd)			
Male, n (%)			
Hispanic Latino or Latina, n (%)			
Race			
White or Caucasian, n (%)			
Black or African-American, n (%)			
Asian, n (%)			
Other Race, n (%)			
Unknown at this time, n (%)			
BMI, kg/m^2, mean (sd)			
Tobacco smoker, n (%)			
Never			
Former			
Current			
Medical History			
MI, n (%)			
PCI, n (%)			
CABG, n (%)			
CHF, n (%)			
Stroke or TIA, n (%)			
Atrial fibrillation, n (%)			
PAD, n (%)			
Renal failure/insufficiency, n (%)			
Diabetes, n (%)			
Hypertension, n (%)			
Hypercholesterolemia/hyperlipidemia, n (%)			
COPD/asthma, n (%)			
Cancer, n (%)			
Anemia, n (%)			
Documented diagnosis of COVID-19, n (%)			
Angiogram results, n (%)			
Number of vessels with > 50% obstruction, n (%)			
0			
1			
2			
3			
Left main disease, n (%)			

Characteristic	All Participants (N=)	Restrictive (N=)	Liberal (N=)
LVEF results available, n (%)			
Most recent LV ejection fraction (%) within the past year, mean (sd)			
LV ejection fraction: Severity level, n (%)			
55% or greater (normal)			
45% to less than 55% (mild)			
30% to less than 45% (moderate)			
less than 30% (severe)			
Index Myocardial Infarction (MI)			
MI classification, n (%)			
Type 1			
Type 2			
Type 4b			
Type 4c			
Unknown			
Symptoms of ischemia, n (%)			
New ST-T changes or new left bundle branch block, n (%)			
Development of pathological Q waves, n (%)			
New loss of viable myocardium or regional wall motion abnormality, n (%)			
Identification of an intracoronary thrombus, n (%)			
Chest pain, n (%)			
Symptoms related to onset of index MI			
Jaw pain, n (%)			
Back pain, n (%)			
Shortness of breath, n (%)			
Epigastric pain, n (%)			
Palpitations, n (%)			
Left arm pain, n (%)			
Syncope, n (%)			
Other symptoms related to MI, n (%)			
Diagnosis of MI at time of randomization confirmed as MI at time of discharge, n (%)			
Events during index hospitalization prior to randomization			
PCI occurred prior to randomization, n (%)			
CABG occurred prior to randomization, n (%)			
Revascularization post-MI and prior to randomization, n (%)			
CHF prior to randomization, n (%)			
Renal dialysis prior to randomization, n (%)			
Intubated on ventilator prior to randomization, n (%)			
Active bleeding prior to randomization, n (%)			
Received RBC transfusion prior to randomization, n (%)			
Number of units, mean (sd)			
Laboratory Measures most recent prior to randomization			
Hemoglobin: Level (g/dL), mean (sd)			
Hemoglobin: Level (g/dL), n (%)			
<8.0			
8.0-8.9			
≥9.0			

Characteristic	All Participants (N=)	Restrictive (N=)	Liberal (N=)
Most recent creatinine value (mg/dL), mean (sd)			
eGFR (mL/mi/1.73m^2), mean (sd)			
Renal function, n (%)			
Renal dialysis during hospitalization pre-randomization			
<30 mL/mi/1.73m^2 and no dialysis			
30-59 mL/mi/1.73m^2 and no dialysis			
≥60 mL/mi/1.73m^2 and no dialysis			
Location at time of randomization			
In ICU/CCU at randomization, n (%)			

Figure 2a: Post-randomization Transfusions during Index Hospitalization

Histogram of number of units transfused as part of the protocol for all randomized patients stratified by assigned treatment.

Figure 2b: Post-randomization Hemoglobin by Assigned Strategy

Line plot of mean hemoglobin at Baseline, Day1, Day 2 and Day 3 for all randomized patients stratified by assigned treatment.

Table 2: MINT Trial Outcomes

	Cumulative Events at 30 days (Patient-Level)				
Characteristic	Restrictive (N=)	Liberal (N=)	Risk Difference* (95% CI)	Risk Ratio* (95% CI)	p-value
Primary Outcome (at 30 days)					
All-cause mortality/adjudicated myocardial infarction (MI), n (%)					
Secondary Outcome (at 30 days)					
All-cause mortality, n (%)					
Adjudicated myocardial infarction, n (%)					
All-cause mortality, adjudicated MI, ischemia driven unscheduled revascularization, unscheduled readmission to the hospital for ischemic cardiac diagnosis, n (%)					
Other Outcome (at 30 days)					
All-cause mortality, adjudicated non-fatal MI, unstable angina, n (%)					
Ischemia driven unscheduled revascularization, n (%)					
Unscheduled readmission to the hospital for ischemic cardiac diagnosis, n (%)					
Heart Failure, n (%)					
Unscheduled readmission to hospital for any reason, n (%)					
Stroke, n (%)					
Pulmonary embolism or deep vein thrombosis, n (%)					
Bleeding event, n (%)					
Cardiac Death, n (%)					
Pneumonia or bacteremia, n (%)					

** Risk Difference = Restrictive - Liberal for event outcomes*

Risk Ratio = Restrictive / Liberal for event outcomes

Restrictive is considered non-inferior to Liberal if the upper bound of the Risk Ratio 95% Confidence Interval is <1.15

Table 3: MINT Additional Outcomes

	Restrictive (N=)	Liberal (N=)	Mean Difference* (95% CI)	p-value		
Outcomes						
Length of hospital stay, number of days, mean (sd)						
Length of stay in the ICU, number of days, mean (sd)						
**EuroQol 5D at 30-days**						
EuroQol 5D results, n (%)						
EQ-5D Utility Index, mean (sd)						
EQ-5D Health Today, mean (sd)						
	**Restrictive (N=)**	**Liberal (N=)**	**Risk Difference* (95% CI)**	**Risk Ratio* (95% CI)**	**p-value**	
No problems identified with:						
EQ-5D Mobility, n (%)						
EQ-5D Self-Care, n (%)						
EQ-5D Usual Activities, n (%)						
EQ-5D Pain/Discomfort, n (%)						
EQ-5D Anxiety/Depression, n (%)	**Restrictive (N=)**	**Liberal (N=)**	**Restrictive K-M Estimate at 180 days**	**Liberal K-M Estimate at 180 days**	**Risk Difference** (95% CI)**	**p-value**
All-cause mortality at 180 days, n (%)						

** Mean difference = Restrictive - Liberal*

Risk Difference = Restrictive - Liberal for event outcomes

Risk Ratio = Restrictive / Liberal for event outcomes

*** For 180-day all-cause mortality Restrictive is considered non-inferior to Liberal if the upper bound of the Risk Difference 95% Confidence Interval is <3.0%*

Figure 3a: 30-day Cumulative Incidence Curves for All-Cause Death / Adjudicated MI
Cumulative risk using Kaplan Meier methods stratified by assigned treatment with estimates at 30-days presented.

Figure 3b: 180-day Cumulative Incidence Curves for All-Cause Death
Cumulative risk using Kaplan Meier methods stratified by assigned treatment with estimates at 30-days and at 180-days presented.

Figure 4a: 30-day Death/MI by Assigned Transfusion Strategy and Predefined Subgroups

All Randomized Participants: Subgroups	Subgroup Sample Size	Restrictive (N=)	Liberal (N=)	Risk Ratio	95% CI	FIGURE RR* (95% CI)	Interaction p-value
All Participants	N(%)	n (%)	n (%)	RR	LL - UL	---------X----------	
MI Category							p-value
STEMI	N(%)	n (%)	n (%)	RR	LL - UL	---------X----------	
non STEMI	N(%)	n (%)	n (%)	RR	LL - UL	---------X--------	
MI Type							p-value
Type 1 MI	N(%)	n (%)	n (%)	RR	LL - UL	---------X----------	
Type 2 MI	N(%)	n (%)	n (%)	RR	LL - UL	---------X--------	
Baseline hemoglobin level							p-value
<8 g/dL	N(%)	n (%)	n (%)	RR	LL - UL	---------X----------	
8-8.9 g/dL	N(%)	n (%)	n (%)	RR	LL – UL	---------X--------	
≥9.0 g/dL	N(%)	n (%)	n (%)	RR	LL – UL	------X-------	
Revascularization post-MI and pre-randomization*							p-value
Yes	N(%)	n (%)	n (%)	RR	LL – UL	---------X----------	
No	N(%)	n (%)	n (%)	RR	LL – UL	---------X--------	
Acute Anemia							p-value
Acute Anemia	N(%)	n (%)	n (%)	RR	LL – UL	------X-------	
Chronic Anemia	N(%)	n (%)	n (%)	RR	LL – UL	---------X----------	
Sex							
Male							
Female							
Age							
< 60 years							
60-69 years							
70-79 years							
≥ 80 years							
Heart Failure							
Yes							
No							
Renal Function							
Renal dialysis							
< 30 mL/mi/1,73m^2							
30-59 mL/mi/1,73m^2							
≥69 mL/mi/1,73m^2							
Diabetes Mellitus (treated)							
Yes							
No							

N (%) indicates the sample size of the subgroup and the proportion of the total sample in that subgroup.

n (%) indicates the number of outcome events in the specified treatment group and subgroup, and the proportion of people in the specified treatment group and subgroup who experienced the event.

RR: Rate Ratio = Restrictive / Liberal

* the No Revascularization group includes XX who received revascularization between randomization and hospital discharge YY of whom had a revascularization that was planned prior to randomization and was not based on new signs or symptoms consistent with ischemia that occurred post-randomization.

Figure 4b: 30-day Death/MI by Assigned Transfusion Strategy and Predefined Subgroups

U.S., Canada, New Zealand and Australia Participants: Subgroups	Subgroup Sample Size	Restrictive (N=)	Liberal (N=)	Risk Ratio	95% CI	FIGURE RR* (95% CI)	Interaction p-value
U.S., Canada, New Zealand and Australia Participants	N(%)	n (%)	n (%)	RR	LL - UL	---------X----------	
Race							p-value
White	N(%)	n (%)	n (%)	RR	LL - UL	---------X----------	
Black	N(%)	n (%)	n (%)	RR	LL - UL	---------X----------	
non-White & non-Black	N(%)	n (%)	n (%)	RR	LL - UL	---------X----------	
Hispanic							p-value
Yes	N(%)	n (%)	n (%)	RR	LL - UL	---------X----------	
No	N(%)	n (%)	n (%)	RR	LL - UL	---------X----------	

N (%) indicates the sample size of the subgroup and the proportion of the total sample in that subgroup.

n (%) indicates the number of outcome events in the specified treatment group and subgroup, and the proportion of people in the specified treatment group and subgroup who experienced the event.

RR: Rate Ratio = Restrictive / Liberal

Supplementary Information: Other 30-day Outcomes and Adverse Events

Characteristic	Cumulative Rate of Event at 30 days (Patient-Level)	
	Restrictive (N=)	Liberal (N=)
Outcome Events		
New suspected MI, n (%)		
Procedures: PCI, n (%)		
Ischemia driven unscheduled PCI, n (%)		
Procedures: CABG, n (%)		
Ischemia driven unscheduled CABG, n (%)		
New unstable angina, n (%)		
Transient Ischemic Attack (TIA), n (%)		
Any indication of Heart Failure, n (%)		
Any Indication of TACO, n (%)		
Strict Definition of TACO, n (%)		
Arrhythmias		
Atrial fibrillation/flutter, n (%)		
Supraventricular tachycardias other than atrial fibrillation, n (%)		
Paroxysmal supraventricular tachycardia, n (%)		
Ventricular tachycardia, n (%)		
Ventricular fibrillation, n (%)		
Cardiac arrest, n (%)		
Mobitz type 1 AV block, n (%)		
Mobitz type 2 AV block, n (%)		
Complete (3rd degree) AV block, n (%)		
Right bundle branch block, n (%)		
Left bundle branch block, n (%)		
Bifascicular block, n (%)		
Asystole, n (%)		
Multifocal atrial tachycardia, n (%)		
Nonparoxysmal junctional tachycardia, n (%)		
Thrombotic/Ischemic Events and Complications of MI		
Chest pain or stable angina, n (%)		
Mechanical complications of MI, n (%)		
Pericarditis, n (%)		
Pericardial effusion/tamponade, n (%)		
Syncope, n (%)		
Cardiomyopathy, n (%)		
Cardiogenic Shock, n (%)		
Other Adverse Events		
Acute respiratory failure, n (%)		
Acute renal failure, n (%)		
Transfusion Reactions		
Transfusion related acute lung injury, n (%)		
Acute hemolytic transfusion reaction, n (%)		
Transfusion associated sepsis, n (%)		
Anaphylactic transfusion reaction, n (%)		

Characteristic	Cumulative Rate of Event at 30 days (Patient-Level)	
	Restrictive (N=)	Liberal (N=)
Urticarial transfusion reaction, n (%)		
Febrile non-hemolytic reaction, n (%)		
Other Infections		
Urinary tract infection, n (%)		
Septic shock, n (%)		
Clostridium, n (%)		
Cellulitis, n (%)		
Endocarditis, n (%)		
Mediastinitis, n (%)		
Osteomyelitis, n (%)		
Sinusitis, n (%)		
Influenza, n (%)<br>Gastroenteritis, n (%)		

Supplementary 30-day Information

Characteristic	Restrictive (N=)	Liberal (N=)
Number of ER visits, n (%)		
0		
1		
2		
3+		
Number of ER visits, mean (sd)		
Number of hospital readmissions, n (%)		
0		
1		
2		
3		
Number of hospital readmissions, mean (sd)		

MINT Statistical Analysis Plan: Summary of Changes

July 24, 2023

December 2016: The basic structure of the statistical analysis plan was laid out in the original MINT protocol approved by both the IRB and DSMB. No substantial changes were made to the statistical plan as the protocol was modified (Current version: Version 7.1 October 24, 2017).

July 8, 2022: The original DSMB-approved Data Safety Monitoring Plan had noted that no interim futility analyses would be conducted since the MINT trial compares two established transfusion strategies with different resource and cost implications, and hence, a null result from a well-powered trial would be important for establishing treatment guidelines and policy. Due to enrollment challenges, we submitted a proposal to NHLBI in December 2021 to extend the trial enrollment period in order to achieve the original target sample size of N=3500 participants. In February 2022, the NHLBI requested that the MINT DSMB review a futility analysis. The MINT Investigators created a revised Interim Statistical Monitoring Plan dated April 28, 2022, and this plan was approved by a blinded statistical team at NHLBI. The futility analysis involved the computation of conditional power of the primary two-sided superiority hypothesis. We also proposed to evaluate the conditional power of the non-inferiority of the restrictive strategy given the clinical preference to use fewer red blood cell units if outcomes were comparable. On July 22, 2022, the DSMB reviewed the trial interim data and futility analysis results. The DSMB recommended that the MINT Trial continue with a target sample size of N=3500.

A Statistical Analysis Plan, separate from the protocol, was created in preparation for the July 22, 2022 DSMB meeting.

This version of the SAP included the following:

- We added a non-inferiority analysis of the restrictive transfusion strategy.
  H_0: RR $\geq$ 1.15 versus H_A: RR < 1.15 using a one-sided alpha-level=0.025.
  This is equivalent to demonstrating that the entire 95% confidence interval for the estimated RR is < 1.15.
- We added the plan for the futility analysis which included an evaluation of conditional power for the primary trial two-sided superiority hypothesis as well as the conditional power of the non-inferiority hypothesis.
- We more precisely defined the baseline variables that would be used to create subgroups for subgroup analyses.
- We added planned secondary analyses, most notably:
  - Evaluate the effect of specified subgroup variables on treatment effectiveness in greater depth.
  - Identify predictors of outcomes from multivariable models for key trial endpoints.
  - Utilize exploratory data analysis methods to create a function of risk factors that interact with assigned treatment for the primary outcome (death/MI) and secondary trial outcomes of interest. This function could potentially be used to identify patient profiles who benefit with one transfusion strategy or the other.
  - Utilize target trial causal inference methods to compare different hemoglobin transfusion thresholds (< 10 g/dL, < 9 g/dL, < 8 g/dL and < 7 g/dL) based on treatment received with respect to the primary trial outcome (death/MI) and 30-day death.
