# Supplementary material for: Anemia Acuity Effect on Transfusion Strategies in Acute Myocardial Infarction: A Secondary Analysis of the MINT Trial
Source: JAMA Netw Open. 2024 Nov 1;7(11):e2442361. doi: 10.1001/jamanetworkopen.2024.42361 (PMC11530937; doi:10.1001/jamanetworkopen.2024.42361)
Supplement: Supplement 2. — eTable 1. Participants’ Characteristics Included and Excluded From the Sub-Study eTable 2. Distribution of RBC Transfusions From Randomization to Hospital Discharge eTable 3. Association Between Acute Anemia and 30-Day Outcomes Using an Alternate Definition of Acute and Chronic Anemia eTable 4. Relative Excess Risk Due to Interaction (RERI) for 30-Day Outcomes eTable 5. Distribution, Association With, and Intervention Effect on 30-Day Transfusion-Related Pulmonary Complications eTable 6. Association Between Acute Anemia and 30-Day Outcomes, Restricted to Participants Hospitalized for 3 Days or More at Time of Randomization eTable 7. Association Between Acute Anemia and 30-Day Outcomes, Restricted to Participants eFigure 1. Effect of a Restrictive Strategy, Compared to a Liberal Strategy, on 30-Day Outcomes Overall and Stratified by Anemia Acuity, Using an Alternate Definition of Acute and Chronic Anemia eFigure 2. Effect of a Restrictive Strategy, Compared to a Liberal Strategy, on 30-Day Outcomes Overall and Stratified by Anemia Acuity, Restricted to Participants Hospitalized for 3 Days or More at Time of Randomization eFigure 3. Effect of a Restrictive Strategy, Compared to a Liberal Strategy, on 30-Day Outcomes Overall and Stratified by Anemia Acuity, Restricted to Participants Who Did Not Receive RBCs Prior to Randomization [file jamanetwopen-e2442361-s002.pdf]

Supplementary Online Content

Carrier FM, Cooper HA, Portela GT, et al. Anemia acuity effect on transfusion strategies in acute myocardial infarction: a secondary analysis of the MINT trial. *JAMA Netw Open*. 2024;7(11):e2442361. doi:10.1001/jamanetworkopen.2024.42361

eTable 1. Participants' Characteristics Included and Excluded From the Sub-Study

eTable 2. Distribution of RBC Transfusions From Randomization to Hospital Discharge

eTable 3. Association Between Acute Anemia and 30-Day Outcomes Using an Alternate Definition of Acute and Chronic Anemia

eTable 4. Relative Excess Risk Due to Interaction (RERI) for 30-Day Outcomes

eTable 5. Distribution, Association With, and Intervention Effect on 30-Day Transfusion-Related Pulmonary Complications

eTable 6. Association Between Acute Anemia and 30-Day Outcomes, Restricted to Participants Hospitalized for 3 Days or More at Time of Randomization

eTable 7. Association Between Acute Anemia and 30-Day Outcomes, Restricted to Participants

eFigure 1. Effect of a Restrictive Strategy, Compared to a Liberal Strategy, on 30-Day Outcomes Overall and Stratified by Anemia Acuity, Using an Alternate Definition of Acute and Chronic Anemia

eFigure 2. Effect of a Restrictive Strategy, Compared to a Liberal Strategy, on 30-Day Outcomes Overall and Stratified by Anemia Acuity, Restricted to Participants Hospitalized for 3 Days or More at Time of Randomization

eFigure 3. Effect of a Restrictive Strategy, Compared to a Liberal Strategy, on 30-Day Outcomes Overall and Stratified by Anemia Acuity, Restricted to Participants Who Did Not Receive RBCs Prior to Randomization

This supplementary material has been provided by the authors to give readers additional information about their work.

© 2024 Carrier FM et al. *JAMA Network Open.*

eTable 1. Participants' characteristics included and excluded from the sub-study

Total MINT sample (n = 3,504)	Included (n = 3,144)	Excluded (n = 360)
Demographics, No. (%)		
Age, mean (SD)[a]	72.3 (11.6)	71.1 (11.7)
Sex (male)	1,715 (54.5)	196 (54.4)
Body mass index, mean (SD)[a]	28.6 (7.1)	28.1 (6.7)
Comorbidities, No. (%)		
Smoking Status[a]		
Never	1,194 (40.2)	134 (40.1)
Former	1,283 (43.2)	145 (43.4)
Current	493 (16.9)	55 (16.5)
History of MI	1,023 (32.5)	115 (31.9)
Most recent ejection fraction, mean (SD)[a]	47.4 (13.7)	47.5 (12.1)
History of stroke	551 (17.5)	67 (18.6)
History of atrial fibrillation	807 (25.7)	93 (25.8)
History of peripheral artery disease	639 (20.3)	81 (22.5)
History of renal failure	1,434 (45.6)	173 (48.1)
History of diabetes mellitus	1,690 (53.8)	206 (57.2)
History of hypertension	2,670 (84.9)	306 (85.0)
History of hypercholesterolemia	2,038 (64.8)	232 (64.4)
History of chronic obstructive pulmonary disease or asthma	747 (23.8)	93 (25.8)
History of cancer	703 (22.4)	66 (18.3)
MI characteristics at randomization, No. (%)		
MI type		
Type 1	1,307 (41.6)	153 (42.5)
Type 2	1,756 (55.9)	199 (55.3)
Other	81 (2.6)	8 (2.2)
Performed angiogram[a]	1,539 (49.0)	199 (55.3)
Percutaneous intervention	935 (29.7)	121 (33.6)
Coronary artery bypass graft surgery	11 (0.3)	0 (0.0)
Time from first symptoms to hospital admission (days), median (Q1, Q3)[a]	0 (0, 1)	0 (0, 0)
Time from hospital admission to randomization (days), median (Q1, Q3)	3 (1, 5)	4 (2, 8)
Anticoagulants at randomization[a,], No. (%)		
**Number of antiplatelets or anticoagulants**		

© 2024 Carrier FM et al. *JAMA Network Open.*

0	215 (6.8)	15 (4.2)
1	482 (15.3)	42 (11.7)
2	933 (29.7)	110 (30.6)
3+	1,513 (48.1)	193 (53.6)
**Antiplatelets**		
Aspirin	2,586 (82.3)	317 (88.1)
P2Y12 Inhibitor	1,699 (54.1)	224 (62.2)
Glycoprotein IIb/IIIa inhibitor	83 (2.6)	5 (1.4)
**Anticoagulants**		
Unfractionated or low molecular weight heparin	2,206 (70.2)	268 (74.4)
Warfarin	129 (4.1)	16 (4.4)
Any other anticoagulant	317 (10.1)	34 (9.4)
Other characteristics prior to randomization, No. (%)		
Any RBC transfusion	1,117 (35.5)	120 (33.3)
Number of RBC units transfused (units), median (Q1, Q3)[a]	0 (0, 1)	0 (0, 1)
Clinical bleed	414 (13.2)	45 (12.5)
Critical care at randomization[b]	1,470 (46.8)	209 (58.1)
Dialysis	358 (11.4)	57 (15.8)
Mechanical ventilation	426 (13.5)	55 (15.3)

[a] The following variables had missing values which were not included in the denominator of the percentages or the calculations of the means (SD): Age (n=1); Body Mass Index (n=139); Smoking Status (n=200); Most Recent Ejection Fraction (n=1246); Performed Angiogram (n=1); Days from Symptoms to Hospital Admission (n=473); Anticoagulants at randomization (n=1); Number of RBC units Transfused (n=12).
[b] Hospitalization in a critical dare unit or a coronary care unit
MI = Myocardial Infarction, SD = Standard deviation, Q1= first quartile, Q3= third quartile

© 2024 Carrier FM et al. *JAMA Network Open.*

eTable 2. Distribution of RBC transfusions from randomization to hospital discharge

n=3,144	Acute anemia (n=1,078)			Chronic anemia (n=2,066)		
**Outcomes**	**Total**	**Liberal (n=539)**	**Restrictive (n=539)**	**Total**	**Liberal (n=1,054)**	**Restrictive (n=1,012)**
Number of RBC units transfused, median (Q1, Q3)	1 (0,2)	2 (1,3)	0 (0,1)	1 (0,2)	2 (1,3)	0 (0,1)

Q1= first quartile, Q3= third quartile, RBC = Red Blood Cells

© 2024 Carrier FM et al. *JAMA Network Open.*

eTable 3. Association between acute anemia and 30-day outcomes using an alternate definition of acute and chronic anemia[a]

30-day outcomes	Crude RR (95% CI)	Adjusted RR (95% CI)[b]
Death or recurrent MI	0.90 (0.68, 1.18)	0.95 (0.72, 1.26)
Death	1.16 (0.83, 1.62)	1.27 (0.89, 1.81)
Recurrent MI	0.71 (0.46, 1.11)	0.73 (0.46, 1.15)
Cardiac death	1.02 (0.60, 1.73)	0.95 (0.55, 1.64)
Heart failure	0.76 (0.47, 1.23)	0.80 (0.49, 1.31)
Pulmonary complications[c]	1.15 (0.87, 1.51)	1.31 (0.98, 1.75)
Major bleeding episode	1.15 (0.85, 1.55)	1.34 (0.98, 1.84)

Acute anemia (n=339) is compared to chronic anemia (n=2805).

[a] Alternate definition: Acute Anemia group included participants with a WHO-defined normal Hgb at admission followed by a drop to <10 g/dL (to be eligible for the MINT trial). All others were placed in the Chronic Anemia group.
[b] The models were adjusted for randomized transfusion strategy, demographics (age, sex, smoking [never, ever, current, unknown], and anemia-related comorbidities (cancer, renal failure, diabetes), pre-randomization characteristics (baseline hemoglobin [g/dL], number of anticoagulants [warfarin, heparin, other] and antiplatelets [aspirin, P2Y12, glycoprotein], MI type [type 1, type 2, other]) with a random effect for site.
[c] Pulmonary complications = TRALI, TACO, pneumonia, or acute respiratory failure
RR = Risk ratio, CI = Confidence intervals, MI = Myocardial infarction, TACO = Transfusion associated circulatory overload, TRALI = Transfusion related acute lung injury.

© 2024 Carrier FM et al. *JAMA Network Open.*

eTable 4. Relative excess risk due to interaction (RERI) for 30-day outcomes

30-day outcomes	RERI (95% CI[a])
Death or recurrent MI	-0.08 (-0.68 to 0.49)
Death	0.06 (-0.84 to 0.78)
Recurrent MI	-0.11 (-1.02 to 0.56)
Cardiac mortality	-0.01 (-1.68 to 1.22)
Heart failure	-0.35 (-1.25 to 0.44)
Pulmonary complications[b]	0.18 (-0.32 to 0.69)
Major bleeding episode	-0.33 (-1.08 to 0.26)

A RERI = 0 means no interaction (no effect modification), a RERI below 0 means a negative interaction and a RERI above 1 means a positive interaction.

[a] Based on 95% clustered bootstrap CIs from 1000 resamples (model convergence occurred in >96% of iterations for all outcomes)

[b] Pulmonary complications = TRALI, TACO, pneumonia, or acute respiratory failure

© 2024 Carrier FM et al. *JAMA Network Open.*

eTable 5. Distribution, association with, and intervention effect on 30-day transfusion-related pulmonary complications

A. Crude distribution of the outcome

n=3,144	Acute anemia (n=1,078)			Chronic anemia (n=2,066)		
**Outcomes**	**Total**	**Liberal (n=539)**	**Restrictive (n=539)**	**Total**	**Liberal (n=1,054)**	**Restrictive (n=1,012)**
TACO & TRALI, No. (%)	12 (1.11)	10 (1.86)	2 (0.37)	20 (0.97)	15 (1.42)	5 (0.49)

B. Association between acute anemia, compared to chronic anemia, and the outcome

Outcomes	Crude RR (95% CI)	Adjusted RR (95% CI)*
TACO & TRALI	1.10 (0.53-2.25)	1.16 (0.56-2.41)

C. Effect of the restrictive RBC transfusion strategy compared to the liberal one on the outcome

Outcomes	Total sample RR (95% CI)	Acute anemia RR (95% CI)	Chronic anemia RR (95% CI)	Interaction p value
TACO & TRALI	0.26 (0.12-0.61)	0.18 (0.04-0.83)	0.33 (0.12-0.89)	0.53

The model was adjusted for randomized transfusion strategy, demographics (age, sex, smoking [never, ever, current, unknown], and anemia-related comorbidities (cancer, renal failure, diabetes), pre-randomization characteristics (baseline hemoglobin [g/dL], number of anticoagulants [warfarin, heparin, other] and antiplatelets [aspirin, P2Y12, glycoprotein], MI type [type 1, type 2, other]) with a random effect for site.
The RERI for this outcome was -0.39 (95% CI -1.60 to 0.32)
TACO = Transfusion associated circulatory overload, TRALI = Transfusion related acute lung injury, RR = Risk Ratio, CI = Confidence Intervals.

© 2024 Carrier FM et al. *JAMA Network Open.*

eTable 6. Association between acute anemia and 30-day outcomes, restricted to participants hospitalized for 3 days or more at time of randomization.

30-day outcomes	Crude RR (95% CI)	Adjusted RR (95% CI)[a]
Death or recurrent MI	1.22 (0.98-1.52)	1.24 (0.98-1.56)
Death	1.59 (1.17-2.17)	1.60 (1.16-2.20)
Recurrent MI	0.91 (0.66-1.28)	0.95 (0.67-1.34)
Cardiac mortality	1.19 (0.76-1.86)	1.10 (0.69-1.75)
Heart failure	0.82 (0.57-1.18)	0.85 (0.58-1.23)
Pulmonary complications[b]	1.46 (1.12-1.89)	1.47 (1.12-1.93)
Major bleeding episode	1.19 (0.90-1.58)	1.14 (0.85-1.52)

Acute anemia (n=881) is compared to chronic anemia (n=806).
RR greater than 1 favor chronic anemia.
[a] The models were adjusted for randomized transfusion strategy, demographics (age, sex, smoking [never, ever, current, unknown], and anemia-related comorbidities (cancer, renal failure, diabetes), pre-randomization characteristics (baseline hemoglobin [g/dL], number of anticoagulants [warfarin, heparin, other] and antiplatelets [aspirin, P2Y12, glycoprotein], MI type [type 1, type 2, other]) with a random effect for site.
[b] Pulmonary complications = TRALI, TACO, pneumonia, or acute respiratory failure
RR = Risk ratio, CI = Confidence intervals, MI = Myocardial infarction, TACO = Transfusion associated circulatory overload, TRALI = Transfusion related acute lung injury.

© 2024 Carrier FM et al. *JAMA Network Open.*

eTable 7. Association between acute anemia and 30-day outcomes, restricted to participants who did not receive RBCs prior to randomization.

30-day outcomes	Crude RR (95% CI)	Adjusted RR (95% CI)[a]
Death or recurrent MI	1.17 (0.93-1.46)	1.15 (0.92-1.45)
Death	1.51 (1.12-2.03)	1.52 (1.12-2.07)
Recurrent MI	0.92 (0.65-1.31)	0.88 (0.61-1.26)
Cardiac mortality	1.12 (0.71-1.75)	0.96 (0.61-1.52)
Heart failure	0.90 (0.62-1.31)	0.95 (0.65-1.40)
Pulmonary complications[b]	1.14 (0.89-1.47)	1.20 (0.92-1.55)
Major bleeding episode	0.97 (0.71-1.33)	0.96 (0.69-1.32)

Acute anemia (n=541) is compared to chronic anemia (n=1,486).

[a] The models were adjusted for randomized transfusion strategy, demographics (age, sex, smoking [never, ever, current, unknown], and anemia-related comorbidities (cancer, renal failure, diabetes), pre-randomization characteristics (baseline hemoglobin [g/dL], number of anticoagulants [warfarin, heparin, other] and antiplatelets [aspirin, P2Y12, glycoprotein], MI type [type 1, type 2, other]) with a random effect for site.

[b] Pulmonary complications = TRALI, TACO, pneumonia, or acute respiratory failure

RR = Risk ratio, CI = Confidence intervals, MI = Myocardial infarction, TACO = Transfusion associated circulatory overload, TRALI = Transfusion related acute lung injury.

© 2024 Carrier FM et al. *JAMA Network Open.*

eFigure 1. Effect of a restrictive strategy, compared to a liberal strategy, on 30-day outcomes overall and stratified by anemia acuity, using an alternate definition of acute and chronic anemia.

30- Day Outcomes	Restrictive n / N (%)	Liberal n / N (%)	Risk Ratios RR (95% CI)	p-value (interaction)
Death/MI				0.73
Total	261/1551 (16.8%)	230/1593 (14.4%)	1.16 (0.98, 1.36)	
Chronic anemia	236/1395 (16.9%)	206/1410 (14.6%)	1.15 (0.97, 1.36)	
Acute anemia	25/156 (16.0%)	24/183 (13.1%)	1.26 (0.75, 2.11)	
Death				0.15
Total	148/1551 (9.5%)	132/1593 (8.3%)	1.16 (0.93, 1.45)	
Chronic anemia	127/1395 (9.1%)	118/1410 (8.4%)	1.09 (0.86, 1.38)	
Acute anemia	21/156 (13.5%)	14/183 (7.7%)	1.80 (0.95, 3.41)	
MI				0.16
Total	136/1551 (8.8%)	113/1593 (7.1%)	1.22 (0.96, 1.55)	
Chronic anemia	129/1395 (9.2%)	100/1410 (7.1%)	1.29 (1.00, 1.65)	
Acute anemia	7/156 (4.5%)	13/183 (7.1%)	0.66 (0.27, 1.60)	
Cardiac Death				0.23
Total	85/1551 (5.5%)	50/1593 (3.1%)	1.72 (1.23, 2.42)	
Chronic anemia	74/1395 (5.3%)	46/1410 (3.3%)	1.60 (1.11, 2.29)	
Acute anemia	11/156 (7.1%)	4/183 (2.2%)	3.30 (1.08, 10.14)	
Heart Failure				0.23
Total	93/1551 (6.0%)	105/1593 (6.6%)	0.89 (0.68, 1.16)	
Chronic anemia	88/1395 (6.3%)	93/1410 (6.6%)	0.94 (0.71, 1.24)	
Acute anemia	5/156 (3.2%)	12/183 (6.6%)	0.49 (0.18, 1.36)	
Pulmonary Complication				0.29
Total	185/1551 (11.9%)	199/1593 (12.5%)	0.98 (0.81, 1.18)	
Chronic anemia	161/1395 (11.5%)	175/1410 (12.4%)	0.94 (0.77, 1.15)	
Acute anemia	24/156 (15.4%)	24/183 (13.1%)	1.27 (0.76, 2.12)	
Bleeding event				0.54
Total	179/1551 (11.5%)	168/1593 (10.5%)	1.08 (0.89, 1.32)	
Chronic anemia	160/1395 (11.5%)	144/1410 (10.2%)	1.11 (0.90, 1.37)	
Acute anemia	19/156 (12.2%)	24/183 (13.1%)	0.92 (0.53, 1.61)	

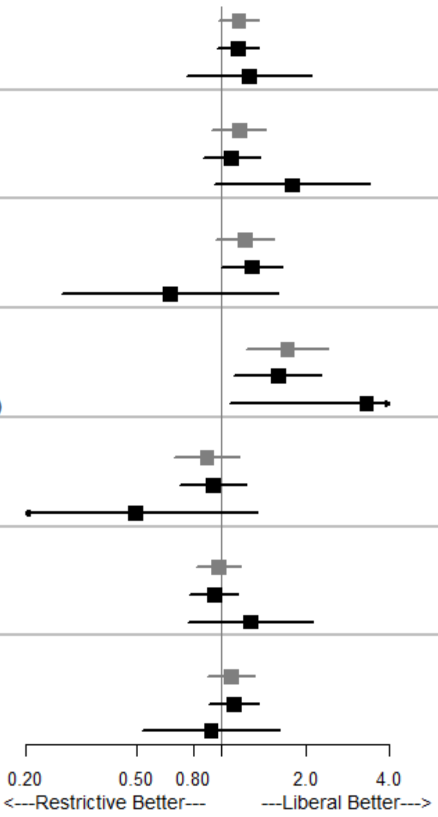

Legend:

n = 3,144
Pulmonary complications = TRALI, TACO, pneumonia, or acute respiratory failure
RR = Risk ratio, CI = Confidence intervals, MI = Myocardial infarction, TACO = Transfusion associated circulatory overload, TRALI = Transfusion related acute lung injury.
The models were adjusted for randomized transfusion strategy, demographics (age, sex, smoking [never, ever, current, unknown], and anemia-related comorbidities (cancer, renal failure, diabetes), pre-randomization characteristics (baseline hemoglobin [g/dL], number of anticoagulants [warfarin, heparin, other] and antiplatelets [aspirin, P2Y12, glycoprotein], MI type [type 1, type 2, other]) with a random effect for site.

© 2024 Carrier FM et al. *JAMA Network Open.*

eFigure 2. Effect of a restrictive strategy, compared to a liberal strategy, on 30-day outcomes overall and stratified by anemia acuity, restricted to participants hospitalized for 3 days or more at time of randomization.

30- Day Outcomes	Restrictive n / N (%)	Liberal n / N (%)	Risk Ratios RR (95% CI)	p-value (interaction)
Death/MI				0.12
Total	144/851 (16.9%)	127/836 (15.2%)	1.10 (0.88, 1.36)	
Chronic anemia	66/403 (16.4%)	50/403 (12.4%)	1.35 (0.96, 1.89)	
Acute anemia	78/448 (17.4%)	77/433 (17.8%)	0.95 (0.71, 1.26)	
Death				0.09
Total	86/851 (10.1%)	77/836 (9.2%)	1.08 (0.81, 1.44)	
Chronic anemia	35/403 (8.7%)	24/403 (6.0%)	1.53 (0.93, 2.52)	
Acute anemia	51/448 (11.4%)	53/433 (12.2%)	0.90 (0.63, 1.28)	
MI				0.68
Total	69/851 (8.1%)	59/836 (7.1%)	1.14 (0.82, 1.59)	
Chronic anemia	35/403 (8.7%)	29/403 (7.2%)	1.22 (0.76, 1.96)	
Acute anemia	34/448 (7.6%)	30/433 (6.9%)	1.07 (0.66, 1.71)	
Cardiac Death				0.44
Total	48/851 (5.6%)	28/836 (3.3%)	1.66 (1.06, 2.62)	
Chronic anemia	22/403 (5.5%)	11/403 (2.7%)	2.05 (1.01, 4.17)	
Acute anemia	26/448 (5.8%)	17/433 (3.9%)	1.42 (0.78, 2.58)	
Heart Failure				0.38
Total	50/851 (5.9%)	61/836 (7.3%)	0.79 (0.55, 1.14)	
Chronic anemia	28/403 (6.9%)	31/403 (7.7%)	0.92 (0.57, 1.50)	
Acute anemia	22/448 (4.9%)	30/433 (6.9%)	0.67 (0.39, 1.13)	
Pulmonary Complication				0.38
Total	102/851 (12.0%)	106/836 (12.7%)	0.94 (0.74, 1.21)	
Chronic anemia	36/403 (8.9%)	46/403 (11.4%)	0.82 (0.54, 1.23)	
Acute anemia	66/448 (14.7%)	60/433 (13.9%)	1.03 (0.75, 1.41)	
Bleeding event				0.23
Total	88/851 (10.3%)	94/836 (11.2%)	0.89 (0.68, 1.17)	
Chronic anemia	41/403 (10.2%)	38/403 (9.4%)	1.08 (0.71, 1.64)	
Acute anemia	47/448 (10.5%)	56/433 (12.9%)	0.77 (0.54, 1.11)	

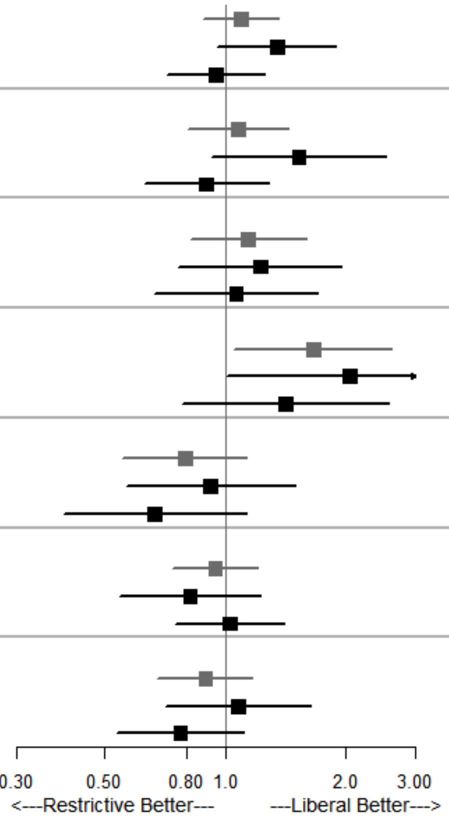

Legend:

n = 1,687
Pulmonary complications = TRALI, TACO, pneumonia, or acute respiratory failure
RR = Risk ratio, CI = Confidence intervals, MI = Myocardial infarction, TACO = Transfusion associated circulatory overload, TRALI = Transfusion related acute lung injury.
The models were adjusted for randomized transfusion strategy, demographics (age, sex, smoking [never, ever, current, unknown], and anemia-related comorbidities (cancer, renal failure, diabetes), pre-randomization characteristics (baseline hemoglobin [g/dL], number of anticoagulants [warfarin, heparin, other] and antiplatelets [aspirin, P2Y12, glycoprotein], MI type [type 1, type 2, other]) with a random effect for site.

© 2024 Carrier FM et al. *JAMA Network Open.*

eFigure 3. Effect of a restrictive strategy, compared to a liberal strategy, on 30-day outcomes overall and stratified by anemia acuity, restricted to participants who did not receive RBCs prior to randomization

Risk Ratios

30- Day Outcomes	Restrictive n / N (%)	Liberal n / N (%)	RR (95% CI)	p-value (interaction)
Death/MI				0.86
Total	174/1008 (17.3%)	136/1019 (13.3%)	1.28 (1.05, 1.58)	
Chronic anemia	120/732 (16.4%)	96/754 (12.7%)	1.30 (1.02, 1.66)	
Acute anemia	54/276 (19.6%)	40/265 (15.1%)	1.25 (0.86, 1.81)	
Death				0.64
Total	97/1008 (9.6%)	79/1019 (7.8%)	1.25 (0.94, 1.65)	
Chronic anemia	60/732 (8.2%)	53/754 (7.0%)	1.19 (0.83, 1.69)	
Acute anemia	37/276 (13.4%)	26/265 (9.8%)	1.36 (0.85, 2.18)	
MI				0.59
Total	89/1008 (8.8%)	65/1019 (6.4%)	1.37 (1.01, 1.87)	
Chronic anemia	67/732 (9.2%)	48/754 (6.4%)	1.44 (1.01, 2.06)	
Acute anemia	22/276 (8.0%)	17/265 (6.4%)	1.19 (0.64, 2.18)	
Cardiac Death				0.53
Total	60/1008 (6.0%)	29/1019 (2.8%)	2.07 (1.34, 3.19)	
Chronic anemia	43/732 (5.9%)	20/754 (2.7%)	2.25 (1.34, 3.78)	
Acute anemia	17/276 (6.2%)	9/265 (3.4%)	1.67 (0.76, 3.68)	
Heart Failure				0.18
Total	65/1008 (6.4%)	69/1019 (6.8%)	0.92 (0.66, 1.26)	
Chronic anemia	51/732 (7.0%)	49/754 (6.5%)	1.04 (0.72, 1.51)	
Acute anemia	14/276 (5.1%)	20/265 (7.5%)	0.62 (0.32, 1.20)	
Pulmonary Complication				0.64
Total	124/1008 (12.3%)	128/1019 (12.6%)	0.99 (0.79, 1.24)	
Chronic anemia	83/732 (11.3%)	93/754 (12.3%)	0.95 (0.72, 1.25)	
Acute anemia	41/276 (14.9%)	35/265 (13.2%)	1.07 (0.71, 1.61)	
Bleeding event				0.52
Total	101/1008 (10.0%)	82/1019 (8.0%)	1.22 (0.92, 1.61)	
Chronic anemia	72/732 (9.8%)	63/754 (8.4%)	1.16 (0.84, 1.59)	
Acute anemia	29/276 (10.5%)	19/265 (7.2%)	1.42 (0.82, 2.47)	

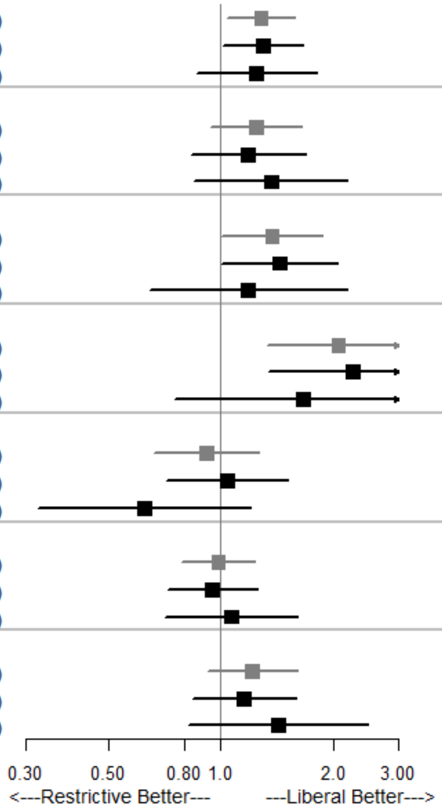

Legend:

n = 2,027
Pulmonary complications = TRALI, TACO, pneumonia, or acute respiratory failure
RR = Risk ratio, CI = Confidence intervals, MI = Myocardial infarction, TACO = Transfusion associated circulatory overload, TRALI = Transfusion related acute lung injury.
The models were adjusted for randomized transfusion strategy, demographics (age, sex, smoking [never, ever, current, unknown], and anemia-related comorbidities (cancer, renal failure, diabetes), pre-randomization characteristics (baseline hemoglobin [g/dL], number of anticoagulants [warfarin, heparin, other] and antiplatelets [aspirin, P2Y12, glycoprotein], MI type [type 1, type 2, other]) with a random effect for site.

© 2024 Carrier FM et al. *JAMA Network Open.*
