# Supplementary material for: Anemia Acuity Effect on Transfusion Strategies in Acute Myocardial Infarction: A Secondary Analysis of the MINT Trial
Source: JAMA Netw Open. 2024 Nov 1;7(11):e2442361. doi: 10.1001/jamanetworkopen.2024.42361 (PMC11530937; doi:10.1001/jamanetworkopen.2024.42361)
Supplement: Supplement 3. — Nonauthor Collaborators. MINT Investigators [file jamanetwopen-e2442361-s003.pdf]

Supplemental Online Content: Nonauthor Collaborators

*Indicates required information. Only first name, last name, and suffix will appear in PubMed.

***Group Name(s): MINT Investigators**

*First Name and Middle Initial(s)	*Last Name	*Suffix (eg, Jr, III)	Academic Degrees	Institution	Location (city, state/province, country)	Role or Contribution, eg, chair, principal investigator	Group (if more than 1 Group listed in the byline) and/or Subgroup (eg, Steering Committee)
Jeffrey L.	Carson			Rutgers Robert Wood Johnson Medical School	United States	Principal Investigator	Study Chair, Executive Committee, Steering Committee, Clinical Coordinating Center, Clinical Site
Fei	Chen			Rutgers Robert Wood Johnson Medical School	United States	Researcher	
Patrick	Desanto			Rutgers Robert Wood Johnson Medical School	United States	Coordinator	
Karen	Dragert			Rutgers Robert Wood Johnson Medical School	United States	Coordinator	Clinical Site and Clinical Coordinating Center
William J.	Kostis			Rutgers Robert Wood Johnson Medical School	United States	Co-Investigator	Steering Committee and Clinical Site
Sarang	Kim			Rutgers Robert Wood Johnson Medical School	United States	Medical Safety Officer	Clinical Coordinating Center
Deborah	McCloskey			Rutgers Robert Wood Johnson Medical School	United States	Research Staff	
Helaine	Noveck			Rutgers Robert Wood Johnson Medical School	United States	Coordinator	Clinical Coordinating Center, Clinical Site, Steering Committee
Sheila	Redding			Rutgers Robert Wood Johnson Medical School	United States	Coordinator	
Ami	Patel			Rutgers Robert Wood Johnson Medical School	United States	Coordinator	Clinical Coordinating Center and Clinical Site
Shahab	Ghafghazi			Louisville - University of Louisville , Jewish Hospital , Norton Hospital	United States	Principal Investigator	

Supplemental Online Content: Nonauthor Collaborators

*Indicates required information. Only first name, last name, and suffix will appear in PubMed.

***First Name and Middle Initial(s)**	***Last Name**	***Suffix (eg, Jr, III)**	Academic Degrees	Institution	Location (city, state/province, country)	Role or Contribution, eg, chair, principal investigator	Group (if more than 1 Group listed in the byline) and/or Subgroup (eg, Steering Committee)
Sharon	Vincent			Louisville - University of Louisville , Jewish Hospital , Norton Hospital	United States	Coordinator	
Hardeep	Dholiya			Louisville - University of Louisville , Jewish Hospital , Norton Hospital	United States	Coordinator	
Dinesh	Kalra			Louisville - University of Louisville , Jewish Hospital , Norton Hospital	United States	Co-Investigator	
Jennifer	Moore			Louisville - University of Louisville , Jewish Hospital , Norton Hospital	United States	Coordinator	
Mallory	Hatfield			Louisville - University of Louisville , Jewish Hospital , Norton Hospital	United States	Coordinator	
Howard A.	Cooper			Westchester Medical Center	United States	Principal Investigator	Clinical Site, Steering Committee
Syed	Haidry			Westchester Medical Center	United States	Researcher	
Ahmed	Hassanin			Westchester Medical Center	United States	Researcher	
Risheek	Kaul			Westchester Medical Center	United States	Research Staff	
Fnu	Namrata			Westchester Medical Center	United States	Coordinator	
Parija	Sharedalal			Westchester Medical Center	United States	Researcher	
Jayakumar	Sreenivasan			Westchester Medical Center	United States	Researcher	
Jasjit	Bhinder			Westchester Medical Center	United States	Researcher	
Mohammed	Hasan Khan			Westchester Medical Center	United States	Researcher	
Anna	Koulova			Westchester Medical Center	United States	Researcher	
Behram	Mody			Westchester Medical Center	United States	Researcher	
Yogita	Rochlani			Westchester Medical Center	United States	Researcher	
Anuritha	Tirumani			Westchester Medical Center	United States	Coordinator	
Frances	Wood			WakeMed Health & Hospitals	United States	Principal Investigator	

Supplemental Online Content: Nonauthor Collaborators

*Indicates required information. Only first name, last name, and suffix will appear in PubMed.

***First Name and Middle Initial(s)**	***Last Name**	***Suffix (eg, Jr, III)**	Academic Degrees	Institution	Location (city, state/province, country)	Role or Contribution, eg, chair, principal investigator	Group (if more than 1 Group listed in the byline) and/or Subgroup (eg, Steering Committee)
Rhonda	Norton			WakeMed Health & Hospitals	United States	Coordinator	
LaMonica	Daniel			WakeMed Health & Hospitals	United States	Coordinator	
Mark	Menegus			Montefiore - Weiler Hospital , Moses Hospital	United States	Principal Investigator	
Samanta	Baboolall			Montefiore - Weiler Hospital , Moses Hospital	United States	Coordinator	
Sheila	Davila			Montefiore - Weiler Hospital , Moses Hospital	United States	Research Staff	
Noelle	Manning			Montefiore - Weiler Hospital , Moses Hospital	United States	Researcher	
Mollie	Machado			Montefiore - Weiler Hospital , Moses Hospital	United States	Coordinator	
Judah	Rauch			Montefiore - Weiler Hospital , Moses Hospital	United States	Researcher	
Angeline	Camilo			Montefiore - Weiler Hospital , Moses Hospital	United States	Coordinator	
Estefania	Hernandez			Montefiore - Weiler Hospital , Moses Hospital	United States	Coordinator	
Muhammad	Iqbal			Montefiore - Weiler Hospital , Moses Hospital	United States	Coordinator	
Ervin	Mazniku			Montefiore - Weiler Hospital , Moses Hospital	United States	Coordinator	
Odilis	Ramirez			Montefiore - Weiler Hospital , Moses Hospital	United States	Coordinator	
Veronica	Day			Montefiore - Weiler Hospital , Moses Hospital	United States	Coordinator	
Barry	Uretsky			University of Arkansas Medical Sciences - Central Arkansas Veterans	United States	Principal Investigator	

*Indicates required information. Only first name, last name, and suffix will appear in PubMed.

*First Name and Middle Initial(s)	*Last Name	*Suffix (eg, Jr, III)	Academic Degrees	Institution	Location (city, state/province, country)	Role or Contribution, eg, chair, principal investigator	Group (if more than 1 Group listed in the byline) and/or Subgroup (eg, Steering Committee)
Jesse	Byrd			University of Arkansas Medical Sciences - Central Arkansas Veterans and University of Arkansas Medical	United States	Coordinator	
Kristin	Miller			University of Arkansas Medical Sciences - Central Arkansas Veterans and University of Arkansas Medical	United States	Coordinator	
Srikanth	Vallurupalli			University of Arkansas Medical Sciences - University of Arkansas Medical	United States	Principal Investigator	
Gregory	Maniatis			Northwell Hospital System - Staten Island University	United States	Principal Investigator	
Richard	Dima			Northwell Hospital System - Staten Island University	United States	Coordinator	
Roman	Royzman			Northwell Hospital System - Staten Island University	United States	Researcher	
Sean	Taylor			Northwell Hospital System - Staten Island University	United States	Coordinator	
Amanda	Tice			Northwell Hospital System - Staten Island University	United States	Researcher	
Brandon	Dilluvio			Northwell Hospital System - Staten Island University	United States	Coordinator	
Luis	Gruberg			Northwell Hospital System - Southside Hospital	United States	Principal Investigator	
Puneet	Gandotra			Northwell Hospital System - Southside Hospital	United States	Researcher	
Rachel	Gentles			Northwell Hospital System - Southside Hospital	United States	Coordinator	
Barbara	Shannon			Northwell Hospital System - Southside Hospital	United States	Coordinator	

*Indicates required information. Only first name, last name, and suffix will appear in PubMed.

***First Name and Middle Initial(s)**	***Last Name**	***Suffix (eg, Jr, III)**	Academic Degrees	Institution	Location (city, state/province, country)	Role or Contribution, eg, chair, principal investigator	Group (if more than 1 Group listed in the byline) and/or Subgroup (eg, Steering Committee)
Maria	Masih			Northwell Hospital System - Southside Hospital	United States	Coordinator	
Robert	Roswell			Northwell Hospital System - Lenox Hill Hospital	United States	Principal Investigator	
Alana	Gulliver			Northwell Hospital System - Lenox Hill Hospital	United States	Researcher	
Meriton	Ruhani			Northwell Hospital System - Lenox Hill Hospital	United States	Coordinator	
Emily	Strober			Northwell Hospital System - Lenox Hill Hospital	United States	Coordinator	
Samia	Tamazi			Northwell Hospital System - Lenox Hill Hospital	United States	Coordinator	
Adam	Gershon			Northwell Hospital System - Lenox Hill Hospital	United States	Researcher	
Kristie	Coleman			Northwell Hospital System - Lenox Hill Hospital	United States	Coordinator	
Bernard	Kadosh			Northwell Hospital System - Lenox Hill Hospital	United States	Researcher	
Rajiv	Jauhar			Northwell Hospital System - North Shore University Hospital	United States	Principal Investigator	
Harsha	Adnani			Northwell Hospital System - North Shore University Hospital	United States	Researcher	
Kyle	Agostini			Northwell Hospital System - North Shore University Hospital	United States	Coordinator	
Loukas	Boutis			Northwell Hospital System - North Shore University Hospital	United States	Researcher	
Ian	Dalangin			Northwell Hospital System - North Shore University Hospital	United States	Researcher	
Barry	Kaplan			Northwell Hospital System - North Shore University Hospital	United States	Researcher	

*Indicates required information. Only first name, last name, and suffix will appear in PubMed.

*First Name and Middle Initial(s)	*Last Name	*Suffix (eg, Jr, III)	Academic Degrees	Institution	Location (city, state/province, country)	Role or Contribution, eg, chair, principal investigator	Group (if more than 1 Group listed in the byline) and/or Subgroup (eg, Steering Committee)
Jae S.	Lee			Northwell Hospital System - North Shore University Hospital	United States	Coordinator	
Perwaiz	Meraj			Northwell Hospital System - North Shore University Hospital	United States	Researcher	
Riona	Timmons			Northwell Hospital System - North Shore University Hospital	United States	Researcher	
Carl	Reimers			Northwell Hospital System - Northern Westchester Hospital	United States	Principal Investigator	
Craig	Basman			Northwell Hospital System - Northern Westchester Hospital	United States	Co-Investigator	
Umar	Rashid			Northwell Hospital System - Northern Westchester Hospital	United States	Co-Investigator	
Aglae	Garvey Rene			Northwell Hospital System - Northern Westchester Hospital	United States	Researcher	
Asha	Mellor			Northwell Hospital System - Northern Westchester Hospital	United States	Coordinator	
Karen	duBourg			Northwell Hospital System - Northern Westchester Hospital	United States	Coordinator	
Joseph	Rossi			UNC Hospitals	United States	Principal Investigator	
Ryan	Orgel			UNC Hospitals	United States	Researcher	
Emma	Reynolds			UNC Hospitals	United States	Coordinator	
Tatum	Scholl			UNC Hospitals	United States	Researcher	
Janet	Bogan			UNC Hospitals	United States	Coordinator	
Olivia	Bogdan			UNC Hospitals	United States	Coordinator	
Matt	Cavender			UNC Hospitals	United States	Co-Investigator	
Gaetana	Cosentino			UNC Hospitals	United States	Researcher	
Gabriela	De Jesus			UNC Hospitals	United States	Researcher	
Megan	Gonzalez			UNC Hospitals	United States	Researcher	
Julia	Hoang			UNC Hospitals	United States	Researcher	
Emma	Kleemann			UNC Hospitals	United States	Researcher	

*Indicates required information. Only first name, last name, and suffix will appear in PubMed.

*First Name and Middle Initial(s)	*Last Name	*Suffix (eg, Jr, III)	Academic Degrees	Institution	Location (city, state/province, country)	Role or Contribution, eg, chair, principal investigator	Group (if more than 1 Group listed in the byline) and/or Subgroup (eg, Steering Committee)
Deanna	Ravenscraft			UNC Hospitals	United States	Coordinator	
Teresa	Reed			UNC Hospitals	United States	Researcher	
Joshua	Roark			UNC Hospitals	United States	Researcher	
Samantha	Smith			UNC Hospitals	United States	Researcher	
Kaitlyn	Sternat			UNC Hospitals	United States	Coordinator	
Oreyane	Tate			UNC Hospitals	United States	Researcher	
Tyrone	Wade			UNC Hospitals	United States	Coordinator	
Hope	Wolf			UNC Hospitals	United States	Coordinator	
Farhad	Abtahian			Rochester General Hospital	United States	Principal Investigator	
Megan	Littleton			Rochester General Hospital	United States	Researcher	
Kayla	Malchoff			Rochester General Hospital	United States	Researcher	
Kelly	Mohr			Rochester General Hospital	United States	Research Staff	
Emily	Pierce			Rochester General Hospital	United States	Researcher	
Jennifer	LaLonde			Rochester General Hospital	United States	Researcher	
Kellie	Sherron			Rochester General Hospital	United States	Research Staff	
Tia	Albro			Rochester General Hospital	United States	Co-Investigator	
Patty	Noll			Rochester General Hospital	United States	Coordinator	
Meechai	Tessalee			AdventHealth - Adventist Hinsdale Hospital , Adventist La Grange Hospital , Adventist Bolingbrook Hospital	United States	Principal Investigator	
Claudia	Eaton			AdventHealth - Adventist Hinsdale Hospital , Adventist La Grange Hospital , Adventist Bolingbrook Hospital	United States	Coordinator	
Gregory	Barsness			Mayo Clinic	United States	Principal Investigator	
Diana	Albers			Mayo Clinic	United States	Researcher	
Jacob	Jentzer			Mayo Clinic	United States	Researcher	

*Indicates required information. Only first name, last name, and suffix will appear in PubMed.

*First Name and Middle Initial(s)	*Last Name	*Suffix (eg, Jr, III)	Academic Degrees	Institution	Location (city, state/province, country)	Role or Contribution, eg, chair, principal investigator	Group (if more than 1 Group listed in the byline) and/or Subgroup (eg, Steering Committee)
J. Dawn	Abbott			Lifespan - Rhode Island Hospital	United States	Principal Investigator	Clinical Site and Steering Committee
Kelly	Franchetti			Lifespan - Rhode Island Hospital	United States	Coordinator	
Catherine	Gordon			Lifespan - Rhode Island Hospital	United States	Research Staff	
Herbert	Aronow			Lifespan - The Miriam Hospital	United States	Principal Investigator	
Ellen	Cerullo			Lifespan - The Miriam Hospital	United States	Coordinator	
Lori-Ann	Desimone			Lifespan - The Miriam Hospital	United States	Coordinator	
Elizabeth	Nowak			Lifespan - The Miriam Hospital	United States	Coordinator	
Elizabeth	Coccio			Lifespan - The Miriam Hospital	United States	Coordinator	
Kodangudi	Ramanathan			Memphis VA Medical Center	United States	Principal Investigator	
Adedayo	Adeboye			Memphis VA Medical Center	United States	Researcher	
Ashley	Armstrong			Memphis VA Medical Center	United States	Coordinator	
Lillie	Johnson			Memphis VA Medical Center	United States	Coordinator	
Anh-Phuong	Pham			Memphis VA Medical Center	United States	Researcher	
Rahman	Shah			Memphis VA Medical Center	United States	Co-Investigator	
Mark	Schmidhofer			UPMC Presbyterian	United States	Principal Investigator	
Mary Jo	Albright			UPMC Presbyterian	United States	Coordinator	
Melissa	Enlow			UPMC Presbyterian	United States	Coordinator	
Barinder	Hansra			UPMC Presbyterian	United States	Researcher	
Tracy	Nicholson			UPMC Presbyterian	United States	Coordinator	
Catalin	Toma			UPMC Presbyterian	United States	Co-Investigator	
Darrell	Triulzi			UPMC Presbyterian	United States	Co-Investigator	
Megan	Basch			UPMC Presbyterian	United States	Coordinator	
Gretchen	Bernardini			UPMC Presbyterian	United States	Coordinator	
Amanda	Bollino			UPMC Presbyterian	United States	Coordinator	
Laurie	Dennis			UPMC Presbyterian	United States	Coordinator	

*Indicates required information. Only first name, last name, and suffix will appear in PubMed.

***First Name and Middle Initial(s)**	***Last Name**	***Suffix (eg, Jr, III)**	Academic Degrees	Institution	Location (city, state/province, country)	Role or Contribution, eg, chair, principal investigator	Group (if more than 1 Group listed in the byline) and/or Subgroup (eg, Steering Committee)
Kathy	Puntil			UPMC Presbyterian	United States	Researcher	
Chelsey	Snyder			UPMC Presbyterian	United States	Coordinator	
Linda	Spearman			UPMC Presbyterian	United States	Researcher	
Jenny	Whitehead			UPMC Presbyterian	United States	Coordinator	
Friederike	Keating			University of Vermont Medical	United States	Principal Investigator	
Bhisham	Harchandani			University of Vermont Medical	United States	Researcher	
Meghan	Sesera			University of Vermont Medical	United States	Researcher	
Jonathan	Halevy			University of Vermont Medical	United States	Researcher	
Molly	Raftery			University of Vermont Medical	United States	Coordinator	
Marilynn	Roth			University of Vermont Medical	United States	Coordinator	
David	Kountz			Jersey Shore University Medical	United States	Principal Investigator	
Michael	Carson			Jersey Shore University Medical	United States	Principal Investigator	
Lynda	Argenzio			Jersey Shore University Medical	United States	Coordinator	
Michael	Kontos			Virginia Commonwealth	United States	Principal Investigator	
Melissa	Sears			Virginia Commonwealth	United States	Coordinator	
Laura	Johnson			Virginia Commonwealth	United States	Coordinator	
Mansoor	Qureshi			St. Joseph Mercy Hospital	United States	Principal Investigator	
Ahmad	Mizyed			St. Joseph Mercy Hospital	United States	Co-Investigator	
Sarah	Whitsett			St. Joseph Mercy Hospital	United States	Coordinator	
Kristina	Wippler			St. Joseph Mercy Hospital	United States	Coordinator	
Jay	Raval			University of New Mexico	United States	Principal Investigator	
Stacey	Clegg			University of New Mexico	United States	Principal Investigator	
Warren	Laskey			University of New Mexico	United States	Principal Investigator	

*Indicates required information. Only first name, last name, and suffix will appear in PubMed.

***First Name and Middle Initial(s)**	***Last Name**	***Suffix (eg, Jr, III)**	Academic Degrees	Institution	Location (city, state/province, country)	Role or Contribution, eg, chair, principal investigator	Group (if more than 1 Group listed in the byline) and/or Subgroup (eg, Steering Committee)
Judith	Mondragon			University of New Mexico	United States	Coordinator	
Tamar	Polonsky			University of Chicago	United States	Principal Investigator	
Matthew	Carlson			University of Chicago	United States	Researcher	
Catherine	Drovetsky			University of Chicago	United States	Coordinator	
Joseph	Gutbrod			University of Chicago	United States	Coordinator	
Micah	Prochaska			University of Chicago	United States	Co-Investigator	
Maud	Jansen			University of Chicago	United States	Coordinator	
Tharani	Jeyaram			University of Chicago	United States	Coordinator	
Noora	Reffat			University of Chicago	United States	Coordinator	
Rajesh	Gupta			University of Toledo	United States	Principal Investigator	
Mujeeb A.	Sheikh			University of Toledo	United States	Principal Investigator	
Meghan	(Redd) Moore			University of Toledo	United States	Coordinator	
Kristin	Fisher			University of Toledo	United States	Research Staff	
Jennifer	Gilmore			University of Toledo	United States	Coordinator	
Christina	Sattler			University of Toledo	United States	Coordinator	
Stephanie	Frank			University of Toledo	United States	Coordinator	
Lynne	Uhl			Beth Israel Deaconess	United States	Principal Investigator	
Kalon	Ho			Beth Israel Deaconess	United States	Co-Investigator	
Hannah	Hunsaker			Beth Israel Deaconess	United States	Researcher	
Jenifer	Kaufman			Beth Israel Deaconess	United States	Coordinator	
Lauren	Lanuto			Beth Israel Deaconess	United States	Researcher	
Jordan	Strom			Beth Israel Deaconess	United States	Co-Investigator	
Patricia	Tyler			Beth Israel Deaconess	United States	Coordinator	
Paul	Mullen			Memorial Hospital at Gulfport	United States	Principal Investigator	
Jennifer	Ratcliffe			Memorial Hospital at Gulfport	United States	Coordinator	
Brandy	Williams			Memorial Hospital at Gulfport	United States	Coordinator	

*Indicates required information. Only first name, last name, and suffix will appear in PubMed.

*First Name and Middle Initial(s)	*Last Name	*Suffix (eg, Jr, III)	Academic Degrees	Institution	Location (city, state/province, country)	Role or Contribution, eg, chair, principal investigator	Group (if more than 1 Group listed in the byline) and/or Subgroup (eg, Steering Committee)
Arthur	Bracey			Baylor St Luke's	United States	Principal Investigator	
Araceli	Boan			Baylor St Luke's	United States	Coordinator	
Gilberto	De Freitas			Baylor St Luke's	United States	Coordinator	
Samantha	Macias			Baylor St Luke's	United States	Coordinator	
George	Younis			Baylor St Luke's	United States	Researcher	
William	Matthai			PENN Presbyterian	United States	Principal Investigator	
Sameer	Khandhar			PENN Presbyterian	United States	Co-Investigator	
Karen	Maslowski			PENN Presbyterian	United States	Coordinator	
Nilay	Patel			Massachusetts General Hospital	United States	Principal Investigator	
Sammy	Elmariah			Massachusetts General Hospital	United States	Principal Investigator	
Christopher	Stowell			Massachusetts General Hospital	United States	Principal Investigator	
David	Dudzinski			Massachusetts General Hospital	United States	Principal Investigator	
Roukoz	Abou-Karam			Massachusetts General Hospital	United States	Coordinator	
Lina	Fu			Massachusetts General Hospital	United States	Coordinator	
Paris	Jamiel			Massachusetts General Hospital	United States	Coordinator	
Cristina	Brueggeman			Massachusetts General Hospital	United States	Coordinator	
Gregary	Marhefka			Thomas Jefferson University	United States	Principal Investigator	
Blaire	Cain			Thomas Jefferson University	United States	Researcher	
Barbara	Gallagher			Thomas Jefferson University	United States	Coordinator	
Perry	Weinstock			Cooper University Hospital	United States	Principal Investigator	
Janah	Aji			Cooper University Hospital	United States	Co-Investigator	
Brian	Corbett			Cooper University Hospital	United States	Co-Investigator	
Elias	Iliadis			Cooper University Hospital	United States	Co-Investigator	

*Indicates required information. Only first name, last name, and suffix will appear in PubMed.

***First Name and Middle Initial(s)**	***Last Name**	***Suffix (eg, Jr, III)**	Academic Degrees	Institution	Location (city, state/province, country)	Role or Contribution, eg, chair, principal investigator	Group (if more than 1 Group listed in the byline) and/or Subgroup (eg, Steering Committee)
Patricia	Niblack			Cooper University Hospital	United States	Coordinator	
Jeffrey	Ogbara			Cooper University Hospital	United States	Researcher	
Simon	Topalian			Cooper University Hospital	United States	Co-Investigator	
Leana	Abreu			Cooper University Hospital	United States	Coordinator	
Amanda	Logan			Cooper University Hospital	United States	Coordinator	
Amanda	Salvatore			Cooper University Hospital	United States	Researcher	
Erin	Siderio			Cooper University Hospital	United States	Researcher	
William	Lawson			Stony Brook University	United States	Principal Investigator	
Ruth J.	Stein			Stony Brook University	United States	Coordinator	
Samantha	Weber-Fishkin			Stony Brook University	United States	Coordinator	
Norma	Keller			NYU Langone Medical Center	United States	Principal Investigator	
Eugene	Yuriditsky			NYU Langone Medical Center	United States	Principal Investigator	
Carlos L.	Alviar			NYU Langone Medical Center	United States	Co-Investigator	
Sripal	Bangalore			NYU Langone Medical Center	United States	Co-Investigator	
Maria	Daly			NYU Langone Medical Center	United States	Coordinator	
Dharshini	Sivakumaran			NYU Langone Medical Center	United States	Coordinator	
Raven	Dwyer			NYU Langone Medical Center	United States	Coordinator	
Carolina	Rodriguez Alvarez			NYU Langone Medical Center	United States	Coordinator	
Shuaib	Ahmad			NYU Langone Medical Center	United States	Coordinator	
Michael	Thomas			University of Michigan	United States	Principal Investigator	
Allison	Schley			University of Michigan	United States	Coordinator	
Gau Shoua	Vue			University of Michigan	United States	Coordinator	
Claudia	Hochberg			Boston Medical Center	United States	Principal Investigator	

*Indicates required information. Only first name, last name, and suffix will appear in PubMed.

***First Name and Middle Initial(s)**	***Last Name**	***Suffix (eg, Jr, III)**	Academic Degrees	Institution	Location (city, state/province, country)	Role or Contribution, eg, chair, principal investigator	Group (if more than 1 Group listed in the byline) and/or Subgroup (eg, Steering Committee)
Omar	Siddiqi			Boston Medical Center	United States	Principal Investigator	
Denise	Fine			Boston Medical Center	United States	Coordinator	
Alice	Jacobs			Boston Medical Center	United States	Co-Investigator	
Joshua	Schulman-Marcus			Albany Medical College	United States	Principal Investigator	
Mikhail	Torosoff			Albany Medical College	United States	Principal Investigator	
Wendy	Stewart			Albany Medical College	United States	Coordinator	
Michael	Gitter			Medical College of Wisconsin	United States	Principal Investigator	
Barbara	Shimada-Krouwer			Medical College of Wisconsin	United States	Coordinator	
Leanne	Harmann			Medical College of Wisconsin	United States	Researcher	
Matthew	Karafin			Medical College of Wisconsin	United States	Co-Investigator	
Pranitha	Patlolla			Medical College of Wisconsin	United States	Coordinator	
Julie	Werra			Medical College of Wisconsin	United States	Coordinator	
Mary	Wexler			Medical College of Wisconsin	United States	Researcher	
Xuming	Dai			NYP Queens	United States	Principal Investigator	
Jackson	Ng			NYP Queens	United States	Coordinator	
Asif	Adam			NYP Queens	United States	Coordinator	
Ajini	Cherian			NYP Queens	United States	Coordinator	
Jay	Traverse			Minneapolis Heart Institute	United States	Principal Investigator	
Carina	Benson			Minneapolis Heart Institute	United States	Coordinator	
Rose	Peterson			Minneapolis Heart Institute	United States	Coordinator	
Amy	McMeans			Minneapolis Heart Institute	United States	Researcher	
Elizabeth	Reedy			Minneapolis Heart Institute	United States	Researcher	
Andrea	Sarafolean			Minneapolis Heart Institute	United States	Coordinator	

*Indicates required information. Only first name, last name, and suffix will appear in PubMed.

*First Name and Middle Initial(s)	*Last Name	*Suffix (eg, Jr, III)	Academic Degrees	Institution	Location (city, state/province, country)	Role or Contribution, eg, chair, principal investigator	Group (if more than 1 Group listed in the byline) and/or Subgroup (eg, Steering Committee)
Eric	McCamant			Lancaster General	United States	Principal Investigator	
Jason	Scott			Lancaster General	United States	Principal Investigator	
Michelle	Jordan			Lancaster General	United States	Researcher	
Kay	Knepper			Lancaster General	United States	Coordinator	
Jennifer	Nguyen			Lancaster General	United States	Research Staff	
Deborah	Ramsey			Lancaster General	United States	Coordinator	
Tara	Tawil			Lancaster General	United States	Researcher	
Todd	Wood			Lancaster General	United States	Researcher	
Dhvni	Dave			Lancaster General	United States	Coordinator	
Heidi	Testa			Lancaster General	United States	Coordinator	
Rajesh	Swaminathan			Durham VA Medical Center	United States	Principal Investigator	
Sunil	Rao			Durham VA Medical Center / NYU Langone Health	United States	Principal Investigator	Steering Committee and Clinical Site
Kathy	Aristy			Durham VA Medical Center	United States	Coordinator	
Mitchell	Krucoff			Durham VA Medical Center	United States	Co-Investigator	
Thomas	Povsic			Durham VA Medical Center	United States	Co-Investigator	
Andrew M.	Goldsweig			University of Nebraska Medical Center	United States	Principal Investigator	Clinical Site and Steering Committee
Shahbaz	Malik			University of Nebraska Medical Center	United States	Principal Investigator	
Yiannis	Chatzizisis			University of Nebraska Medical Center	United States	Researcher	
John	Higgins			University of Nebraska Medical Center	United States	Researcher	
Poonam	Velagapudi			University of Nebraska Medical Center	United States	Researcher	
David	Barton			University of Nebraska Medical Center	United States	Researcher	

*Indicates required information. Only first name, last name, and suffix will appear in PubMed.

*First Name and Middle Initial(s)	*Last Name	*Suffix (eg, Jr, III)	Academic Degrees	Institution	Location (city, state/province, country)	Role or Contribution, eg, chair, principal investigator	Group (if more than 1 Group listed in the byline) and/or Subgroup (eg, Steering Committee)
Jeremy	Stone			University of Nebraska Medical Center	United States	Researcher	
Samer	Sayyed			University of Nebraska Medical Center	United States	Researcher	
Arav	Jhand			University of Nebraska Medical Center	United States	Researcher	
Daniel	Johnson			University of Nebraska Medical Center	United States	Researcher	
James	Sullivan			University of Nebraska Medical Center	United States	Researcher	
Furquan	Khattak			University of Nebraska Medical Center	United States	Researcher	
Kara	Stout			University of Nebraska Medical Center	United States	Researcher	
Jennifer	Nickol			University of Nebraska Medical Center	United States	Co-Investigator	
David	Shin			University of Nebraska Medical Center	United States	Co-Investigator	
Mansi	Oberoi			University of Nebraska Medical Center	United States	Co-Investigator	
Taylor	Johnson			University of Nebraska Medical Center	United States	Coordinator	
Melissa	Howard			University of Nebraska Medical Center	United States	Researcher	
Zaria	Starfeldt			University of Nebraska Medical Center	United States	Coordinator	
Ryan	Ruskamp			University of Nebraska Medical Center	United States	Coordinator	
Nathan	Muhn			University of Nebraska Medical Center	United States	Coordinator	

*Indicates required information. Only first name, last name, and suffix will appear in PubMed.

*First Name and Middle Initial(s)	*Last Name	*Suffix (eg, Jr, III)	Academic Degrees	Institution	Location (city, state/province, country)	Role or Contribution, eg, chair, principal investigator	Group (if more than 1 Group listed in the byline) and/or Subgroup (eg, Steering Committee)
Camila	Pacheco			University of Nebraska Medical Center	United States	Coordinator	
Tyler	Patterson			University of Nebraska Medical Center	United States	Researcher	
Angie	Adler			University of Nebraska Medical Center	United States	Researcher	
Andrew P.	DeFilippis			Vanderbilt	United States	Principal Investigator	Clinical Site and Steering Committee
Karita	Lidani			Vanderbilt	United States	Coordinator	
Cassandra	Reynolds			Vanderbilt	United States	Researcher	
Nicholas	Brooks			Vanderbilt	United States	Coordinator	
Stephan	Bierenga			Vanderbilt	United States	Coordinator	
Adam C.	Salisbury			Saint Luke's Mid America Heart	United States	Principal Investigator	
Jamie	Hall			Saint Luke's Mid America Heart	United States	Coordinator	
David	Landers			Hackensack University Medical	United States	Principal Investigator	
Robert	Hendel			Tulane Medical Center	United States	Principal Investigator	
Wilson	Ginete			Essentia Health	United States	Principal Investigator	
Ganesh	Raveendran			University of Minnesota	United States	Principal Investigator	
Craig	Hudak			Greenville Health System	United States	Principal Investigator	
Ramin	Ebrahimi			UCLA / Veterans Affairs Greater LA	United States	Principal Investigator	
Richard	Bach			Washington University St Louis	United States	Principal Investigator	
Margie	Palazzolo			Washington University St Louis	United States	Coordinator	
Kimberly	Striler			Washington University St Louis	United States	Researcher	

*Indicates required information. Only first name, last name, and suffix will appear in PubMed.

***First Name and Middle Initial(s)**	***Last Name**	***Suffix (eg, Jr, III)**	Academic Degrees	Institution	Location (city, state/province, country)	Role or Contribution, eg, chair, principal investigator	Group (if more than 1 Group listed in the byline) and/or Subgroup (eg, Steering Committee)
Paolo	Mascari			Lahey Hospital	United States	Principal Investigator	
Hal	Wasserman			Danbury Hospital	United States	Principal Investigator	
Joseph	Delehanty			University of Rochester	United States	Principal Investigator	
Raj C.	Shah			Rush University Medical Center	United States	Principal Investigator	
Lesley A.	Schmaltz			Rush University Medical Center	United States	Researcher	
Steve	Attanasio			Rush University Medical Center	United States	Researcher	
Sorin	Brener			NYP Brooklyn Methodist	United States	Principal Investigator	
Brendan	Duffy			Aultman Cardiology	United States	Principal Investigator	
Paul	Grunenwald			Alexian Brothers Medical Center - Alexian Brothers Medical , St. Alexius Medical Center	United States	Principal Investigator	
Jonathan	Doroshow			Lankenau Medical Center	United States	Principal Investigator	
Muhammad Abubakar	Shakir			Lankenau Medical Center	United States	Researcher	
Adriano	Caixeta			Hospital São Paulo	Brazil	Principal Investigator	
Attilio	Galhardo			Hospital São Paulo	Brazil	Coordinator	
Paula	Santiago Teixeira			Hospital São Paulo	Brazil	Co-Investigator	
Joyce	Umbelino S. Yamamoto			Hospital São Paulo	Brazil	Coordinator	
Dalton	Precoma			Sociedade Hospitalar Angelina Caron	Brazil	Principal Investigator	

*Indicates required information. Only first name, last name, and suffix will appear in PubMed.

***First Name and Middle Initial(s)**	***Last Name**	***Suffix (eg, Jr, III)**	Academic Degrees	Institution	Location (city, state/province, country)	Role or Contribution, eg, chair, principal investigator	Group (if more than 1 Group listed in the byline) and/or Subgroup (eg, Steering Committee)
Cristina	Correa			Sociedade Hospitalar Angelina Caron	Brazil	Researcher	
Maynara	Martins			Sociedade Hospitalar Angelina Caron	Brazil	Coordinator	
Carlos	Rochitti			Sociedade Hospitalar Angelina Caron	Brazil	Researcher	
Camila	Richter			Sociedade Hospitalar Angelina Caron	Brazil	Researcher	
Carlos A.K.	Nakashima			Sociedade Hospitalar Angelina Caron	Brazil	Researcher	
Eliane	Sehnem			Sociedade Hospitalar Angelina Caron	Brazil	Researcher	
Frederico	Toledo Campo Dall'Orto			Hospital Maternidade e Pronto Socorro Santa Lucia	Brazil	Principal Investigator	
Gislayne	Rogante Ribeiro			Hospital Maternidade e Pronto Socorro Santa Lucia	Brazil	Coordinator	
Ricardo Reinaldo	Bergo			Hospital Maternidade e Pronto Socorro Santa Lucia	Brazil	Researcher	
Claire Marie	Pedroso Dias Corsini			Hospital Maternidade e Pronto Socorro Santa Lucia	Brazil	Researcher	
Kemilys Marine	Ferreira			Hospital Maternidade e Pronto Socorro Santa Lucia	Brazil	Researcher	
Pedro B.	De Andrade			Santa Casa de Marília	Brazil	Principal Investigator	
Robson A.	Barbosa			Santa Casa de Marília	Brazil	Researcher	
Marianna	Dracoulakis			Hospital da Bahia	Brazil	Principal Investigator	
Natalia	Alvaia			Hospital da Bahia	Brazil	Coordinator	
Rodolfo	Dourado			Hospital da Bahia	Brazil	Co-Investigator	
Jéssica	Ribeiro			Hospital da Bahia	Brazil	Researcher	
Camilla	Vieira			Hospital da Bahia	Brazil	Coordinator	

*Indicates required information. Only first name, last name, and suffix will appear in PubMed.

*First Name and Middle Initial(s)	*Last Name	*Suffix (eg, Jr, III)	Academic Degrees	Institution	Location (city, state/province, country)	Role or Contribution, eg, chair, principal investigator	Group (if more than 1 Group listed in the byline) and/or Subgroup (eg, Steering Committee)
Taís	Sarmento			Hospital da Bahia	Brazil	Researcher	
Aline	Giacomo			Hospital da Bahia	Brazil	Researcher	
Mariana	Lopes			Hospital da Bahia	Brazil	Researcher	
Lília N.	Maia			Hospital de Base de Rio Preto	Brazil	Principal Investigator	
Osana	Costa			Hospital de Base de Rio Preto	Brazil	Researcher	
Paulo Jhones	Dutra			Hospital de Base de Rio Preto	Brazil	Researcher	
Danieli	Frassatto			Hospital de Base de Rio Preto	Brazil	Researcher	
Juliana Vieira	Garcia			Hospital de Base de Rio Preto	Brazil	Researcher	
Maria Angélica	Lemos			Hospital de Base de Rio Preto	Brazil	Researcher	
Thaise	Loverdi			Hospital de Base de Rio Preto	Brazil	Researcher	
Marcelo	Nakazone			Hospital de Base de Rio Preto	Brazil	Researcher	
Nadielly	Prado			Hospital de Base de Rio Preto	Brazil	Researcher	
Osvaldo	Da Silva Júnior			Hospital de Base de Rio Preto	Brazil	Researcher	
Natalia	Cordeiro da Silva			Hospital de Base de Rio Preto	Brazil	Researcher	
Sidney	Gomes			Hospital de Base de Rio Preto	Brazil	Researcher	
Anna Helena Felipe Pereira	Centurione			Hospital de Base de Rio Preto	Brazil	Researcher	
Luiz Eduardo	Fontelles Ritt			Hospital Cárdio Pulmonar	Brazil	Principal Investigator	
Queila	Borges De Oliveira			Hospital Cárdio Pulmonar	Brazil	Researcher	
Karina	De Carvalho Cordeiro			Hospital Cárdio Pulmonar	Brazil	Researcher	
Alexandre	Quadros			Instituto de Cardiologia do RS	Brazil	Principal Investigator	
Giulia	Bonatto			Instituto de Cardiologia do RS	Brazil	Co-Investigator	
Bruna	Machado			Instituto de Cardiologia do RS	Brazil	Coordinator	
Silvia	Poleth			Instituto de Cardiologia do RS	Brazil	Researcher	
Camille	Correia			Instituto de Cardiologia do RS	Brazil	Researcher	

*Indicates required information. Only first name, last name, and suffix will appear in PubMed.

*First Name and Middle Initial(s)	*Last Name	*Suffix (eg, Jr, III)	Academic Degrees	Institution	Location (city, state/province, country)	Role or Contribution, eg, chair, principal investigator	Group (if more than 1 Group listed in the byline) and/or Subgroup (eg, Steering Committee)
Raphael	Guimarães			Instituto de Cardiologia do RS	Brazil	Researcher	
Israel	Cabral			Instituto de Cardiologia do RS	Brazil	Researcher	
Dário Celestino	Sobral Filho			Pronto Socorro Cardiológico de Prof. Luiz Tavares	Brazil	Principal Investigator	
Maria Cleide Freire Clementino	Silva			Pronto Socorro Cardiológico de Prof. Luiz Tavares	Brazil	Researcher	
Osmário	Tavares De Carvalho			Pronto Socorro Cardiológico de Prof. Luiz Tavares	Brazil	Researcher	
José Gildo	De Moura Monteiro Júnior			Pronto Socorro Cardiológico de Prof. Luiz Tavares	Brazil	Researcher	
Sergio	Nascimento			Pronto Socorro Cardiológico de Prof. Luiz Tavares	Brazil	Researcher	
Fernando	De Martino			Hospital de Clinicas UFTM	Brazil	Principal Investigator	
Paula M.	Camasmie			Hospital de Clinicas UFTM	Brazil	Coordinator	
Leonilda	Castanho Da Rocha			Hospital de Clinicas UFTM	Brazil	Researcher	
Pâmela Joice R.	De Oliveira Assunção			Hospital de Clinicas UFTM	Brazil	Researcher	
Fábio	Rodrigues de Oliveira			Hospital de Clinicas UFTM	Brazil	Researcher	
Paulo H.M.	Vilela			Hospital de Clinicas UFTM	Brazil	Researcher	
Rodrigo Cunha	de Sousa			Hospital de Clinicas UFTM	Brazil	Researcher	
Estevão L.	Figueiredo			Instituto Orizonti - NUPEC	Brazil	Principal Investigator	
Fernando Carvalho	Neuenschwander			Instituto Orizonti - NUPEC	Brazil	Researcher	
Cristina Carvalho	Neuenschwander			Instituto Orizonti - NUPEC	Brazil	Researcher	
Izabela R.	Falco			Instituto Orizonti - NUPEC	Brazil	Coordinator	
Arlete	Matos			Instituto Orizonti - NUPEC	Brazil	Researcher	

Supplemental Online Content: Nonauthor Collaborators

*Indicates required information. Only first name, last name, and suffix will appear in PubMed.

*First Name and Middle Initial(s)	*Last Name	*Suffix (eg, Jr, III)	Academic Degrees	Institution	Location (city, state/province, country)	Role or Contribution, eg, chair, principal investigator	Group (if more than 1 Group listed in the byline) and/or Subgroup (eg, Steering Committee)
Thao	Huynh			Montreal General Hospital	Canada	Principal Investigator	
Nancy	Fuentes			Montreal General Hospital	Canada	Researcher	
Amanda	Barnett			Montreal General Hospital	Canada	Researcher	
Caroline	Boudreault			Montreal General Hospital	Canada	Coordinator	
Greg	Schnell			Foothills Medical Centre	Canada	Principal Investigator	
Leslie	Poirier			Foothills Medical Centre	Canada	Coordinator	
Darlene	Ramadan			Foothills Medical Centre	Canada	Coordinator	
Manohara	Senaratne			Grey Nuns Community Hospital	Canada	Principal Investigator	
Bernadette	Fernando			Grey Nuns Community Hospital	Canada	Coordinator	
Mike	Hanninen			Grey Nuns Community Hospital	Canada	Co-Investigator	
Anushka	Jayasekara			Grey Nuns Community Hospital	Canada	Researcher	
Janek	Senaratne			Grey Nuns Community Hospital and University Of Alberta Hospital	Canada	Co-Investigator	
Lalantha	Coonghe			Grey Nuns Community Hospital	Canada	Coordinator	
Sharada	Manchikanti			Grey Nuns Community Hospital	Canada	Coordinator	
Gamintha	Nanayakkara			Grey Nuns Community Hospital	Canada	Coordinator	
Vikas	Tandon			Hamilton Health Sciences/St. Joseph's Healthcare Hamilton - Hamilton General Hospital and Juravinski Hospital	Canada	Principal Investigator	
Patricia	Power			Hamilton Health Sciences/St. Joseph's Healthcare Hamilton - Hamilton General Hospital	Canada	Coordinator	
Ryan	Proc			Hamilton Health Sciences/St. Joseph's Healthcare Hamilton - Hamilton General Hospital	Canada	Researcher	
Kajenny	Srivaratharajah			Hamilton Health Sciences/St. Joseph's Healthcare Hamilton - Hamilton General Hospital	Canada	Co-Investigator	

Supplemental Online Content: Nonauthor Collaborators

*Indicates required information. Only first name, last name, and suffix will appear in PubMed.

***First Name and Middle Initial(s)**	***Last Name**	***Suffix (eg, Jr, III)**	Academic Degrees	Institution	Location (city, state/province, country)	Role or Contribution, eg, chair, principal investigator	Group (if more than 1 Group listed in the byline) and/or Subgroup (eg, Steering Committee)
Lisa	Trombetta			Hamilton Health Sciences/St. Joseph's Healthcare Hamilton - Hamilton General Hospital	Canada	Coordinator	
Pablo	Mendoza			Hamilton Health Sciences/St. Joseph's Healthcare Hamilton - Hamilton General Hospital	Canada	Researcher	
Krysten	Gregus			Hamilton Health Sciences/St. Joseph's Healthcare Hamilton - Juravinski Hospital	Canada	Coordinator	
Jacqueline	Hare			Hamilton Health Sciences/St. Joseph's Healthcare Hamilton - Juravinski Hospital	Canada	Coordinator	
Kelly	Lawrence			Hamilton Health Sciences/St. Joseph's Healthcare Hamilton - Juravinski Hospital	Canada	Coordinator	
Ameen	Patel			Hamilton Health Sciences/St. Joseph's Healthcare Hamilton - Juravinski Hospital	Canada	Co-Investigator	
Shirley	Pettit			Hamilton Health Sciences/St. Joseph's Healthcare Hamilton - Juravinski Hospital	Canada	Researcher	
Jocelyn	Kuber			Hamilton Health Sciences/St. Joseph's Healthcare Hamilton - Juravinski Hospital	Canada	Coordinator	
Summer	Nasr			Hamilton Health Sciences/St. Joseph's Healthcare Hamilton - Juravinski Hospital	Canada	Coordinator	
John	Neary			Hamilton Health Sciences/St. Joseph's Healthcare Hamilton - St. Joseph's Healthcare Hamilton	Canada	Principal Investigator	

Supplemental Online Content: Nonauthor Collaborators

*Indicates required information. Only first name, last name, and suffix will appear in PubMed.

***First Name and Middle Initial(s)**	***Last Name**	***Suffix (eg, Jr, III)**	Academic Degrees	Institution	Location (city, state/province, country)	Role or Contribution, eg, chair, principal investigator	Group (if more than 1 Group listed in the byline) and/or Subgroup (eg, Steering Committee)
Muammar	Abdulrahman			Hamilton Health Sciences/St. Joseph's Healthcare Hamilton - St. Joseph's Healthcare Hamilton	Canada	Coordinator	
David	Cohen			Hamilton Health Sciences/St. Joseph's Healthcare Hamilton - St. Joseph's Healthcare Hamilton	Canada	Co-Investigator	
Yola	El Dahr			Hamilton Health Sciences/St. Joseph's Healthcare Hamilton - St. Joseph's Healthcare Hamilton	Canada	Coordinator	
Faraaz	Quraishi			Hamilton Health Sciences/St. Joseph's Healthcare Hamilton - St. Joseph's Healthcare Hamilton	Canada	Researcher	
Laura	Tomat			Hamilton Health Sciences/St. Joseph's Healthcare Hamilton - St. Joseph's Healthcare Hamilton	Canada	Coordinator	
Spencer	Wikkerink			Hamilton Health Sciences/St. Joseph's Healthcare Hamilton - St. Joseph's Healthcare Hamilton	Canada	Coordinator	
David	Laflamme			Hôpital Charles Lemoyne	Canada	Principal Investigator	
Miriam	Schnorr Meloche			Hôpital Charles Lemoyne	Canada	Coordinator	
Christel	Simard			Hôpital Charles Lemoyne	Canada	Coordinator	
Lise	Blais			Hôpital Charles Lemoyne	Canada	Coordinator	

Supplemental Online Content: Nonauthor Collaborators

*Indicates required information. Only first name, last name, and suffix will appear in PubMed.

*First Name and Middle Initial(s)	*Last Name	*Suffix (eg, Jr, III)	Academic Degrees	Institution	Location (city, state/province, country)	Role or Contribution, eg, chair, principal investigator	Group (if more than 1 Group listed in the byline) and/or Subgroup (eg, Steering Committee)
Jean-Pierre	Dery			Quebec Heart and Lung Institute	Canada	Principal Investigator	
Christina	Avena-Cyr			Quebec Heart and Lung Institute	Canada	Coordinator	
Melanie	Roy			Quebec Heart and Lung Institute	Canada	Coordinator	
Micheline	Charron			Quebec Heart and Lung Institute	Canada	Coordinator	
Kevin	Bainey			University Of Alberta Hospital	Canada	Principal Investigator	
Norma	Hogg			University Of Alberta Hospital	Canada	Coordinator	
Suzanne	Welshsan			University Of Alberta Hospital	Canada	Coordinator	
Richard	Haichin			Royal Victoria Hospital	Canada	Principal Investigator	
Coralie	Gasc			Royal Victoria Hospital	Canada	Coordinator	
Reza	Sahebjamei			Royal Victoria Hospital	Canada	Coordinator	
Fatemeh	Vaezi-Poor			Royal Victoria Hospital	Canada	Researcher	
Payam	Dehghani			Regina General Hospital	Canada	Principal Investigator	
Sabiha	Sultana			Regina General Hospital	Canada	Coordinator	
Ata Ur Rehman	Quraishi			QE II Health Sciences Center	Canada	Principal Investigator	
Hussein	Beydoun			QE II Health Sciences Center	Canada	Co-Investigator	
David	Fillmore			QE II Health Sciences Center	Canada	Coordinator	
Deborah	Keller			QE II Health Sciences Center	Canada	Coordinator	
Nick	Bartlett			QE II Health Sciences Center	Canada	Coordinator	
Brian J.	Potter			Centre Hosp. Universitaire de Montreal	Canada	Principal Investigator	
François M.	Carrier			Centre Hosp. Universitaire de Montreal	Canada	Principal Investigator	

*Indicates required information. Only first name, last name, and suffix will appear in PubMed.

*First Name and Middle Initial(s)	*Last Name	*Suffix (eg, Jr, III)	Academic Degrees	Institution	Location (city, state/province, country)	Role or Contribution, eg, chair, principal investigator	Group (if more than 1 Group listed in the byline) and/or Subgroup (eg, Steering Committee)
Fatna	Benettaib			Centre Hosp. Universitaire de Montreal	Canada	Coordinator	
Adriana	Carbonaro			Centre Hosp. Universitaire de Montreal	Canada	Researcher	
Isabelle	Chausse			Centre Hosp. Universitaire de Montreal	Canada	Researcher	
Katherine	Coutu-Beaudry			Centre Hosp. Universitaire de Montreal	Canada	Coordinator	
Maya	Hatwik			Centre Hosp. Universitaire de Montreal	Canada	Research Staff	
Martine	Lebrasseur			Centre Hosp. Universitaire de Montreal	Canada	Coordinator	
Cindy	Prié			Centre Hosp. Universitaire de Montreal	Canada	Coordinator	
Caroline	Vallières			Centre Hosp. Universitaire de Montreal	Canada	Coordinator	
Dounia	Boumahni			Centre Hosp. Universitaire de Montreal	Canada	Coordinator	
Genevieve	Dallaire			Centre Hosp. Universitaire de Montreal	Canada	Coordinator	
Renée	Duclos			Centre Hosp. Universitaire de Montreal	Canada	Coordinator	
Ali	Ghamraoui			Centre Hosp. Universitaire de Montreal	Canada	Coordinator	
Mélissa	Gagnon-Hamelin			Centre Hosp. Universitaire de Montreal	Canada	Coordinator	
Emilie	Sau			Centre Hosp. Universitaire de Montreal	Canada	Researcher	
Michael	Goldfarb			Jewish General Hospital	Canada	Principal Investigator	
Geraldine	Ricafort			Jewish General Hospital	Canada	Coordinator	

Supplemental Online Content: Nonauthor Collaborators

*Indicates required information. Only first name, last name, and suffix will appear in PubMed.

***First Name and Middle Initial(s)**	***Last Name**	***Suffix (eg, Jr, III)**	Academic Degrees	Institution	Location (city, state/province, country)	Role or Contribution, eg, chair, principal investigator	Group (if more than 1 Group listed in the byline) and/or Subgroup (eg, Steering Committee)
Claudine	Robert			Jewish General Hospital	Canada	Researcher	
Sophie	Florencio			Jewish General Hospital	Canada	Researcher	
Christopher	Fordyce			Vancouver General Hospital	Canada	Principal Investigator	
Jackie	Chow			Vancouver General Hospital	Canada	Coordinator	
Reilly	Ische			Vancouver General Hospital	Canada	Coordinator	
Ngaire	Meadows			Vancouver General Hospital	Canada	Researcher	
Andrew	Starovoytov			Vancouver General Hospital	Canada	Coordinator	
Sydney	Thorsteinsson			Vancouver General Hospital	Canada	Researcher	
Naomi	Uchida			Vancouver General Hospital	Canada	Researcher	
Lily	Cai			Vancouver General Hospital	Canada	Coordinator	
Shirley	Lim			Vancouver General Hospital	Canada	Researcher	
Jenny	Petterson			Vancouver General Hospital	Canada	Coordinator	
Himantha	De Silva			Vancouver General Hospital	Canada	Coordinator	
Stella	Chan			Vancouver General Hospital	Canada	Coordinator	
Celine	Yan			Vancouver General Hospital	Canada	Coordinator	
Ying Tung	Sia			Centre integre universitaire de sante et de services sociaux de la Mauricie-et-du-Centre-du-Quebec	Canada	Principal Investigator	
Miguel	Barrero			Centre integre universitaire de sante et de services sociaux de la Mauricie-et-du-Centre-du-Quebec	Canada	Co-Investigator	
Ricardo	Costa			Centre integre universitaire de sante et de services sociaux de la Mauricie-et-du-Centre-du-Quebec	Canada	Co-Investigator	

Supplemental Online Content: Nonauthor Collaborators

*Indicates required information. Only first name, last name, and suffix will appear in PubMed.

***First Name and Middle Initial(s)**	***Last Name**	***Suffix (eg, Jr, III)**	Academic Degrees	Institution	Location (city, state/province, country)	Role or Contribution, eg, chair, principal investigator	Group (if more than 1 Group listed in the byline) and/or Subgroup (eg, Steering Committee)
Ariel	Diaz			Centre integre universitaire de sante et de services sociaux de la Mauricie-et-du-Centre-du-Quebec	Canada	Co-Investigator	
Martin	Faucher			Centre integre universitaire de sante et de services sociaux de la Mauricie-et-du-Centre-du-Quebec	Canada	Researcher	
Carl-Éric	Gagné			Centre integre universitaire de sante et de services sociaux de la Mauricie-et-du-Centre-du-Quebec	Canada	Co-Investigator	
Jean-François	Naud			Centre integre universitaire de sante et de services sociaux de la Mauricie-et-du-Centre-du-Quebec	Canada	Co-Investigator	
Philippe	Rheault			Centre integre universitaire de sante et de services sociaux de la Mauricie-et-du-Centre-du-Quebec	Canada	Co-Investigator	
Yanek	Pépin-Dubois			Centre integre universitaire de sante et de services sociaux de la Mauricie-et-du-Centre-du-Quebec	Canada	Researcher	
Vincent	Spagnoli			Centre integre universitaire de sante et de services sociaux de la Mauricie-et-du-Centre-du-Quebec	Canada	Researcher	

Supplemental Online Content: Nonauthor Collaborators

*Indicates required information. Only first name, last name, and suffix will appear in PubMed.

***First Name and Middle Initial(s)**	***Last Name**	***Suffix (eg, Jr, III)**	Academic Degrees	Institution	Location (city, state/province, country)	Role or Contribution, eg, chair, principal investigator	Group (if more than 1 Group listed in the byline) and/or Subgroup (eg, Steering Committee)
Isabelle	Roy			Centre integre universitaire de sante et de services sociaux de la Mauricie-et-du-Centre-du-Quebec	Canada	Coordinator	
Benoit	Daneault			Centre Hospitalier Sherbrooke	Canada	Principal Investigator	
Julie	Caron			Centre Hospitalier Sherbrooke	Canada	Coordinator	
Mina	Madan			Sunnybrook Health Sciences Centre	Canada	Principal Investigator	
Sara	Jabeen			Sunnybrook Health Sciences Centre	Canada	Coordinator	
Nidhya Lakshmi	Thulasiraj			Sunnybrook Health Sciences Centre	Canada	Researcher	
Norberto	Garcia			Sunnybrook Health Sciences Centre	Canada	Coordinator	
Suneet	Khurana			Sunnybrook Health Sciences Centre	Canada	Coordinator	
Terry	McPherson			London Health Sciences Centre - Victoria Hospital - LHSC , University Hospital	Canada	Principal Investigator	
Rehana	Bajwa			London Health Sciences Centre - Victoria Hospital - LHSC , University Hospital	Canada	Researcher	
Cassie	Wagner			London Health Sciences Centre - Victoria Hospital - LHSC , University Hospital	Canada	Coordinator	
John	Ducas			St. Boniface General Hospital	Canada	Principal Investigator	
Kunal	Minhas			St. Boniface General Hospital	Canada	Principal Investigator	
Kiran	Atwal			St. Boniface General Hospital	Canada	Coordinator	

Supplemental Online Content: Nonauthor Collaborators

*Indicates required information. Only first name, last name, and suffix will appear in PubMed.

*First Name and Middle Initial(s)	*Last Name	*Suffix (eg, Jr, III)	Academic Degrees	Institution	Location (city, state/province, country)	Role or Contribution, eg, chair, principal investigator	Group (if more than 1 Group listed in the byline) and/or Subgroup (eg, Steering Committee)
Malek	Kass			St. Boniface General Hospital	Canada	Co-Investigator	
Stephen Allan	Schaffer			St. Boniface General Hospital	Canada	Co-Investigator	
Neil	Brass			Royal Alexandra Hospital	Canada	Principal Investigator	
Rebecca	Cairns			Royal Alexandra Hospital	Canada	Researcher	
Anna	Law			Royal Alexandra Hospital	Canada	Researcher	
Akshay	Bagai			St. Michael's Hospital	Canada	Principal Investigator	
Mona	Doorsian			St. Michael's Hospital	Canada	Coordinator	
Neil	Fam			St. Michael's Hospital	Canada	Co-Investigator	
Jan	Friedrich			St. Michael's Hospital	Canada	Co-Investigator	
Andrew	Baker			St. Michael's Hospital	Canada	Researcher	
John	Graham			St. Michael's Hospital	Canada	Coordinator	
Michael	Kutryk			St. Michael's Hospital	Canada	Co-Investigator	
Gyan	Sandhu			St. Michael's Hospital	Canada	Coordinator	
Imrana	Khalid			St. Michael's Hospital	Canada	Coordinator	
Marlene	Santos			St. Michael's Hospital	Canada	Researcher	
Simon	Robinson			Victoria Heart Institute	Canada	Principal Investigator	
Kim	Allen			Victoria Heart Institute	Canada	Researcher	
Noreen	Lounsbury			Victoria Heart Institute	Canada	Coordinator	
Sheryll	Sorensen			Victoria Heart Institute	Canada	Coordinator	
Elizabeth	Martin			Victoria Heart Institute	Canada	Coordinator	
Vladimír	Džavík			UHN	Canada	Principal Investigator	
Anna	Tran			UHN	Canada	Coordinator	
Razi	Khan			Royal Columbian Hospital	Canada	Principal Investigator	
Nicolas	Michaud			Hotel Dieu de Levis	Canada	Principal Investigator	

*Indicates required information. Only first name, last name, and suffix will appear in PubMed.

***First Name and Middle Initial(s)**	***Last Name**	***Suffix (eg, Jr, III)**	Academic Degrees	Institution	Location (city, state/province, country)	Role or Contribution, eg, chair, principal investigator	Group (if more than 1 Group listed in the byline) and/or Subgroup (eg, Steering Committee)
Philippe Gabriel	Steg			Hôpital BICHAT - APHP ; Université Paris-Cité, INSERM U-1148	France	Principal Investigator	Executive Committee, Steering Committee, EU Clinical Coordinating Center
Gregory	Ducrocq			Hôpital BICHAT - APHP ; Université Paris-Cité, INSERM U-1148	France	Principal Investigator	Clinical Events Committee, EU Clinical Coordinating Center
Arthur	Darmon			Hôpital BICHAT - APHP	France	Co-Investigator	
Quentin	Fischer			Hôpital BICHAT - APHP	France	Co-Investigator	
Neila	Sayah			Hôpital BICHAT - APHP	France	Co-Investigator	
Hélène	Abergel			Hôpital BICHAT - APHP	France	Co-Investigator	
Catherine	Bouffard			Hôpital BICHAT - APHP	France	Co-Investigator	
Audrey	Cailliau			Hôpital BICHAT - APHP	France	Co-Investigator	
Axelle	Fuentes			Hôpital BICHAT - APHP	France	Co-Investigator	
Nicolas	Pham			Hôpital BICHAT - APHP	France	Researcher	
Alexandre	Gautier			Hôpital BICHAT - APHP	France	Researcher	
Jules	Mesnier			Hôpital BICHAT - APHP	France	Researcher	
Etienne	Puymirat			HEGP - APHP	France	Principal Investigator	
Mélissa	Sriwaran			HEGP - APHP	France	Researcher	
Rachel	Elysee			HEGP - APHP	France	Co-Investigator	
Antoine	Fayol			HEGP - APHP	France	Co-Investigator	
Amandine	Grelier			HEGP - APHP	France	Co-Investigator	
Nesrine	Hafsi			HEGP - APHP	France	Co-Investigator	
Hasina	Rakotosamimanana			HEGP - APHP	France	Co-Investigator	
Victoria	Tea			HEGP - APHP	France	Co-Investigator	
Sarah	Arab			HEGP - APHP	France	Researcher	
Hinde	Irekti			HEGP - APHP	France	Researcher	
Gilles	Lemesle			CHRU Lille	France	Principal Investigator	

Supplemental Online Content: Nonauthor Collaborators

*Indicates required information. Only first name, last name, and suffix will appear in PubMed.

*First Name and Middle Initial(s)	*Last Name	*Suffix (eg, Jr, III)	Academic Degrees	Institution	Location (city, state/province, country)	Role or Contribution, eg, chair, principal investigator	Group (if more than 1 Group listed in the byline) and/or Subgroup (eg, Steering Committee)
Anne	Druart			CHRU Lille	France	Co-Investigator	
Isabelle	Pilat			CHRU Lille	France	Co-Investigator	
Basile	Verdier			CHRU Lille	France	Co-Investigator	
Emile	Ferrari			CHU Nice - Hôpital Pasteur	France	Principal Investigator	
Etienne	Fourrier			CHU Nice - Hôpital Pasteur	France	Co-Investigator	
Mohamed	Labbaoui			CHU Nice - Hôpital Pasteur	France	Co-Investigator	
Nassim	Redjimi			CHU Nice - Hôpital Pasteur	France	Co-Investigator	
Amina	Zamiti-Smondel			CHU Nice - Hôpital Pasteur	France	Co-Investigator	
Rabab	Bencherif			CHU Nice - Hôpital Pasteur	France	Researcher	
Benoit	Lattuca			CHU Caremeau - Nîmes	France	Principal Investigator	
Elodie	Delelo			CHU Caremeau - Nîmes	France	Co-Investigator	
Johanne	Silvain			Hôpital Pitié Salpétrière - APHP	France	Principal Investigator	
Olivier	Barthelemy			Hôpital Pitié Salpétrière - APHP	France	Co-Investigator	
Fatima	Benattia			Hôpital Pitié Salpétrière - APHP	France	Co-Investigator	
Perrine	Devos			Hôpital Pitié Salpétrière - APHP	France	Co-Investigator	
Soraya	Merbah			Hôpital Pitié Salpétrière - APHP	France	Co-Investigator	
Juliane	Posson			Hôpital Pitié Salpétrière - APHP	France	Co-Investigator	
Nikki	Procopi			Hôpital Pitié Salpétrière - APHP	France	Co-Investigator	
Thomas	Wallet			Hôpital Pitié Salpétrière - APHP	France	Co-Investigator	
Michel	Zeitouni			Hôpital Pitié Salpétrière - APHP	France	Co-Investigator	
Arnaud	Ferrante			Hôpital Pitié Salpétrière - APHP	France	Researcher	
Camille	Granger			Hôpital Pitié Salpétrière - APHP	France	Researcher	
Stéphane	Manzo-Silberman			Hôpital Pitié Salpétrière - APHP	France	Researcher	
Tomy	Salloum			Hôpital Pitié Salpétrière - APHP	France	Researcher	
Gérald	Vanzetto			Hôpital Michalon - Grenoble	France	Principal Investigator	

*Indicates required information. Only first name, last name, and suffix will appear in PubMed.

*First Name and Middle Initial(s)	*Last Name	*Suffix (eg, Jr, III)	Academic Degrees	Institution	Location (city, state/province, country)	Role or Contribution, eg, chair, principal investigator	Group (if more than 1 Group listed in the byline) and/or Subgroup (eg, Steering Committee)
Gilles	Barone Rochette			Hôpital Michalon - Grenoble	France	Co-Investigator	
Sara	Blanc Vannet			Hôpital Michalon - Grenoble	France	Co-Investigator	
Clémence	Charlon			Hôpital Michalon - Grenoble	France	Co-Investigator	
Océane	Crebier			Hôpital Michalon - Grenoble	France	Co-Investigator	
Stéphanie	Marliere			Hôpital Michalon - Grenoble	France	Co-Investigator	
Pauline	Peretout			Hôpital Michalon - Grenoble	France	Co-Investigator	
Julie	Hildt			Hôpital Michalon - Grenoble	France	Researcher	
Laura	Cetran			Hôpital Haut Lévêque - Pessac	France	Principal Investigator	
Thomas	Baudinet			Hôpital Haut Lévêque - Pessac	France	Co-Investigator	
Pierre	Coste			Hôpital Haut Lévêque - Pessac	France	Co-Investigator	
Amandine	Ruissel			Hôpital Haut Lévêque - Pessac	France	Co-Investigator	
Marine	Servat			Hôpital Haut Lévêque - Pessac	France	Researcher	
Thibault	Lhermusier			CHU Rangueil - Toulouse	France	Principal Investigator	
Frédéric	Bouisset			CHU Rangueil - Toulouse	France	Co-Investigator	
Francisco	Campelo-Parada			CHU Rangueil - Toulouse	France	Co-Investigator	
Kimberly	Lemoine			CHU Rangueil - Toulouse	France	Co-Investigator	
Mouin	Nasr			CHU Rangueil - Toulouse	France	Co-Investigator	
Marine	Poisay			CHU Rangueil - Toulouse	France	Co-Investigator	
Clément	Servoz			CHU Rangueil - Toulouse	France	Co-Investigator	
Stéphanie	Tribeau			CHU Rangueil - Toulouse	France	Co-Investigator	
Yves	Cottin			CHU le Bocage - Dijon	France	Principal Investigator	
Florence	Bichat			CHU le Bocage - Dijon	France	Coordinator	
Mailis	Saint-Jalmes			CHU le Bocage - Dijon	France	Co-Investigator	
Victor	Buzura			Centre Hospitalier sud francilien	France	Principal Investigator	

Supplemental Online Content: Nonauthor Collaborators

*Indicates required information. Only first name, last name, and suffix will appear in PubMed.

*First Name and Middle Initial(s)	*Last Name	*Suffix (eg, Jr, III)	Academic Degrees	Institution	Location (city, state/province, country)	Role or Contribution, eg, chair, principal investigator	Group (if more than 1 Group listed in the byline) and/or Subgroup (eg, Steering Committee)
Yann	Rosamel			Centre Hospitalier sud francilien	France	Principal Investigator	
Sarah	Hadjih			Centre Hospitalier sud francilien	France	Co-Investigator	
Denis	Angoulvant			CHRU Tours - Hôpital Trousseau	France	Principal Investigator	
Emilie	Collet			CHRU Tours - Hôpital Trousseau	France	Co-Investigator	
Fabrice	Ivanes			CHRU Tours - Hôpital Trousseau	France	Co-Investigator	
Katia	Lassouani			CHRU Tours - Hôpital Trousseau	France	Co-Investigator	
Mohammed	Mouzouri			CHRU Tours - Hôpital Trousseau	France	Co-Investigator	
Djedjiga	Naudin			CHRU Tours - Hôpital Trousseau	France	Co-Investigator	
Thibaud	Genet			CHRU Tours - Hôpital Trousseau	France	Researcher	
Jean Guillaume	Dillinger			Hôpital Lariboisière - APHP	France	Principal Investigator	
Nadhira	Bennacer			Hôpital Lariboisière - APHP	France	Co-Investigator	
Lea	Cacoub			Hôpital Lariboisière - APHP	France	Co-Investigator	
Antoine	Lequipar			Hôpital Lariboisière - APHP	France	Co-Investigator	
Baya	Mahtout			Hôpital Lariboisière - APHP	France	Co-Investigator	
Christophe	Thuaire			CH Louis Pasteur, Chartres	France	Principal Investigator	
Marina	Leniliot			CH Louis Pasteur, Chartres	France	Researcher	
Emilie	Tachot			CH Louis Pasteur, Chartres	France	Co-Investigator	
Corine	Thobois			CH Louis Pasteur, Chartres	France	Co-Investigator	
Christel	Vassaliere			CH Louis Pasteur, Chartres	France	Researcher	
Batric	Popovic			CHU Nancy	France	Principal Investigator	
Jessica	Breistroff			CHU Nancy	France	Co-Investigator	
Aurelie	Gutehrle			CHU Nancy	France	Co-Investigator	
Maximilien	Saint-Gilles			CHU Nancy	France	Co-Investigator	
Jeanne	Varlot			CHU Nancy	France	Co-Investigator	

*Indicates required information. Only first name, last name, and suffix will appear in PubMed.

*First Name and Middle Initial(s)	*Last Name	*Suffix (eg, Jr, III)	Academic Degrees	Institution	Location (city, state/province, country)	Role or Contribution, eg, chair, principal investigator	Group (if more than 1 Group listed in the byline) and/or Subgroup (eg, Steering Committee)
Eric	Durand			CHU de Rouen	France	Principal Investigator	
Delphine	Beziau-Gasnier			CHU de Rouen	France	Coordinator	
Najime	Bouhzam			CHU de Rouen	France	Co-Investigator	
Solenn	Catherine			CHU de Rouen	France	Co-Investigator	
Bryan	Marais			CHU de Rouen	France	Co-Investigator	
Christophe	Tron			CHU de Rouen	France	Co-Investigator	
Claire	Vezier			CHU de Rouen	France	Co-Investigator	
Thibaut	Hemery			CHU de Rouen	France	Researcher	
Claire	Bouleti			CHU Poitiers	France	Principal Investigator	
Arthur	Ramonatxo			CHU Poitiers	France	Co-Investigator	
Sonia	Azzakani			CHU Poitiers	France	Co-Investigator	
Nathalie	Berger			CHU Poitiers	France	Co-Investigator	
Celine	Boucher			CHU Poitiers	France	Co-Investigator	
François	Roubille			CHU Arnaud de Villeneuve - Montpellier	France	Principal Investigator	
Chloé	Bonneton			CHU Arnaud de Villeneuve - Montpellier	France	Researcher	
Gautier	Desmoulin			CHU Arnaud de Villeneuve - Montpellier	France	Researcher	
Fabien	Huet			CHU Arnaud de Villeneuve - Montpellier	France	Researcher	
Sandra	Kahlouche			CHU Arnaud de Villeneuve - Montpellier	France	Coordinator	
Laurent	Delorme			Clinique du Pont de Chaume - Montauban	France	Principal Investigator	
Angélique	Andrieu			Clinique du Pont de Chaume - Montauban	France	Co-Investigator	
Javier	Molina-Martin De Nicolas			Clinique du Pont de Chaume - Montauban	France	Co-Investigator	

Supplemental Online Content: Nonauthor Collaborators

*Indicates required information. Only first name, last name, and suffix will appear in PubMed.

*First Name and Middle Initial(s)	*Last Name	*Suffix (eg, Jr, III)	Academic Degrees	Institution	Location (city, state/province, country)	Role or Contribution, eg, chair, principal investigator	Group (if more than 1 Group listed in the byline) and/or Subgroup (eg, Steering Committee)
Nathalie	Durand			Clinique du Pont de Chaume - Montauban	France	Researcher	
Marion	Berous			Clinique du Pont de Chaume - Montauban	France	Researcher	
Romain	André			Clinique du Pont de Chaume - Montauban	France	Researcher	
Ian	Crozier			Christchurch Hospital	New Zealand	Principal Investigator	
John	Elliott			Christchurch Hospital	New Zealand	Principal Investigator	
Catherine	Cruickshank			Christchurch Hospital	New Zealand	Coordinator	
Houda	El Banna			Christchurch Hospital	New Zealand	Researcher	
Michael	Hume			Christchurch Hospital	New Zealand	Researcher	
Jocelyne	Benatar			Auckland Hospital	New Zealand	Principal Investigator	
Jithendra	Somaratne			Auckland Hospital	New Zealand	Researcher	
Diana	Gatland			Auckland Hospital	New Zealand	Researcher	
Danica	Kistanna			Auckland Hospital	New Zealand	Coordinator	
Cathrine	Patten			Auckland Hospital	New Zealand	Researcher	
Doreen	Singh			Auckland Hospital	New Zealand	Researcher	
Samraj	Nandra			Whangarei Hospital	New Zealand	Principal Investigator	
Chuen Siang	Low			Whangarei Hospital	New Zealand	Co-Investigator	
Susan	Vallancey			Whangarei Hospital	New Zealand	Researcher	
Ian	Ternouth			Taranaki Hospital	New Zealand	Principal Investigator	
Neelam	Dalman			Taranaki Hospital	New Zealand	Co-Investigator	
Aleisha	Easton			Taranaki Hospital	New Zealand	Co-Investigator	
Carolyn	Jackson			Taranaki Hospital	New Zealand	Researcher	
Simon	Kirkham			Taranaki Hospital	New Zealand	Researcher	
Cathy	Vickers			Taranaki Hospital	New Zealand	Researcher	

*Indicates required information. Only first name, last name, and suffix will appear in PubMed.

*First Name and Middle Initial(s)	*Last Name	*Suffix (eg, Jr, III)	Academic Degrees	Institution	Location (city, state/province, country)	Role or Contribution, eg, chair, principal investigator	Group (if more than 1 Group listed in the byline) and/or Subgroup (eg, Steering Committee)
Nick	Fisher			Nelson Hospital	New Zealand	Principal Investigator	
Jette	Koelle			Nelson Hospital	New Zealand	Researcher	
Charlotte	McNab			Nelson Hospital	New Zealand	Researcher	
David	Brieger			Concord Repatriation General Hospital	Australia	Principal Investigator	
Shahab	Pathan			Concord Repatriation General Hospital	Australia	Researcher	
Imants	Rubenis			Concord Repatriation General Hospital	Australia	Researcher	
Su	Swe			Concord Repatriation General Hospital	Australia	Researcher	
Sohaib	Virk			Concord Repatriation General Hospital	Australia	Researcher	
Graham	Hillis			Royal Perth Hospital	Australia	Principal Investigator	
Michelle	Bonner			Royal Perth Hospital	Australia	Researcher	
Charmaine	Dias			Royal Perth Hospital	Australia	Researcher	
Lorraine	Hillis			Royal Perth Hospital	Australia	Researcher	
Maria Mori	Brooks			University of Pittsburgh	United States	Principal Investigator	Executive Committee, Steering Committee, and Data Coordinating Center
John H.	Alexander			Duke Clinical Research Institute, Duke University	United States	Executive Committee	Executive Committee and Steering Committee
Shaun G.	Goodman			St. Michael's Hospital, University of Toronto	Canada	Executive Committee	Executive Committee and Steering Committee

*Indicates required information. Only first name, last name, and suffix will appear in PubMed.

*First Name and Middle Initial(s)	*Last Name	*Suffix (eg, Jr, III)	Academic Degrees	Institution	Location (city, state/province, country)	Role or Contribution, eg, chair, principal investigator	Group (if more than 1 Group listed in the byline) and/or Subgroup (eg, Steering Committee)
Paul C.	Hébert			Centre de recherche du Centre hospitalier de l'Université de Montréal	Canada	Principal Investigator	Executive Committee, Steering Committee, and Canadian Coordinating Center
Renato D.	Lopes			Brazilian Clinical Research Institute (BCRI) and Duke Clinical Research Institute	Brazil	Principal Investigator	Executive Committee, Steering Committee, Clinical Events Committee, Brazilian Clinical Coordinating Center
Tabassome	Simon			Hopital St Antoine;<br>Sorbonne Université, INSERM U-1148	France	Principal Investigator	Executive Committee, Steering Committee, EU Clinical Coordinating Center
Harvey	White			Green Lane Coordinating Centre Ltd	New Zealand	Principal Investigator	New Zealand Coordinating Center, Executive Committee, Steering Committee
Caroline	Alsweiler			Green Lane Coordinating Centre Ltd	New Zealand	Coordinator	New Zeeland Coordinating Center and Steering Committee
Bernard R.	Chaitman			St Louis University	United States	Principal Investigator	Clinical Events Committee Chair, Steering Committee
Dean A.	Fergusson			Ottawa Hospital Research Institute	Canada	Steering Committee	Steering Committee
Erin	Morton			Finders University	Australia	Coordinator	Australian Coordinating Center and Steering Committee

Supplemental Online Content: Nonauthor Collaborators

*Indicates required information. Only first name, last name, and suffix will appear in PubMed.

***First Name and Middle Initial(s)**	***Last Name**	***Suffix (eg, Jr, III)**	Academic Degrees	Institution	Location (city, state/province, country)	Role or Contribution, eg, chair, principal investigator	Group (if more than 1 Group listed in the byline) and/or Subgroup (eg, Steering Committee)
Darrell J.	Triulzi			University of Pittsburgh	United States	Steering Committee	Steering Committee
Lilian Mazza	Barbosa			Brazilian Clinical Research Institute (BCRI)	Brazil	Researcher	Brazil: Brazilian Clinical Research Institute
Pedro Gabriel Melo	de Barros e Silva			Brazilian Clinical Research Institute (BCRI)	Brazil	Researcher	Brazilian Clinical Coordinating Center and Clinical Events Committee
Liliane A T	Arnaldi Seixas			Brazilian Clinical Research Institute (BCRI)	Brazil	Researcher	Brazil: Brazilian Clinical Research Institute
Paola E.	Arantes			Brazilian Clinical Research Institute (BCRI)	Brazil	Researcher	Brazil: Brazilian Clinical Research Institute
Larissa	Teixeira Aleixo Silva			Brazilian Clinical Research Institute (BCRI)	Brazil	Data Management	Brazil: Brazilian Clinical Research Institute
Georgia	Beatriz Oliveira Damásio			Brazilian Clinical Research Institute (BCRI)	Brazil	Researcher	Brazil: Brazilian Clinical Research Institute
Laurence	Berard			APHP, Plateforme de Recherche Clinique de l'Est Parisien (URCEST-CRCEST-CRB)	France	Coordinator	EU Clinical Coordinating center
Alexandra	Rousseau			APHP, Plateforme de Recherche Clinique de l'Est Parisien (URCEST-CRCEST-CRB)	France	Data Management	EU Clinical Coordinating center
Sandra	Paco			APHP, Plateforme de Recherche Clinique de l'Est Parisien (URCEST-CRCEST-CRB)	France	Project Manager	EU Clinical Coordinating center
Narimane	Benhamadi			APHP, Plateforme de Recherche Clinique de l'Est Parisien (URCEST-CRCEST-CRB)	France	Researcher	EU Clinical Coordinating center

*Indicates required information. Only first name, last name, and suffix will appear in PubMed.

*First Name and Middle Initial(s)	*Last Name	*Suffix (eg, Jr, III)	Academic Degrees	Institution	Location (city, state/province, country)	Role or Contribution, eg, chair, principal investigator	Group (if more than 1 Group listed in the byline) and/or Subgroup (eg, Steering Committee)
Véléda	Blesboi			APHP, Plateforme de Recherche Clinique de l'Est Parisien (URCEST-CRCEST-CRB)	France	Researcher	EU Clinical Coordinating center
Marie	Delon			APHP, Plateforme de Recherche Clinique de l'Est Parisien (URCEST-CRCEST-CRB)	France	Researcher	EU Clinical Coordinating center
Alexia	Ep			APHP, Plateforme de Recherche Clinique de l'Est Parisien (URCEST-CRCEST-CRB)	France	Researcher	EU Clinical Coordinating center
Kaïna	Mahmoudi			APHP, Plateforme de Recherche Clinique de l'Est Parisien (URCEST-CRCEST-CRB)	France	Researcher	EU Clinical Coordinating center
Katia	Ledra			APHP, Plateforme de Recherche Clinique de l'Est Parisien (URCEST-CRCEST-CRB)	France	Researcher	EU Clinical Coordinating center
Irène	Garcia Ramos			APHP, Plateforme de Recherche Clinique de l'Est Parisien (URCEST-CRCEST-CRB)	France	Researcher	EU Clinical Coordinating center
Marie	Henault			APHP, Plateforme de Recherche Clinique de l'Est Parisien (URCEST-CRCEST-CRB)	France	Researcher	EU Clinical Coordinating center
Alexia	Dantigny			APHP, Plateforme de Recherche Clinique de l'Est Parisien (URCEST-CRCEST-CRB)	France	Researcher	EU Clinical Coordinating center
Aichetou	Kone			APHP, Plateforme de Recherche Clinique de l'Est Parisien (URCEST-CRCEST-CRB)	France	Researcher	EU Clinical Coordinating center
Djouher	Firoud			APHP, Plateforme de Recherche Clinique de l'Est Parisien (URCEST-CRCEST-CRB)	France	Researcher	EU Clinical Coordinating center
Sarah	Arab			APHP, Plateforme de Recherche Clinique de l'Est Parisien (URCEST-CRCEST-CRB)	France	Researcher	EU Clinical Coordinating center
Federica	Scialo			APHP, Plateforme de Recherche Clinique de l'Est Parisien (URCEST-CRCEST-CRB)	France	Researcher	EU Clinical Coordinating center

Supplemental Online Content: Nonauthor Collaborators

*Indicates required information. Only first name, last name, and suffix will appear in PubMed.

***First Name and Middle Initial(s)**	***Last Name**	***Suffix (eg, Jr, III)**	Academic Degrees	Institution	Location (city, state/province, country)	Role or Contribution, eg, chair, principal investigator	Group (if more than 1 Group listed in the byline) and/or Subgroup (eg, Steering Committee)
Véronique	Pignot			APHP, Plateforme de Recherche Clinique de l'Est Parisien (URCEST-CRCEST-CRB)	France	Data Management	EU Clinical Coordinating center
Elodie	Drouet			APHP, Plateforme de Recherche Clinique de l'Est Parisien (URCEST-CRCEST-CRB)	France	Coordinator	EU Clinical Coordinating center
Eunice	Nubret			APHP, DRCI	France	Project Manager	EU Clinical Coordinating center
Margot	Bobin			APHP, DRCI	France	Researcher	EU Clinical Coordinating center
Christophe	Aucan			APHP, DRCI	France	Researcher	EU Clinical Coordinating center
Lauren	Demerville			APHP, DRCI	France	Legal	EU Clinical Coordinating center
Serge	Bureau			APHP, DRCI	France	Researcher	EU Clinical Coordinating center
Sarah	Preston			Green Lane Coordinating Centre	New Zealand	Researcher	New Zeeland Coordinating Center
Maria	Simula			Green Lane Coordinating Centre	New Zealand	Researcher	New Zeeland Coordinating Center
Derek	Chew			Flinders University	Australia	Principal Investigator	Australia Coordinating Center
Yogesh	Sharma			Flinders University	Australia	Principal Investigator	Australia Coordinating Center
Asha	Aravind			Flinders University	Australia	Researcher	Australia Coordinating Center
Christine	Hincks			Flinders University	Australia	Researcher	Australia Coordinating Center
Ivana	Hunt			Flinders University	Australia	Coordinator	Australia Coordinating Center
Simeoni	Thomas			Flinders University	Australia	Researcher	Australia Coordinating Center

Supplemental Online Content: Nonauthor Collaborators

*Indicates required information. Only first name, last name, and suffix will appear in PubMed.

*First Name and Middle Initial(s)	*Last Name	*Suffix (eg, Jr, III)	Academic Degrees	Institution	Location (city, state/province, country)	Role or Contribution, eg, chair, principal investigator	Group (if more than 1 Group listed in the byline) and/or Subgroup (eg, Steering Committee)
Fiona	Wollaston			Flinders University	Australia	Researcher	Australia Coordinating Center
Marnie	Bertolet			University of Pittsburgh	United States	Co-Investigator	Data Coordinating Center
Brandon	Herbert			University of Pittsburgh	United States	Data Analyst	Data Coordinating Center
Sheryl	Kelsey			University of Pittsburgh	United States	Co-Investigator	Data Coordinating Center
Manuel	Lombardero			University of Pittsburgh	United States	Data Analyst	Data Coordinating Center
Rocco	Mercurio			University of Pittsburgh	United States	Data Management	Data Coordinating Center
Gerard	Portela			University of Pittsburgh	United States	Data Analyst	Data Coordinating Center
Jennifer	Stevenson			University of Pittsburgh	United States	Coordinator	Data Coordinating Center
Donna	Stoliker			University of Pittsburgh	United States	Data Management	Data Coordinating Center
Ella	Zadorozny			University of Pittsburgh	United States	Data Management	Data Coordinating Center
Jane F.	Eckstein			St Louis University	United States	Researcher	Clinical Events Committee
Simone	Glynn			National Heart Lung and Blood Institute (NHLBI)	United States	NHLBI	
Eric	Leifer			National Heart Lung and Blood Institute (NHLBI)	United States	NHLBI	
Erin	Smith			National Heart Lung and Blood Institute (NHLBI)	United States	NHLBI	
George	Sopko			National Heart Lung and Blood Institute (NHLBI)	United States	NHLBI	

Supplemental Online Content: Nonauthor Collaborators

*Indicates required information. Only first name, last name, and suffix will appear in PubMed.

***First Name and Middle Initial(s)**	***Last Name**	***Suffix (eg, Jr, III)**	Academic Degrees	Institution	Location (city, state/province, country)	Role or Contribution, eg, chair, principal investigator	Group (if more than 1 Group listed in the byline) and/or Subgroup (eg, Steering Committee)
Phil	Tonkins			National Heart Lung and Blood Institute (NHLBI)	United States	NHLBI	
Pablo	Cure			National Heart Lung and Blood Institute (NHLBI)	United States	NHLBI	
Catherine	Levy			National Heart Lung and Blood Institute (NHLBI)	United States	NHLBI	
Naomi	Luban			Data and Safety Monitoring Board (DSMB)	United States	DSMB Chair	
Barbara	Konkle			Data and Safety Monitoring Board (DSMB)	United States	DSMB Vice Chair	
Deepak	Bhatt			Data and Safety Monitoring Board (DSMB)	United States	DSMB Member	
Eldad	Hod			Data and Safety Monitoring Board (DSMB)	United States	DSMB Member	
Cassandra	Josephson			Data and Safety Monitoring Board (DSMB)	United States	DSMB Member	
KyungMann	Kim			Data and Safety Monitoring Board (DSMB)	United States	DSMB Member	
Agnes	Lee			Data and Safety Monitoring Board (DSMB)	United States	DSMB Member	
Leslie	Serchuck			Data and Safety Monitoring Board (DSMB)	United States	DSMB Member	
Peter	Stone			Data and Safety Monitoring Board (DSMB)	United States	DSMB Member	
Darby	Thompson			Data and Safety Monitoring Board (DSMB)	United States	DSMB Member	
Kathryn	Weise			Data and Safety Monitoring Board (DSMB)	United States	DSMB Member	
