# Supplementary material for: Anemia Acuity Effect on Transfusion Strategies in Acute Myocardial Infarction: A Secondary Analysis of the MINT Trial
Source: JAMA Netw Open. 2024 Nov 1;7(11):e2442361. doi: 10.1001/jamanetworkopen.2024.42361 (PMC11530937; doi:10.1001/jamanetworkopen.2024.42361)
Supplement: Supplement 4. — Data Sharing Statement [file jamanetwopen-e2442361-s004.pdf]

Data Sharing Statement

Carrier. Anemia Acuity Effect on Transfusion Strategies in Acute Myocardial Infarction. *JAMA Netw Open*. Published November 01, 2024. doi:10.1001/jamanetworkopen.2024.42361

Data
Additional Information: NCT02981407 (ClinicalTrials.gov)
Data available: Yes
Data types: Deidentified participant data
How to access data: NIH Data Repository
When available: beginning date: 11-11-2025

Supporting Documents
Document types: Statistical/analytic code
How to access documents: Submitted with their manuscript
When available: With publication

Additional Information
Who can access the data: anyone requesting the data
Types of analyses: for any purpose
Mechanisms of data availability: Through NIH repository
